# A phylogenetic approach to study the origin and evolution of plasmodesmata-localized glycosyl hydrolases family 17

**DOI:** 10.3389/fpls.2014.00212

**Published:** 2014-05-23

**Authors:** Rocio Gaudioso-Pedraza, Yoselin Benitez-Alfonso

**Affiliations:** Centre for Plant Sciences, School of Biology, University of LeedsLeeds, UK

**Keywords:** plasmodesmata, callose regulation, GH17 domain, beta 1,3 glucanases, phylogenetic analysis

## Abstract

Colonization of the land by plants required major modifications in cellular structural composition and metabolism. Intercellular communication through plasmodesmata (PD) plays a critical role in the coordination of growth and cell activities. Changes in the form, regulation or function of these channels are likely linked to plant adaptation to the terrestrial environments. Constriction of PD aperture by deposition of callose is the best-studied mechanism in PD regulation. Glycosyl hydrolases family 17 (GHL17) are callose degrading enzymes. In Arabidopsis this is a large protein family, few of which have been PD-localized. The objective here is to identify correlations between evolution of this protein family and their role at PD and to use this information as a tool to predict the localization of candidates isolated in a proteomic screen. With this aim, we studied phylogenetic relationship between Arabidopsis GHL17 sequences and those isolated from fungi, green algae, mosses and monocot representatives. Three distinct phylogenetic clades were identified. Clade alpha contained only embryophytes sequences suggesting that this subgroup appeared during land colonization in organisms with functional PD. Accordingly, all PD-associated GHL17 proteins identified so far in *Arabidopsis thaliana* and Populus are grouped in this ‘embryophytes only’ phylogenetic clade. Next, we tested the use of this knowledge to discriminate between candidates isolated in the PD proteome. Transient and stable expression of GFP protein fusions confirmed PD localization for candidates contained in clade alpha but not for candidates contained in clade beta. Our results suggest that GHL17 membrane proteins contained in the alpha clade evolved and expanded during land colonization to play new roles, among others, in PD regulation.

## Introduction

**Figure 1:**
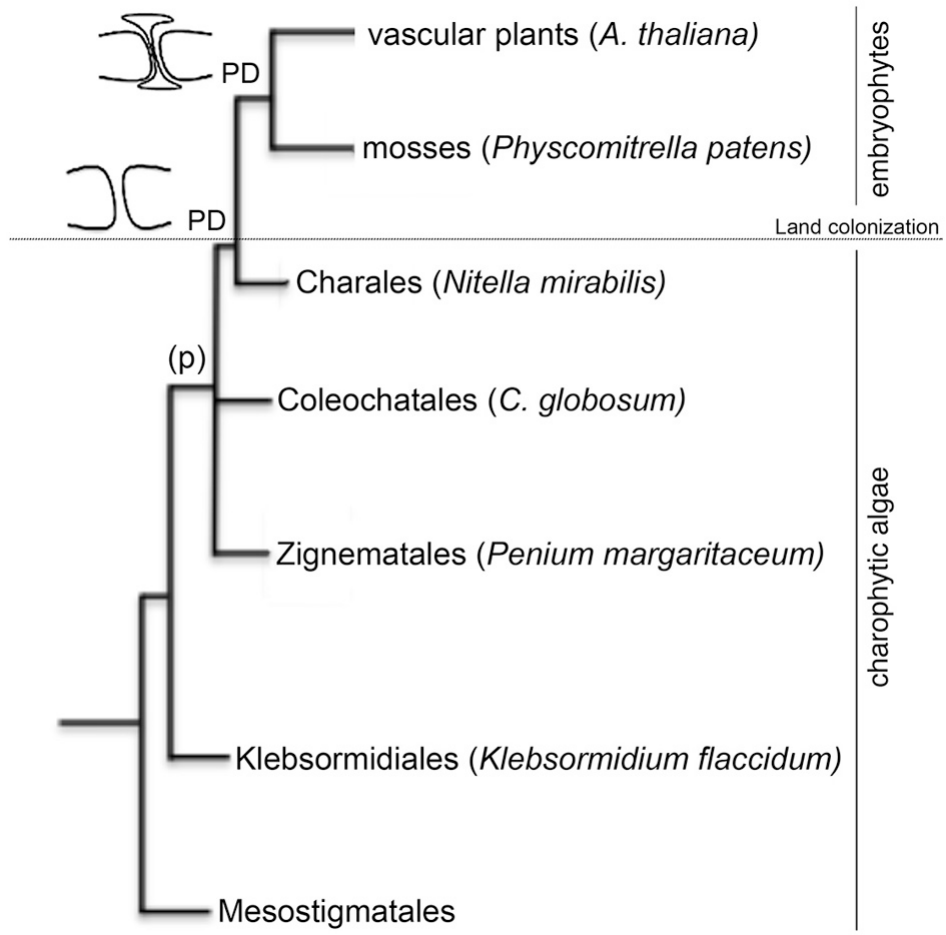
**Phylogenetic relationships of the species used in this study**. The cladogram is based on the current view of land plant evolution (Qiu, 2008). Members of the order Mesostigmatales, Klebsormidiales, Zygnematales, Coleochatales, and Charales form the charophytic green algae lineage (land plant ancestors). Representatives from these orders selected for this study are named in the figure. Embryophytes (such as the moss *Physcomitrella patents* and the vascular plant *Arabidopsis thaliana*) evolved from charophytic algae during land colonization. Phragmoplast (p) were found in organisms belonging to the Coleochatales and the Charales. Plasmodesmata (PD) appeared in all embryophytes.

Cell-to-cell communication is a requisite for the evolution of multicellular organisms. Plant intercellular connections (plasmodesmata, PD) are thought to originate with the appearance of multicellularity in green algae but their structural complexity increased, presumably, as a result of changes in cell-wall composition during adaptation to terrestrial environments (Lucas and Lee, 2004; Popper et al., 2011). Similarities between intercellular connections in charophytic algae and in early land plants suggest that they have a common evolutionary origin. Plasmodesmata occur in all embryophytes (including mosses) and, in their simplest form, also appear in representatives of charophytic green algae (Franceschi et al., 1994; Cook et al., 1997; Raven, 1997; Graham et al., 2000; Qiu, 2008). The presence of phragmoplast (p, enlarged cytoplasmic connection formed in the later stages of plant cell mitosis) in the zygnematalean taxa suggest that PD likely originate during the evolution of phragmoplast-containing charophyceans (Figure 1).

In their primary form, PD arise during cytokinesis, presumably via enclosure of endoplasmic reticulum by cell wall depositions (Hepler, 1981; Cook et al., 1997). Important features of plant PDs (such as neck constriction and central desmotubule like structure) appear in *Chara* species but since the colonization of land by plants (more than 400 million years ago) numerous modifications in PD ultrastructure and regulation are expected. A more complete understanding of the evolutionary steps involved in the origin of plant PDs, their function and regulation should be possible through the identification of plasmodesma-associated proteins and analysis of their evolutionary appearance in charophycean algae and land plants. Plasmodesma-associated proteins have been isolated in model plants, such as Arabidopsis and tobacco, using genetic and proteomic screens but the composition of the channel in model and non-model organisms is far from being resolved (Faulkner and Maule, 2011). Genome sequencing projects and prediction tools for protein structure and targeting has been proven useful to establish protein localization and function in different intracellular compartments (e.g., Pires and Dolan, 2010; Ma et al., 2011; Tardif et al., 2012). Known PD proteins display characteristic features of membrane-localized proteins (such as secretory signal peptides, glycosyl phosphatidylinositol anchors or transmembrane domains) but no specific sequence signature for PD-binding has been yet discovered.

Recently we have obtained information on the identity of Arabidopsis PD proteins, including several callose (beta 1,3 glucans) metabolic enzymes (Levy et al., 2007; Fernandez-Calvino et al., 2011; Vaten et al., 2011; Benitez-Alfonso et al., 2013). Callose deposition at PD neck region correlates with a reduction in symplastic transport during tissue maturation (Burch-Smith and Zambryski, 2012; Slewinski et al., 2012). Callose also acts as a reversible regulator of intercellular transport in response to developmental and environmental signals (Levy et al., 2007; Benitez-Alfonso et al., 2010; Maule et al., 2011, 2013; Rinne et al., 2011; Zavaliev et al., 2011). This implies that the activity of callose biosynthetic (callose synthases, CalS) and degrading enzymes (glycosyl hydrolase family 17, GHL17) must be rapidly and efficiently regulated at PD sites. Not surprisingly, PD-associated CalS and GHL17 proteins have been recently identified (Guseman et al., 2010; Vaten et al., 2011; Slewinski et al., 2012; Benitez-Alfonso et al., 2013; Zavaliev et al., 2013).

The role of plasmodesmata-localized GHL17 proteins in plant development and response to viral pathogens has been well established (Levy et al., 2007; Zavaliev et al., 2011; Burch-Smith and Zambryski, 2012). The identification of these enzymes in crop species could lead to the development of biotechnological approaches to improve plant growth and response to environmental and developmental signals. This task is hindered by the lack of tools to discriminate between plasma membrane (PM) and PD GHL17 proteins. Generation of fluorescent fusions and transgenics to determine intracellular localization will be required but, without any preliminary method to screen for candidates, this process could become very expensive and time consuming especially when dealing with large multigenic families such as GHL17.

Callose metabolic enzymes are conserved in fungi, oomycetes, algae and plants which indicate that this is a very ancient metabolic pathway (Bachman and McClay, 1996; Popper et al., 2011). What is not known is when this pathway was recruited to play an active role in PD regulation. The answer to this question might underlie in the evolutionary diversification of these enzymes to play PD-specific functions in land plants.

In this paper we present evidences supporting a potential correlation between the evolutionary origin of GHL17 proteins and their likelihood to target PD sites. Through phylogenetic analysis we identified a clade of membrane proteins that appear to have diverged early during land plants adaptation to terrestrial environments. The intracellular localization of predicted membrane GHL17 proteins isolated from Arabidopsis and Populus suggest that this “embryophytes only” subgroup is enriched in PD proteins (Pechanova et al., 2010; Fernandez-Calvino et al., 2011; Rinne et al., 2011; Benitez-Alfonso et al., 2013; Zavaliev et al., 2013). We used this information for the preliminary screen of 4 candidates identified through the proteomic screen of PD-enriched cell wall fractions. Two of the proteins belonged to clade alpha and were previously described to localize at PD. We tested the localization of two proteins that belonged to clade beta and found, through fluorescent imaging of m-Citrine protein fusions, that they accumulate preferentially in the apoplast. Our results suggest that at least a portion of GHL17 membrane proteins contained in clade alpha evolved in embryophytes differently from proteins contained in clade beta to specifically target PD and control callose on site.

## Materials and methods

### Retrieval of GHL17 sequences and analysis of protein domains

To isolate sequences containing the 1,3-beta glucosidase domain (GH17) from charophycean algae, *Physcomitrella patens* and selected embryophytes (*Arabidopsis thaliana, Populus trichocarpa* and *Oryza sativa*) BLAST (Altschul et al., 1990) searches were performed using as query five representative GHL17 sequences from *Arabidopsis thaliana* (At3g13560, At3g57260, At4g14080, At4g31140, At5g42100). For charophycean algae we searched the National Centre for Biotechnology Information (http://www.ncbi.nlm.nih.gov/) non-redundant (NR), high-throughput genome sequence (HTGS), whole genome shotgun (WGS), genome survey sequence (GSS) and expressed sequence tag (EST) databases. We obtained partial ESTs that were translated to amino acid sequences using Expasy translate tool. Presence of GH17 domain was confirmed in these sequences using the Conserved Domain (Marchler-Bauer et al., 2007) and SMART (http://smart.embl-heidelberg.de; Letunic et al., 2012) search engines. To isolate GH17 proteins from embryophytes sequenced genomes (*Physcomitrella patens, Populus trichocarpa* and *Oryza sativa*) a BLAST search against the Refseq protein database for each specific organism was performed using as query the same five Arabidopsis representative listed above and the GHL17 consensus domain sequence (cl18348). Similarly, to isolate beta-1,3-glucanases from fungi representatives (*Candida albicans, Aspergillus clavatus, Aspergillus fumigatus, Aspergillus niger, Candida glabrata, Debaryomyces hansenii, Ashbya gossypii, Fusarium graminearum, Kluyveromyces lactis, Saccharomyces cerevisiae, Scheffersomyces stipitis, Schizosaccharomyces pombe, Yarrowia lipolytica*) the consensus domain sequence (ci18819) was used to search the reference genome databases. Only protein sequences containing GH17 domain (confirmed in SMART) and predicted to be complete were considered. Aramemnon (http://aramemnon.uni-koeln.de/request.ep) was also used to search and/or confirm the identity of the proteins isolated in the Rice annotation project database or in Phytozome.

To eliminate redundancies, and/or to identify overlapping regions in isolated ESTs, sequences obtained for each organism were aligned using Muscle (Edgar, 2004). The resulting sequences are summarized in Table 1. These were screened for characteristic features of this family, the presence of a secretory signal peptide (SP), glycosyl phosphatidylinositol anchor (GPI) and carbohydrate-binding module (X8), using the prediction programs SMART, SignalP 4.1 Serve, Phobius, GPI-SOM, FragAnchor, PredGPI and BIG-PI respectively (Eisenhaber et al., 2003; Fankhauser and Maser, 2005; Poisson et al., 2007; Pierleoni et al., 2008; Petersen et al., 2011; Letunic et al., 2012). According to the results obtained full length sequences were classified in the following types: type 0 showed no obvious SP (non-secreted proteins); type 1 contains SP and might (or might not) contain one or more X8 domains (predicted secreted proteins); type 2 contains SP, one or more X8 domains and GPI anchor and type 3 contains SP and GPI anchor but not X8 domain. The presence of GPI anchor in type 2 and 3 proteins was used to predict their membrane localization. The classification of the sequences analyzed is provided in Table 2.

**Table 1:** **List of sequences used for constructing the phylogenetic trees**.

**Organism**	**Identifier in this paper**	**Sequence identifier**
*Klebsormidium flaccidum*	KfGHL17_1	HO446722 +
		HO446665^*^
*Klebsormidium flaccidum*	KfGHL17_2	HO451810.1
*Penium margaritaceum*	PmGHL17_1	JO220251.1
*Chaetosphaeridium globosum*	CgGHL17_1	HO400516.1
*Nitella mirabilis*	NtGHL17_1	JV792233.1
*Nitella mirabilis*	NtGHL17_2	JV742253.1
*Nitella mirabilis*	NtGHL17_3	JV760383.1
*Physcomitrella patens*	PpGHL17_1	XP_001761806.1
*Physcomitrella patens*	PpGHL17_2	XP_001772420.1
*Physcomitrella patens*	PpGHL17_3	XP_001780679.1
*Physcomitrella patens*	PpGHL17_4	XP_001762206.1
*Physcomitrella patens*	PpGHL17_5	XP_001780506.1
*Physcomitrella patens*	PpGHL17_6	XP_001779924.1
*Physcomitrella patens*	PpGHL17_7	XP_001767901.1
*Physcomitrella patens*	PpGHL17_8	XP_001771454.1
*Physcomitrella patens*	PpGHL17_9	XP_001782572.1
*Physcomitrella patens*	PpGHL17_10	XP_001773368.1
*Physcomitrella patens*	PpGHL17_11	XP_001782548.1
*Physcomitrella patens*	PpGHL17_12	XP_001772976.1
*Physcomitrella patens*	PpGHL17_13	XP_001757439.1
*Physcomitrella patens*	PpGHL17_14	XP_001754617.1
*Physcomitrella patens*	PpGHL17_15	XP_001775842.1
*Physcomitrella patens*	PpGHL17_16	XP_001762304.1
*Physcomitrella patens*	PpGHL17_17	XP_001757144
*Physcomitrella patens*	PpGHL17_18	XP_001777261.1
*Candida albicans*	CaGHL17_1	P43070.1
*Aspergillus clavatus*	AcGHL17_1	XP_001269132.1
*Aspergillus fumigatus*	AfgHL17_1	XP_752511.1
*Aspergillus niger*	AnGHL17_1	XP_001392475.1
*Candida glabrata*	CglGHL17_1	XP_446374.1
*Debaryomyces hansenii*	DhGHL17_1	XP_462355.1
*Ashbya gossypii*	AgGHL17_1	NP_986324.2
*Fusarium graminearum*	FgGHL17_1	XP_383705.1
*Kluyveromyces lactis*	KlGHL17_1	XP_455217.1
*Saccharomyces cerevisiae*	ScGHL17_1	NP_011798.1
*Scheffersomyces stipitis*	SsGHL17_1	XP_001387556.1
*Schizosaccharomyces pombe*	SpoGHL17_1	NP_594455.1
*Yarrowia lipolytica*	YlGHL17_1	XP_500465.1
*Oryza sativa*	OsGHL17_1	NP_001052739.1
*Oryza sativa*	OsGHL17_2	NP_001044874.1
*Oryza sativa*	OsGHL17_3	NP_001047027.1
*Oryza sativa*	OsGHL17_4	NP_001046220.1
*Oryza sativa*	OsGHL17_5	NP_001058028.1
*Oryza sativa*	OsGHL17_6	NP_001044198.1
*Oryza sativa*	OsGHL17_7	NP_001051111.1
*Oryza sativa*	OsGHL17_8	NP_001049413.1
*Oryza sativa*	OsGHL17_9	NP_001060087.2
*Oryza sativa*	OsGHL17_10	NP_001059752.1
*Oryza sativa*	OsGHL17_11	NP_001068140.2
*Oryza sativa*	OsGHL17_12	NP_001057968.1
*Oryza sativa*	OsGHL17_13	BAD31779.1
*Oryza sativa*	OsGHL17_14	NP_001173461.1
*Oryza sativa*	OsGHL17_15	NP_001050810.1
*Oryza sativa*	OsGHL17_16	NP_001056153.1
*Oryza sativa*	OsGHL17_17	NP_001062739.1
*Oryza sativa*	OsGHL17_18	BAD01673.1
*Oryza sativa*	OsGHL17_19	NP_001061277.1
*Oryza sativa*	OsGHL17_20	NP_001045844.1
*Oryza sativa*	OsGHL17_21	AA037977
*Oryza sativa*	OsGHL17_22	AAP44659
*Oryza sativa*	OsGHL17_23	ABF94756.1
*Oryza sativa*	OsGHL17_24	ABF95444.1
*Arabidopsis thaliana*	At2g05790	NP_178637.2
*Arabidopsis thaliana*	At4g26830	NP_194413.2
*Arabidopsis thaliana*	At5g55180	NP_001154780.1
*Arabidopsis thaliana*	At4g18340	NP_193568.2
*Arabidopsis thaliana*	At1g30080	NP_174300.2
*Arabidopsis thaliana*	At2g26600	NP_850082.1
*Arabidopsis thaliana*	At3g15800	NP_188201.1
*Arabidopsis thaliana*	At2g27500	NP_001031432
*Arabidopsis thaliana*	At5g42100	NP_974868.1
*Arabidopsis thaliana*	At1g32860	NP_174563.2
*Arabidopsis thaliana*	At5g24318	NP_001119271.1
*Arabidopsis thaliana*	At3g46570	NP_190241.1
*Arabidopsis thaliana*	At2g39640	NP_181494.1
*Arabidopsis thaliana*	At3g55430	NP_191103.1
*Arabidopsis thaliana*	At5g42720	NP_199086.2
*Arabidopsis thaliana*	At4g34480	NP_195174.6
*Arabidopsis thaliana*	At2g16230	NP_179219.4
*Arabidopsis thaliana*	At3g13560	NP_974303.1
*Arabidopsis thaliana*	At1g11820	NP_001184967.1
*Arabidopsis thaliana*	At1g66250	NP_176799.2
*Arabidopsis thaliana*	At2g01630	NP_001077866.1
*Arabidopsis thaliana*	At4g29360	NP_567828.3
*Arabidopsis thaliana*	At5g56590	NP_200470.1
*Arabidopsis thaliana*	At3g55780	NP_191137.1
*Arabidopsis thaliana*	At3g61810	NP_191740.1
*Arabidopsis thaliana*	At3g07320	NP_683538.1
*Arabidopsis thaliana*	At3g23770	NP_189019.1
*Arabidopsis thaliana*	At4g14080	NP_193144.1
*Arabidopsis thaliana*	At5g58480	NP_200656.2
*Arabidopsis thaliana*	At4g17180	NP_193451.2
*Arabidopsis thaliana*	At5g64790	NP_201284.1
*Arabidopsis thaliana*	At3g04010	NP_187051.3
*Arabidopsis thaliana*	At5g18220	NP_197323.1
*Arabidopsis thaliana*	At1g64760	NP_001031232.1
*Arabidopsis thaliana*	At2g19440	NP_179534.1
*Arabidopsis thaliana*	At3g24330	NP_189076.1
*Arabidopsis thaliana*	At5g20870	NP_197587.1
*Arabidopsis thaliana*	At5g58090	NP_200617.2
*Arabidopsis thaliana*	At4g31140	NP_194843.1
*Arabidopsis thaliana*	At1g77790	NP_177902.1
*Arabidopsis thaliana*	At1g77780	NP_177901.1
*Arabidopsis thaliana*	At5g20390	NP_197539.1
*Arabidopsis thaliana*	At5g20560	NP_197556.1
*Arabidopsis thaliana*	At1g33220	NP_174592.1
*Arabidopsis thaliana*	At5g20340	NP_197534.1
*Arabidopsis thaliana*	At5g20330	NP_197533.1
*Arabidopsis thaliana*	At4g16260	NP_193361.4
*Arabidopsis thaliana*	At3g57270	NP_191286.1
*Arabidopsis thaliana*	At3g57240	NP_191283.2
*Arabidopsis thaliana*	At3g57260	NP_191285.1
*Populus trichocarpa*	PtGHL17_1	XP_002297638.2
*Populus trichocarpa*	PtGHL17_2	XP_002304004.2
*Populus trichocarpa*	PtGHL17_3	XP_002314794.2
*Populus trichocarpa*	PtGHL17_4	XP_002305879.1
*Populus trichocarpa*	PtGHL17_5	XP_006389594.1
*Populus trichocarpa*	PtGHL17_6	XP_006371969.1
*Populus trichocarpa*	PtGHL17_7	XP_002316783.2
*Populus trichocarpa*	PtGHL17_8	XP_002333242.1
*Populus trichocarpa*	PtGHL17_9	XP_002302861.2
*Populus trichocarpa*	PtGHL17_10	XP_002318439.2
*Populus trichocarpa*	PtGHL17_11	XP_006384505.1
*Populus trichocarpa*	PtGHL17_12	XP_006379239.1
*Populus trichocarpa*	PtGHL17_13	XP_002312097.1
*Populus trichocarpa*	PtGHL17_14	XP_002312098.1
*Populus trichocarpa*	PtGHL17_15	XP_002303070.2
*Populus trichocarpa*	PtGHL17_16	XP_002298356.1
*Populus trichocarpa*	PtGHL17_17	XP_002332000.1
*Populus trichocarpa*	PtGHL17_18	XP_002317055.2
*Populus trichocarpa*	PtGHL17_19	XP_002306003.2
*Populus trichocarpa*	PtGHL17_20	XP_006385314.1
*Populus trichocarpa*	PtGHL17_21	XP_002300505.2
*Populus trichocarpa*	PtGHL17_22	XP_002300634.2
*Populus trichocarpa*	PtGHL17_23	XP_002299750.2
*Populus trichocarpa*	PtGHL17_24	XP_002312820.1
*Populus trichocarpa*	PtGHL17_25	XP_002325214.2
*Populus trichocarpa*	PtGHL17_26	XP_002328249.1
*Populus trichocarpa*	PtGHL17_27	XP_002321273.1
*Populus trichocarpa*	PtGHL17_28	XP_006386924
*Populus trichocarpa*	PtGHL17_29	XP_002329975.1
*Populus trichocarpa*	PtGHL17_30	XP_002321266.1
*Populus trichocarpa*	PtGHL17_31	XP_002329954.1
*Populus trichocarpa*	PtGHL17_32	XP_002315222.2
*Populus trichocarpa*	PtGHL17_33	XP_002332466.1
*Populus trichocarpa*	PtGHL17_34	XP_002329964.1
*Populus trichocarpa*	PtGHL17_35	XP_002332467.1
*Populus trichocarpa*	PtGHL17_36	XP_002324127.1
*Populus trichocarpa*	PtGHL17_37	XP_002329956.1
*Populus trichocarpa*	PtGHL17_38	XP_002302261.1
*Populus trichocarpa*	PtGHL17_39	XP_002313970.1
*Populus trichocarpa*	PtGHL17_40	XP_002319699.1
*Populus trichocarpa*	PtGHL17_41	XP_006372260.1
*Populus trichocarpa*	PtGHL17_42	XP_002330836.1
*Populus trichocarpa*	PtGHL17_43	XP_002308921.2
*Populus trichocarpa*	PtGHL17_44	XP_002306606.2
*Populus trichocarpa*	PtGHL17_45	XP_002299791.2
*Populus trichocarpa*	PtGHL17_46	XP_002309443.2
*Populus trichocarpa*	PtGHL17_47	XP_002310612.1
*Populus trichocarpa*	PtGHL17_48	XP_002323325.2
*Populus trichocarpa*	PtGHL17_49	XP_002314934.2
*Populus trichocarpa*	PtGHL17_50	XP_002315775.2
*Populus trichocarpa*	PtGHL17_51	XP_002308018.2
*Populus trichocarpa*	PtGHL17_52	XP_002314086.1
*Populus trichocarpa*	PtGHL17_53	XP_002324967
*Populus trichocarpa*	PtGHL17_54	XP_002305174.1

*The table includes the source organism, abbreviation used in this study and sequence identifier en NCBI*.

* *This ORF was obtained by translating the sequence resulting from overlapping these two ESTs*.

**Table 2:** **Classification of embryophyte sequences based on protein structure and phylogenetic distribution**.

**Sequence identifier**	**Type**	**Branch**
PpGHL17_1	1	α
PpGHL17_2	1	α
PpGHL17_3	1	α
PpGHL17_4	0	α
PpGHL17_5	1	α
PpGHL17_6	1	α
PpGHL17_7	2	α
PpGHL17_8	0	α
PpGHL17_9	0	α
PpGHL17_10	2	β
PpGHL17_11	1	α
PpGHL17_12	2	β
PpGHL17_13	2	β
PpGHL17_14	1	β
PpGHL17_15	1	β
PpGHL17_16	0	β
PpGHL17_17	0	α
PpGHL17_18	0	α
OsGHL17_1	3	α
OsGHL17_2	3	α
OsGHL17_3	3	α
OsGHL17_4	3	α
OsGHL17_5	3	α
OsGHL17_6	1	α
OsGHL17_7	3	α
OsGHL17_8	2	α
OsGHL17_9	2	α
OsGHL17_10	2	α
OsGHL17_11	2	β
OsGHL17_12	2	β
OsGHL17_13	2	β
OsGHL17_14	2	β
OsGHL17_15	2	β
OsGHL17_16	2	β
OsGHL17_17	2	β
OsGHL17_18	2	β
OsGHL17_19	2	β
OsGHL17_20	2	β
OsGHL17_21	2	β
OsGHL17_22	1	α
OsGHL17_23	3	α
OsGHL17_24	2	β
At2g05790	1	α
At4g26830	1	α
At5g55180	1	α
At4g18340	1	α
At1g30080	1	α
At2g26600	3	α
At3g15800	3	α
At2g27500	1	α
At5g42100	3	α
At1g32860	3	α
At5g24318	1	α
At3g46570	1	α
At2g39640	1	α
At3g55430	1	α
At5g42720	3	α
At4g34480	1	α
At2g16230	1	α
At3g13560	2	α
At1g11820	1	α
At1g66250	2	α
At2g01630	2	α
At4g29360	2	α
At5g56590	2	α
At3g55780	1	α
At3g61810	1	α
At3g07320	1	α
At3g23770	1	α
At4g14080	1	α
At5g58480	2	β
At4g17180	1	β
At5g64790	2	β
At3g04010	2	β
At5g18220	2	β
At1g64760	2	β
At2g19440	2	β
At3g24330	2	β
At5g20870	2	β
At5g58090	2	β
At4g31140	2	β
At1g77790	1	γ
At1g77780	3	γ
At5g20390	1	γ
At5g20560	1	γ
At1g33220	1	γ
At5g20340	1	γ
At5g20330	1	γ
At4g16260	1	γ
At3g57270	1	γ
At3g57240	1	γ
At3g57260	1	γ
PtGHL17_1	2	α
PtGHL17_2	1	α
PtGHL17_3	2	α
PtGHL17_4	0	α
PtGHL17_5	2	α
PtGHL17_6	2	α
PtGHL17_7	1	α
PtGHL17_8	1	α
PtGHL17_9	1	α
PtGHL17_10	1	α
PtGHL17_11	1	α
PtGHL17_12	1	α
PtGHL17_13	1	α
PtGHL17_14	1	α
PtGHL17_15	1	α
PtGHL17_16	1	α
PtGHL17_17	1	α
PtGHL17_18	3	α
PtGHL17_19	1	α
PtGHL17_20	3	α
PtGHL17_21	3	α
PtGHL17_22	1	α
PtGHL17_23	3	α
PtGHL17_24	1	α
PtGHL17_25	3	α
PtGHL17_26	3	α
PtGHL17_27	1	α
PtGHL17_28	1	α
PtGHL17_29	3	α
PtGHL17_30	1	α
PtGHL17_31	1	α
PtGHL17_32	2	α
PtGHL17_33	1	α
PtGHL17_34	2	α
PtGHL17_35	1	β
PtGHL17_36	2	β
PtGHL17_37	1	α
PtGHL17_38	0	γ
PtGHL17_39	2	β
PtGHL17_40	2	β
PtGHL17_41	2	β
PtGHL17_42	1	β
PtGHL17_43	1	γ
PtGHL17_44	0	γ
PtGHL17_45	1	γ
PtGHL17_46	2	β
PtGHL17_47	2	β
PtGHL17_48	1	γ
PtGHL17_49	1	γ
PtGHL17_50	0	γ
PtGHL17_51	1	γ
PtGHL17_52	1	γ
PtGHL17_53	2	β
PtGHL17_54	3	α

The table classifies the sequences used in this paper according to the presence of signal peptide, X8 domain and/or GPI anchor as described in Materials and Methods. It also mentions the branch in the tree where this sequence appears. Consult Table 1 to access the sequence corresponding to each identifier in NCBI.

### Alignments, sequence conservation, and phylogenetic analysis

All sequences isolated from representatives of charophycean algae and fungi, *P. patens, Oryza sativa* and *Arabidopsis thaliana* (Table 1) were aligned using Muscle (Edgar, 2004). Sequences from algae were incomplete which generate large gaps. These gaps were mostly avoided when only the domain was used. Therefore we constructed trees with both, full sequences and domain only. These alignments are provided in Supplementary data 1. To calculate the best fitting model of amino acid evolution MEGA5 was used (Tamura et al., 2013). This suggests WAG+G+F as the best model under the Akaike Information Criterion. Dendograms were obtained using three different methods of tree reconstruction [maximum likelihood (ML), neighbor-joining (NJ) and Bayesian inference (Bayesian)]. A majority-rule consensus tree was built by Bayesian inference using Mr. Bayes (Huelsenbeck and Ronquist, 2001). Convergence was reached after 960000 generations (3720000 when using domain only) and posterior probabilities were calculated for each clade. Using the same model a ML analysis was performed with MEGA5 (Tamura et al., 2013) and bootstrap values were determined from a population of 100 replicates. A NJ tree was also generated using Phylip (Felsenstein, 1997) as well as bootstrap values, which were determined from a population of 100 replicates. The tree was visualized using Figtree (http://tree.bio.ed.ac.uk/software/figtree/). A similar protocol was followed for phylogenetic comparison of *Arabidopsis thaliana* and *Populus trichocarpa* sequences (alignments provided in Supplementary data 2). In this case convergence was reached after 45000 generations.

A graphical representation of the GH17 domain alignment was performed using weblogo3 (Crooks et al., 2004). In the logo the overall height of the stack indicates the sequence conservation at that position.

### Generation of transgenic plant material

Construction of p*35S*-mCitrine-PdBG1 (At3g13560) was described elsewhere (Benitez-Alfonso et al., 2013). N-terminal and GPI-anchor domains were predicted for At4g31140 and At5g58090 using SignalP 4.1 Serve and GPI-SOM (Fankhauser and Maser, 2005; Petersen et al., 2011). mCitrine protein fusions were obtained by overlapping PCR (Tian et al., 2004) and expressed in the binary vector pB7WG2.0 using Gateway technology. The mCitrine was fused in frame between amino acids 454–455 in the case of At4g31140 and between amino acids 445–446 in the case of At5g58090.

Transient expression was verified by agroinfiltration in *Nicotiana benthamiana* leaves. Stable transgenic lines were generated using the floral dip method, followed by selection with BASTA. T2 seeds were sterilized and germinated in long day conditions on plates containing MS medium supplemented with BASTA (25 μg/ml).

### Callose staining

Callose deposition at PD was detected in plant samples vacuum infiltrated with 0,1% (w/v) aniline blue in 0,1M sodium phosphate (pH 9.0) and incubated in the dark for 1–2 h before imaging.

### Microscopy

Confocal analysis was performed on a Zeiss LSM700 Inverted microscope using a 488 nm excitation laser for mCitrine, the 405 nm laser for aniline blue fluorochrome and 585 nm laser to detect chloroplast autofluorescence. Emission was collected using the filters: BP 505–530 for mCitrine, the DAPI filter for aniline blue (463 nm) and LP 615 filter for chloroplasts (581 nm). The images corresponded to stacks of z- optical sections. Sequential scanning was used to image tissues expressing mCitrine and stained with aniline blue.

## Results

### Identification of GHL17 sequences in charophytes and embryophytes suggest gene family expansion

The presence of intercellular connections (phragmoplast and/or less evolved PD) has been described in some species belonging to the *Charophytes* (Figure 1) but so far, in this lineage, regulation of PD by callose metabolism has only been demonstrated in embryophytes (Scherp et al., 2001; Schuette et al., 2009). The presence of β-1,3 glucans in the cell wall of unicellular organisms indicate an ancient origin for this metabolic pathway but how and when it evolved to control PD transport is unknown (Sorensen et al., 2011). In an attempt to answer this question, we isolated sequences encoding GH17 domains from charophytes, bryophytes, and vascular plants. Based on the availability of sequence information, we selected representative species from the charophycean orders: Klebsormidiales (*Klebsormidium flaccidum*), Zignematales (*Penium margaritaceum*), Coleochatales (*Chaetosphaeridium globosum*) and Charales (*Nitella mirabilis*). 14 partial transcripts were isolated but only 7 (2 from *Klebsormidium*, 1 from *Penium*, 1 from *C. globosum* and 3 from *Nitella*) contained key aminoacids forming the active site of GHL17 (Table 1).

Full-length GHL17 sequences were isolated from moss (*Physcomitrella patents*) and from monocots (*Oryza sativa*) and dicots (*Arabidopsis thaliana* and *Populus trichocarpa*) model plants using genome information and protein annotation databases. In total we were able to identify 18 sequences in *Physcomitrella*, 24 sequences in *Oryza sativa*, 50 sequences in *Arabidopsis thaliana* and 54 in *Populus trichocarpa* (Table 1). The increasing number of sequences isolated in land plants with respect to those isolated in algae and moss suggests that an expansion in this gene family have occurred during or immediately after land colonization.

We used prediction tools to determine the structure and localization of the proteins encoded by the sequences identified. This was not possible for algae representatives because only partial transcripts were isolated. For moss, rice, Arabidopsis and Populus sequences, secretory signal peptides (SP) and the presence of C-terminal GPI anchoring domains were predicted using several bioinformatics websites (see Material and Methods). GHL17 sequences were also classified according to the presence of one or more carbohydrate binding domains (named X8 or CBM43). We classified sequences in 4 types according to the presence of one or more of these features (see Material and Methods and Table 2). Type 2 and 3 displayed a SP and GPI-anchor signature that predicts their localization at the PM or at membranous subdomains (such as PD). From the 18 sequences isolated in *Physcomitrella* only 4 were classified as type 2. Arabidopsis genome contained 21 membrane predicted sequences (42% of the total), which were experimentally verified in a proteomic analysis (Borner et al., 2003). The number of membrane predicted GHL17 was very similar in rice and *Populus trichocarpa* (22 in rice, 21 in poplar). When comparing moss and vascular plants a major increase in the number of predicted membrane-targeted proteins is detected consistent with the hypothesis that GHL17 evolved and expanded to support or adopt specialized functions at membraneous domains in terrestrial environments.

### Key amino acid residues in the GH17 domain are conserved throughout evolution

Research on GHL17 protein structure revealed two strictly conserved glutamate residues that act as the proton donor and the nucleophile in all reactions catalyzed by glycosyl hydrolases (Jenkins et al., 1995; Wojtkowiak et al., 2013). A number of aromatic and hydrophilic residues located near the catalytic cleft, presumably involved in substrate specificity and enzyme activity, are also conserved among all plant GHL17 proteins (Wojtkowiak et al., 2013).

**Figure 2:**
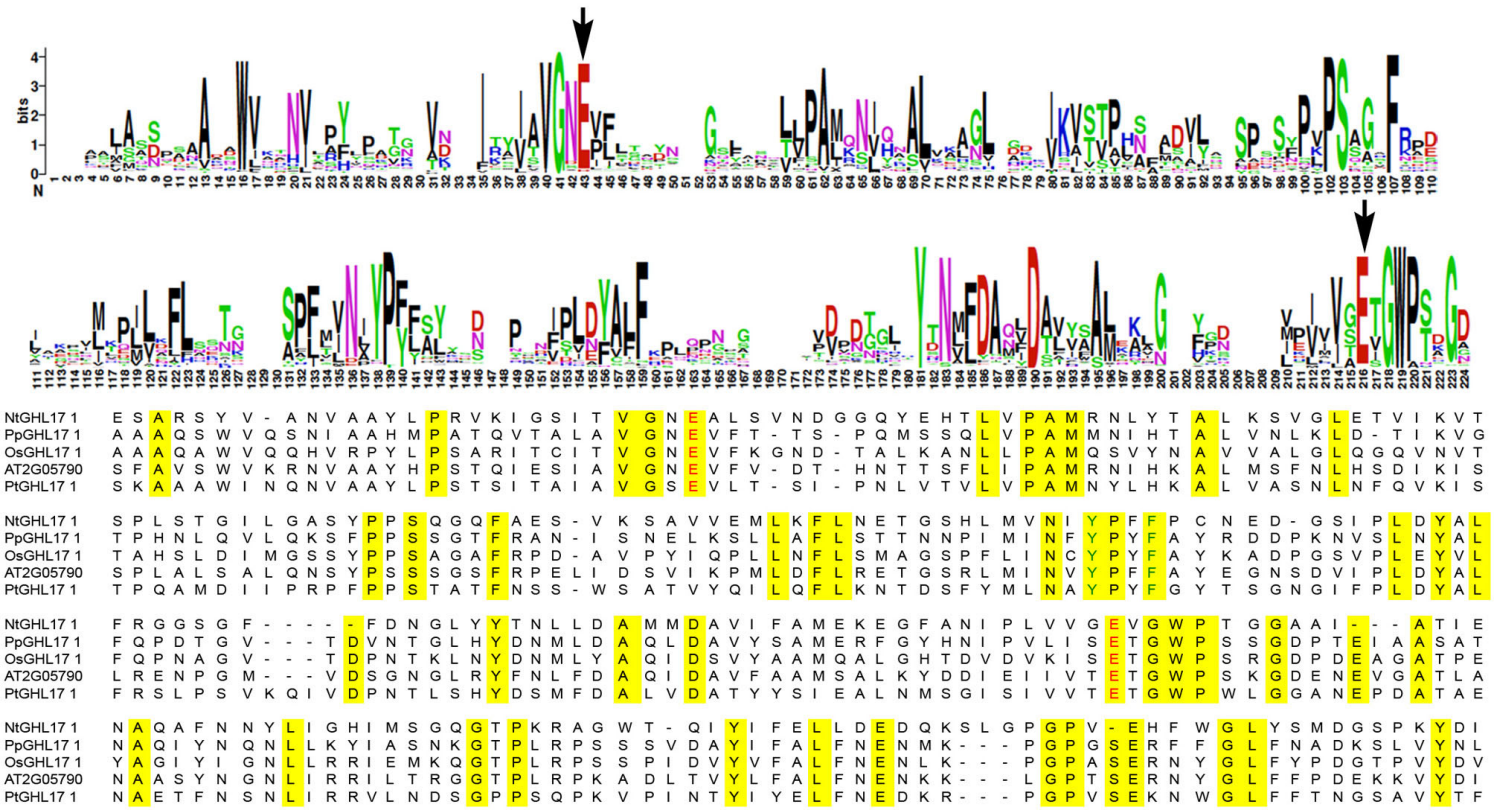
**Sequence conservation in the domain region of GHL17 proteins**. The top panels show the consensus region for GH17 using weblogo. This was obtained by aligning all the sequences isolated from green algae and embryophytes (consult Table 1 to obtain the NCBI identifier for these sequences). The bottom panel shows an alignment of representative domain sequences from *Nitella mirabilis* (NtGHL17_1), from moss (PpGHL17_1) and from the vascular plants *Arabidopsis thaliana* (At2G05790), *Oryza sativa* (OsGHL17_1) and *Populus trichocarpa* (PtGHL17_1). Conserved aminoacids are highlighted in yellow in the alignment. The position of the glutamate residues (E) actively involved in the catalytic reaction is indicated with arrows in the weblogo and in red in the alignment. Notice conserved domains around the catalytic sites. Tyr (Y) and Phe (F) residues conserved in plants and presumably important in substrate binding are indicated in green in the bottom panel.

To study the molecular evolution of the GH17 domain in green algae, moss and plants, we translated and aligned the domain region of the retrieved sequences using MEGA5 (Supplementary data 1). We also included sequences isolated from fungi representatives to analyze domain conservation in a different lineage. The results revealed that the glutamate catalytic residues (E) are highly conserved among all charophycean representatives, fungi and embryophytes (highlighted in red in the alignment shown in Supplementary data 1 and in Figure 2). Similarly, the residues surrounding the catalytic site are mostly conserved in all selected representatives (Supplementary data 1, Figure 2). Moreover a region contained the aromatic residues Tyr200 and Phe203 (location refer to At2g05790 sequence), which is involved in substrate interaction (Wojtkowiak et al., 2013), is also conserved in all streptophytes (Figure 2).

The high degree of similarity between the catalytic sites of GHL17 proteins in green algae, fungi and land plants supports the ancestral origins of this metabolic pathway.

### Phylogeny revealed a group of GHL17 proteins that appeared in embryophytes only

The phylogenetic distribution of Arabidopsis GHL17 sequences has been studied before (Doxey et al., 2007). Based on tree topology, these proteins were grouped into three distinct clades: α, β, and γ. Predicted membrane GHL17 were evenly distributed in clade α and β. We investigated the evolutionary origin of these clades by comparing the phylogenetic distribution of GHL17 sequences isolated from charophycean green algae, fungi *Physcomitrella patens, Oryza sativa* and *Arabidopsis thaliana*. Although plants and fungi evolved in a different lineage, they share a common eukaryotic origin, which is reflected in the conservation of key aminoacids in the GH17 domain (Supplementary data 1).

**Figure 3:**
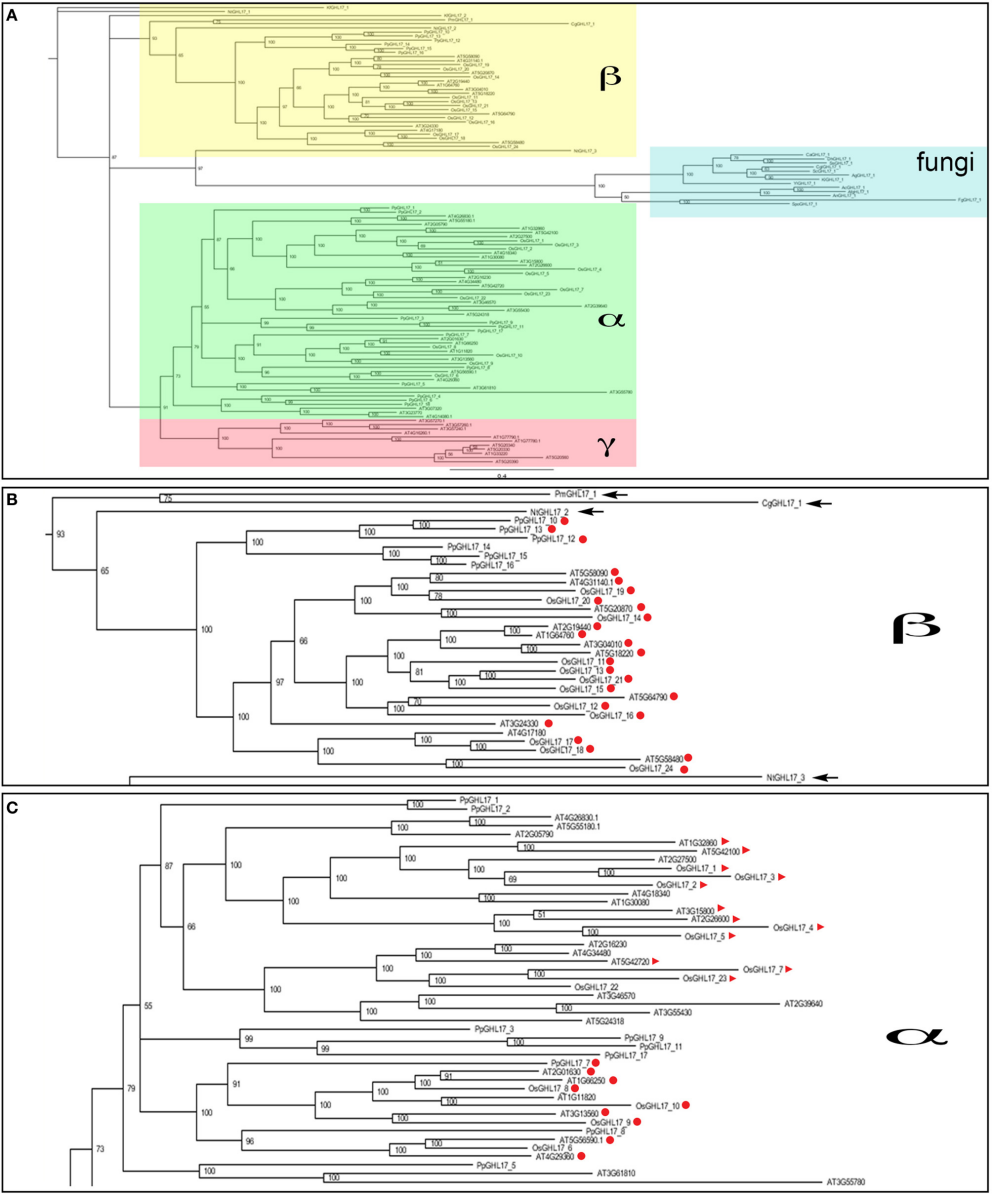
**Bayesian phylogenetic consensus tree of GHL17 sequences isolated from fungi, green algae and embryophytes representatives (A)**. All sequences are cited in Table 1 and alignment provided in Supplementary data 1. Bayesian posterior probabilities are indicated in the branches. Clades α (in green), β (in yellow), and γ (in red), as defined for Arabidopsis in Doxey et al. (2007), are indicated. Fungi sequences form a separate group consistent with a different evolutionary lineage. **(B)** shows a close-up of clade β and **(C)** shows a portion of the α clade. Algae sequences are arrowed in **(B)** and membrane predicted proteins, type 2 and 3, are marked in red circles and red triangles respectively.

Unrooted phylogenetic trees were generated using three search algorithms: Bayesian inference (Bayesian), Maximum Likelihood (ML) and Neighbor Joining (NJ) (Figure 3A and supplementary data 3). The tree topology was generally well supported by all 3 methods, with the exception of several higher order branches in ML and NJ bootstrap values. The three phylogenetic clades (α, β, and γ) described by Doxey et al. (2007) are color coded in Figure 3A. Fungi selected sequences branch off at the same point as some algae representatives and near the point of connection of plant sequences forming the clade beta. This suggests a more ancestral origin for this clade (Figure 3B). Clade alpha and gamma contained embryophytes only and, for the purpose of this paper, they could be considered as a single clade (Figure 3C).

Only partial transcripts were isolated for algae representatives hence gaps were introduced in the alignments that could affect the accuracy and reliability of the trees. To confirm the tree topology, we manually eliminate these gaps to generate trees containing the sequence region encoding the domain only (marked in yellow in Supplementary data 1). As shown in supplementary data 3, the distribution of sequences in the different clades and the relationship between the different branches was conserved in these “domain only” trees.

As in Arabidopsis, even distribution of predicted membrane sequences between the alpha and the beta clade was observed in rice (Figures 3B,C). Interestingly, type 3 proteins were almost exclusively found in the alpha clade. In summary our phylogenetic analysis suggest that GHL17 membrane proteins contained in clade alpha appeared in early embryophytes presumably to adopt new functions at the cell periphery.

### PD localized GHL17 proteins are contained in the α clade

Since cell wall composition and PD complexity evolved during land plant colonization, it seems logical to assume that callose, and specialized callose metabolic enzymes, were adopted at some stage during this evolutionary process to regulate PD aperture. The presence of charophytic sequences and the proximity to a fungi branch suggests a more ancestral origin for membrane proteins included in the beta clade (Figure 3B). We hypothesize that PD-targeted GHL17 proteins evolved with the appearance of early embryophytes, hence likely be contained within the alpha clade (Figure 3C).

The Bayesian tree shows (with high support values) 10 predicted membrane proteins (type 2 and 3) from Arabidopsis contained in the alpha clade whereas 10 type 2 sequences appeared in a compact clade within the beta subgroup surrounded by sequences isolated from green algae (Figures 3B,C). Data from several publications reported the intracellular localization of several GHL17 proteins in Arabidopsis. The root developmental regulators At3g13560, At2g01630, and At1g66250 (Benitez-Alfonso et al., 2013) and the virus-induced protein At5g42100 (Levy et al., 2007) were PD-localized whereas At3g57260 was preferentially expressed in the apoplast (Zavaliev et al., 2013). Confirming our hypothesis, all PD localized proteins were grouped in the alpha clade (Figure 3C).

**Figure 4:**
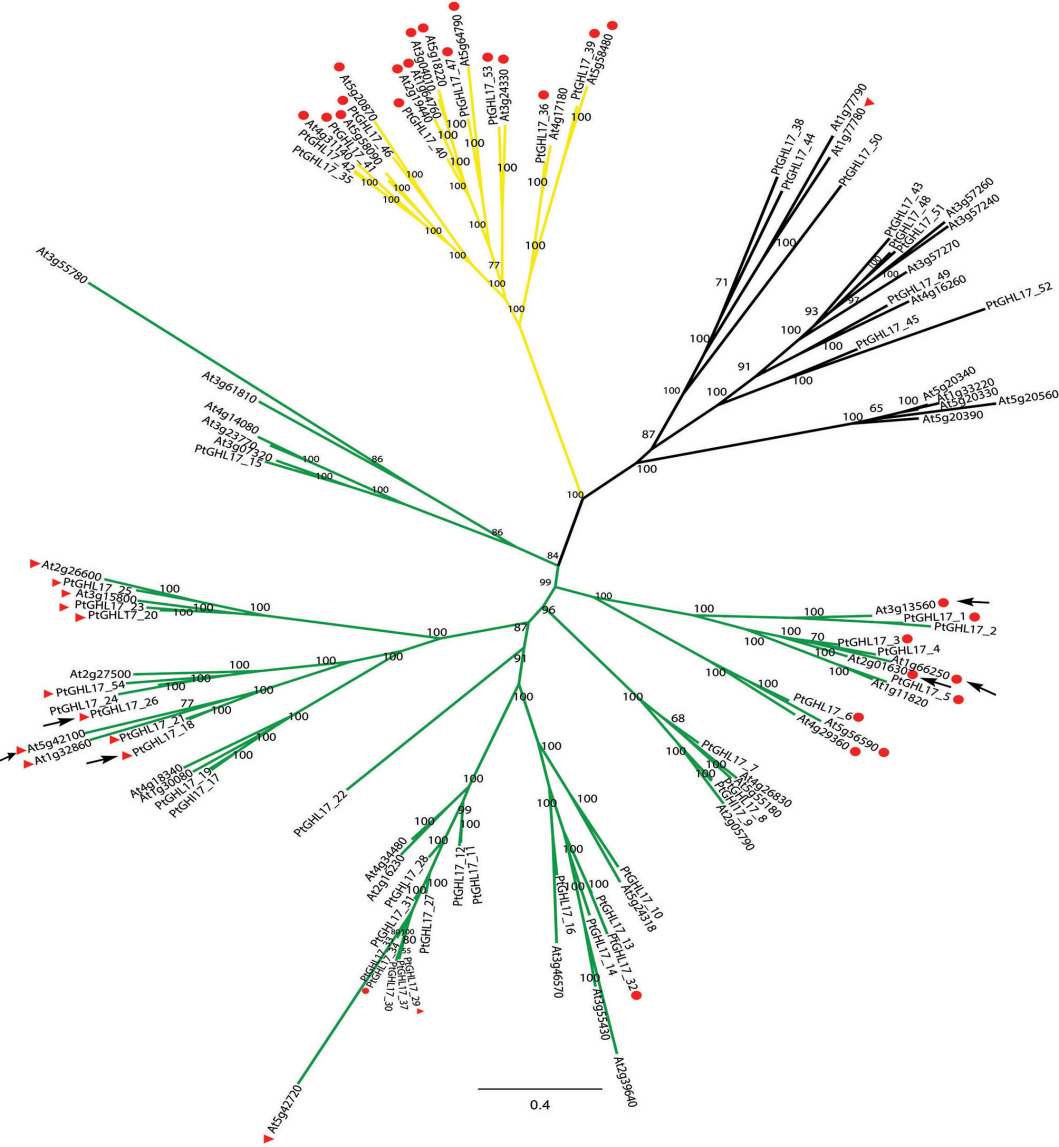
**Majority consensus tree generated by Bayesian inference of phylogeny of GHL17 proteins isolated from *A. thaliana* (At) and *P. trichocarpa* (Pt) (sequences cited in Table 1)**. Bayesian posterior probabilities are indicated in the tree branches. In accordance with the phylogenetic tree presented in Figure 3, branches forming clades α (green), β (yellow) and γ (black) have been indicated. Type 2 and 3 proteins (GPI-anchored proteins) are indicated with red circles and red triangles respectively. The position of PtGHL17_18 and PtGHL17_26 (reported to localize at PD by Rinne et al., 2011), as well as the position of PD-localized Arabidopsis proteins has been indicated with arrows.

The localization of few GHL17 proteins from Populus has been recently reported (Pechanova et al., 2010; Rinne et al., 2011). To test the relationship between the appearance of the alpha clade and protein localization, we constructed a Bayesian tree with GHL17 sequences isolated from Arabidopsis and from *Populus trichocarpa*. BLAST searches against the Populus genome identified a total of 54 non-redundant sequences containing the GH17 domain (Table 1). Classification of these sequences according to bioinformatic predictions identified 21 putative membrane proteins (Table 2). A multiple sequence alignment was conducted and unrooted phylogenetic trees were generated using the Bayesian, ML and NJ algorithms (Figure 4 and Supplementary data 2 and 4). According to tree topology, Populus GHL17 proteins also appeared grouped in 3 clades α, β, and γ, each well supported by high probability values in each tree (Figure 4 and Supplementary data 4). As before, type 3 proteins were contained within the α clade whereas type 2 proteins were distributed between the α and β clades.

Orthologs of PtGHL17_18 and PtGHL17_26 were both found to target PD whereas PtGHL17_48 and PtGHL17_49 orthologs were mainly localized at the PM and lipid bodies (Rinne et al., 2011). As expected, PtGHL17_18 and PtGHL17_26 are membrane predicted proteins contained in the alpha clade (Figure 4). The results confirmed a potential link between the phylogenetic distribution of GHL17 proteins and their intracellular localization.

### Using phylogenetic distribution to discriminate between candidates for PD localization

**Figure 5:**
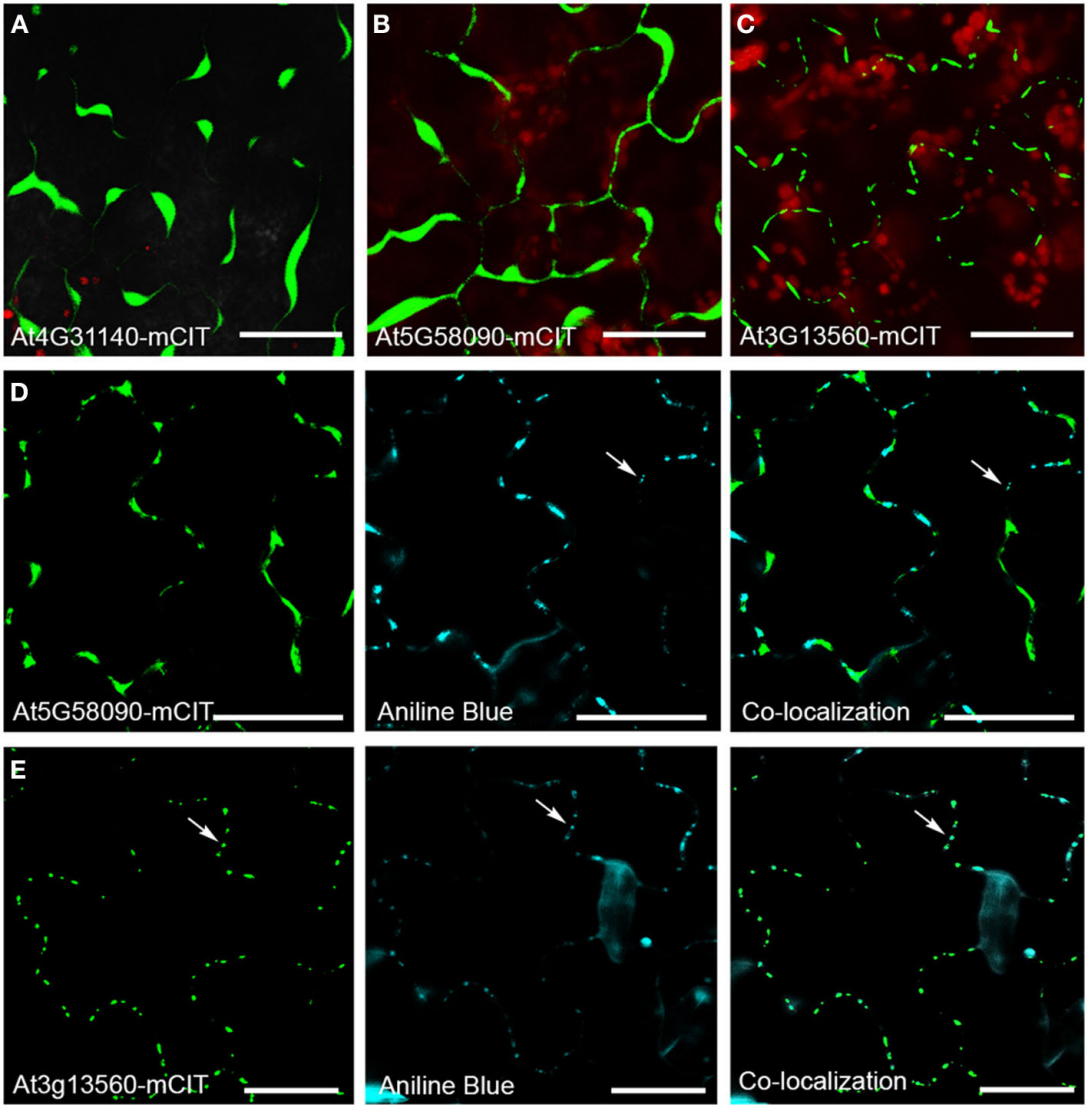
**Intracellular localization of GHL17 protein m-Citrine fusions. (A,B,C)** Show At4g31140-mCit, At5g58090-mCitm, and At3g13560-mCit transient expression in tobacco leaves. Chloroplast auto-fluorescence appears in red. **(D,E)** Show At5g58090-mCit and At3g13560-mCit fluorescence (green) in Arabidopsis leaves expressing the fusion proteins under the 35S promoter. Aniline blue staining of callose deposits (blue) and the green and blue channels superimposed are also shown. Notice that At3g13560 expression, but not At5g58090, co-localizes with callose deposits at PD (white arrows). Scale bars = 20 μm.

To identify novel PD components the proteomic composition of PD-enriched cell walls has been analyzed (Bayer et al., 2006; Fernandez-Calvino et al., 2011). Several GHL17 proteins were isolated through these screens, including the predicted membrane localized proteins At3g13560, At5g42100, At4g31140, and At5g58090. Different from At3g13560 and At5g42100 (included in the alpha clade), At4g31140 and At5g58090 were found in clade beta. Successful separation of PD membranous section from the desmotubule and the PM is quite challenging (if not impossible) therefore a number of false positives was expected. The results presented above suggest that proteins excluded from the alpha clade are not likely targeted to PD sites. Therefore, we tested the intracellular localization of At4g31140 and At5g58090 using as control At3g13560-mCitrine (a previously PD-localized GHL17 protein). m-Citrine fluorescent fusions were obtained and expressed transiently in tobacco leaves. The results are shown in Figure 5. Transient expression of either At4g31140-mCit or At5g58090-mCit led to protein accumulation in the apoplast (Figures 5A–C). At5g58090-mCit also appears to be associated with the endoplasmic reticulum (data not shown).

Transient assays can be misleading. Therefore we obtained stable transgenic lines expressing p*35s*-At5g58090-mCit to confirm the subcellular localization of this protein. Leaves isolated from 10 days-old seedlings expressing p*35s*-At5g58090-mCit and leaves isolated from seedlings overexpressing At3g13560-mCit (grown in the same plate) were stained with aniline blue to reveal callose deposits at PD sites. The intracellular localization of these proteins in stable lines reproduced the results obtained in transient assays (Figures 5D,E): At5g58090-mCit was found at the cell periphery and in the apoplast whereas At3g13560-mCit was found in a punctuated pattern along the cell wall (presumably PD sites). Co-localization with callose deposits at PD was found for At3g13560 but not for At5g58090 (white arrows in Figures 5D,E). This result suggests that PD localization of GHL17 proteins could be related to their evolutionary origin, hence with the appearance of the alpha clade.

## Discussion

GHL17 proteins play many different roles in plant development and response to biotic and abiotic stresses (Doxey et al., 2007). Functional specialization can be predicted by studying protein sequence, gene expression and phylogeny (Doxey et al., 2007). Here, we used phylogenetic tree reconstruction to study when in land plant evolution GHL17 membrane proteins diversify to play a role at PD. First, we identified sequences encoding for a GH17 domain in representatives of green algae, fungi, bryophytes and vascular plants. Fungi, as plants, deposit callose at the cell wall but don't form plasmodesmata connections. Therefore they are an ideal organism to analyze the evolution of 1,3 beta glucanases in a different lineage.

Study of the protein sequences isolated suggests that the key amino acids involved in GH17 catalytic activity are highly conserved throughout evolution. This is in agreement with other reports that demonstrate the presence of beta 1,3 glucans in the cell wall of ancient unicellular algae where it is required for cell division and cell wall biogenesis (Scherp et al., 2001; Sorensen et al., 2011). Specialization of GHL17 proteins to play specific roles in the control of PD transport is therefore likely a consequence of evolutionary functional diversification within this family.

Classification of embryophytes GHL17 proteins according to the presence or absence of a signal peptide, of a GPI-anchored domain and of one or more carbohydrate binding domain (X8) predicted PM or PD localization for a set of proteins. The number of membrane predicted proteins increased from 4 identified in moss to 21–22 identified in vascular plants suggesting that an expansion occur in this protein family during land plant evolution. This might have been necessary to support the adaptation of multicellular organism to terrestrial environments, which might require specialized GHL17 proteins to assume divergent or redundant functions at the PM or membraneous subdomains.

Using phylogenetic analysis we found that membrane-targeted sequences are evenly distributed in two major clades (Figure 3). Clade alpha contained GHL17 sequences that appeared in embryophytes only whereas the beta clade comprised land plants and algae proteins and is closely related to a branch containing fungi sequences. This result suggest that clade alpha evolved early during land colonization in the Streptophyte lineage, whereas clade beta is form by proteins of a more ancestral origin (Figures 3B,C). Ultrastructural studies revealed the accumulation of callose at PD sites in early embryophytes (Scherp et al., 2001; Schuette et al., 2009) therefore GHL17 proteins participating in the regulation of callose at PD sites will likely appear in clade alpha.

Indeed, we noticed that all Arabidopsis PD-located GHL17 proteins (identified up to date) are clustered in the alpha clade. This established an interesting link between the phylogenetic distribution of GHL17 proteins and their intracellular localization. This correlation was confirmed in Populus: membrane proteins belonging to the alpha clade were reported to localize at PD but this was not the case for proteins contained in other clades (Rinne et al., 2011). We tested the use of this knowledge for the discrimination of false positives isolated in a proteomic screen of Arabidopsis PD. Two proteins from the beta clade were identified in the PD proteome but intracellular localization of mCitrine protein fusions revealed that they accumulate in the apoplast (Figure 5). Our results suggest that phylogenetic analysis could be potentially a useful tool for the preliminary detection of false positive when screening for PD-localized GHL17 proteins.

To summarize, the results obtained so far suggest that, during (or immediately after) colonization of terrestrial habitats by streptophytes, GHL17 gene family evolved and expanded to play specialized roles at the cell membrane, including PD regulation. Completion of genome sequence and further studies on callose regulation in ancestral charophyceans will be essential to confirm or refute this theory. Study of phylogenetic relationships between ancestral PM targeted GHL17 and those that evolved with embryophytes was used here to discriminate between PD-localized and non PD-localized proteins in Arabidopsis and Populus. This knowledge could theoretically be applied to the preliminary screening of GHL17 proteins (aiming to identified those that serve specialized roles are PD sites) in other land plant representatives.

## Author contributions

Rocio Gaudioso-Pedraza performed the research, analyzed the data and designed the Figures. Yoselin Benitez-Alfonso designed the experiments, wrote the manuscript, performed research and interpreted the data for the work.

### Conflict of interest statement

The authors declare that the research was conducted in the absence of any commercial or financial relationships that could be construed as a potential conflict of interest.
