# Supplementary material for: A phylogenetic approach to study the origin and evolution of plasmodesmata-localized glycosyl hydrolases family 17
Source: Front Plant Sci. 2014 May 23;5:212. doi: 10.3389/fpls.2014.00212 (PMC4033164; doi:10.3389/fpls.2014.00212)
Supplement: Supplementary file 1 [file DataSheet1.DOCX]

Alignment of GHL17 sequences from Green algae and fungi representatives, moss, Arabidopsis and rice.

KfGHL17_1 -------------------------------------IPGKFVNSRD------------------

KfGHL17_2 -----------------------------------------------------------------

PmGHL17_1 -----------------------------------------------------------------

CgGHL17_1 -----------------------------------------------------------------

NtGHL17_1 -----------------------------------------------------------------

NtGHL17_2 -------------------------------MAAPRNSGFGLDCWLLVSATFVLKFFLWL-----

NtGHL17_3 -------------------MVMAVSRRRQQALSLFHSAAPRPLPLPLHPTRMPLSNLMGLTTTTA

PpGHL17_1 -------------------------------MSSSSTRSVTPSFTAS--------EFYFI-----

PpGHL17_2 ---------------------------MDKAMRTSMHGRSCLKLAIF--------VVLGC-----

PpGHL17_3 -----------------------------------------------------------------

PpGHL17_4 -----------------------------------------------------------------

PpGHL17_5 ---------------------------------MMRGGMQWGFAAVV--------VGLVY-----

PpGHL17_6 --------------------------------MHSSRKQGFFLKVVI--------LSACH-----

PpGHL17_7 -------------------------------------MAVRVAHMMR--------IFQAI-----

PpGHL17_8 -----------------------------------------------------------------

PpGHL17_9 --------------------------MLITLIFLLDHRHGYRERDCA--------CGYSFGGRR-

PpGHL17_10 ---------------------------------MAREPGVWRVVVVT------------------

PpGHL17_11 -------------------------------------MATLGGIVFV--------AALIV-----

PpGHL17_12 --------------------------------------MTASAAICV------------------

PpGHL17_13 -------------------------------------MVGSRIAWCC--------VSMGM-----

PpGHL17_14 ------------------------------MVAVAGRQALAFARVAA--------VVLLC-----

PpGHL17_15 ------------------------------MAGHRMQSRQVFTSSVA--------VVALC-----

PpGHL17_16 -----------------------------------------------------------------

PpGHL17_17 ---------------------------------MGSVEVGSDVPDLH--------YGGGK-----

PpGHL17_18 -----------------------------------------MENHVM------------------

CaGHL17_1 --------------------------------------MQIKFLTTL------------------

AcGHL17_1 ---------------------------------------MRVSTLLP----------IAL-----

AfgHL17_1 ----------------------------------------MRVSTLL--------PLALA-----

AnGHL17_1 -------------------------------------MRASSLLPAL------------------

CglGHL17_1 ---------------------------------------MRFST-------------VAA-----

DhGHL17_1 -------------------------------------------MQLK--------YLTVL-----

AgGHL17_1 -------------------------------------MRFSATVSAA------------------

FgGHL17_1 -----------------------------------MKFFSTLSTLAV------------------

KlGHL17_1 ---------------------------------------MRFSTLVS----------ATL-----

ScGHL17_1 ---------------------------------MRFSTTLATAATAL------------------

SsGHL17_1 ---------------------------------------MQIKY-------------LTA-----

SpoGHL17_1 ------------------------------------MQFLSSFVFAA--------LALLP-----

YlGHL17_1 ---------------------------------------MKFTFAAV------------------

AT3G57270.1 --------------------------------------MDLRFLASL--------TLLLG-----

AT3G57260.1 ---------------------------------MSESRSLASPPMLM--------ILLSL-----

AT3G57240.1 ------------------------------MKMCNGSSFLASLPLLL--------LLLSF-----

AT4G16260.1 -----------------------------------------MTTLFL--------LIALF-----

AT5G56590.1 ---------------------------------------MARDFKLI--------FSISI-----

AT1G77790.1 -------------------------------------MDSKLIRFAV--------VIMLL-----

AT1G77780.1 -------------------------------------------MFLI--------ATLLF-----

AT4G26830.1 ----------------------------------------MAVSFLP--------YFLIL-----

AT4G29360 --------------------------------------MGQRLNLVF--------WIFVS-----

AT3G07320 ----------------------------------------MSLLLHL--------FALSL-----

AT2G16230 ----------------------------------------MALSILF--------LLLFI-----

AT5G20340 -------------------------------------MLYLPKKLFL--------FFFSC-----

AT2G05790 ----------------------------------------MDLLTPL--------IFLLL-----

AT5G55180.1 --------------------------------------MAVFVLSLL--------ILSSF-----

AT5G20330 -------------------------------------MLYSPKKLFL--------FFLSC-----

AT2G01630 ------------------------------------------MAALL--------LLFLF-----

AT4G34480 ---------------------------------------MALSISIY--------FLLIF-----

AT1G66250 --------------------------------------MASLLHLLL--------LSLSL-----

AT5G20390 -------------------------------------MDCHRKTFLL--------KFLCV-----

AT3G61810 -------------------------------MKMILMTTMQNAFWIL--------LVFSI-----

AT1G32860 ------------------------------------MELTSFHRSSL--------LFLIS-----

AT2G27500 ------------------------------------MATHSLSFFFR--------VLLLL-----

AT1G33220 -------------------------------------MVYSSKKLFL--------FFLSC-----

AT3G15800 -----------------MGYCVPLRK-----------SPTSHMVFSS--------FFLSF-----

AT3G23770 ---------------------------------------MTPFALFL--------FTLLA-----

AT5G42720 ---------------------------------------MRASVYSL--------ILLFF-----

AT2G26600 -------------------------------------MRSSKLLLLK--------FFFFF-----

AT4G14080.1 ---------------------------------------MSLLAFFL--------FTILV-----

AT3G46570 -------------------------------------MLGSRVVHQL--------YFLIL-----

AT4G18340 -------------------------------MTSRTFTRHSSLIHVF--------LLLSL-----

AT1G30080 -----------------------------MSNMFSRIAMTNSIVLLL--------FSLTF-----

AT5G42100 --------------------------------------MASSSLQSL--------FSLFC-----

AT5G58090 --------------------------------------MGWGSVLLL------------------

AT1G11820 ---------------------------------MAFTSMVSTVPVLF--------FFFTL-----

AT3G24330 --------------------------MAGERSKLTTNHFYNHQIILC--------YFLII-----

AT4G31140.1 --------------------------------------MLFKGVFAV--------FFVIT-----

AT4G17180 ----------------------------------------MGSGVGV--------ALFAL-----

AT3G13560 ---------------------------------------MLLPRWFA--------EALLL-----

AT5G24318 -----------------------------------MNYVLPFFSLSM--------FTIVG-----

AT2G39640 -----------------------------------MAKTIRSFILPF--------LLIVA-----

AT2G19440 ---------------------------------------MNLLAFVV------------------

AT3G04010 --------------------------------MNYRRKQSAITNSAV--------VFIII-----

AT5G18220 -----------------------------------MSNRRKQSTTAG--------IFLCI-----

AT1G64760 -----------------------------------------MSNLLA--------LVVGF-----

AT5G58480 ----------------------------------------MARRLFL--------LLLAV-----

AT3G55430 -----------------------------------MAKAPPSISL-L--------LLLCA-----

AT5G20870 -----------------------------------MSSHDTYQKLIL--------FCLSI-----

AT3G55780 -----------------------------------MKKMHSLSSYLL--------LLISL-----

AT5G64790 -------------------------------------MAGRAAMLVN--------VGTVI-----

AT5G20560 -------------------------------------MEDYSKNLFL--------LLFSC-----

OsGHL17_1 --------------------------MAAGEQPGTGSPPQPSTRLFL--------LVLAV-----

OsGHL17_2 ----------------------------------MELCVRASSLLLG--------LLLQL-----

OsGHL17_3 -------------------------------MATAARAASCTPAACA--------VLVIP-----

OsGHL17_4 ---------------------MPRTARKARVLLLSSPPPPPSPPVMLRRGGVVLLLVVVV-----

OsGHL17_5 MPRPGTEATTTTTSGCEADCGVPAMKATLMMMSCRSRSRSGRAHWML--------LLFCL-----

OsGHL17_6 -------------------------------------MAHVAFVALL------------------

OsGHL17_7 ----------------------------------MAGRRQLLLLLLL--------WGFLQ-----

OsGHL17_8 ---------------------------------------MKKLFFFL--------FLLIL-----

OsGHL17_9 ---------------------------------MLEHRNMWPASLKG--------VVILL-----

OsGHL17_10 --------------------------------MGSRSRGGRHLLLLL--------LLHLLSLHSS

OsGHL17_11 --------------------------------------MAAATVWRC--------AVVVG-----

OsGHL17_12 --------------------------------MAPPPRLPAGAAALL--------LLLAV-----

OsGHL17_13 ---------------------------------------MARLVVVI--------VAVAM-----

OsGHL17_14 ------------------------------------MGSCARVHALA--------VAWAV-----

OsGHL17_15 ---------------------------------MASRSGDRRLAVVA------------------

OsGHL17_16 -----------------------------------MAAAAAAVGVVA--------LLLLL-----

OsGHL17_17 ----------------------------------------MGAFAAA--------LLALL-----

OsGHL17_18 ---------------------------------MAAANAGAMVRFVA--------AFLAA-----

OsGHL17_19 ------------------------------------MAPAPLAGGRL--------VLVVV-----

OsGHL17_20 ----------------------------------MGSSWWWHGRAAA--------AVACW-----

OsGHL17_21 -------------------------------MADLAVLQWWLGAMVA--------MAAAA-----

OsGHL17_22 -------------MIWSASVPWVVVIAVFTLHATLGRRVHIFFVRLLLLLLLSHFSFLLWARREL

OsGHL17_23 -----------------------------------MGARRWLDAAVT--------SGLLQ-----

OsGHL17_24 ------------------------------MRPSRRRHPLPPLAPGL--------LLILL-----

KfGHL17_1 ------------GRSNSTASVG-------------------ANYV-GVNWGV----LSSQPLPGN

KfGHL17_2 -----------------------------------------------------------------

PmGHL17_1 --------------------------------------------------------L--------

CgGHL17_1 ------------SRRRRPSTSS------------------------GSSWGS-------------

NtGHL17_1 --------------------------------------------------------M--------

NtGHL17_2 ------------GLGAQCCFAQ------------------VPSAVIGINYGR----VAFDTIKAS

NtGHL17_3 AMEPYGKICCALVMLSLVQFIS-------------------AAQI-GVGFGM----VGRNVLPNS

PpGHL17_1 ------------TTLPLTDCTG--------------------GSV-GVNYGT----LGNNLPTPS

PpGHL17_2 ------------SCFSFAVGTG--------------------GTV-GVNYGT----LGNNLPSSA

PpGHL17_3 ------------MIRCLVSVDD-------------------AATI-GIGYGT----SGDNLPPTE

PpGHL17_4 ------------MSCVLYFAEA--------------------RTI-GINYGR----LGDNLPTAS

PpGHL17_5 ------------LFHAAVFAEA---------------------NI-GINYGR----VADNLPRPK

PpGHL17_6 ------------FFISQLASVN-----------ADNGYAPQAHTI-GINYGT----LGDNLPPPS

PpGHL17_7 ------------SIFILISSAE--------------------ASI-GVNYGT----YGDNLPTPT

PpGHL17_8 ------------MLVCVYIDAT---------------------AI-GAVYGR----NGNNIPDAT

PpGHL17_9 ------------CTRFHCGCNG------------------------GINVGR----VGDNLPGPG

PpGHL17_10 ------------LLFLVVSQTV--------------------NGL-GVNWGT----ISVNPLPPG

PpGHL17_11 ------------VAMEPVLTAS--------------------ATI-GIAIGV----VGNDLP---

PpGHL17_12 ------------------------------------------GGL-GVNWGN----QAFNPLPPK

PpGHL17_13 ------------LLLMGASQFV--------------------SGL-GVNWGT----MSVNPMPPG

PpGHL17_14 ------------ITGWQLGKGN--------------------GEL-GVNWGT----ISHDPLPNN

PpGHL17_15 ------------LVGWQMEMAV--------------------ADV-GVNWGT----ISSDPLSND

PpGHL17_16 -----------------METAV--------------------AEL-GVNWGR----ISSDPLSND

PpGHL17_17 ------------GSEKHRDRCP--------------------TTV-GICYGR----VADNLPSPP

PpGHL17_18 ----------------------------------------------GINYGR----IANDLPAPD

CaGHL17_1 ------------ATVLTSVAAM--------------------GDL-AFNLGVKND-DGTCKDVST

AcGHL17_1 ------------AAGTAVASKQ--------------------GTL-GFALGNKNA-DSSCKSQSD

AfgHL17_1 ------------AGTAAASKN---------------------GTL-GFALGNKNE-GGKCKVQSD

AnGHL17_1 ------------LAAIPAVSAS--------------------GTL-GLSIGDKNG-DSSCKSTSD

CglGHL17_1 ------------LAFGASQVAA-------------------IGEL-AFNLGVK-NNDGTCKTTND

DhGHL17_1 ------------AAAVSSVSAI--------------------GDL-AFNIGVKDN-SGNCKSIEE

AgGHL17_1 ------------LLGASSVHAM--------------------GDL-AFNLGVKKH-DGTCKYTQD

FgGHL17_1 ------------ALMMTGEAVA--------------------GTYKGFSMGANRA-DGVCKWEAD

KlGHL17_1 ------------LAGASTVAAV--------------------GEL-AFNLGVKTH-DGSCKQKDD

ScGHL17_1 ------------FFTASQVSAI--------------------GEL-AFNLGVKNN-DGTCKSTSD

SsGHL17_1 ------------VAALLASASA-------------------IGDL-GFNLGVK-DNAGNCKTADE

SpoGHL17_1 ------------LSAMAVDEAA-------SEIASSTKPASTNGTL-SFCLGVKHA-DGTCKYTDD

YlGHL17_1 ------------TAALASSAMA-------------------LGGL-GVDLGVKRESDGECKNAGD

AT3G57270.1 ------------LFFVNTNPTG--------------------GQV-GVCYGR----NGNNLPSPA

AT3G57260.1 ------------VIASFFNHTA--------------------GQI-GVCYGM----LGDTLPSPS

AT3G57240.1 ------------ILASFFDTAV--------------------GQI-GVCYGR----NGNNLRPAS

AT4G16260.1 ------------ITTILNPTSG--------------------ESV-GVCYGM----MGNNLPSQS

AT5G56590.1 ------------LLLLLDCCYG--------------------GKV-GVCYGR----SADDLPTPS

AT1G77790.1 ------------SIQIFCTAGV------------------AGDIT-GDCYGR----NGNNLPTPA

AT1G77780.1 ------------LSARLTTAGN------------------MNSFA-GVCYGR----NGDNLPSPA

AT4G26830.1 ------------SFLSAIDAHS--------------------GMV-GVNYGR----IANNLPSPE

AT4G29360 ------------ILAFLNFGMA--------------------SKI-GICYGR----NADNLPSPN

AT3G07320 ------------LISVSGAKFS--------------------GRP-GINYGQ----LGNNLPSPS

AT2G16230 ------------LFSISPSNAQ--------------------SFI-GVNYGL----LSDNLPPPS

AT5G20340 ------------IVVIVNYNNS-----DFVNAANSIGFVNAANSI-GLNYGL----LGDNLPSPS

AT2G05790 ------------LLSPFSLSDA--------------------GSI-GVNYGR----ISDELPSAF

AT5G55180.1 ------------SAIPFTYADS--------------------GMI-GVNYGR----IADNLPAPE

AT5G20330 ------------IVLYVNSNNS--------------GFVTAANSI-GLNYGL----LGDNLPSPS

AT2G01630 ------------LFASSALSQD--------------------SLI-GVNIGT----EVTNMPSPT

AT4G34480 ------------LSHFPSSHAE--------------------PFI-GVNYGQ----VADNLPPPS

AT1G66250 ------------LVLASASPSP---------------PADEGSYI-GVNIGT----DLSDMPHPT

AT5G20390 ------------AFLLNYSNVG---------------FVDAATNI-GLNYGL----LGDNLPPPS

AT3G61810 ------------SMASCSTIPSLVGPQIGINYGEYSSNLEDSSPVIGINYGR----YGSNLPPPE

AT1G32860 ------------LTLIILPTTT--------------------TSI-GVNYGQ----IGDNLPSPT

AT2G27500 ------------FLTLSERIKG--------------------QGV-GINYGQ----IANNLPSPA

AT1G33220 ------------IMLTFNYNTS--------------GFVAAANSI-GLNYGL----LGDNFPTPS

AT3G15800 ------------LLVFSILSSQ--------------TAVAFIGTY-GVNYGR----IADNLPSPD

AT3G23770 ------------LSSSCCSAIG---------PQNNRTVLALASRI-GINYGK----LGNNLPFPY

AT5G42720 ------------SCLLHLSKSQ--------------------PFL-GVNYGL----TADNLPPPS

AT2G26600 ------------FFNLTSLEYQ--------------VDGAFVGTY-GINYGR----IADNIPSPE

AT4G14080.1 ------------FSSSCCSATR----FQGHRYMQRKTMLDLASKI-GINYGR----RGNNLPSPY

AT3G46570 ------------TLILPFSAAY---------------------QV-GVNYGT----VANNLPPPH

AT4G18340 ------------VFSGNILQTV--------------------TSL-GINYGQ----VGNNLPSPD

AT1G30080 ------------LEHGLLFQRV--------------------SSL-GINYGQ----VGDNLPPPD

AT5G42100 ------------LALFSLPLIV--------------------SSI-GINYGQ----VANNLPPPK

AT5G58090 ------------LAVALLCQRA--------------------SSI-GANWGT----QASHPLPPD

AT1G11820 ------------LLISANSSSL--------SHNIKVQEQDKDPFV-GFNIGT----DVSNLLSPT

AT3G24330 ------------SQVSIASSNT--------------------SNV-GVNWGI----MASHQLPPE

AT4G31140.1 ------------LLYASLLIEV--------------------EGI-GVNWGS----QARHPLPPA

AT4G17180 ------------SLLLVSHEVE--------------------SAI-GVNWGT----LSFHKMRPS

AT3G13560 ------------LLSILACSNA--------------------AFI-GVNIGT----DLTNMPPPS

AT5G24318 ------------VLLILSTGSE--------------------ASI-GVNYGT----LANNLPPPQ

AT2G39640 ------------GVIFQLSAVT--------------------SAI-GINYGT----LG-NLQPPQ

AT2G19440 ------------GFGIMGIVMV--------------------DGL-GVNWGT----MATHKLPPK

AT3G04010 ------------SAVCFLSGGV--------------------SGL-GVNWGT----MASHQLPPK

AT5G18220 ------------TIVSLLSGDV--------------------SAL-GVNWGT----MSTHQLPPK

AT1G64760 ------------VIVIGHLGIL-------------------VNGL-GVNWGT----MATHKLPPK

AT5G58480 ------------TAGLSLTGTT-------------------VRAV-GINWGT----EASHPLPPS

AT3G55430 ------------AVFLTIPAVI--------------------SAI-GVNYGT----LG-NLPPPT

AT5G20870 ------------FFLQNILKNV--------------------EGL-ACNWGT----QASHPLPPN

AT3G55780 ------------TAIATPTTTS-------------------ATTI-GVTYSTPASISGTVQLSPD

AT5G64790 ------------MTVLTLASLV--------------------GGF-GVNWGN----IASHPLNPN

AT5G20560 ------------TALIISYYNV--------------DSLSTASVV-GLNYGL----LGDNLPSPS

OsGHL17_1 ------------ILTDQVLAAS-----------------AQGMSI-GINYGQ----IADNLPSPT

OsGHL17_2 ------------LSSVDVVSAA--------------------QKF-GINYGQ----IANNLPDPT

OsGHL17_3 ------------ILVLIMAGQV---------------RVAEALSI-GVNYGQ----IANNLPSPS

OsGHL17_4 ------------LGLLAARCDG-------------KKVSSFVGTY-GVNYGR----IADNLPPPT

OsGHL17_5 ------------LLAFPSHGPR--------------AVEAFPGGY-GINYGR----IANNIPSPD

OsGHL17_6 ------------FLLLVGHCLG--------------------GKV-GICYGR----NADDLPAPD

OsGHL17_7 ------------LIRLPYSASQ--------------------SFI-GINYGD----VADNLPPPA

OsGHL17_8 ------------AASVVHGEDG--------------------AYI-GVNIGT----AMTSVPAPT

OsGHL17_9 ------------MLMVFNVSGA---------------------FV-GINVGT----DISNPPSAS

OsGHL17_10 ------------LLPWAAVSAA-------------GGGGSGDPYV-GVTIGT----AVTNLLSPS

OsGHL17_11 ------------VVIMAAAAVV--------------------DGL-GVNWGT----MATHRLPPK

OsGHL17_12 ------------ASRAAADGNA--------------------VDV-GVNWGS----QLSHPLLPK

OsGHL17_13 ------------AAWWAVAAVE---------------------GL-GINWGT----QATHPLPPK

OsGHL17_14 ------------AALLSRATPA--------------------GAL-AANWGT----RALHPLPGD

OsGHL17_15 ------------FAAAVLLSAA--------------------EGL-GVNWGT----MASHPLPPR

OsGHL17_16 ------------PLAASAYGDG-----------------LGRAAV-GVNWGT----MTSHPILPC

OsGHL17_17 ------------LAAMAAPPAE--------------------AAV-GVNWGT----LSSHRVPPP

OsGHL17_18 ------------VLVMMVPCAP----------------RAAAAAV-GVNWGT----VSAHRMPAP

OsGHL17_19 ------------VAAACMARWS--------------------EGI-GVNWGT----QLSHPLPAS

OsGHL17_20 ------------VWLAAAAAVG-------------------VEAI-GANWGT----QASHPLAPD

OsGHL17_21 ------------SWSGGVLPAA--------------------EAL-GMNWGT----QASHPLPPK

OsGHL17_22 ESMEAESRKLLLALAVSLCCFV--------------AASRAQSYI-GVNYGE----VADNLPAPE

OsGHL17_23 ------------ALLFHLATSQ--------------------SFI-GVNYGT----IADNLPPPA

OsGHL17_24 ------------LHALAPPPAA--------------------AAV-GVNWGF----SSSHPLPAA

KfGHL17_1 VTVRI--LQD-AG-FTSVKFF-----SAPPEYLRAL-AN-T---S----IEVQVLMPLDQVMNVS

KfGHL17_2 ------------------------------------------------------MVPNNYI----

PmGHL17_1 -------------------------------------AD-Q------------------------

CgGHL17_1 -----------------------------------------------------------------

NtGHL17_1 -------------------------------------AG-T---G----VEVVLGIPLGQV----

NtGHL17_2 IAVEI--MKR-NG-IKKVKIY-----NADPRVMKALMAGDF--------TDVMITVTNEEL----

NtGHL17_3 QALTI--LRD-RQ-CYLLKTW-----SIDPNWLDEVERVYA---GTSQQVEVTVAIPNSEL----

PpGHL17_1 QVAQL--LLS-TS-LRNVKIY-----NADKAIMEAF-AN-T---N----IRLVVGIGTESI----

PpGHL17_2 QVAQL--LLS-TS-LRNVKIY-----NADKAIMEAF-AN-T---N----IKLVVGIGTESI----

PpGHL17_3 QVVQF--LKT-LN-VTKVKIY-----NTDANVIRAF-AN-S---G----MDLSITVPNGDI----

PpGHL17_4 ETVTL--IKN-LG-IGRVRIF-----DHDGPTIKAF-AG-S---G----LEFIIGMGNDEI----

PpGHL17_5 DVAKL--VQS-IG-VKHIKIF-----DYEKEIIRAF-DH-T---G----ISLIVCVPNQEI----

PpGHL17_6 AAIAT--IKS-MQ-IGRVKIF-----NPNADILNAL-AN-S---G----LETVVAIPNDQI----

PpGHL17_7 QAVAL--LKK-SG-VTQARIY-----DTNPSVLNAF-QG-S---N----IQLVVGVRNDEI----

PpGHL17_8 TAAAL--MQQ-YD-ISRVRIF-----DHDPSIIQAF-AS-T---Q----IRVMIAVTNEEI----

PpGHL17_9 RIVEL--LQR-----RKVRIY-----DADLKMLTAF-KG-S---G----IKVTVAVPNDAV----

PpGHL17_10 YVVKM--LQA-NG-IKKVKLF-----DAAYDVIRAL-AG-T---D----IEVMVAAPNNLL----

PpGHL17_11 -------SRA----VTNVRIY-----NADREMLTAF-KN-S---S----IIVTVAVPNYSV----

PpGHL17_12 DVVKL--LQM-NS-VTKVKIF-----DANYDILKSL-VG-S---G----IEVMVAAPNYAL----

PpGHL17_13 YVVKM--LQA-NG-IKKVKLF-----DADHDVVKSM-AG-T---D----IEVMVAAPNDLL----

PpGHL17_14 IVVKL--LQD-NN-FAKVKLF-----DADPNVIESM-RG-T---N----LEVMVAITNDML----

PpGHL17_15 IVVQM--LKD-NN-FVKVKLF-----DANSDVIESM-RG-T---N----LEVMIAITNEML----

PpGHL17_16 VVVQM--LKD-NN-FVKVKLF-----DANSEVIESM-RG-I---N----LEVMVAITNDML----

PpGHL17_17 EVVSL--LRS-RG-VTDVKIY-----DAAGDILRAF-EN-S---G----IILSVAVPNEEV----

PpGHL17_18 EAIQQ--IRT-MK-IGRVKIF-----NSDATVLSAL-AN-T---G----LEVVSGLPNEDI----

CaGHL17_1 FEGDLDFLKS-HS--KIIKTYA----VSDCNTLQNLGPA-AEAEG----FQIQLGIWPNDD----

AcGHL17_1 YEKDF--DAL-KGVTTLVRTYSASDCDTAKNIIPAA-KAKQ--------FKVVLGVWPDYD----

AfgHL17_1 YETDFDTLKE-VT--SLVRIYSASDCDTAKHIIPAA-KA-K---N----FKVVLGVWPDYD----

AnGHL17_1 YEADFDALKS-VT--TLVRTYSASDCNTAQNIVPAA-KA-K---G----FKVVLGVWADYD----

CglGHL17_1 YENDL--KVL-KSYTSTVKVYAASDCNTLENLGPAA-EA-E---G----FNIFLGIWPNDE----

DhGHL17_1 YESDFSVLES-QS--KIVKAYAVSDCNTLQNLGPAA-EE-A---G----FQVMFGIWPNDD----

AgGHL17_1 YLDDFEALRP-YT--DTVKVYS----TSDCNTLQFLGPA-ADRAG----FKLWVGVWPDDD----

FgGHL17_1 WKKDFQAIKSWNKGFNAVRLYSASDCNTLVKAVPAA-KA-T---G----MKILVGIWSTDD----

KlGHL17_1 YLADF--ETL-KGYTSKVKVYA----ASDCNTLQILGPA-AEEAG----FTIFLGIWPTDS----

ScGHL17_1 YETELQALKS-YT--STVKVYA----ASDCNTLQNLGPA-AEAEG----FTIFVGVWPTDD----

SsGHL17_1 YAADL--KNI-AGYSKVVKTYAVSDCNTLQILGPAA-ED-A---G----FQVMLGIWPTDS----

SpoGHL17_1 YLADFEVLAP-YT--NMIRTYATSDCNTLEYLLPALAQSPY---N----FSAILGVWPTDD----

YlGHL17_1 YKADL--EAL-KGLTDTIRIYA----AGDCDALREL-GPVAEAAN----FKLMIGVWPNDD----

AT3G57270.1 ETIAL--FKQ-KN-IQRVRLY-----SPDHDVLAAL-RG-S---N----IEVTLGLPNSYL----

AT3G57260.1 DVVAL--YKQ-QN-IQRMRLY-----GPDPGALAAL-RG-S---D----IELILDVPSSDL----

AT3G57240.1 EVVAL--YQQ-RN-IRRMRLY-----DPNQETLNAL-RG-S---N----IELVLDVPNPDL----

AT4G16260.1 DTIAL--FRQ-NN-IRRVRLY-----DPNQAALNAL-RN-T---G----IEVIIGVPNTDL----

AT5G56590.1 KVVQL--IQQ-HN-IKYVRIY-----DYNSQVLKAF-GN-T---S----IELMIGVPNSDL----

AT1G77790.1 DTVAL--YKS-NN-IDAIRMY-----EPFADMLEAL-RG-S---G----LLVAFGPRNEDI----

AT1G77780.1 KTVSL--YKK-IN-VGGIRLY-----EPFPDLIVSL-QG-T---G----LLVAIGPRNEAI----

AT4G26830.1 KVVNL--LKS-QG-INRIKIF-----DTDKNVLTAL-AN-S---K----IKVIVALPNELL----

AT4G29360 RVSEL--IQH-LN-IKFVRIY-----DANIDVLKAF-AN-T---G----IELMIGVPNADL----

AT3G07320 DSVNL--IKS-LN-AKRVKLY-----DANPKILAAL-NG-T---D----ITVSVMVPNELL----

AT2G16230 QTAKL--LQS-TS-IQKVRLY-----NADSSIITSL-VG-T---G----IGIVIGVANGDL----

AT5G20340 KVITL--YKS-ID-ITKIRIF-----DPNTEVLNAL-RGHR---D----IAVTVGVRDQDL----

AT2G05790 KVVQL--LKS-QG-ITRVKIF-----DADPSVLKAL-SG-S---G----IKVTVDLPNELL----

AT5G55180.1 KVVEL--LKT-QG-INRIKLY-----DTETTVLTAL-AN-S---G----IKVVVSLPNENL----

AT5G20330 NVINL--YKS-IG-ISRIRIF-----DPNTEVLNAL-RGHR---D----IEVTVGVKDQDL----

AT2G01630 QVVAL--LKS-QN-INRVRLY-----DADRSMLLAF-AH-T---G----VQVIISVPNDQL----

AT4G34480 ETVKL--LQS-TS-IQKVRLY-----GADPAIIKAL-AG-T---G----VGIVIGAANGDV----

AT1G66250 QVVAL--LKA-QE-IRHIRLY-----NADPGLLIAL-AN-T---G----IKVIISIPNDQL----

AT5G20390 EVINL--YKS-LS-VTNIRIF-----DTTTDVLNAF-RGNR---N----IGVMVDVKNQDL----

AT3G61810 AIPSL--VNS-LS-IKHVKTF-----DLDPRITKSF-AN-T---G----ITLSLCIPNDKI----

AT1G32860 DVIPL--IKS-IG-ATKVKLY-----DANPQILKAF-SN-T---G----IEFIIGLGNEYL----

AT2G27500 RVAVL--LRS-LN-ITRVKLY-----DADPNVLFSF-SN-S---Q----VDFMIGLGNEYL----

AT1G33220 NVLNL--YKS-IG-ITKIRIF-----DPKTEVLNAL-RGHR---N----IEVTVGVRDQDL----

AT3G15800 AVATL--LKS-AK-IRNTRIY-----DADHSVLTAF-RG-T---G----IEIIVGLGNEFL----

AT3G23770 QSINL--IKT-IK-AGHVKLY-----DADPETLKLL-ST-T---N----LYVTIMVPNNQI----

AT5G42720 ASAKL--LQS-TT-FQKVRLY-----GSDPAVIKAL-AN-T---G----IEIVIGASNGDV----

AT2G26600 KVVLL--LKQ-AK-IRNVRIY-----DVDHTVLEAF-SG-T---G----LDLVVGLPNGFL----

AT4G14080.1 QSINF--IKS-IK-AGHVKLY-----DADPESLTLL-SQ-T---N----LYVTITVPNHQI----

AT3G46570 QVVNF--IKT-KTIINHVKIF-----DTNHDILAAF-SGIT---G----ISLTVTVPNSDI----

AT4G18340 KVINL--LRS-LR-ITKTRIY-----DTNPQILSAF-AN-S---N----IEIIVTIENQVL----

AT1G30080 KVLQL--LSS-LH-INKTRIY-----DTNPRVLTSF-AN-S---N----IELFVTVENEML----

AT5G42100 NVIPL--LKS-VG-ATKVKLY-----DADPQALRAF-AG-S---G----FELTVALGNEYL----

AT5G58090 IVVRM--LRE-NG-IQKVKLF-----DAEYDTLRAL-GK-S---G----IEVMVGIPNEML----

AT1G11820 ELVKF--LQA-QK-VNHVRLY-----DADPELLKAL-AK-T---K----VRVIISVPNNQL----

AT3G24330 KVVKM--LMD-NS-FTKLKLF-----EADQNILDAL-IG-S---D----IEVMIGIPNRFL----

AT4G31140.1 TVVRL--LRE-NG-IQKVKLF-----EADSAILKAL-SR-T---G----IQVMVGIPNDLL----

AT4G17180 TVVDL--LKA-NK-ITKVKLF-----DANPDALRAL-MG-T---G----IQVMIGIPNEML----

AT3G13560 DIVTL--LKS-QQ-ITHVRLY-----DANSHMLKAF-AN-T---S----IEVMVGVTNEEI----

AT5G24318 QVAEF--LLH-STVINRIRLF-----DTDPQILQAF-AN-T---G----IAVTVTVPNDQI----

AT2G39640 QVVDF--IKT-KTTFDSVKIY-----DANPDILRAF-AG-S---E----INITIMVPNGNI----

AT2G19440 KVVQM--LKD-NN-INKVKLF-----DADETTMSAL-SG-S---G----LEVMVAIPNDQL----

AT3G04010 TVVEM--LKD-NN-IQKVKLF-----DADTNTMGAL-AG-S---G----VEVMVAIPNDLL----

AT5G18220 TVVQM--LKD-NN-VKKVKLF-----DADTNTMVAL-AG-S---G----IEVMVAIPNDQL----

AT1G64760 TVVQM--LKD-NN-INKVKLF-----DADETTMGAL-AG-S---G----LEVMVAIPNDQL----

AT5G58480 KVVEL--LKS-NG-IVKVKLF-----DADPKVLRAL-SG-S---N----IGVTIGIQNSML----

AT3G55430 QVANF--IKT-QTSIDSVKIF-----DVNPDILRAF-AG-T---G----ISVVVTVPNGDI----

AT5G20870 IVVKL--LRD-NG-FNKVKLF-----EADPGALRAL-GK-S---G----IQVMVGIPNDLL----

AT3G55780 RIAEK--VVS-MN-IPAVRLL-----DSNPAMIRAF-AY-T---N----VSLFLSVPNPLV----

AT5G64790 IVVQM--LKD-NK-INKVKLF-----DADSWTMNAL-AG-T---G----MEVMVGIPNNLL----

AT5G20560 NVIKF--YKS-QN-VAKIRIF-----EPNKDVLNAL-RGNR---D----IGVTVGIKNEDL----

OsGHL17_1 RVSGL--LRS-MQ-ISKVKLY-----DADQNVLSAF-LD-T---G----VEFVVGIGNENV----

OsGHL17_2 QVAGL--LQS-LN-VNKVKLY-----DADPKVLMAF-AN-T---G----VEFIIAIGNENL----

OsGHL17_3 RVSWL--LRS-LK-ISKVKLF-----DADPHVLRAF-LG-T---G----VEFVVGIGNEAV----

OsGHL17_4 EVVKL--LRM-AR-IKNVKIY-----DADHTVLDAF-RG-S---G----LNLVIAVTNGEV----

OsGHL17_5 KVVQL--LRA-SK-IRNVKIY-----DSDHSVLDAF-KG-S---G----LNLVIAIPNELV----

OsGHL17_6 KVVQL--IQQ-QS-LKYVRIY-----DTNIDVIKAF-AN-T---G----VELMVGVPNSDL----

OsGHL17_7 STARL--LQS-TT-ITKVRLY-----GTDPAVISAF-AG-T---G----ISLLLGAANGDI----

OsGHL17_8 QITTL--LRS-QN-IRHVRLY-----DADPAMLAAL-AN-T---G----IRVIVSVPNEQL----

OsGHL17_9 DTVSI--LKE-KK-IQHVRLL-----DSDHQMLSAL-AN-T---G----IEVVVGVPNDQL----

OsGHL17_10 DLPEF--LRA-QR-ITHVRLY-----DADPRMLSAL-AS-S---G----ARAIVGVPNDEL----

OsGHL17_11 VMARL--LKD-NG-FKKVKIF-----DADATTMSGL-AG-T---G----IEAMIAVPNDML----

OsGHL17_12 SVVQM--LKE-NG-ILKVKLF-----DADPWPVGAL-VD-S---G----IEVMLGIPNDML----

OsGHL17_13 AVVQL--LKD-NG-IAKVKLF-----DTDFAAMSAL-AG-S---G----VEVMVAIPNKDL----

OsGHL17_14 VTVRL--LRD-NG-FDKVKLF-----EADPSALRAL-GH-T---G----IQVMVGLPNELL----

OsGHL17_15 AVVRM--LQD-NG-ISKVKLF-----DADAGTMEAL-AG-S---G----VEVMVAIPNNLL----

OsGHL17_16 EVVRM--LAA-NG-VARVKMF-----DADPWTAAPL-AH-T---G----IQVMLAVPNDQL----

OsGHL17_17 VVVDL--LRA-NR-IGKVKLF-----DADPAVLRAL-AG-S---G----LQVMVGVTNAEL----

OsGHL17_18 VVVEL--MRA-NR-IGRVKLF-----DADQAALRAL-MG-S---G----LQVMVGITNEML----

OsGHL17_19 TVVQL--LKD-NG-FDRVKLF-----DAEDGILAAL-KG-S---G----IQVMVGIPNDML----

OsGHL17_20 TVVQM--LKD-NG-FDKVKLF-----DAGEDTMSAL-RK-S---G----LEVMVGIPNDML----

OsGHL17_21 IVAQL--LQD-NG-IKKVKLF-----DADQDTLSAL-AG-T---G----IEVMVAIPNVML----

OsGHL17_22 ETAKL--LKS-TT-ISKVRLY-----GVDPGIMRAL-AG-T---G----ISLVVGVANGDI----

OsGHL17_23 STANL--LKS-TS-IGKVRLY-----EPQPDLVAAL-AG-S---N----ISILLGVPNGDV----

OsGHL17_24 QVVRG--LLLPNS-VPRVRLA-----AASPDALAAL-SG-T---G----VAVTVGVPNELL----

KfGHL17_1 SYNQSLALAHTNALA---WVDANV---VL-WI--RQG-VN--ITSLAVGN**E**PFVLGLN--VTFGA

KfGHL17_2 ---VYLSTNPDAARS---WVNTWV---GK-YI-GR---VQ--FKYVVVGDTPLRLVYN--CYYCA

PmGHL17_1 ------------------WVYNNL---IR-RL-PSG--VN--IRSVSVGN**E**PYLTTKR---AFDN

CgGHL17_1 ------------------WLCHSG---RL-GM-PT-------MPS---GT**E**H-------------

NtGHL17_1 ---NGFASDYESARS---YVA-NV---AA-YL-PR---VK--IGSITVGN**E**ALSVNDG--GQYEH

NtGHL17_2 ---GGLAASRDEVTK---WVDNNV---AK-YL-HDGG-LK--ITCVAVGNDPFRAETF--DEYKD

NtGHL17_3 ----QKSTWDVGYLN---WVASQL---QA-HK------KI--IRYLAIGN**E**PFATWNR--AVAMP

PpGHL17_1 ---PLLASSPAAAQS---WVQSNI---AA-HM-PA---TQ--VTALAVGN**E**VFTTSPQ----MSS

PpGHL17_2 ---PLLASSSTAAQA---WVQSNI---AA-YV-PG---TQ--ITALAVGN**E**VFTTSPQ----MAS

PpGHL17_3 ---IHMATDMTFTQN---WVIYNL---QP-FV-PA---TT--ITTIAVGN**E**ILTSDTA----DTD

PpGHL17_4 ---PALAKDASAADA---WVAANV---VP-YY-PA---TN--IVYIMVGN**E**LFADQTL--AATWL

PpGHL17_5 ---IGFAQSEKAART---WVHNHI---RK-RVLRG---AK--ITYIVVGN**E**ILSGIPE----IWP

PpGHL17_6 ---GQIGTNPAAAEA---WIAQNV---DT-YY-PA---TN--IVTILVGN**E**VFSDASL----PWT

PpGHL17_7 ---VAIGQDNATAYK---WVNDHI---VP-YA-SK---CN--ITAIAVGN**E**VLSYESS----QAV

PpGHL17_8 ---PAIAASQGSADD---WVNKYV---AP-YI-RL---TN--INAIAVGN**E**VITSRPD----LSS

PpGHL17_9 ---ATVASSQQEADR---WVRTHV---KP-FV------SF--IDRIAVGN**E**WLHGHKR----DVS

PpGHL17_10 ---ATLAGDPKAAED---WVKANV---TS-YN-FKGG-AN--IRWVAVGN**E**PFLTAYE--GMYLN

PpGHL17_11 ---GTFASSPEAALN---WINTN-------------------------GRDVSI-----------

PpGHL17_12 ---YDLANNPNAATE---WVKQNV---TR-FN-FKGG-VD--IKWVAVGN**E**PFLTAYN--GSYLN

PpGHL17_13 ---FKLATLKGAADA---WVKQNV---TR-FN-FKGG-VN--IRWVAVGN**E**PFLTAYE--GQYLN

PpGHL17_14 ---AAMAAGTDAAAA---WVKQNV---TA-HL-GSGG-VN--IKYVAVGN**E**PFLNGYN--GKFTD

PpGHL17_15 ---ASMAASPDAAAA---WVKANV---TS-HL-GTGG-VN--IKYVAVGN**E**PFLNGYN--GRYID

PpGHL17_16 ---ATIAASIDAAAA---WVKANV---TS-HL-GNNG-VN--IKYAAVGN**E**PFLTGYN--GKYVD

PpGHL17_17 ---AGIADSQVMANS---WVEKNI---RP-YP-Q----TK--IGSLGVGN**E**FLSDGRN----DAS

PpGHL17_18 ---PSVAQSQWAADQ---WVKKNV---LS-YY-PA---TN--IVSIVVGN**E**LFSYPSM--KSTWD

CaGHL17_1 ---AHFEAEKEALQN---------------YL-PKISVST--IKIFLVGS**E**ALYREDL----TAS

AcGHL17_1 ---QSFNQDFSALKA---SVHGNE---DV-------------VDAITVGS**E**VLYRKGL----TAD

AfgHL17_1 ---KSFTDDFNALKE---AVPGNE------EV----------IDAITVGS**E**VLYRKSL----TPQ

AnGHL17_1 ---QSFDQDFNALKQ---IVPGNE---DV-------------VSAITVGS**E**VLYRGSL----SAQ

CglGHL17_1 ---AHFQAEKNALSS---FLPNLK---SS----------T--IAGFLVGS**E**ALYRDDL----TAS

DhGHL17_1 ---AHFEEEKQALKD---YLPNIS---VD----------T--VKVFTVGS**E**ALYREDL----TAD

AgGHL17_1 ---AHFSMEKDALKTYLPWIKSDT------------------VRGFLVGS**E**ALYRDDM----PAE

FgGHL17_1 ---AHFGRDKAALLK---AIKQHG---TG-W-----------IAAISVGS**E**DLYRKDI----SPQ

KlGHL17_1 ---AHFEEEKQALTQYLPWIKTST------------------IEAFLVGS**E**ALYREDM----TAS

ScGHL17_1 ---SHYAAEKAALQT---------------YL-PKIKEST--VAGFLVGS**E**ALYRNDL----TAS

SsGHL17_1 ---AHFDAEKQALKD---YLPSIS---AS----------T--VKSFLVGS**E**ALYRGDL----TPQ

SpoGHL17_1 ---AHYDLEKQALMQ---------------YL-PQYGVDH--VRAITVGS**E**VLYRNDL----PAD

YlGHL17_1 ---NHFASEQFALKSYLPWLSKST------------------VPYITVGS**E**ALYRKDM----TPQ

AT3G57270.1 ---QSVASSQSQANA---WVQTYV---MN-YA-NG---VR--FRYISVGN**E**VKISDSY-----AQ

AT3G57260.1 ---ERLASSQTEADK---WVQENV---QS-YR-DG---VR--FRYINVGN**E**VKPSV-------GG

AT3G57240.1 ---QRLASSQAEADT---WVRNNV---RN-YA-NV----T--FRYISVGN**E**VQPSDQA-----AS

AT4G16260.1 ---RSLT-NPSSARS---WLQNNV---LN-YY-PA---VS--FKYIAVGN**E**VSPSNGG------D

AT5G56590.1 ---NAFSQSQSNVDT---WLKNSV---LP-YY-PT---TK--ITYITVGA**E**STDDPHI---NASS

AT1G77790.1 ---QSLAHDPAAATN---FVSTWI---TP-YQ--N-D-VA--IKWITIGN**E**VFPGE------IAQ

AT1G77780.1 ---KTLAEEYQFALN---WDKTFI---AP-YK--N---VA--FNWITVGN**E**VIEGE------IGR

AT4G26830.1 ---SSAASHQSFADN---WIKTHI---MP-YF-PA---TE--IEAIAVGN**E**VFVDPTI-----TP

AT4G29360 ---LAFAQFQSNVDT---WLSNNI---LP-YY-PS---TK--ITSISVGL**E**VTEAPDN----ATG

AT3G07320 ---VNISKSASLSDD---WIRSNI---LP-FY-PT---TK--IRYLLVGN**E**ILSLPDS---ELKS

AT2G16230 ---PSIASDLNIASQ---WINSNV---LP-FY-PA---SN--IILINVGN**E**VLLSNDL---NLVN

AT5G20340 ---AALSASEEAVKG---WFATNI---EP-YL-SD---IN--IAFITVGN**E**VIP------GPIGP

AT2G05790 ---FSAAKRTSFAVS---WVKRNV---AA-YH-PS---TQ--IESIAVGN**E**VFVDTHN----TTS

AT5G55180.1 ---ASAAADQSYTDT---WVQDNI---KK-YI--PAT--D--IEAIAVGN**E**VFVDPRN----TTT

AT5G20330 ---AALAASEEAVKG---WFAANI---ES-YL-AD---VN--ITFITVGN**E**VIP------GPIGP

AT2G01630 ---LGISQSNATAAN---WVTRNV---AA-YY-PA---TN--ITTIAVGS**E**VLTSLTN----AAS

AT4G34480 ---PSLASDPNAATQ---WINSNV---LP-FY--PAS--K--IMLITVGN**E**ILMSNDP---NLVN

AT1G66250 ---LGIGQSNSTAAN---WVKRNV---IA-HY-PA---TM--ITAVSVGS**E**VLTSLSN----AAP

AT5G20390 ---EALSVSEEAVNT---WFVTNI---EP-YL-AD---VN--ITFIAVGN**E**VIP------GEIGS

AT3G61810 ---PSLATNLSEAES---IIRNFI---LP-YH-KN---TI--ITAISVGN**E**VSLLPQF-----SN

AT1G32860 ----SKMKDPSKALT---WIKQNV---TP-FL-PA---TN--ITCITIGN**E**ILALNDS---SLTT

AT2G27500 ---QNMSTDPTKAQD---WLQQRL---EP-HI-SK---TR--ITSIVVGN**E**IFKTNDH---VLIQ

AT1G33220 ---GALSANEEAVKG---WFATNI---EP-YL-AD---VN--IAFITVGN**E**VIP------GPIGP

AT3G15800 ---KDISVGEDRAMN---WIKENV---EP-FI--RGG-TK--ISGIAVGN**E**ILGGTDI---GLWE

AT3G23770 ---ISIGADQAAADN---WVATNV---LP-FH-PQ---TR--IRFVLVGN**E**VLSYSSDQDKQIWA

AT5G42720 ---PGLASDPSFARS---WVETNV---VP-YY-PA---SK--IVLIAVGN**E**ITSFGDN---SLMS

AT2G26600 ---KEMSSNADHAFS---WVKENI---QS-FL--P-K-TR--IRGIAIGN**E**VLGGGDS---ELAG

AT4G14080.1 ---TALSSNQTIADE---WVRTNI---LP-YY-PQ---TQ--IRFVLVGN**E**ILSYNSG---NVSV

AT3G46570 ---ISLS-KLSNARS---WLSDNL---LP-FL-LT---TS--IRYIAVGN**E**VVATSDK---TLIT

AT4G18340 ----PLLQDPQQATQ---WVDSHI---KP-YV-PA---TR--ITGIMVGN**E**LFTDDDS---SLIG

AT1G30080 ---PSLV-DPQQALQ---WVTTRI---KP-YF-PA---TK--IGGIAVGN**E**LYTDDDS---SLIG

AT5G42100 ----AQMSDPIKAQG---WVKENV---QA-YL-PN---TK--IVAIVVGN**E**VLTSNQS---ALTA

AT5G58090 ---ATLASSLKAAEK---WVAKNV---ST-HI-STDN-VN--IRYVAVGN**E**PFLSTYN--GSYLS

AT1G11820 ---LAIGSSNSTAAS---WIGRNV---VA-YY-PE---TL--ITAISVGD**E**VLTTVPS----SAP

AT3G24330 ---KEMAQDTSVAAS---WVEENV---TA-YS-YNGG-VN--IKYIAVGN**E**PFLQTYN--GTYVE

AT4G31140.1 ---APLAGSVAAAER---WVSQNV---SA-HV-SSNG-VD--IRYVAVGN**E**PFLKAFN--GTFEG

AT4G17180 ---STFNSDL--------FVQQNL---SR-FI-GKNG-AD--IRYVAVGN**E**PFLTGYG--GQFQN

AT3G13560 ---LKIGRFPSAAAA---WVNKNV---AA-YI-PS---TN--ITAIAVGS**E**VLTTIPH----VAP

AT5G24318 ---PHLT-NLSSAKQ---WISDHI---QP-HF-PS---TN--IIRILVGN**E**VISTADH---LLIR

AT2G39640 ---PAMV-NVANARQ---WVAANV---LP-FQ-QQ---IK--FKYVCVGN**E**ILASNDN---NLIS

AT2G19440 ----KVMGSYDRAKD---WVHKNV---TR-YN-FNGG-VN--ITFVAVGN**E**PFLKSYN--GSFIN

AT3G04010 ---LAMG-NYQRAKD---WVQRNV---SR-FN-FNNG-VK--IKYVAVGN**E**PFLTAYN--GSFIN

AT5G18220 ---KAMG-SYNRAKD---WVRRNI---TR-FN-DD---VK--IKYVAVGN**E**PFLTAYN--GSFIN

AT1G64760 ----KVMTSYDRAKD---WVRKNV---TR-YN-FDGG-VN--ITFVAVGN**E**PFLKSYN--GSFIN

AT5G58480 ---KSLNASVKVAES---WVHDNV---TR-YF-NGGNRVR--IEYVAVGE**E**PFLQSYG--NQYKP

AT3G55430 ---PALA-NGRQARR---WVSVNI---LP-FH-PQ---TK--IKYISVGN**E**ILLTGDN---NMIN

AT5G20870 ---ATMASTVTNAEL---WVQQNV---SQ-YI-SRYG-TD--IRYVAVGN**E**PFLKTYN--NRFVR

AT3G55780 ---PLLASNRSLAMR---WVYRHV---LP-FY-PR---TK--ISIISVGNDVISYSPD----VSP

AT5G64790 ---ESLADDYDNAKD---WVKENV---TQ-YI-RKGG-VD--IKYVAVGN**E**PFLSAYN--GSFLK

AT5G20560 ---EALAANKDAVKS---WFSTNI---DP-YI-AD---VN--ITFITVGNQAIPGDKH-----GP

OsGHL17_1 ----SAMVDPAAAQA---WVQQHV---RP-YL-PS---AR--ITCITVGN**E**VFKGNDT---ALKA

OsGHL17_2 ---QSMAGNPGAARQ---WVTQHV---QP-FL-PA---TR--ITCITVGN**E**VFSGNDT---GMMA

OsGHL17_3 ---PAMA-SPAAAES---WLQLHV---VP-HLRAG---AR--ITCITVGN**E**VFKGNDT---ALQA

OsGHL17_4 ---KDIAASPAKAMD---WLNENV---QP-YY-PS---TR--IVGITVGN**E**VLGGADA---GLAE

OsGHL17_5 ---KDFAANESRSID---WLNENV---QP-YL-PQ---TR--IVGITVGN**E**VLGGQDT---SLAE

OsGHL17_6 ---LPFAQYQSNVDT---WLKNSI---LP-YY-PA---TM--ITYITVGA**E**VTESPVN----VSA

OsGHL17_7 ---PNFASSPAAAAA---WVAAHL---PSTSS-PA-------ISAVSLGN**E**VLFADTS----LAS

OsGHL17_8 ---LAIGNSNATAAN---WVARNV---AA-HY-PS---VN--ITAIAVGS**E**VLSTLPN----AAP

OsGHL17_9 ---LRVGQSRSTAAD---WINKNV---AA-YI-PA---TN--ITHIAVGN**E**VLTTEPN----AAL

OsGHL17_10 ---LALGSSPATASA---WVARRV---LP-YA-GANSSTPGLIAAIAVGD**E**VPTALPS----ALP

OsGHL17_11 ---AAVG-DYGRARE---WVKENV---TR-YS-FDGG-VD--IRYVAVGN**E**PFLKAYN--GQFDR

OsGHL17_12 ----ETMNSYGNAQD---WVKENV---TS-YG-DK---LK--IKYVAVGN**E**PFLKAYN--GSFMK

OsGHL17_13 ---ATMASDYGNAKD---WVKKNV---KR-FD-FDGG-VT--IKYVAVGN**E**PFLKAYN--GSFIN

OsGHL17_14 ---APVSSSVAAAEQ---WVLHNV---SS-YI-SKLG-VD--IRAVAVGN**E**PFLKSYK--GKFEA

OsGHL17_15 ----DLLTDYDAARD---WVHENV---SR-YS-FDGG-VN--IKYVAVGN**E**PFLSSLN--GTFLN

OsGHL17_16 ---ARLAGDPRRAYR---WAEQNV---SA-YLEAG---VD--VRYVAVGN**E**PFLKSYN--GSLIN

OsGHL17_17 ---AAVAGSPAAADA---WVAQNV---SR-YV-GRGG-VD--IRYIAVGN**E**PFLTSYQ--GQFQS

OsGHL17_18 ---QGIAASPAAADA---WVARNV---SR-YV-GPGG-AD--IRYIAVGN**E**PFLTSYQ--GQFQS

OsGHL17_19 ---ADLAAGAKAADD---WVATNV---SN-HV-NNG--VD--IRYVAVGN**E**PFLETFN--GTYLN

OsGHL17_20 ---AAMASSMAAANK---WVDQNV---SN-YL-NDGV--K--IRYVAVGN**E**PFLETYN--GSFLQ

OsGHL17_21 ----DSITDYDTAKE---WVRRNV---SR-YN-FDGG-VT--IKYVAVGN**E**PFLAAYN--GTFDK

OsGHL17_22 ---PSLAADPAAASR---WLAANV---LP-FV-PAST-----ISVVAVGN**E**VLESGDA---SLAA

OsGHL17_23 ---PNLASSPAAASA---WAAANIPTTVP-------------VSAISVGN**E**LLNSGDP---TLAP

OsGHL17_24 ---RPLATSRKAAAA---WVHDNV---TR-YA-SG---VR--FEYVAVGD**E**SFLLNHG--QQNQS

KfGHL17_1 YVMPAVRSVYFALQQRGLN-DTVKLTVPMDAGSTFG-N--TYP-PSVSEFQEP-MKPILLDMLQF

KfGHL17_2 ALPLTVASIQAAILELGLT---TQVSVPLAANDVLDFAAGAWP-PNTAAFNPV-IGPTLLLILKT

PmGHL17_1 YLVLACHRLYAALKRADLH-ERIKLTVSFN-SEILA-D--SFP-PSSGRFTSA-WVDEITNITAL

CgGHL17_1 YNALVDYQID----------TLVKVTVPFT-TDILS----ELSPPSAGAFNDS-ALSFMSPILNL

NtGHL17_1 TLVPAMRNLYTALKSVGLE-TVIKVTSPLS-TGILG-A--SYP-PSQGQFAES-VKSAVVEMLKF

NtGHL17_2 VVSSALKNVVDALKLRNLD-KTVRVTVPVN-ADVAQ----GTDPPSNGSFKDE---VRMREICDV

NtGHL17_3 HLVASFHRVQKMLIDSGLD-KTIKLIIPYS-AEVLT-N--TYP-VANTVFHDG-IMQVMRETLPH

PpGHL17_1 QLVPAMMNIHTALVNLKL--DTIKVGTPHN-LQVLQ-K--SFP-PSSGTFRAN-ISNELKSLLAF

PpGHL17_2 QLVPAMVNIHTALVNLKL--DYIKVSTPHN-LQVLQ-K--SFP-PSSGAFRAN-ITNEVKSLLAF

PpGHL17_3 NLVPAMVNLHSALVTAGL--GDIKVSTPHA-FSVLN-V--SFP-PSASVFRPSFAASVMKPLLDF

PpGHL17_4 QVVPAIQNIHDSLQNHSL--SAIRVSTAVE-YSILA-V--SFP-PSKGSFRPDVAASVMTPLLKY

PpGHL17_5 ALVPAMWQIHSGLVYYGLD-HLIKVSTPHS-MGVMG-A--SYP-PSAGVFAENIRTSIMEPMLRF

PpGHL17_6 SLVPAMQNLYNSLSTRGWS-DKIKVSTAVA-ADVLA-S--SYP-PSVGTFRADIAVPVILPLLRF

PpGHL17_7 MLLPAMKLIHTALVSYSLD-SMMKVTTPMS-ADLLV-S--KFP-PSIGAFSANLTKTTLVPMLDF

PpGHL17_8 SLVPAMQNIHNSLVRLGYD-ASIKVSSPHG-IGLLD-V--SYP-PSAGHFFDS-LTAVVHPMLAF

PpGHL17_9 PLVLAMQNIHRSLVKLSL--SKIKVTTPHA-FDAIG-----FP-PSKGRFP---YPADMKRILNL

PpGHL17_10 TTLPALRNIVNALAKAGQA-NTVRTIIPFN-FDILN----GAVKPSETRFKVE-YLDQIRPMLQI

PpGHL17_11 -LLSAMQNTHASLVALSLN---IKVTTPHA-SDATG-----FP-PSEGKFPKP---DAMKRILQF

PpGHL17_12 TTLPAFQNMQAALDAAGH--TGVRAIIPFN-ADVLT----NVK-PSATTFKPE-YIAQIGPMLEI

PpGHL17_13 TTLPALKNVVDALAKAGHA-NTVRAIIPFN-ADILD----GAPLPSATRFKAE-YLDQILPMLQI

PpGHL17_14 VTYPALKNVQAALAAAGLA-ETVKAVVPCN-ADILS----NNPLPSQQTFRSD-LAPIMLNIAQA

PpGHL17_15 ATLPALKNVQAALAAAGLV-DTVKAVVPCN-ADILS----DNPYPSQQTFRAD-LAPVMLGIAAA

PpGHL17_16 VTLPALKNVQAALAAAGLA-ETVKAVVPCN-ADILS----DNSYPSQQTFRAD-LAPVMLDIVTA

PpGHL17_17 KLVPAMNNIQQALESAGL--NHIKVSTPLA-FQLSV----SYP-PSAGQFADK-DLSVVSGILDF

PpGHL17_18 KLIPAINNLHTSLAKNKLT-DHIKLSTAVA-LDVLA-S--SYP-PSGGAFKEELVGPYLKPLLKY

CaGHL17_1 ELASKINDIKGLVKGIKGKNGKSYSSVPVGTVDSWD------------VLVDGASKPAI------

AcGHL17_1 NLLKRIQQVQNEFPQVTVG-----------FVDSWN------------KIADGTADPIIKGGVNY

AfgHL17_1 ALLARIQQVQKEFPKITVG----------M-VDSWN------------KFADGTADPIIQGGVTY

AnGHL17_1 ALLSKIQQVQNQFPSVTVG----------T-VDSWN------------KFADGTADPIIQGGVTY

CglGHL17_1 QLADKINDIRNYVSNIKDSQGNSYSGKQVGTVDSWN------------VLVAGYNAPVITAS-DF

DhGHL17_1 ELAEKINDIKDTLKDIKDKNGKSYSSVQVGTVDSWN------------VLVDGGSKPAI------

AgGHL17_1 DLAKRINEVRNFVKDIKDINGNSFSGKPVGTVDSWN------------VIVDGRSRPVI------

FgGHL17_1 KLAQQIYDVRGMVHQYN---KALKVGH----TDTWT------------AWVDG-TNDVVTKACDI

KlGHL17_1 ELADAISDVKDYIKDIKDSDGNSYSGKQVGTVDSWN------------VLVDG-GSATAIQAADF

ScGHL17_1 QLSDKINDVRSVVADISDSDGKSYSGKQVGTVDSWN------------VLVAG-YNSAVIEASDF

SsGHL17_1 ELASAISDIKDIVADIKDKDGNSFSSVPVGTVDSWN------------VLVDYYSQPAIKAA-DV

SpoGHL17_1 VLAERIYDVRGLVQ------QKLGFDVPVGTADSWN------------LWAGG-SGDVVITASDF

YlGHL17_1 QLADKINDIKNQLKGIKDK-NGQTFDVPVGTVDSWNVIVDGYSSPAV------------------

AT3G57270.1 FLVPAMENIDRAVLAAGLG-GRIKVSTSVD-MGVLR-E--SYP-PSKGSFRGD-VMVVMEPIIRF

AT3G57260.1 FLLQAMQNIENAVSGAGLE---VKVSTAIA-TDTTT-D--TSP-PSQGRFRDE-YKSFLEPVIGF

AT3G57240.1 FVLPAMQNIERAVSSLG-----IKVSTAID-TRGIS----GFP-PSSGTFTPE-FRSFIAPVISF

AT4G16260.1 VVLPAMRNVYDALRGANLQ-DRIKVSTAID-MTLIG-N--SFP-PSSGEFRGD-VRWYIDPVIGF

AT5G56590.1 FVVPAMQNVLTALRKVGLS-RRIKVSTTLS-LGILS-R--SFP-PSAGAFNSS-YAYFLRPMLEF

AT1G77790.1 FVAAAIKNVNVALTNSGV--TGISVTTVLA-MTALT-N--TYP-PSAATFLPD-LTEIMTEITSI

AT1G77780.1 YVPQAMKNIKAALTEIGN--SKIHVTTVIS-TAALA-N--SYP-PSAGVFKPA-ITELITEIVSI

AT4G26830.1 YLVNAMKNIHTSLVKYKLD-KAIKISSPIA-LSALA-N--SYP-PSSGSFKPELIEPVVKPMLAL

AT4G29360 LVLPAMRNIHTALKKSGLD-KKIKISSSHS-LAILS-R--SFP-PSSASFSKK-HSAFLKPMLEF

AT3G07320 SLVPAMRKIQRSLKSLGV--KKVKVGTTLA-TDVLQ-S--SFP-PSSGEFREDISGLIMKPMLQF

AT2G16230 QLLPAMQNVQKALEAVSLG-GKIKVSTVHA-MTVLG-N--SEP-PSAGSFAPS-YQAGLKGILQF

AT5G20340 QVLPVMQSLTNLVKSRNLP---ISISTVVAMWNLEQ----SYP-PSAGMFTSQ-AREQLVPVLKL

AT2G05790 FLIPAMRNIHKALMSFNLH-SDIKISSPLA-LSALQ-N--SYP-SSSGSFRPELIDSVIKPMLDF

AT5G55180.1 YLVPAMKNVQSSLVKFNLD-KSIKISSPIA-LSALA-S--SYP-PSAGSFKPELIEPVIKPMLDL

AT5G20330 QVLPVMQSLTNLVKSRNLP---ISISTVVAMSNLEQ----SYP-PSAGMFTSQ-AREQLVPVLKL

AT2G01630 VLVSALKYIQAALVTANLD-RQIKVSTPHSSTIILD----SFP-PSQAFFNKT-WDPVIVPLLKF

AT4G34480 QLLPAMQNVQKALEAVSLG-GKIKVSTVNS-MTVLG-S--SDP-PSSGSFAAG-YQTGLKGILQF

AT1G66250 VLVSAIKNVHAALLSANLD-KLIKVSTPLSTSLILD----PFP-PSQAFFNRS-LNAVIVPLLSF

AT5G20390 YVLPVMKSLTNIVKSRSLP---ILISTTVAMTNLGQ----SYP-PSAGDFMPQ-AREQLTPVLKF

AT3G61810 HLVSAMVNVHKAIKRYRLH-KKIKVSTTHS-LAILS-R--RFP-PSTAVFHQSIGDSVLEPLIRF

AT1G32860 NLLPAMQGVHSALITAGLS-DQISVTTAHS-LSILK-S--SFP-PSAGEFQPD-LLDSLTPILEF

AT2G27500 SLLPAMKSVYAALTNLGLE-KQVTVTSAHS-LDILS-T--SYP-PSSGSFKEE-FIQYLQPLLDF

AT1G33220 QVLPVMQSLTILVKSMNLP---ISISTVVAMSNLEQ----SYP-PSAGEFTSQ-AREQLVPVLKL

AT3G15800 ALLPAAKNVYSALRRLGLH-NVVEVSSPHS-EAVFA-N--SYP-PSSCTFRDD-VAPFMKPLLAF

AT3G23770 NLVPAMRKVVNSLRARGI--HNIKVGTPLA-MDALR-S--SFP-PSSGTFREDIAVPVMLPLLKF

AT5G42720 QLLPAMKNVQTALEAASLGGGKIKVSTVHI-MSVLA-G--SDP-PSTAVFKPE-HADILKGLLEF

AT2G26600 ALLGAAKNVYNALKKMNLE-DTVQITTAHS-QAVFS-D--SYP-PSSCVFKEN-VVQFMKPLLEF

AT4G14080.1 NLVPAMRKIVNSLRLHGI--HNIKVGTPLA-MDSLR-S--SFP-PSNGTFREEITGPVMLPLLKF

AT3G46570 HLLPAMETLTLALHLANV--SRILVSTPHS-LGILSGS--SEP-PSSGKFRKGYDKAIFSPILDF

AT4G18340 YMMPAIINIHKALVQLGLD-RYIQVSSPSS-LAVLG-E--SYP-PSAGSFKPE-VSSVMQQLLDF

AT1G30080 YLMPAMMSIHGALVQTGLD-KYIQVSTPNS-LSVLQ-E--SYP-PSAGCFRPE-VAGVMTQLLGF

AT5G42100 ALFPAMQSIHGALVDCGLN-KQIFVTTAHS-LAILD---VSYP-PSATSFRRD-LLGSLTPILDF

AT5G58090 TTFPALRNIQIAIIKAGLQ-NQVKVTCPLN-ADVYD-SSTTF--PSGGDFRAN-IRDLMITIVKF

AT1G11820 LLLPAIESLYNALVASNLH-TQIKVSTPHAASIMLD----TFP-PSQAYFNQT-WHSIMVPLLQF

AT3G24330 FTLPALINIQRALEEADL--KNVKVTVPFN-ADIYF-SPEANPVPSAGDFRPE-LRDATIEIINF

AT4G31140.1 ITLPALQNIQSAIIKAGLA-TQVKVTVPLN-ADVYQ-S--ASNLPSDGDFRPE-IRDLMLNIVKF

AT4G17180 YVVPTMVNLQQSLVRANLA-SYVKLVVPCN-ADAYQ----SNV-PSQGMFRPE-LTQIMTQLVSF

AT3G13560 ILASALNNIHKALVASNLN-FKVKVSSPMS-MDIMP-K--PFP-PSTSTFSPS-WNTTVYQLLQF

AT5G24318 TLIPAMQSLHTALVSASLH-RRIQISTPHS-LGTLT-N--STP-PSSAKFRRGYDAQVLKPLLSF

AT2G39640 NLVPAMQSLNEALKASNL--TYIKVTTPHAFTISYN----RNT-PSESRFTND-QKDIFTKILEF

AT2G19440 LTFPALQNIQNALNEAGLG-SSVKATVPLN-ADVYD-SPSSNPVPSAGRFRPD-IIGQMTQIVDF

AT3G04010 LTYPALFNIQTALNEAGVG-DFTKATVPLN-ADVYN-SPPDNQVPSAGRFRSD-IIQEMTQIVNF

AT5G18220 LTYPALFNIQKALNEAGVG-DFIKATVPLN-ADVYN-SPLENPVPSAGSFRQD-IFEEMKLIVNF

AT1G64760 LTFPALANIQNALNEAGLG-NSVKATVPLN-ADVYD-SPASNPVPSAGRFRPD-IIGQMTQIVDF

AT5G58480 FVIGAAMNIQNALVKANLA-NEVKVVVPSSFDSFLS----ESGRPSSGHFRAD-LNKTMIELLSF

AT3G55430 NLLPAMRNLNNALVRAGV--RDVKVTTAHS-LNIIA-YDLTGA-PSSGRFRPGWDKGILAPILAY

AT5G20870 STYPALQNVQAALVKAGLG-RQVKVTVPLN-ADVYE-S--SDGLPSSGDFRSD-IKTLMISIVRF

AT3G55780 FLLRAMQNVHLSLVDLRI--YKISVSTTFSFFNIVP-T--AFP-PSSAQFQQPNGEVIIRPILQF

AT5G64790 TTFPALKNIHKALKEAGHT-NIMKATIPQN-AEVYQ-S--ANDKPSEGDFRKD-VKQTMLDIVNF

AT5G20560 HVLPVIQSLTDLVKSRNLQ---ISISTTVTTTSLAR----LKP-PSAGVLTPQ-ARQQLVPVLRL

OsGHL17_1 NLLPAMQSVYNAVVALGLQ-GQVNVTTAHS-LDIMG-S--SYP-PSAGAFRPD-AVPYIQPLLNF

OsGHL17_2 SLLPAMKAIYAAVGELGLG-GQVTVSSAHS-VNVLA-T--SFP-PSSGAFRED-LAQYIQPLLDF

OsGHL17_3 SLLPALRSVHQALGALGLQ-GRVNVTTAHS-LDIMG---VSYP-PSAGAFHPS-AAPHLQPFLAF

OsGHL17_4 ALIGAVVNIHDALKMLGLA-TKIELTTPHSEAVFAN----SYP-PSACVFRDD-LMVYLKPLLDF

OsGHL17_5 PLVQAVKNVYNGLKKFHLQ-DKIELFTPHS-EAVFA-T--SYP-PSACVFKED-VMVYMKPLLDF

OsGHL17_6 LVVPAMRNVHTALKKAGLH-KKITISSTHS-LGILS-R--SFP-PSAGAFNSS-YAYFLKPMLEF

OsGHL17_7 QLVPALQNIHAALPPN----SSVKVSTVHA-MDVLA-S--SDP-PSSGAFKPE-LAAALDPLLAF

OsGHL17_8 LLMPAIRYLQNALVAAALD-RYIKISTPHSSSIILD----SFP-PSQAFFNRS-LDPVLVPLLKF

OsGHL17_9 VLVPALQFLQSALLAANLN-TQVKISSPHS-TDMIS-K--PFP-PSTATFNST-WSSIMLQYLQF

OsGHL17_10 VLLPAIQSLAAALAAANL--SSIPVSTPLPFSVVLD----PFP-PSQAFFNQSLAKSFILPLLSH

OsGHL17_11 ATVPALRNIQRALDEAGYG-KRIKATVPVN-ADVYD-SPASNPVPSAGRFRDD-VAGTMADMVRF

OsGHL17_12 TTFPALKNIQKALNEAGVG-DKVKATVPLN-ADVYV-S--PDNKPSSGAFRPD-IQGLMTDMVKF

OsGHL17_13 ITLPALQNVQNALNDAGIG-DRIKATVPLN-ADVYE----STV-PSAGRFRPE-IAGLMTDIVKF

OsGHL17_14 ATLPAVQNVQAALVKAGLA-RQVRVTVPLN-ADVYE-S--LDGRPSAGDFRPD-IAGLMVGLVRF

OsGHL17_15 VTFPALQNIQRALYDAGHG-DTIKATVPLN-ADVYN-SPENMQVPSAGRFRPD-IAGLMTEIVQF

OsGHL17_16 VTFPALKNMQRALDKLGLG-DHVKAVVPLN-ADVYN-SPENKPVPSAGSFRKD-INALMVDIVNF

OsGHL17_17 YVIPAMTNIQQSLVKANLA-SYVKLVVPCN-ADAYQ----SASLPSQGVFRTE-LTQIMTQLAAF

OsGHL17_18 YVLPAMTNIQQSLVKANLA-RYIKLVVPCN-ADAYQ----SASVPSQGVFRPD-LIQIITQLAAF

OsGHL17_19 TTFPAMQSVQAALKKAGLA-DKVKVTVPLN-ADVYQ-S--PTGKPSDGDFRAD-IHGLMLTIVQF

OsGHL17_20 TTFPAIRNIQSALVKAGLG-SQVRVTCPLN-ADVYQ-S--STSKPSDGDFRTD-IHDLMLTIVKF

OsGHL17_21 VTLPALMNIQNALNDAGLG-DSIKATVPLN-ADVYD-SPQDQQVPSAGRFRAD-IADLMTQMVQF

OsGHL17_22 ALLPAMQNLRAAAAAAGDGAARIKFSTVNT-MAVLA-Q--SDP-PSTGAFHPD-ISPQLTQILGF

OsGHL17_23 QLLPAMQNLLAALPAG----STTKISTVHS-MAVLS-S--SDP-PSSGAFHAD-LAGSLDPVLDF

OsGHL17_24 FLVSAAANIQRALVDAKLS-NKMKVVVPCS-SDVYL-N--TSALPSKAYFRPE-VNETIAELLSF

KfGHL17_1 LAQTRSFLTLNLYPFVSLKYD---NTIDIDFAVGR-------PGHGFTDQGYYYSDLLSAQIDAT

KfGHL17_2 AFEANANLLVELDPYAIIQAN---TTIPLNMALGTAQMGD--PGTFQLITGQNYTNIYDIYLDAI

PmGHL17_1 IASTGSFFSINIYPFFSFRSD---PKIPMGYVMFNA-TDK--ETVMDNGTRIAYRNMVDAGYDAV

CgGHL17_1 LQETNSRFWLEVFP--PLIADKIRPYPTLEYLYVNWT-----LGFVDLGSDLFYTNTLMHALDAT

NtGHL17_1 LNETGSHLMVNIYPFFPCNED---GSIPLDYALFR-------GGSGFFDNGLYYTNLLDAMMDAV

NtGHL17_2 LSRANSAFTVNINPFYMAKDN---PTIPLDFVMFQP------------VVAGDYSNQLDKMYDGV

NtGHL17_3 IASSGSAFSINLYTYFAFANG----GVPLDVALGRS------------------GSLLKNMIDGV

PpGHL17_1 LSTTNNPIMINFYPYFAYRDD--PKNVSLNYALFQPD-----TGVTDVNTGLHYDNMLDAQLDAV

PpGHL17_2 LSATSSPIMVNFYPYFAYRDD--PKNVSLNYSLFQPN-----TGVTDVNTGLHYNNMLDAQLDSV

PpGHL17_3 LNQTGSPFMVNIFPFFSYMFN--YNTINLDYALLNPNA----PPVNDPGNGKIYTNLWDAQIDAI

PpGHL17_4 LDSTDSYLFVNVYPYFGWSTN--SQYIPLDYALFTR------NTTFTTDGQYGYANLLDAQLDAM

PpGHL17_5 LKLTGSTLMMNIYPYFPYRDD--PVNISPGYALFLNNA----TGVDDPNTGLHYSNLFDAMLDSS

PpGHL17_6 LSSTHSYFYINLYPFLSYTTS--GGDISLNYALFAS------GSNSVVDGSLTYTNLLDAQLDAV

PpGHL17_7 LSAIGSFYFLNVYPHKEYQHG--QTNISLEFALFQNN-----PGVVDSATGFLYTNAFDSLLDAT

PpGHL17_8 LQETKAPFMLNIYPFYSYQNSIVSGSVPLDYALFQPA-----SPVVDAGNGLVYNSLYDAQIDAV

PpGHL17_9 LQTTKSAFTLNVYPFFAYKVN---ANVNREYAVFNP------NSNHVIDMGRRYTNLFDAQVDTH

PpGHL17_10 FNSTGAPFSVNLYPFISKYQS---PDFPLDYAFFEG------STSAVTDGTFIYKNALDASLDAL

PpGHL17_11 LKEKNSAFMLNVYPFFAYTLN---AAIDRNYAVFNP------NNKPVIDMGRTYTNLFDALVDTH

PpGHL17_12 MNRTGAPFCMNLYPYISLYMD---SGYPVDYAFFSG------TTSPNVDGAITYQNALDASLDGL

PpGHL17_13 FNSTNSPFSVNLYPFISKYQN---PSFPLDYAFFGG------TTSPLIDGSNTYTNALDASVDAL

PpGHL17_14 LDSTKSPFVVNLYPFLSLVLG---TNFPIDYAFFSG------YSTPLVDGTKTYDNVFDASYDGV

PpGHL17_15 LKSTNSPFVVNLYPFLNLVLQ---ANFPIDFAFFSG------FATPIVDGTRVYTNVFDAGFDGV

PpGHL17_16 LQSTNSPFVVNLYPFLNLVLQ---PTFPVDFAFFTG------YNTPLVDGTRIYTNVFDASFDGV

PpGHL17_17 VRRKNSVFMMNIYPFFAYRFD--SVNIDINYALFNP------NAPTINDSGRAYRNLFDAQVDSV

PpGHL17_18 LYNSQSHLYVNVYPYFAWASD--PDHIPLNYALFGA------STPGVVDNGKAYYNLLDSQLDAV

CaGHL17_1 --DAADVVYSNSFSYWQKNSQ-----ANASYSLF------------------------DDVMQAL

AcGHL17_1 ILANG-------FAYWQGQEL---SNATNTY--F------------------------DDMAQAL

AfgHL17_1 FLANG-------FAYWQGQEL---SNATNTY--F------------------------DDMAQAL

AnGHL17_1 LLANG-------FAYWQDVDI---NNAPSTY--SSD----------------------MTQAEDH

CglGHL17_1 V-------MANAFSYWQGQTM-----QNASYSFF------------------------DDIMQAL

DhGHL17_1 --DAADFVFANAFSYWQGQTS-----KNASYSFF------------------------DDIMQAL

AgGHL17_1 --EASDLVMANAFSYWQGQTM-----QNASYSFF------------------------DDIMQAL

FgGHL17_1 AITNG-------FPYWQGVPI---KDALRLKTFQNS-----------------YWNV-KKHVQAV

KlGHL17_1 V-------MANAFSYWQGQTM-----DNASYSFF------------------------DDIMQAL

ScGHL17_1 V-------MANAFSYWQGQTM-----QNASYSFF------------------------DDIMQAL

SsGHL17_1 V-------YANAFSYWQGQTK-----ANASYSFF------------------------DDIMQAL

SpoGHL17_1 IMSND-------FPYWQGQNT-----SNMTNTFISD------------------------TLAAL

YlGHL17_1 --KAADVVFANAFSYWQGQTM-----ANASYSFF------------------------DDIMQAL

AT3G57270.1 LVSKNSPLLLNLYTYFSYAGN--VGQIRLDYALFTA------PSGIVSDPPRSYQNLFDAMLDAM

AT3G57260.1 LASKQSPLLVNLYPYFSYMGD--TANIHLDYALFTA------QSTVDNDPGYSYQNLFDANLDSV

AT3G57240.1 LSSKQSPLLVNNYPYFSYTGN--MRDIRLDYTLFTA------PSTVVNDGQNQYRNLFHAILDTV

AT4G16260.1 LTSTNSALLANIYPYFSYVDN--PRDISLSYALFTS------PSVVVWDGSRGYQNLFDALLDVV

AT5G56590.1 LAENKSPFMIDLYPYYAYRDS--PNNVSLDYVLFESS-----SEVIDPNTGLLYKNMFDAQVDAL

AT1G77790.1 LSETNSPLMTNIYPYFAYASD--PYHISLDYASFKS------NTPVVIDGDLYYNNMFEAMVDGF

AT1G77780.1 LSSTDSPLMVNVYPYFAYASD--PSHVSLEYATFRS------TSPVVTDGKYQYTNIFDATLDAF

AT4G26830.1 LQQTSSYLMVNAYPFFAYAAN--ADKISLDYALFKEN-----AGNIDSGTGLKYNSLFDAQIDAV

AT4G29360 LVENESPFMIDLYPYYAYRDS--TEKVPLEYALFESS-----SQVVDPATGLLYSNMFDAQLDAI

AT3G07320 LNRTKSFLFVDVYPYFAWAQD--PTHVDLDYAIFESTN----VTVTDPVSNLTYHNLFDQMIDAF

AT2G16230 LSDTGSPFAINPYPFFAYQSD--PRPETLAFCLFQPN-----PGRVDSNTGIKYMNMFDAQVDAV

AT5G20340 LSQTNSPILVKIYPYFSYASD--PSSIRLDYATFNT------EAIVVQDGSLGYSNMFDAIFDAF

AT2G05790 LRETGSRLMINVYPFFAYEGN--SDVIPLDYALLREN-----PGMVDSGNGLRYFNLFDAQIDAV

AT5G55180.1 LRKTSSHLMVNAYPFFAYAAN--ADKISLDYALFKEN-----AGNVDSGNGLKYNSLLDAQIDAV

AT5G20330 LSQTSTPILVNIYPYFAYASD--PANIRLDYASFNT------KSIVVQDGSLGYSNMFDAIFDAF

AT2G01630 LQSTGSPLLLNVYPYFDYVQS--NGVIPLDYALFQPLQAN--KEAVDANTLLHYTNVFDAIVDAA

AT4G34480 LSDTGSPFAINPYPFFAYQSD--PRPETLAFCLFEPN-----AGRVDSKTGIKYTNMFDAQVDAV

AT1G66250 LQSTNSYLMVNVYPYIDYMQS--NGVIPLDYALFKPIPPN--KEAVDANTLVRYSNAFDAMVDAT

AT5G20390 LSQTNTPILVNIYPYFAYAAD--PINIQLDYAIFNT------NKVVVQDGPLGYTNMFDVIFDAF

AT3G61810 LQRTNSPLMVNVYPYLAYKQS--FPSIPLDFALFQPMNSPRRRRYIDPYTGVAYTNLFDIMLDSV

AT1G32860 HRKTDSPFLINAYPFFAYKGN--PKEVPLDFVLFQPN-----QGIVDPATGFHYDNMLFAQIDAV

AT2G27500 HSQIESPFLINAYPFFAYKDS--PKEVPLEYVLFQPN-----QGMVDPNTNLHYDNMLFAQVDAL

AT1G33220 LSQTSTPILVNIYPYFPYASD--PTNIPLDYATFNT------KATVVQDGPLGYSNMFDAIFDAF

AT3G15800 FWQIQSPFYINAYPFLAYKSD--PITIDINYALFEHN-----KGILDPKTKLHYDNMFDAMVDAS

AT3G23770 LNGTNSFFFLDVYPYFPWSTD--PVNNHLDFALFESN-----STYTDPQTGLVYTNLLDQMLDSV

AT5G42720 NSETGSPFAVNPYPFFAYQDD--RRPETLAYCLFQAN-----PGRVDPNSNLKYMNMFDAQVDAV

AT2G26600 FQQIGSPFCLNAYPFLAYTYN--PKEIDINYALFKPT-----EGIYDPKTDLHYDNMLDAQIDAA

AT4G14080.1 LNGTNSYFFLNVHPYFRWSRN--PMNTSLDFALFQGH-----STYTDPQTGLVYRNLLDQMLDSV

AT3G46570 HNRTKSPFMVNPYPYFGFGPE------TLNYALFNTND----VVYVDPVTKLNYTNMFDAQLDAV

AT4G18340 LEATKSPFWINAYPYFAYKDN--PQEIPVDYVLFNRN-----IGMTDPNTRLHYDNMMYAQVDAV

AT1G30080 LRNTNSPFWINAYPYFAYKDS--PTKIPLDYVLFNPN-----PGMVDPYTKYRYDNMLYAQVDAV

AT5G42100 HVKTGSPILINAYPFFAYEEN--PKHVSLDFVLFQPN-----QGFTDPGSNFHYDNMLFAQVDAV

AT5G58090 LSENGGPFTVNIYPYISLYTN---PDFPVDYAFFDG------NAQPLNDGGTFYYNMFDANYDTL

AT1G11820 LSKTGSPLMMNLYPYYVYMQN--KGVVPLDNCLFEPLTPS--KEMVDPNTLLHYTNVLDAMVDAA

AT3G24330 LYSHDSPFTVNIYPFLSLYGN---AYFPLDFAFFDG------TNKSLRDGNLVYTNVFDANLDTL

AT4G31140.1 LSDNGAPFTINIYPFISLYND---PNFPVEFAFFDG------TGTPINDNGRIYDNVLDANYDTL

AT4G17180 LNSNGSPFVVNIYPFLSLYGN---SDFPQDYAFFEG------SSHPVPDGPNTYYNAFDGNFDTL

AT3G13560 LKNTGSFFMLNAYPYYGYTTA--NGIFPLDYALFKQLSPV--KQIVDPNTLLHYNSMFDAMVDAA

AT5G24318 LRSTSSPFVVNPYPFFGYSIE------TLDFALFRPN-----PGLFDQHTKLLYTNMLDAQLDSV

AT2G39640 HRQAKSPFMINAYTFFTMDTN------NVNYAIFGPS-----NAITDTNTQQTYTNMFDAVMDAT

AT2G19440 LGNNSAPITINIYPFLSLYGN---DDFPLNYAFFD-------GAKPVDDNGIAYTNVFDANFDTL

AT3G04010 LAQNKAPFTVNIYPFLSLYLS---SDFPFEYAFFD-------GQNTVNDNGVIYTNVFDASFDTL

AT5G18220 LAHNKAPFTVNIYPFLSLYLS---SDFPFDYAFFN-------GQNTVSDNGVIYTNVFDANFDTL

AT1G64760 LGKNNAPITINIYPFLSLYGN---DDFPLNYAFFD-------GAEPINDNGIDYTNVFDANFDTL

AT5G58480 LTKHHSPFFVTISPFLSFHQN---KNISLDFSLFKE------TAKAHKDGRKTYRNSFDLSYDTL

AT3G55430 HRRTKSPFMVNPYPYFGFDPK------NVNFAIFRT--PY--KAVRDPFTRHVYTNMFDALMDST

AT5G20870 LADSVSPITFNIYPFLSLNAD---PNFPREYAFFPNGGGGG-GAKPVVDGSISYTNVFDANFDTL

AT3G55780 LERTNSSFLINLYPYNMYRSS---FSIPIGFALFEE-FPF--NFRDDLTTGVRYRNLFDMMVDAV

AT5G64790 FHENDLPFTVNIYPFLSLYLN---EHFPVEFAFLDG------DGQTMTDKGKNYDNVFDANYDTL

AT5G20560 LSQTSTPIFVNIYPYYFHASD--PKNVPLEYANFNN------DQIVVKDGALKYSNLFDAIFDAF

OsGHL17_1 LSMAGSPFLINCYPYFAYKAD--PGSVPLEYVLFQPN-----AGVTDPNTKLNYDNMLYAQIDSV

OsGHL17_2 HGQTNSPFLINAYPFFAYKAS--PGSVSLPYVLFEPN-----PGVRDPNTNLSYDNMLYAQIDAV

OsGHL17_3 LSAARAPFLINCYPYFAYKDD--PARVPLEYVLFQPN-----AGVVDPRTRLVYDNMLYAQVDAV

OsGHL17_4 FSKTGAPFYVNAYPFLAYMSD--PAHIDVNYALFKPN-----AGIYDAKTRLRYDNMFEAQVDAA

OsGHL17_5 FQQIGSPFYVNAYPFLAYISD--PEHIDINYALFKPN-----PGIVDPNTSLHYDNMFDAQIDAA

OsGHL17_6 LVENQAPFMVDLYPYYAYQNS--PSNVSLNYALFSPQSQD----VIDPNTGLVYTNMFDAQIDSI

OsGHL17_7 LSKTGSPFLINPYPYFAYLSD--PRPETLAFCLFQPN-----AGRPDAGSGLTYTNMFDAQVDAV

OsGHL17_8 LQSTGSPLMLNVYPYYDYMRS--NGVIPLDYALFRPLPPN--KEAVDANTLLHYTNVFDAVVDAA

OsGHL17_9 LNNTASPFMLNAQPYYDYVKG--QGVFPLEYALFRSLNPD--SQISDPNTNLFYTNMFDAMVDAT

OsGHL17_10 LANTSAPLMLNLYPYYSMMQS--NGVIPLDNALFKPLPPS--LEMVDPNTLLHYTNVFDAMLDAV

OsGHL17_11 LNRSGAPLTVNIYPFLSLYGN---DDFPLDYAFFDGGP----PPRPVVDNGINYTNVFDANFDTL

OsGHL17_12 LHEHGSPFVVNIYPFLSLYQS---DDFPFEFAFVDG------GKTIQDKGGISYSNVFDANYDTL

OsGHL17_13 LAKNNAPFTVNIYPFLSLYLD---EHFPINFAFFDG------GSTPVNDGGIMYTNVFDANFDTL

OsGHL17_14 LLDNGGFLTINIYPFLSLQAD---PNFPADYAYF-PSPGSPPSQASVQDGGVLYTNVFDANYDTL

OsGHL17_15 LNQSGAPFTVNIYPFLSLYGN---DNFPLDYAFFDGT-----TSPVVDTNGIQYTNVFDANFDTL

OsGHL17_16 LNMNNAPFVVNIYPFLSLYQN---PNFPLNFSFFDG------GSKPVYDKGVVYTNVFDANFDTL

OsGHL17_17 LSSSGAPFVVNIYPFLSLYQS---SDFPQDYAFFEG------STHPVVDGPNTYYNAFDGNFDTL

OsGHL17_18 LSSSGAPFVVNIYPFLSLYQS---SDFPQDYAFFDG------SSHPVVDGPNVYYNAFDGNFDTL

OsGHL17_19 LADTGAPFVANVYPFISLYKD---PNFPLDYAFFQG------SSAPVVDGGVTYQNTFDANHDTL

OsGHL17_20 LSDTGGAFTVNIYPFISLYSD---SNFPVDYAFFDG------AASPIVDGSATYTNMFDANYDTL

OsGHL17_21 LANNSAPFTVNIYPFISLYLN---DDFPVDFAFFDG------GATPVVDNGISYTNVFDANFDTL

OsGHL17_22 LSKTTAPFMINPYPYFAYQSD--PRPETLAFCLFQPN-----AGRVDAGSKIKYTNMFDAQVDAV

OsGHL17_23 LKQNGAPFMINPYPYFAYASD--TRPETLAFCLFQPN-----PGRVDAGSGLTYTNMFDAQLDAI

OsGHL17_24 LANHSSPFMVELNPFSSFQHK---KNLSLDYYLFQL------MSHPVSDGHIKYDNYFDASIDAL

KfGHL17_1 YIAMNKL---YPQGAQNVSLQLG**E**TGWPSAGGYGATLQNASDYQGAQNVSLQLGETGWPSAGGYG

KfGHL17_2 AYAATAFR--FPT----LNITVASTGWPTDGG-------------------------------GN

PmGHL17_1 VSALTKIG--FPD----VELVVG**E**S----------------------------------------

CgGHL17_1 YAALDKWG--YGN----VSVIIG**E**AGWPTAGL-------------------------------PR

NtGHL17_1 IFAMEKEG--FAN----IPLVVG**E**VGWPTGGA-------------------------------AI

NtGHL17_2 VAALRKIG--FPD----MQVVVG**E**VGWPTDGH-------------------------------EL

NtGHL17_3 RVALDKVG--FPQ----IPILVG**E**TGWPSAEA-------------------------------KG

PpGHL17_1 YSAMERFG--YHN----IPVLIS**E**TGWPSSGD----------------------------PTEIA

PpGHL17_2 YSAMERFG--YHN----IPVLIS**E**TGWPSSGD--------------------------PTE--IA

PpGHL17_3 ISAMASLG--HPS----IPIVVT**E**SGWPSVGD----------------------------VTQVG

PpGHL17_4 AAAMATVG--YPN----VRIAIS**E**TGWPSVGD----------------------------SNELG

PpGHL17_5 IFAMKNLG--YHD----IPVIVT**E**TGWPSIGE----------------------------EWEKA

PpGHL17_6 ISAMEKLG--FGD----VRVAVG**E**TGWPTKAD----------------------------ATQTG

PpGHL17_7 YAALAKLN--HTD----LTIVVS**E**TGWPSQGE----------------------------AYEKG

PpGHL17_8 IAACLKLN---KT----VGVTVT**E**TGWPSDGD---------------------------PSYEPA

PpGHL17_9 RSAMAAIG--YPD----FPLVIG**E**TGWPSAGS-----------------------------NARG

PpGHL17_10 ISALTAEG--YPA----MEILLG**E**IGWPTDGN-------------------------------EF

PpGHL17_11 RSAMATLG--YPD----FPLVIG**E**SGWPSAGS-----------------------------NARG

PpGHL17_12 ASALSKAG--YPS----MPIMLG**E**IGWPTDGA-------------------------------RS

PpGHL17_13 IAALGAAG--YPN----MPVVLG**E**IGWPTDGN-------------------------------EY

PpGHL17_14 VSALVTAG--YPN----MVVSVG**E**IGWPTDGN-------------------------------AY

PpGHL17_15 VGALNNAG--YPN----MAVIVG**E**IGWPTDGA-------------------------------NY

PpGHL17_16 VVALNNAG--YPN----MDVIVG**E**IGWPTDGA-------------------------------NY

PpGHL17_17 YAAMSRLG--YAN----TPLMIT**E**TGWASDGG------------------------------GVG

PpGHL17_18 NAATEKVG--YGQ----VRLALS**E**TGWPSAGD----------------------------ANQLG

CaGHL17_1 QTLQTAKG--STD----IEFWVG**E**TGWPTDGS---------------------------SYGDSV

AcGHL17_1 EHIEKVAP--NPD---KIRFGNG**E**TGWPTTGG--------------------------SNYGPAV

AfgHL17_1 GHIEQVAGSNADK----IRFGNG**E**TGWPTTGG--------------------------TNYGPAV

AnGHL17_1 IKQVAGAN--AGN----IRFGNG**E**TGWPTDGG--------------------------SDYGAAK

CglGHL17_1 QTIQGVKG--STD----ITFWVG**E**TGWPTDGT---------------------------NFEASY

DhGHL17_1 QTIQAAKG---TD----IDFWVG**E**TGWPTEGT---------------------------NFESAD

AgGHL17_1 QTVQTVKG--SSD----ISFYVG**E**TGWPTEGS---------------------------SFETSQ

FgGHL17_1 ----------NSK----ATVWVG**E**TGWPTKGP---------------------------NYQKAA

KlGHL17_1 QTIQTTKG--STD----IQFWVG**E**TGWPTDGT---------------------------NFESAY

ScGHL17_1 QVIQSTKG--STD----ITFWVG**E**TGWPTDGT---------------------------NFESSY

SsGHL17_1 QVIQTTKG--DTN----IEFWVG**E**TGWPTEGT---------------------------NFEDSI

SpoGHL17_1 ERVQSVKG--TNN----VTFWVG**E**TGWPTDGP---------------------------SYGEAD

YlGHL17_1 QTIQTTKG--TTD----IDFWVG**E**TGWPTDGG---------------------------AFGDSQ

AT3G57270.1 YSALEKSG--GAS----LEIVVA**E**TGWPTGGG-------------------------------TD

AT3G57260.1 YAALEKSG--GGS----LEIVVS**E**TGWPTEGA-------------------------------VG

AT3G57240.1 YASLEKAG--GGS----LEIVVS**E**SGWPTAGG-------------------------------AA

AT4G16260.1 YSAVERSG--GGS----LPVVVS**E**SGWPSNGG-------------------------------NA

AT5G56590.1 YYALTALN--FRT----IKIMVT**E**TGWPTKGS---------------------------PKEKAA

AT1G77790.1 NAALEKIN--AAN----VVVMVA**E**TGWPTEGN------------------------------PPH

AT1G77780.1 NVALEKIN--HGS----VKVYVA**E**TGWPTRGN------------------------------DPY

AT4G26830.1 YAALSAVG--FKG----VKVMVT**E**TGWPSVGD----------------------------ENEIG

AT4G29360 YFALTAMS--FKT----VKVMVT**E**SGWPSKGS----------------------------PKETA

AT3G07320 VFAMKRVG--YPD----IRIWVA**E**TGWPNNGD----------------------------YDQIG

AT2G16230 HSALKSIG--FEK----VEVLVA**E**TGWPSTGD----------------------------SNEVG

AT5G20340 VWAMEKEG--VKD----LPMVVS**E**TGWPSAGN------------------------------GNI

AT2G05790 FAAMSALK--YDD----IEIIVT**E**TGWPSKGD----------------------------ENEVG

AT5G55180.1 FAAMSAVG--FND----VKLVVT**E**TGWPSAGD----------------------------ENEIG

AT5G20330 VWAMEKEG--VKN----LPMVVS**E**TGWPSAGN------------------------------GNF

AT2G01630 YFAMSYLN--FTN----IPIVVT**E**SGWPSKGG----------------------------PSEHD

AT4G34480 HSALKSMG--FEK----VEIVVA**E**TGWASRGD----------------------------ANEVG

AT1G66250 YFAMAFLN--FTN----IPVLVT**E**SGWPSKGE----------------------------TNEPD

AT5G20390 VWAMEKEG--VKD----LPMVVT**E**TGWPSAGN------------------------------GNL

AT3G61810 DSAVKSLG--LPE----IPVVVS**E**IGWPTRGD----------------------------PGETA

AT1G32860 YSALAAAG--FKS----LRVEIS**E**TGWPSKGD----------------------------DDEVG

AT2G27500 YSAIKTLG--HTD----IEVRIS**E**TGWPSKGD----------------------------ENEIG

AT1G33220 VWAMEKEG--VKD----LPMVVS**E**TGWPSAGN------------------------------GNL

AT3G15800 YAALEKAG--YTK----VPVIVS**E**TGWASKGD----------------------------ADEPG

AT3G23770 IFAMTKLG--YPN----ISLAIS**E**TGWPNDGD----------------------------IHETG

AT5G42720 YSALNSMG--FKD----VEIMVA**E**TGWPYKGD----------------------------PEEAG

AT2G26600 YMALQDAG--FKK----MEVMIT**E**TGWASKGD----------------------------SDEPA

AT4G14080.1 LFAMTKLG--YPH----MRLAIS**E**TGWPNFGD----------------------------IDETG

AT3G46570 YSAMKRFG--YGD----VDIVVA**E**TGWPSAGE----------------------------PNQTG

AT4G18340 AFAAAKLG--YRN----IEVRVA**E**TGWPSKGD----------------------------VGEIG

AT1G30080 IFAMARLG--FKD----IEVGVS**E**TGWPSKGD----------------------------GDEVG

AT5G42100 YHALDAVGISYKK----VPIVVS**E**TGWPSNGD----------------------------PQEVG

AT5G58090 VHALEKNG--FGN----MPIIIG**E**IGWPTDGD-------------------------------SN

AT1G11820 YVSMKNLN--VSD----VAVLVT**E**SGWPSKGD----------------------------SKEPY

AT3G24330 ICAMERYS--FLG----MKIIVG**E**VGWPTDGD-------------------------------KN

AT4G31140.1 VWSLQKNG--FGN----LTIIVG**E**VGWPTDGD-------------------------------KN

AT4G17180 VAALTKLG--YGQ----MPIVIG**E**IGWPTDGA-------------------------------VG

AT3G13560 YYSMEALN--FSK----IPVVVT**E**TGWPSSGG----------------------------SDEAA

AT5G24318 YSAMDKLG--FSD----VEIVIG**E**IGWPSEGD----------------------------IDQIG

AT2G39640 YSAMKALG--YGD----VDIAVG**E**TGWPTACD------------------------------ASW

AT2G19440 VSALKAVG--HGD----MPIIVG**E**VGWPTEGD-------------------------------KH

AT3G04010 LASLNALN--HGN----MEVIVG**E**VGWPTDGD-------------------------------KN

AT5G18220 LASLKALG--HGD----MTVIVG**E**VGWPTDGD-------------------------------KN

AT1G64760 VSSLKAVG--HGD----MPIIVG**E**VGWPTEGD-------------------------------KH

AT5G58480 VSALFTIG--FSE----VDIVVSKIGWPTDGA-------------------------------EN

AT3G55430 YSAMKALG--YGD----VNIVVG**E**TGWPSACD------------------------------APW

AT5G20870 VSALEKNGFDANK----IEIIVG**E**VGWPTDGD-------------------------------QN

AT3G55780 ISSMAVMG--HEN----LPVIVA**E**TGWPSSGI--------------------------DASE-VD

AT5G64790 VYALKKAG--IHD----MKIIVG**E**VGWPTDGH-------------------------------KY

AT5G20560 LWAMEKEG--VKG----LPLVVS**E**TGWPSAGN------------------------------GGM

OsGHL17_1 YAAMQALG--HTD----VDVKIS**E**TGWPSRGD----------------------------PDEAG

OsGHL17_2 YAAMKAMG--HTD----IGVRIS**E**TGWPSKGD----------------------------EDEAG

OsGHL17_3 YAAIQAMG--HTD----IDVKVS**E**TGWPSRGD----------------------------PDEAG

OsGHL17_4 YFALEAAG--YPE----MEVRVA**E**TGWASAGD----------------------------ATEAG

OsGHL17_5 YAALQAAG--YRD----MEVRVA**E**TGWASSGD----------------------------QTEAG

OsGHL17_6 FFALMALN--FKT----LKIMVT**E**TGWPNKGA----------------------------AKETG

OsGHL17_7 RAALDAKG--YKD----VEIVVA**E**TGWPHSGG----------------------------ADEAG

OsGHL17_8 YFAMAYLN--VTN----VPVMVT**E**TGWPHKGD---------------------------PSNEPD

OsGHL17_9 YNSMQAMN--FTG----IPVMVT**A**SGWPSHGG----------------------------QNEKA

OsGHL17_10 HVAVKNLN--ATGGGGPVPVLVT**E**TGWPSYGD---------------------------RRAEPY

OsGHL17_11 VSALKRIG--FGS----LPIVIG**E**VGWPTDGD-------------------------------KH

OsGHL17_12 VTALKKAG--VPS----LKVVVG**E**VGWPTDGD-------------------------------KN

OsGHL17_13 VAALKAVG--HGD----MPIIVG**E**VGWPTDGD-------------------------------KN

OsGHL17_14 ISALEKHG--LGA----IAVVVG**E**IGWPTDGD-------------------------------KS

OsGHL17_15 VSALVAAG--VGG----LPVVVG**E**VGWPTDGD-------------------------------KH

OsGHL17_16 VWSLRKAG--VPD----MKIIVG**E**VGWPTDGD-------------------------------KH

OsGHL17_17 VAALGKIG--YGQ----LPIAIG**E**VGWPTEGA-------------------------------PS

OsGHL17_18 VSALSKIG--YGQ----LPIAIG**E**VGWPTEGA-------------------------------PS

OsGHL17_19 VAALRRNG--YPN----VSIIVG**E**VGWPTDGD-------------------------------AN

OsGHL17_20 IWALKKNG--FGN----LPVIVG**E**IGWPTDGD-------------------------------MN

OsGHL17_21 VAALKGVG--HGD----MPIVVG**E**VGWPTDGD-------------------------------KH

OsGHL17_22 KSALGRAG--YGD----VEIVVA**E**TGWPTRGD----------------------------AGEAG

OsGHL17_23 RAALDAKG--YSG----VDIVIA**E**TGWPYKGD----------------------------ADEGG

OsGHL17_24 VTSLTKAG--FSN----MDIIVGRVGWPSDGA-------------------------------VN

KfGHL17_1 ATLQNASDYLTYSVSPLA--GGQGTPRYPNRTV-KSYIFALFDEDLK---YTIGIGD-----Y--

KfGHL17_2 ATLANAQTYVNYVIQRAL--SNGGTPYRQSRVT-ELYLNEIIDQDLI--ELTDVPFP-----NGW

PmGHL17_1 ----------------------RGRPLR--------------------------TGP-----TQ-

CgGHL17_1 ANTVSAQEFTQGLVNYCL--SGLGTPARPDIIP-GCFIRSLVDEDLRGNFSTEPNGP-----W--

NtGHL17_1 ATIENAQAFNNYLIGHIM--SGQGTPKRAGWT--QIYIFELLDEDQK----SLGPGP-----V--

NtGHL17_2 ATVQNAQRFNQACIKHLT--SGTGTPSVKNKQI-DAYLFSLFDEDAK----SVEPGP-----F--

NtGHL17_3 ASVDNARDYTRHVNSYAS-----------KSDIQSVFFFEAFDEPGK-------PDP-----PL-

PpGHL17_1 ASATNAQIYNQNLLKYIA--SNKGTPLRPSSSV-DAYIFALFNENMK-------PGP-----GS-

PpGHL17_2 VSATNAQIYNQNLIKYVT--SNKGTPLRPSTSV-DAYIFALFNENMK-------PGP-----GS-

PpGHL17_3 PSVANAQTYNNNLVKLVLADPPKGTPLRPGVAT-PTYIFSLFNENLK-------TGK-----IT-

PpGHL17_4 ASRSNAQTYNQNLVTHILSSPTRGTPMRPGIFV-PTFIFALYNENAK-------PGA-----TS-

PpGHL17_5 AGLENAKTFNNNLLKHVK--SGKGTPARPDTTI-QIFIFALFNEYQK-------PGP-----LS-

PpGHL17_6 ASVQNAAMYNRRLVRKLLSSSTNGTPKRPNVFI-PTFIFALFNENQK-------PGP-----ES-

PpGHL17_7 LSPSNAQTYNANLVKHVL--SKVGSPGRPGVLI-ITYIYELFNEDKR-------QGP-----LS-

PpGHL17_8 ANYWNARMYNQNLVKRSM--NNSGTPMRPGVEF-DAYIVSLYDENLR-------PTP-----PAS

PpGHL17_9 VNIQDAQTYNNNLVKHEL--SSEGTPMRRNVRM-PTYIFALFNENLK-------GGG-----I--

PpGHL17_10 ANPTLAGKYNQQLINHLQ--SKVGTPLRPNTFT-EFYMFGLLDENIK----SILPGP-----F--

PpGHL17_11 VNIQDAQTYNNNLVKHVL--SNKGTPMRPNVRM-PTYIFALFNENLK-------GGG-----I--

PpGHL17_12 ATVDLAGRYMQDMITHLQ--SGIGTPLRPNVFV-EFYLFGLLDENSK----SIAPGP-----F--

PpGHL17_13 ATRELAGTYNQQLINHLQ--SNKGTPMRPNTFT-EFYMFGWLDENIK----SILPGA-----F--

PpGHL17_14 ANITLAQKFNQQLVNHVE--SGKGTPLRPGKL--EAYVFSLLDENAK----STLPGN-----F--

PpGHL17_15 ANIGLAQKFNQQLVNHLE--SGVGTPLRPGKL--EAYLFGLLDENAK----STLPGN-----F--

PpGHL17_16 ATIELAQKFNQQLVNHLE--SGVGTPLRPGKL--EAYVFGLLDENAK----STLPGN-----F--

PpGHL17_17 ASLLNAKTYNNNLVQHVL---RNGTPVRPNVKI-QTFIFALFNENQK-------QGY-----PI-

PpGHL17_18 CNLANAATYNRRLVRKMVSTSKVGTPLKPGVYI-PTFIFALFNENQK-------TGQ-----GT-

CaGHL17_1 PSVENAADQWQKGICALR---AWGI---------NVAVYEAFDEAWK----PDTSGT-----SSV

AcGHL17_1 ASTENAATYYQSAVCGIL---DWGI---------DVFYFEAFDEAWK-PDTKGDNGE-----MQD

AfgHL17_1 ASTANAADYYKSAVCGML---AWGV---------DVFYFEAFDESWK-PKTKGDNGE-----MQD

AnGHL17_1 AGTQNAAQYYKSAVCPML---QKGT---------DVFYFEAFDEVWK-PNSTGDNGQ-----SMD

CglGHL17_1 PSVDNAKQFWKEGICAMR---AWGV---------NVIVFEAFDEDWK----PNTSGT-----SDV

DhGHL17_1 PTLENAKNYWQNAICAIR---GWGV---------NVAVFEAFDEEWK----PNTSGT-----SDV

AgGHL17_1 PSLENAKQFWKEGICAMR---SWGI---------NTIVFEAFDEDWK----PDTSGI-----SDV

FgGHL17_1 ATTASLQQYYNNVGCWLW--QQKDA---------SGFWFTAFDTPAQ-------TTE-----V--

KlGHL17_1 PGLDNAKQFWADGICAMR---GWGV---------NVIVFEAFDEDWK----PDTSGI-----SDV

ScGHL17_1 PSVDNAKQFWKEGICSMR---AWGV---------NVIVFEAFDEDWK----PNTSGT-----SDV

SsGHL17_1 PSVDNAADFWQEAICAMR---GWGV---------NVAVFEYSDEIWK----PDTSGT-----SDV

SpoGHL17_1 ATVDIASEFFQEALCNIR---RKGI---------DIFFFEAFDEDWK-------GDS-----SSV

YlGHL17_1 PGVKQAAQFWQEGICAIR---AWGI---------NTLVFEAFDETWK----PDTKGDNGEEVSGV

AT3G57270.1 TNIENARIYNNNLIKHVK----NGTPKRPGKEI-ETYLFAIYDENQK-------PTP-----PYV

AT3G57260.1 TSVENAKTYVNNLIQHVK----NGSPRRPGKAI-ETYIFAMFDENKK------EPTY--------

AT3G57240.1 TGVDNARTYVNNLIQTVK----NGSPRRPGRAT-ETYIFAMFDENSK-------QGP-----ET-

AT4G16260.1 ASFDNARAFYTNLASRVR--ENRGTPKRPGRGV-ETYLFAMFDENQK-------SPE-----I--

AT5G56590.1 ASSDNAETYNSNIIRHVV--TNQGTPAKPGEAM-NVYIFSLFNENRK-------AGL-----DS-

AT1G77790.1 TSVDNAKAYNMGIRTCGRSAERKRTPRRQNTPV-DVFLFAMFKENQK-------DGP-----V--

AT1G77780.1 TSVENARAYNQGLLKKLT--TGKGTPRRPNVPV-ITFFFEMFNEDLK-------QGA-----V--

AT4G26830.1 ASESNAAAYNAGLVKRVL--TGKGTPLRPTEPL-NVYLFALFNENQK-------PGP-----TS-

AT4G29360 ATPENALAYNTNLIRHVI--GDPGTPAKPGEEI-DVYLFSLFNENRK-------PGI-----ES-

AT3G07320 ANIYNAATYNRNVVKKLAADPPVGTPARPGKVL-PAFVFALYNENQK-------TGP-----GT-

AT2G16230 PSVENAKAYNGNLIAHLR--SMVGTPLMPGKSI-DTYIFALFDENLK-------PGP-----SF-

AT5G20340 TTPDIAGTYNRNFVKHIA--SGKGTPKRPNKGI-DGFLFATFNENQK-------PVG-----T--

AT2G05790 ATLANAASYNGNLIRRIL--TRGGTPLRPKADL-TVYLFALFNENKK-------LGP-----TS-

AT5G55180.1 AGSANAAAYNGGLVKRVL--TGNGTPLKPKEPL-NVYLFALFNENQK-------TGP-----TS-

AT5G20330 TTPAIASTYNRNFVKHIA--SGKGTPKRPNKSM-NGFLFATFNENQK-------PAG-----T--

AT2G01630 ATVENANTYNSNLIQHVI--NKTGTPKHPGTAV-TTYIYELYNEDTR-------PGP-----VS-

AT4G34480 ASVDNAKAYNGNLIAHLR--SMVGTPLMPGKPV-DTYIFALYDENLK-------PGP-----SS-

AT1G66250 ATLDNANTYNSNLIRHVL--NKTGTPKRPGIAV-STYIYELYNEDTK-------AGL-----S--

AT5G20390 TTPDIASIYNTNFVKHVE--SGKGTPKRPKSGI-SGFLFATFNENQK-------PAG-----T--

AT3G61810 ANLENARVFNQRLVEHLR--RRWNKV--------PVYIFALFDEDQK-------TGN-----AV-

AT1G32860 ATPENAKRYNGNLIKMMMSGKKTKTPLKPNNDL-SIYVFALFNENLK-------PGP-----TS-

AT2G27500 ASPENAALYNGNLLKLIQ--QRKGTPAKQSVPI-DVYVFALFNENLK-------PGP-----VS-

AT1G33220 TTPDIAGTYNRNFVKHIV--SGKGTPKRPNNGM-DGFLFATFNENQK-------PAG--------

AT3G15800 ASVKNARTYNRNLRKRLQ--KRKGTPYRPDMVV-RAYVFALFNENSK-------PGP-----TS-

AT3G23770 ANIVNAATYNRNLIKKMTANPPLGTPARRGAPI-PTFLFSLFNENQK-------PGS-----GT-

AT5G42720 ATVENARAYNKNLIAHLK--SGSGTPLMPGRVI-DTYLFALYDENLK-------PGK-----GS-

AT2G26600 ATPENARTYNYNLRKRLA--KKKGTPLRPKTVL-KAYIFALFNENSK-------PGK-----SS-

AT4G14080.1 ANILNAATYNRNLIKKMSASPPIGTPSRPGLPI-PTFVFSLFNENQK-------SGS-----GT-

AT3G46570 VGLDYAAAYNGNLIKHVN--SGKGTPLMPNRVF-ETYVFSLFNENLK-------SSV-----S--

AT4G18340 ASPVNAATYNRNLMMRQF--AGEGTPARRNARL-DVYIFALFNEDMK-------PGP-----TS-

AT1G30080 ATVANAAVYNKNILRRQL--QNEGTPLRPNLSF-DVYLFALFNEDLK-------PGP-----TS-

AT5G42100 ATCDNARKYNGNLIKMMM-SKKMRTPIRPECDL-TIFVFALFNENMK-------PGP-----TS-

AT5G58090 ANLDYAKKFNQGFMAHIS--GGKGTPRRPGPI--DAYLFSLIDEDAK----SVQPGY-----F--

AT1G11820 ATIDNADTYNSNLIKHVF--DRTGTPLHPEMTS-SVYIYELFNEDLR-------APP-----VS-

AT3G24330 ANVKSAKRFNQGMVKHAM--SGNGTPARKGVIM-DVYLFSLVDEDAK----SIAPGT-----F--

AT4G31140.1 ANLMYARRYNQGFMNRQK--ANKGTPMRPGAM--DAYLFGLIDEDAK----SIQPGN-----F--

AT4G17180 ANLTAARVFNQGLISHVL--SNKGTPLRPGSPPADVYLFGLLDEGAK----STLPGN-----F--

AT3G13560 ATVANAETFNTNLIKRVL--NNSGPPSQPDIPI-NTYIYELYNEDKR-------SGP-----VS-

AT5G24318 VDVDTAAEFNKNLIARVD--SGTGTPLMPNRTF-ETYIFALFNENLK-------SGP-----TS-

AT2G39640 CSPQNAENYNLNIIKRAQ---VIGTPLMPNRHI-DIFIFALFNEDGK-------PGP-----TR-

AT2G19440 ANSGSAYRFYNGLLPRLG--ENRGTPLRPTYI--EVYLFGLLDEDAK----SIAPGE-----F--

AT3G04010 ANVPNAERFYSGLLPRLA--NNVGTPMRKGYI--EVYLFGFIDEDAK----SVAPGN-----F--

AT5G18220 ANIPNAERFYTGLLPKLA--ANRGTPMRPGYI--EVYLFGFIDEDAK----SIAPGN-----F--

AT1G64760 ANAGSAYRFYNGLLPRLG--TNKGTPLRPTYI--EVYLFGLLDEDAK----SIAPGP-----F--

AT5G58480 ATSLTAEAFFKGLIVHLE----KKTASLPRPPV-ETYIESLLDEDQR----NLSAGN-----F--

AT3G55430 CSPANAAWFNLNIIKRAQ---GQGTPLMPNRRF-ETYIFGLFNEEGK-------PGP-----TA-

AT5G20870 ANPAMAQRFNQGLLNRIL--QGQGTPRRRMAP--EVYIFSLVDEDAK----SIDPGK-----F--

AT3G55780 ATLLYSEMFLKALLTHLR--SGCGTPLRKEGVS-EVYIFELVEKDAK-------QG---------

AT5G64790 ASPKLAEKFYAGLMKRLA--KDGGTPTRPERL--EVYLFGFLDEDMK----SILPGP-----F--

AT5G20560 TTPALQYTYIGNFVKHVA--SGKGTPKRPNSRI-DAYIFETYNENQK-------PVG-----I--

OsGHL17_1 ATPEYAGIYIGNLLRRIE--MKQGTPLRPSSPI-DVYVFALFNENLK-------PGP-----AS-

OsGHL17_2 ATVENAAAYNGNLMQRIA--MNQGTPLKPNVPI-DVFVFALFNEDMK-------PGP-----TS-

OsGHL17_3 ATPENAGTYIGNLLRRIE--MKQGTPLRPQAPI-DVYVFALFNENLK-------PGP-----AS-

OsGHL17_4 ADPANARAYNFNLRKRLF--LRKGTPYRPGRVA-----KALFNENLK-------PGP-----TT-

OsGHL17_5 ASVENARTYNFNLRKRLF--LRKGTPLKPKRPV-KAYIFALFNENSK-------PGP-----SS-

OsGHL17_6 ATPDNAQTYNTNLIRHVV--NDSGTPAKPGEEI-DVYIFSLFNENRK-------PGI-----ES-

OsGHL17_7 ATVGNARAFVSGLVSHLR--SMAGTPRAPGKPV-DTYLFAVYDEDLK-------PGK-----PS-

OsGHL17_8 ATSDNADTYNSNLIRHVM--NTTGTPKHPGVAV-PTYIYELYDEDTR-------PGS-----TS-

OsGHL17_9 ANVDNALAYNTNLIRHVL--NNSGTPGQPNNQV-STFIFELFNEDLR-------AGP-----VS-

OsGHL17_10 ATRDNADAYNSNLIKHVN--DKPGTPMRPGAQA-SVYIYELFNEDLR-------PGP-----VS-

OsGHL17_11 ATVPYAQRFYSGLLKRLA--ARRGTPLRPRARI-EVYLFGLMDEDTK----SVAPGN-----F--

OsGHL17_12 ANLKLARRYYDGLLKKLS--KKEGTPLRPGKM--DVYMFGLFDEDMK----SILPGN-----F--

OsGHL17_13 ARVDLAQRFYAGLLKRLA--ANVGTPARPNQYI-EMYLFGLVDEDMK----SVAPGS-----F--

OsGHL17_14 ANAANAQRFNQGLFDRIL--AGKGTPRRPQMP--DVYVFALLDEDAK----SIDPGS-----F--

OsGHL17_15 ARADLAQRFYAGLLRKLA--SNAGTPLRPNQYV-EVYLFSLVDEDAK----SVAPGN-----F--

OsGHL17_16 ANVRYAQKFYDGFLKKMV--RNIGTPLRPGWM--EVYLFALIDENQK----SVLPGR-----F--

OsGHL17_17 ANLTAARAFNQGLMNRVM--NNKGTPLRPGVPPADVYLFSLFDEEQK----SILPGN-----F--

OsGHL17_18 ANLTAARAFTQGLISHVL--SNKGTPLRPGVPPMDVYLFSLLDEEQK----STLPGN-----F--

OsGHL17_19 ANPQYARQFNQGFLTHIA--SGQGTPLRPGPV--DAYLFSLIDEDQK----SIEPGN-----F--

OsGHL17_20 ANIQMAQHFNQGFLTHIA--TGRGTPMRPGPV--DAYLFSLIDEDEK----SIQPGN-----F--

OsGHL17_21 ATATYAQRFYNGLLKRLA--ANAGTPARPGQYI-EVYLFGLLDEDAK----SVAPGD-----F--

OsGHL17_22 ATADNARAYVSNLVSHLR--SGAGTPLMPGKPV-DTYLFALYDEDLK-------PGP-----TS-

OsGHL17_23 ATVDNARAYNGNLVAHLK--SQVGTPRTPGKSV-DTYLFALYDEDLK-------GGP-----ES-

OsGHL17_24 ATPAIAQSFMTGLVNHLA--RKSGTPLRPKVPPIETYLFSLLDEDQR----SIASGS-----Y--

KfGHL17_1 ELHWGLLY-ANGTSKYAG------NSSS-------------------------------------

KfGHL17_2 QRHRGLFT-PQGAPKFAA------NLRG-------------------------------------

PmGHL17_1 TPRWRS---PAPSPRRRE-----------------------------------------------

CgGHL17_1 QGAWGLFD-NTSAPKYTV------NFQG-------------------------------------

NtGHL17_1 EHFWGLYS-MDGSPKYDI------DLTA-------------------------------------

NtGHL17_2 ERHWGILD-SSGTPKYNL------DLTG-------------------------------------

NtGHL17_3 ERHFGLFT-NTGTPKFDF-----------------------------------------------

PpGHL17_1 ERFFGLFN-ADKSLVYNL------GIVT-------------------------------------

PpGHL17_2 ERFFGLFN-PDKSIVYNL------GIVTNTYPPISATPPYTSPISPTPPVVYPPPQPSLPPPVYS

PpGHL17_3 EKNWGLFH-PDMSPVYTA------SLST-------------------------------------

PpGHL17_4 ERNWGLLY-PDGTAVYPI------DIKS-------------------------------------

PpGHL17_5 ERNFGLFY-PNETKVYDI------SFT--------------------------------------

PpGHL17_6 ERNWGLLY-PNLGAVYPI------DLTG-------------------------------------

PpGHL17_7 TRSMGLFS-AEMAPVYAV------DLSG-------------------------------------

PpGHL17_8 AQHWGLFY-VNGTHKYGFNYLNGSDVPG-------------------------------------

PpGHL17_9 ENNWGLYH-PNMTPVYSI------NLSV-------------------------------------

PpGHL17_10 ERHWGMFY-YDGVAKYPL------DLAA-------------------------------------

PpGHL17_11 ENNWGLYH-PNMTPVYSI------NLSV-------------------------------------

PpGHL17_12 ERHWGVFY-YDGVAKYPL------NLAS-------------------------------------

PpGHL17_13 ERHWGMFF-YDGVAKYEL------NLAG-------------------------------------

PpGHL17_14 ERHWGVFN-FDGSVKYLL------DLTG-------------------------------------

PpGHL17_15 ERHWGIFN-FDGTIKYPL------DLTG-------------------------------------

PpGHL17_16 ERHWGIFN-FDGTVKYPL------DLTG-------------------------------------

PpGHL17_17 EKNFGLYY-PDKRPVYDI------RLQA-------------------------------------

PpGHL17_18 EKHWGLLY-PNGTNVYSI------DMTG-------------------------------------

CaGHL17_1 EKHWGVWQ-SDKTLKYSI-----------------------------------------------

AcGHL17_1 EKHWGAFS-ADRKVKFDL-----------------------------------------------

AfgHL17_1 ETHWGAFT-ADRKAKFDL-----------------------------------------------

AnGHL17_1 EKHWGLFT-ADRKLKFDV-----------------------------------------------

CglGHL17_1 EKHWGVWT-SGDNLKYSL-----------------------------------------------

DhGHL17_1 EKHWGVFD-ENNKPKFDL-----------------------------------------------

AgGHL17_1 EKHWGVFT-ADRKPKFNL-----------------------------------------------

FgGHL17_1 EKYFGVAN-QDRKLKFSL-----------------------------------------------

KlGHL17_1 EKYWGVWD-ANRNLKFKL-----------------------------------------------

ScGHL17_1 EKHWGVFT-SSDNLKYSL-----------------------------------------------

SsGHL17_1 EKHWGVWD-SNGKLKYDI-----------------------------------------------

SpoGHL17_1 EPYFGAMY-SNRTLKYNL-----------------------------------------------

YlGHL17_1 EKYWGVYD-SNLKPKFDT-----------------------------------------------

AT3G57270.1 EKFWGLFY-PNKQPKYDI-----------------------------------------------

AT3G57260.1 EKFWGLFH-PDRQSKYEV------NFN--------------------------------------

AT3G57240.1 EKFWGLFL-PNLQPKYVV-----------------------------------------------

AT4G16260.1 EKNFGLFF-PNKQPKFPI-----------------------------------------------

AT5G56590.1 ERNWGLFY-PDQTSVYQL------DFTG-------------------------------------

AT1G77790.1 EQSFGIFA-PDMTPVYDL-----------------------------------------------

AT1G77780.1 EQSFGFFD-PNMAPVYDM-----------------------------------------------

AT4G26830.1 ERNYGLFY-PNEGKVYNV------PFTK-------------------------------------

AT4G29360 ERNWGMFY-ANGTNVYAL------DFTG---------------------------------ENTT

AT3G07320 ERHFGLLH-PNGTQVYGI------DLSG-------------------------------------

AT2G16230 EQSFGLFK-PDLSMAYDI------GLTK-------------------------------------

AT5G20340 EQNFGLYNPNDMKPIYNL-----------------------------------------------

AT2G05790 ERNYGLFF-PDEKKVYDI------PFTT-------------------------------------

AT5G55180.1 ERNYGLFY-PNENKVYDV------SLNG-------------------------------------

AT5G20330 EQNFGLYNPSDMKPIYKL-----------------------------------------------

AT2G01630 EKNWGLFY-TNGTPVYTL------RLAG-------------------------------------

AT4G34480 ERAFGLFK-TDLSMVYDV------GLAK-------------------------------------

AT1G66250 EKNWGLFN-ANGEPVYVL------RLTN-------------------------------------

AT5G20390 EQNFGLYNPTDMKPIYKM-----------------------------------------------

AT3G61810 EKHWGLLY-GNGSRKYDL------NFSP-------------------------------------

AT1G32860 ERNYGLFK-PDGTQAYSL------GFAL-------------------------------------

AT2G27500 ERNYGLFY-PDGKPVYNV------GMQG-------------------------------------

AT1G33220 ---------TDMKPIYKL-----------------------------------------------

AT3G15800 ERNFGLFK-PDGTIAYDI------GLTG-------------------------------------

AT3G23770 ERHWGILN-PDGTPIYDI------DFSG-------------------------------------

AT5G42720 ERAFGLFR-PDLTMTYDI------GLTK-------------------------------------

AT2G26600 ETHFGLFK-PDGTISYDI------GFNN-------------------------------------

AT4G14080.1 QRHWGILH-PDGSPIYDV------DFTG-------------------------------------

AT3G46570 EQNFGLFK-PDFTPVYDV------GIMK-------------------------------------

AT4G18340 EKNYGIFQ-PDGSLAYNL------GFST-------------------------------------

AT1G30080 ERNYGLYQ-PDETMTYNV------GLLS-------------------------------------

AT5G42100 ERNYGLFN-PDGTPVYSL------GIKT-------------------------------------

AT5G58090 ERHWGIFT-FDGLPKYAL------NLGT-------------------------------------

AT1G11820 EASWGLFY-GNSTPVYLL------HVSG-------------------------------------

AT3G24330 ERHWGIFE-FDGRPKYEL------DLSG-------------------------------------

AT4G31140.1 ERHWGIFY-IDGQPKYQL------SLGS-------------------------------------

AT4G17180 ERHWGIFS-FDGQAKYRL------NLGL-------------------------------------

AT3G13560 ERNWGILF-PNGTSVYPL------SLSG-------------------------------------

AT5G24318 ERNFGIFR-SDLTPIYDI------GILR-------------------------------------

AT2G39640 ERNWGIFK-PDFSPMYDV------GVLK-------------------------------------

AT2G19440 ERHWGIFK-FDGQPKFPI------DLSG-------------------------------------

AT3G04010 ERHWGIFK-FDGQPKFPV------DFRG-------------------------------------

AT5G18220 ERHWGIFK-YDGQPKFPA------DLSG-------------------------------------

AT1G64760 ERHWGIFK-FDGQPKFPI------DLSG-------------------------------------

AT5G58480 ERHWGVFT-FDGQAKYNF------SFNH-------------------------------------

AT3G55430 ERNWGLFR-ADFSPVYDV------GLLR-------------------------------------

AT5G20870 ERHWGIFS-YDGAVKYPL------SLGN-------------------------------------

AT3G55780 IRNWGLLH-HNMTSKYSF------DFSD-------------------------------------

AT5G64790 ERHWGIFR-YDGTPKFML------DFTG-------------------------------------

AT5G20560 YQHFGLYD--------PY-----------------------------------------------

OsGHL17_1 ERNYGLFY-PDGTPVYDV------GLRG-------------------------------------

OsGHL17_2 ERNYGLFY-PNGSPVYAI-----------------------------------------------

OsGHL17_3 ERNYGLFY-PDGTPVYNV------GLRG-------------------------------------

OsGHL17_4 ERHYGLFK-PDGSVSIDL------GFKG-------------------------------------

OsGHL17_5 ERHYGLFN-ADGRIAYDI------GYEG-------------------------------------

OsGHL17_6 ERNWGLFF-PDQSSIYSL------DWTG-------------------------------------

OsGHL17_7 EKSFGLFQTTTLAETYPT------GLMR-------------------------------------

OsGHL17_8 EKYWGLFD-MNGIPAYTL------HLTG-------------------------------------

OsGHL17_9 EKNWGIMF-PNATTVYSL------TFED-------------------------------------

OsGHL17_10 EANWGLFH-GNGTPVYLL------HVSG-------------------------------------

OsGHL17_11 ERHWGIFT-FDGRPKFPL------DLRG-------------------------------------

OsGHL17_12 ERHWGIFT-YDGKPKFPM------DLSG-------------------------------------

OsGHL17_13 ERHWGVLR-YDGQPKFAM------DLAG-------------------------------------

OsGHL17_14 ERHWGVFN-YDGSRKYNL------RLAG-------------------------------------

OsGHL17_15 ERHWGILR-YDGQPKYSM------DLAG-------------------------------------

OsGHL17_16 ERHWGLLT-YDGKPKFSM------DLSG-------------------------------------

OsGHL17_17 ERHWGIFS-FDGQAKYPL------NLGL-------------------------------------

OsGHL17_18 ERHWGVFS-FDGQAKYPL------NLGL-------------------------------------

OsGHL17_19 ERHWGVFY-YDGQPKYPL------SLRG-------------------------------------

OsGHL17_20 ERHWGIFT-YDGLPKYQL------NLGQ-------------------------------------

OsGHL17_21 ERHWGILR-FDGQPKYPV------DLTG-------------------------------------

OsGHL17_22 ERSFGLYH-TDLTMAYDA------GLTS-------------------------------------

OsGHL17_23 ERSFGLYR-TDLTANYDI------GLAA-------------------------------------

OsGHL17_24 ERHHGIFT-FDGQAKYYV------NLGQ-------------------------------------

KfGHL17_1 ---------GVPAAAPSGALSNTTALAPSAANSTSRTGTWCIAVSSA-----DSQQ-------LT

KfGHL17_2 ----------------TNAPPRPMNGLQDLIRL---PTRFCVAKPGV-----PDTA-------LL

PmGHL17_1 -----------------------------------------------------------------

CgGHL17_1 --------------------------SKEVVAVQPQTLRWCVVRDGA-----KDSS-------VR

NtGHL17_1 ----------------------------GWVPDLQMTSRWCVAAADA-----DTWT-------LN

NtGHL17_2 -----------------GGGDVPLQIPYEPKFR---KRQWCLAPDRVRFENLNETRDALFPQLCP

NtGHL17_3 -----------------SIASSPGGPASPVQWN---GHNWASSCDFKGNDLSNVQT-------PA

PpGHL17_1 --------------------NTYPPATATPPYGGNPGKTWCVAKPGS-----SERD-------VA

PpGHL17_2 PPVPPANIYPTPPTPPVLTPSPPYYAPVQSGNP---GKTWCVAKPGS-----GESE-------VA

PpGHL17_3 -----------------------------------------------------------------

PpGHL17_4 -----------------------------------------------------------------

PpGHL17_5 -----------------------------------------------------------------

PpGHL17_6 ---------------------------------------------------------------QM

PpGHL17_7 ---------------------SQVTQVPIGPPAASATRTWCVAKQDA-----SQDA-------LQ

PpGHL17_8 --GGGGGGGGNGSTPGSPPGSGGGGGGGSSGGAIPGQKVWCIAKSSA-----SNTS-------LI

PpGHL17_9 -----------------------------------------------------------------

PpGHL17_10 ---------------GTGAPPQTIKNAEFPPYM---SAQYCVLNENA-----DRTN-------LS

PpGHL17_11 -----------------------------------------------------------------

PpGHL17_12 ---------------GTTNAATAIKSLVNPPYM---AEQFCVLNTSV----IDRTN-------LT

PpGHL17_13 ------------------GTDAPAQTLKNAEYPPYMSAQYCVLNTYA-----DRTN-------LS

PpGHL17_14 ---------------GVSGTQTRLIGAQNVPYY---PSQWCVLNPIK-----DLST-------LP

PpGHL17_15 ---------------GVTGRQTSLVGSKNVPYY---PHQWCVLKTTA-----DLSL-------LP

PpGHL17_16 ---------------GVDGTQTALVGSKNVPYY---PRQWCVLKPTA-----DLSL-------LP

PpGHL17_17 -----------------------------------------------------------------

PpGHL17_18 ----TLSDGQYTPLSDNPIFTTAPPPTLPPGNVPSTTGTWCVAKPGM-----NPPM-------LQ

CaGHL17_1 -----------------------------------------------------------------

AcGHL17_1 -----------------------------------------------------------------

AfgHL17_1 -----------------------------------------------------------------

AnGHL17_1 -----------------------------------------------------------------

CglGHL17_1 -----------------------------------------------------------------

DhGHL17_1 -----------------------------------------------------------------

AgGHL17_1 -----------------------------------------------------------------

FgGHL17_1 -----------------------------------------------------------------

KlGHL17_1 -----------------------------------------------------------------

ScGHL17_1 -----------------------------------------------------------------

SsGHL17_1 -----------------------------------------------------------------

SpoGHL17_1 -----------------------------------------------------------------

YlGHL17_1 -----------------------------------------------------------------

AT3G57270.1 -----------------------------------------------------------------

AT3G57260.1 -----------------------------------------------------------------

AT3G57240.1 -----------------------------------------------------------------

AT4G16260.1 --------------------------------------TFSAARDGT------------------

AT5G56590.1 -------------------KSNGFHSNSSGTNSSGSSNSWCIASSKA-----SERD-------LK

AT1G77790.1 -----------------------------------------------------------------

AT1G77780.1 ---------------------------------------WNIARTSN------------------

AT4G26830.1 ------------------KSTTPVNGNRGKVPVTHEGHTWCVSNGEV-----AKEK-------LQ

AT4G29360 PVSPTNSTTGTSPSPSSSPIINGNSTVTIGGGGGGGTKKWCIASSQA-----SVTE-------LQ

AT3G07320 ---------------KTEYKESLPAPENNDLYK---GKIWCVVAKGA-----NWTQ-------LG

AT2G16230 --------------------TTSSQTSQSPQLGKVTSMGWCVPKEDA-----TQEQ-------LQ

AT5G20340 -----------------------------------------------------------------

AT2G05790 --EGLKHYRDGGHTPVTGGDQVTKPPMSGGVSKSLNGYTWCVANGDA-----GEER-------LQ

AT5G55180.1 ----------------KSTPVNDNKEKVVPVKPSLVGQTWCVANGKT-----TKEK-------LQ

AT5G20330 -----------------------------------------------------------------

AT2G01630 --------------------------AGAILANDTTNQTFCIAKEKV-----DRKM-------LQ

AT4G34480 -----------------------SSSSSQTPSGKVTSSGWCVPKKGA-----TNEE-------LQ

AT1G66250 --------------------------SGSVLANDTTNQTYCTAREGA-----DTKM-------LQ

AT5G20390 -----------------------------------------------------------------

AT3G61810 -----------------------------------------------------------------

AT1G32860 ---------------NDVVRGASGGGTGGGNSSSGGGRDKSPVFPVS------------------

AT2G27500 -----------------YLPDIIYTSRATTIKI---LNLWRVV--------------------MG

AT1G33220 -----------------------------------------------------------------

AT3G15800 -----------------------LKSSSATRYRFKSS--------------------------LV

AT3G23770 -------------RRSFSGFDSLPKPSNNVPFK---GNVWCVAVDGA-----DEAE-------LG

AT5G42720 --------------------TTNYNQTSMAPLSPTRPRLPPAAAPTR------------------

AT2G26600 ---------------------LKSDSPKSLISSSKSARYYVALVISV------------------

AT4G14080.1 -------------QTPLTGFNPLPKPTNNVPYK---GQVWCVPVEGA-----NETE-------LE

AT3G46570 -----------------------------------------------------------------

AT4G18340 -------------MSTTTANSESVTYSSSATKAKRSLEYWTILILAM----------------VQ

AT1G30080 -----------------SSSLTSTSTTSSTSIISLTSSASTALKKGK-----------------Q

AT5G42100 -----------------SSTHSSGSGSSNSTGGSSSGGGGNTGGSSS------------------

AT5G58090 ------------------TNTGALIQAKGVRYL---ERKWCVMKPNV---RLDDPQ-------VA

AT1G11820 --------------------------SGTFLANDTTNQTYCIAMDGV-----DAKT-------LQ

AT3G24330 -----------------KGNDKPLVPVEDVKYL---PKTWCILDPNA----YNLDD-------LP

AT4G31140.1 --------------------GNGLIPAKDVHYL---AKKWCILAPNA---NLQDPQ-------LG

AT4G17180 -------------------GNRGLKNAKNVQYL---PSRWCVAHPSR-----DMTQ-------VG

AT3G13560 -----------------GSSSAALNG----------SSMFCVAKADA-----DDDK-------LV

AT5G24318 ----------------PTFRSSDPVYNPRSPVRGSSSKRWCVTKAGA-----ETVA-------LQ

AT2G39640 ----------------GGGSPLPFPPINN-------NGKWCVGKPEA-----TLMQ-------LQ

AT2G19440 -----------------QGQNKLLIGAENVTYQ---PKKWCMFNTEA----KDLTK-------LA

AT3G04010 -----------------QGQKKFLTGAQNVQYF---LNQWCMFNPNG---RGNMSR-------LG

AT5G18220 -----------------AGQKKILTGAQNVQYL---RNQWCMFNPNALTFSNNTNQ-------LG

AT1G64760 -----------------QGQSKFLIGAQNVPYL---PNKWCTFNPEA----KDLTK-------LA

AT5G58480 ---------------------KNQVNAQNVQYL---PPKWCVVNNNK-----DLSN-------AS

AT3G55430 ----------------NGQGGGGRPALPAPSTA---GGKWCVARSGA-----TNTQ-------LQ

AT5G20870 --------------------GRPLVPTKGVRYQ---AREWCVLSTQAAGNGAAT---------WQ

AT3G55780 -----------GGKVRRFKEILVGFFVQVVMIG---YGYVCRYFETI-----IWYV-------VN

AT5G64790 -----------------QGRQMVPVAAKGVQYL---EKQWCVVNKDT----VNLDE-------VG

AT5G20560 -----------------------------------------------------------------

OsGHL17_1 -------------------------------YLPPMDESKSARKAVS----------------VL

OsGHL17_2 ------------------NTGAGGVSGRTGPFDPYSAQMFSSASRLA----VRTASLTLLLLVLP

OsGHL17_3 -----------------------YLPPMASHEAATQVIHWFLLIATA------------------

OsGHL17_4 -----------------LVPSSSLPSSSIISFKRARERGWMALVQYS------------------

OsGHL17_5 -------------------LLPSSAPSYFLSLRKIQAGGWIVHYSAT------------------

OsGHL17_6 ------------------RGNVDIMTGGNITNA---NGTWCVASANA-----SETD-------LQ

OsGHL17_7 -----------------NGTAAGLAPAMAPAAAAPTLPVKPSPAPAR----------------LP

OsGHL17_8 --------------------------SGVLLANDTTNQTYCVAREGA-----DEKM-------LQ

OsGHL17_9 ----------------------MATTNTDSPVL---RGTFCVANSSA-----PHSA-------LK

OsGHL17_10 -----------------------AGGFLANDTT---DRTFCIASDDA-----DEKA-------VQ

OsGHL17_11 -----------------AGRPAMPVPAKGVKYL---PRRWCVLNPNVTDDDAGR---------LA

OsGHL17_12 -----------------HGNDKPLAGVPGVEYL---PKQWCVFDDGA----EDKSK-------LP

OsGHL17_13 -----------------QGRNTMLVPAKGIEYL---PKTWCVINTNA----KDVSK-------LG

OsGHL17_14 --------------------GRSIAPARGVRYL---SRQWCVLRPDA---SPADPA-------IG

OsGHL17_15 -----------------QGRDTALVAARGVAYL---PRAWCVLNPSATPDAMSR---------VG

OsGHL17_16 -------------------DGLDNLVGVEVEYL---PAQWCVFNKDAKDKFKD----------LP

OsGHL17_17 -------------------GNPVLKNAREVPYL---PSRWCIANPAQ-----NLDN-------VA

OsGHL17_18 -------------------GSPVLKNAKDVPYL---PPRWCVANPGR-----DLNN-------VE

OsGHL17_19 -------------GGNGNGNGSTLMPAKGVTYL---QRRWCVMKPSA---SLVDQK-------VG

OsGHL17_20 --------------------SHGLLKAKNVKYL---ERKWCVLKPSI---GLTDPR-------LS

OsGHL17_21 -----------------QGQNTMLVPAKGVTYL---PRTWCVINTNA----KDTSK-------LA

OsGHL17_22 ---------------SSGGAASPSNGGASQQQPRGGGGGWCVASAGA-----TEAD-------LQ

OsGHL17_23 ----------APGTAAPATVTPVTVQNTPQPSRGMTPTGYCVTAAGV------------------

OsGHL17_24 -------------------GSKALENAPDVQYL---PSKWCVLDNNK-----DISN-------VS

KfGHL17_1 AGLNYAC--GPGGADCT------AIQP-GGPCYVQLGVANRVQSFASYAYNSYYQSHKLDPGACF

KfGHL17_2 LQQIQAC---SGGADCT------PFAN-GGSCF----LPDTVAAHTSYAFNDYYQKHAQWSPACD

PmGHL17_1 -------------------------------------APH-------------------------

CgGHL17_1 GAYDWVC--KIGKIDCS------PLFNEDGAC-----GNLTLQQQASYAFNEWWQRQGQRDFTCD

NtGHL17_1 KALDWAC---AQPIDCS------PIRSLGSGCYIADSDPDSTRRQASYVMNSYYQLKRQDPSACA

NtGHL17_2 KNPQNAS----VQSDCT------STAG-NSSCS----SPADPRREMSVWVNSIYQISGQKRDTCQ

NtGHL17_3 DKCGSTCAQTTGCTHYTWTHWPLGLEHGRGTCWM---KSGTVSKSDAYRTQDESSICGVVPPPVD

PpGHL17_1 NALNFAC--GEGGADCG------TIQP-GGPCY----NPNTLLSHASFAFNVYYQKMGRNYWNCY

PpGHL17_2 NALNFAC--GEGGADCG------EIQA-GGACY----SPNTVLSHASFAFNTYYQKMGRNYWNCY

PpGHL17_3 -----------------------------------------------------------------

PpGHL17_4 ------------------------------------------ARNKNYIHSK-------------

PpGHL17_5 -----------------------------------------------------------------

PpGHL17_6 LDTQYA-----------------PILP--------------------------------------

PpGHL17_7 AALDYAC--GLGQADCL------PIQP-GQACF----LPNTRTSHASWAINSYYQKNSNSANACN

PpGHL17_8 QGIDWAC--GAGKAKCD------PIQR-GGDCY----LPDTPYSHASYAFNIHYHWFQTDPRSCI

PpGHL17_9 -----------------------------------------------------------------

PpGHL17_10 QNVAFAC----SRTDCT------PLYP-GSSC-----GGLSAVQNASYSFNSYFQFQNQDPNACN

PpGHL17_11 -----------------------------------------------------------------

PpGHL17_12 QNVDYAC----GIADCT------ALNN-GSTCA-------TLAEPASYAFNSYFQAMSQDPGACN

PpGHL17_13 ENVAFAC----SRADCT------PLFP-GSSC-----AGLSLEQNASYSFNAYFQFQNQDPAACN

PpGHL17_14 ANLDYAC----SRADCT------PLTT-GGSC-----SGLTLQQNASYAFNQYYQFNNQLKSACD

PpGHL17_15 ANLDYAC----ARADCT------PLFY-GGSC-----SGLTLHQNASFAFNNYYQFNNQLQAACD

PpGHL17_16 ANLDYAC----GSTDCT------PLFS-GGSC-----SGLTLQQNASYAFNNYYQFNNQLPSACD

PpGHL17_17 -----------------------------------------------------------------

PpGHL17_18 AALDFAC--GPGGADCQ------PLQV-GGSCY----NPDTILDHSSYAFNSYYQRTKAAGGSCN

CaGHL17_1 --------------------------------------------------------------DCK

AcGHL17_1 --------------------------------------------------------------KCN

AfgHL17_1 --------------------------------------------------------------TCP

AnGHL17_1 -----TC----------------------------------------------------------

CglGHL17_1 --------------------------------------------------------------DCS

DhGHL17_1 --------------------------------------------------------------SCD

AgGHL17_1 --------------------------------------------------------------SCD

FgGHL17_1 -----TC----------------------------------------------------------

KlGHL17_1 --------------------------------------------------------------DCD

ScGHL17_1 --------------------------------------------------------------DCD

SsGHL17_1 --------------------------------------------------------------NCH

SpoGHL17_1 --------------------------------------------------------------NCT

YlGHL17_1 --------------------------------------------------------------TCK

AT3G57270.1 ------------------------------------------------NFY--------------

AT3G57260.1 -----------------------------------------------------------------

AT3G57240.1 ------------------------------------------------NFN--------------

AT4G16260.1 -----------------------AVE---------------------------------------

AT5G56590.1 GALDWAC--GPGNVDCT------AIQP-SQPCF----QPDTLVSHASFVFNSYFQQNRATDVACS

AT1G77790.1 --------------------------------------------------------------FCK

AT1G77780.1 -------------------------------------------ASPSWVIWTNWVIWTMLITRLF

AT4G26830.1 EALDYAC--GEGGADCR------PIQP-GATCY----HPESLEAHASYAFNSYYQKNSRRVGTCF

AT4G29360 TALDWAC--GPGNVDCS------AVQP-DQPCF----EPDTVLSHASYAFNTYYQQSGASSIDCS

AT3G07320 DALSYAC--SQGNNTCD------PIQR-GGPCQ----KPDLTVLHASYAFSSYWAQFRKIGGTCS

AT2G16230 DSLDWVC---GQGIDCG------PIMP-GGVCF----EPNNVASHTAYAMNLYFQKSPENPTDCD

AT5G20340 -----------------------------------------------------------------

AT2G05790 GGLDYAC--GEGGADCR------PIQP-GANCY----SPDTLEAHASFAFNSYYQKKGRAGGSCY

AT5G55180.1 EGLDYAC--GEGGADCR------PIQP-GATCY----NPESLEAHASYAFNSYYQKNARGVGTCN

AT5G20330 -----------------------------------------------------------------

AT2G01630 AALDWAC--GPGKVDCS------ALMQ-GESCY----EPDDVVAHSTYAFNAYYQKMGKASGSCD

AT4G34480 ASLDWAC---GHGIDCG------AIQP-GGACF----EPNNVVSHAAYAMNMYFQKSPKQPTDCD

AT1G66250 AALDWAC--GPGKIDCS------PIKQ-GETCY----EPDNVVAHANYAFDTYYHQTGNNPDACN

AT5G20390 -----------------------------------------------------------------

AT3G61810 -----------------------PI----------------------------------------

AT1G32860 -----------------------PVAP-------------DSASTGYLAISASPVTGKRKGKGAI

AT2G27500 LAVAWFILDMGDKMRMR------------------------------------------------

AT1G33220 -----------------------------------------------------------------

AT3G15800 SASAFTC--------------------------------------LLLLFHRLFHL---------

AT3G23770 QALNFAC--GRSNATCA------ALAP-GGECY----APVTVTWHASYAFSSYWAQFRNQSSQCY

AT5G42720 ---------------------------------------QTLPSPPQMILPSPVTPSDKNSGQTD

AT2G26600 -----------------------------------------------------------------

AT4G14080.1 ETLRMAC--AQSNTTCA------ALAP-GRECY----EPVSIYWHASYALNSYWAQFRNQSIQCF

AT3G46570 -------------------------------------------TNKVYTLQLL------------

AT4G18340 VVMLRLF----------------------------------------------------------

AT1G30080 RLMYWTC---------------------------------------VYLLAIHMLIRRSY-----

AT5G42100 ----------------------------GGGIY----QPVTGNPSPDYMSISSAGGKGRFVECVL

AT5G58090 PAVSYAC----SLGDCT------SLGV-GTSC-----ANLDGKQNISYAFNSYYQIQDQLDTACK

AT1G11820 AALDWAC--GPGRSNCS------EIQP-GESCY----QPNNVKGHASFAFNSYYQKEGRASGSCD

AT3G24330 DNIDYAC----SLSDCT------ALGY-GSSC-----NHLTATGNVSYAFNMYYQMHDQKTWDCD

AT4G31140.1 PSVSYAC----DHADCT------SLGY-GSSC-----GNLNLAQNVSYAFNSYYQVSNQLDSACK

AT4G17180 DHLRLAC----SEADCT------TLND-GGSC-----SQLGEKDNISYAFNSYYQMQMQHEKSC-

AT3G13560 DGLNWAC--GQGRANCA------AIQP-GQPCY----LPNDVKSHASFAFNDYYQKMKSAGGTCD

AT5G24318 RNIDYVC---GLGLDCR------PINE-GGLCY----LPNTVKAHSKYAMNLYYQTMEKHEFDCD

AT2G39640 ANIDWVC---SHGIDCT------PISP-GGICF----DNNNMTTRSSFIMNAYYQSKGCVDVVCD

AT2G19440 ANIDYAC----TFSDCT------ALGY-GSSC-----NTLDANGNASYAFNMYFQVKNQDEDACI

AT3G04010 DNINYAC----SHSDCT------ALGY-GSSC-----GNLDANGNASYAFNMYFQVQNQEAQACD

AT5G18220 DNVNYAC----TFSDCT------ALGY-GSSC-----GNLDEVGNASYAFNMYFQVQNQKAEACD

AT1G64760 ANIDYAC----TFSDCT------ALGY-GSSC-----NTLDANGNASYAFNMFFQVKNQDESACY

AT5G58480 ARALEAC----AVADCT------SILP-GGSC-----SGIRWPGNVSYAFNSLYQQNDHSAESCN

AT3G55430 DSINWVC---GQGVDCK------PIQA-GGSCF----NPSSLRTHASFVMNAYFQSHGRTDGACN

AT5G20870 ASATYAC----QNADCT------SLGP-GSSC-----AALDPTANASYAFNMYFQKMDHRRGSCD

AT3G55780 FMNNYECLDTRKTLICD------DYSS--------------------------------------

AT5G64790 PDLDYAC----YHGDCT------AMEA-GSTC-----SKLTKVQNISYAFNMYFQIQDQDVRACD

AT5G20560 -----------------------------------------------------------------

OsGHL17_1 ALIAIAS----------------------------------------------------------

OsGHL17_2 LLSSFSC----------------------------------------------------------

OsGHL17_3 --------------------------------------------SVVFALS--------------

OsGHL17_4 ----------------------------------------------------------ATLLSCT

OsGHL17_5 ------------------------------------------VILSVFIF---------------

OsGHL17_6 NGLNWAC--GPGNVDCS------AIQP-SQPCY----QPDTLASHASYAFNSYYQQNGANDVACD

OsGHL17_7 GQQPQVT----------------PLQPGSAAAA----GPSALCAPGTATTTA----RGAAAAACS

OsGHL17_8 AALDWAC--GPGKVDCS------ALMQ-GQPCY----DPDNVEAHATYAFNAYYHGMGMGSGTCY

OsGHL17_9 QSLDWAC--GPGSANCS------AIQP-GQPCY----KSDDIVAVASYAFNDYYHRTRASGGTCN

OsGHL17_10 AAMDWAC--GPGRTDCT------AIQP-GQGCY----EPNDVRSHASFAFDSYYQSQGKAAGSCY

OsGHL17_11 DNVGYAC----SHSDCT------ALGY-GCSC-----GALDARGNASYAFNVYYQAQGQADAACD

OsGHL17_12 GNIQYAC----ASGDCT------ALGY-GCSC-----NGLDEKSNISYAFNMYFQMQDQDVRACD

OsGHL17_13 DNINFAC----TYADCT------PLGF-GSSC-----NGMDTNGNASYAFNAYFQAQSQKEEACN

OsGHL17_14 GAVGYAC----QYADCT------SLGA-GSSC-----GGLDARGNVSYAFNQFFQAANQMKGSCN

OsGHL17_15 DNVNYAC----TYADCT------SLGY-GSTC-----NGMDAAGNASYAFNAYFQVQNQVEESCG

OsGHL17_16 AAVNYAC----SNADCT------PLGY-GSSC-----NNLSHDGNISYAFNIYFQTMDQDVRACS

OsGHL17_17 NHLKLAC----SMADCT------TLDY-GGSC-----YGIGEKANVSYAFNSYYQQQKQDAKSCD

OsGHL17_18 NHLKLAC----TMADCT------TLYY-GGSC-----NAIGEKGNISYAFNSYYQLRKQDAQSCD

OsGHL17_19 DGVSYAC----GLADCT------SLGY-KTSC-----GGLDAKGNVSYAFNSYYQVNDQDDRACD

OsGHL17_20 DSISYAC----SLADCT------SLGY-KTSC-----GGLDTRSNISYAFNSYYQKNDQDDVACG

OsGHL17_21 DNINFAC----TFADCT------ALGY-GSTC-----AGMDANGNASYAFNAYFQVQNQKDDACD

OsGHL17_22 ADLDYAC--AQVGVDCG------AIQA-GGACF----EPNTVRAHAAYAMNQLYQAAGRHPWNCD

OsGHL17_23 --------------------------------------PGTTQGQQVPQTSSCYIPAGAVSRRAD

OsGHL17_24 SSFSAAC----SNADCT------ALSP-GGSC-----SGIGWPGNVSYAFNNFYQQHDQSEEGCS

KfGHL17_1 FNNTAVLTRV--DPSPNSSCQYPSS----------------------------------------

KfGHL17_2 FGG-TAQLVT--EDPSYGNCSFTGGV---------------------------GLPGYGVPGTVR

PmGHL17_1 -----------------------------------------------------------------

CgGHL17_1 F?V?LE-----------------------------------------------------------

NtGHL17_1 FEN-MFEVTT--TDPSTDTCRQIGKPVTNVGAEGNRNVTQSVVGGGGGGGGRGGENGSSPRPPAP

NtGHL17_2 INGRKGIITE--ENPSMGNCTFKLAL--------------------------DPSMLDNAANQLI

NtGHL17_3 WHGNDWAFACDFDDHDLSSAPSTGAQCGLTCFQTSGCTHFTWTQMNGGTCWMKSGDVSKADAFAT

PpGHL17_1 FGG-TGVITI--TDPSTS----------------------------------------------L

PpGHL17_2 FGG-TSVITI--TDPSYSGCRFH------------------------------------------

PpGHL17_3 -----------------------------------------------------------------

PpGHL17_4 -----------------------------------------------------------------

PpGHL17_5 -----------------------------------------------------------------

PpGHL17_6 -----------------------------------------------------------------

PpGHL17_7 FQG-TATLTT--KDPSYTACVYPSNT------------------------------------HLA

PpGHL17_8 FGG-DAELTY--VDPSYGSCYYVPSG---------------------------------------

PpGHL17_9 -----------------------------------------------------------------

PpGHL17_10 FQG-LGRITT--ENPSVGNCRFIIGL----------------------------------TKYTP

PpGHL17_11 -----------------------------------------------------------------

PpGHL17_12 FQG-YAMIVT--ENPSQGACRFPISL--------------------------------------V

PpGHL17_13 FQG-LASITT--VDPSAGNCRFIIGL----------------------------VKSKPSTSRSD

PpGHL17_14 FQG-LAQVVT--TDPSVGSCKFVIGV--------------------------------------A

PpGHL17_15 FQS-LAQVVN--TDPSVGTCKFLIGV--------------------------------------R

PpGHL17_16 FQG-LAQVTT--TDPSSGTCKFTIVV--------------------------------------C

PpGHL17_17 -----------------------------------------------------------------

PpGHL17_18 FGG-AATLTT--TDPSHDTCKFPTS----------------------------------------

CaGHL17_1 FN---------------------------------------------------------------

AcGHL17_1 K----------------------------------------------------------------

AfgHL17_1 KH---------------------------------------------------------------

AnGHL17_1 -----------------------------------------------------------------

CglGHL17_1 FN---------------------------------------------------------------

DhGHL17_1 F----------------------------------------------------------------

AgGHL17_1 -----------------------------------------------------------------

FgGHL17_1 -----------------------------------------------------------------

KlGHL17_1 FSS--------------------------------------------------------------

ScGHL17_1 FS---------------------------------------------------------------

SsGHL17_1 FG---------------------------------------------------------------

SpoGHL17_1 SE---------------------------------------------------------------

YlGHL17_1 FD---------------------------------------------------------------

AT3G57270.1 -----------------------------------------------------------------

AT3G57260.1 -----------------------------------------------------------------

AT3G57240.1 -----------------------------------------------------------------

AT4G16260.1 -----------------------------------------------------------------

AT5G56590.1 FGG-AGVKVN--KDPSYDKCIYITAG------------------------GNKTKATNATALTSS

AT1G77790.1 WR---------------------------------------------------------------

AT1G77780.1 Y----------------------------------------------------------------

AT4G26830.1 FGG-AAHVVT--QPPRYGKCEFPTGH---------------------------------------

AT4G29360 FNGASVEVD---KDPSYGNCLYMIAP--------------ATDGFNRTMAGNITGNITAIDSPLA

AT3G07320 FNG-LATQTI--KDPSYGRCEFPSVTL--------------------------------------

AT2G16230 FSK-TARITS--ENPSYSSCVYPRAG-------------------DGSITGEVTKYVTSDKATEK

AT5G20340 F----------------------------------------------------------------

AT2G05790 FGGAAYVVS---QPPKYGRCEFPTGY---------------------------------------

AT5G55180.1 FGGAAYVVSQ------------------------------------------------------P

AT5G20330 F----------------------------------------------------------------

AT2G01630 FKG-VATVTT--TDPSRGTCVFPGSA----------------KSNQTLGNNTSALAPSANSTTSG

AT4G34480 FSK-TATVTS--QNPSYNNCVYPGGG-------------------GGGGGGGGGSKAVMNKYVSS

AT1G66250 FNG-VASITT--TDPSHGTCVFAGSR--------------------GNGRNGTSVNITAPSANST

AT5G20390 F----------------------------------------------------------------

AT3G61810 -----------------------------------------------------------------

AT1G32860 LSLVVSMLLARHLL---------------------------------------------------

AT2G27500 -----------------------------------------------------------------

AT1G33220 F----------------------------------------------------------------

AT3G15800 -----------------------------------------------------------------

AT3G23770 FNG-LARETT--TNPGNERCKFPSVTL--------------------------------------

AT5G42720 VHNSTPRSAS-------------------------------------------------------

AT2G26600 -------------------SAFLLMI---------------------------------------

AT4G14080.1 FNG-LAHETT--TNPGNDRCKFPSVTL--------------------------------------

AT3G46570 -----------------------------------------------------------------

AT4G18340 -----------------------------------------------------------------

AT1G30080 -----------------------------------------------------------------

AT5G42100 FFFLLCIIKLRL-----------------------------------------------------

AT5G58090 FPN-ISEVTK--TDPSTGTCRFPIMI---------------------------------------

AT1G11820 FKG-VAMITT--TDPSHGSCIFPGSK------------------------KVGNRTQTVVNSTEV

AT3G24330 FLG-LGLITD--EDPSDELCEFPVMI------------------------------DTGDSTRLQ

AT4G31140.1 FPG-LSIVST--RDPSVGSCKFKIMI----------------------------------KSEDA

AT4G17180 -----------------------------------------------------------------

AT3G13560 FDG-TAITTT--RDPSYRTCAYTGSL--------------------NANATNGNFPPDALGPASP

AT5G24318 FDN-TGEITT--IDPSYGNCEYQAN----------------------------------------

AT2G39640 FSG-TGIVTS--TNPSTSTCPIPIGEGGGGNGAKSKSANWCMAKQEATETQLQANIDWVCSQGID

AT2G19440 FQG-LATITT--KNISQGQCNFPIQI---------------------------------------

AT3G04010 FEG-LATITT--QNISQGQCNFPIQI-------------------------------------GE

AT5G18220 FEG-LAIITT--RNISREQCNFPIQI---------------------------------------

AT1G64760 FQG-LATITT--QNISQGQCNFPIQI---------------------------------------

AT5G58480 FGG-LGLITT--VDPSEDNCRFSIQL-----------------------------------DTSH

AT3G55430 FSG-TGMIVG--NNPSNGACKY-------------------------------------------

AT5G20870 FNN-LGVVTK--IDPSSGSCRFPIEI-------------------------------DTSRQQMS

AT3G55780 --G-NGILGL--THRNYRS----------------------------------------------

AT5G64790 FKG-AAMITK--VNASVGSCLFPVQI------------------------------------VSG

AT5G20560 -----------------------------------------------------------------

OsGHL17_1 ----ITLILS-------------------------------------------------------

OsGHL17_2 -----------------------------------------------------------------

OsGHL17_3 -----------------------------------------------------------------

OsGHL17_4 F---IFLVLS-------------------------------------------------------

OsGHL17_5 ----LALVT--------------------------------------------------------

OsGHL17_6 FGG-TGVRTT--KDPSYDTCVYMAAG---------------------------SKMSTTNSSNLP

OsGHL17_7 SPSAAESPRTMSVISIIAGVLLMYLL---------------------I-----------------

OsGHL17_8 FSG-VAVITT--TDPSHGSCVYAGSG-----------------GKNGTSLLNGTSLAPSSNSTAG

OsGHL17_9 FNS-TAMVTS--TDPSHGSCIFAGST----------------------------GSNGSNGGAAS

OsGHL17_10 FQG-VGMVTT--TDPSHDSCIFPGSKLLSNVTKS----------DGANTTTAQTSDAEGSAIWRL

OsGHL17_11 FQG-LAVVTEDDRDVAQGACNFSVQV---------------------------------------

OsGHL17_12 FDG-LAKITT--KNASARGCAFPIQI------------------------------------ISA

OsGHL17_13 FQG-LAVPTE--TDPTTAQCNFTIQI---------------------------------------

OsGHL17_14 FNN-LSMITT--SDPSQGTCRFQIEI-------------------------------DTGRHDLA

OsGHL17_15 FQG-LAVQTQ--QDPSTNACNFTIQI------------------------------------EPS

OsGHL17_16 FEG-LAKITT--INASQGGCLFPVQI---------------------------------------

OsGHL17_17 FDG-NGMITY--LDPSMGECRFLVGI-------------------------------DDSKSSAV

OsGHL17_18 FDG-LGMITY--LDPSIGDCRFLVGI-------------------------------DDSRTSTI

OsGHL17_19 FKG-IATTTT--VDPSAGSCRFIIEI-----------------------------------APTA

OsGHL17_20 FSN-LATITG--QDPSTGTCRFGIMI------------------------------------EVD

OsGHL17_21 FQG-LAMPTQ--TDPSTPACNFTIQI---------------------------------------

OsGHL17_22 FRS-SATLTS--DNPSYGSCVYTGGQ*--------------------------------------

OsGHL17_23 AAV--------------------------------------------------------------

OsGHL17_24 FNG-LGLITT--VDPSVDNCMFNLAI---------------------------------------

KfGHL17_1 ----------------------------------------------------------------

KfGHL17_2 SAGGEGRVGFALRVAVTCLLSALLGHGLV-----------------------------------

PmGHL17_1 ----------------------------------------------------------------

CgGHL17_1 ----------------------------------------------------------------

NtGHL17_1 PRNESPDGGVVTRGRRGGG---------------------------------------------

NtGHL17_2 SIKHSSGMHALWTTIAFALTLSFLLLVVVN----------------------------------

NtGHL17_3 DDQSSVCGVVNRGEVEGEVGELIKLAIE------------------------------------

PpGHL17_1 KIRTFRQFPCEEVDIVG-----------------------------------------------

PpGHL17_2 ----------------------------------------------------------------

PpGHL17_3 ----------------------------------------------------------------

PpGHL17_4 ----------------------------------------------------------------

PpGHL17_5 ----------------------------------------------------------------

PpGHL17_6 ----------------------------------------------------------------

PpGHL17_7 SGQRSAAKYLAPKVIILLSSAALALCFSTR----------------------------------

PpGHL17_8 -ATRVVKRTLFRSLMMFVVALSLALVTV------------------------------------

PpGHL17_9 ----------------------------------------------------------------

PpGHL17_10 QTRPTSGTAAQSGILVLTIFSLLVSSFRFWLDSAL-----------------------------

PpGHL17_11 ----------------------------------------------------------------

PpGHL17_12 PTTGSSSGAPSWRLSSAALTLALVLSSLGVLWPIT-----------------------------

PpGHL17_13 AGSAFRPKNYNNGIIVAISALFLSNLFCFS----------------------------------

PpGHL17_14 ESSTSTPSAPGSAGMI------------------------------------------------

PpGHL17_15 PGNSQAIAPPESSSSRSDGLFLLFVIHTYL----------------------------------

PpGHL17_16 P---------------------------------------------------------------

PpGHL17_17 ----------------------------------------------------------------

PpGHL17_18 ----------------------------------------------------------------

CaGHL17_1 ----------------------------------------------------------------

AcGHL17_1 ----------------------------------------------------------------

AfgHL17_1 ----------------------------------------------------------------

AnGHL17_1 ----------------------------------------------------------------

CglGHL17_1 ----------------------------------------------------------------

DhGHL17_1 ----------------------------------------------------------------

AgGHL17_1 ----------------------------------------------------------------

FgGHL17_1 ----------------------------------------------------------------

KlGHL17_1 ----------------------------------------------------------------

ScGHL17_1 ----------------------------------------------------------------

SsGHL17_1 ----------------------------------------------------------------

SpoGHL17_1 ----------------------------------------------------------------

YlGHL17_1 ----------------------------------------------------------------

AT3G57270.1 ----------------------------------------------------------------

AT3G57260.1 ----------------------------------------------------------------

AT3G57240.1 ----------------------------------------------------------------

AT4G16260.1 ----------------------------------------------------------------

AT5G56590.1 ASTPRGNELLQWILKLCLMISLFFSLQTMNSQAL------------------------------

AT1G77790.1 ----------------------------------------------------------------

AT1G77780.1 ----------------------------------------------------------------

AT4G26830.1 ----------------------------------------------------------------

AT4G29360 SPSSTNEAFRQMVVAVSVLLPCFVVCSSIW----------------------------------

AT3G07320 ----------------------------------------------------------------

AT2G16230 NGSECFSSLYLARFIISIYFFCLFPSLRIM----------------------------------

AT5G20340 ----------------------------------------------------------------

AT2G05790 ----------------------------------------------------------------

AT5G55180.1 PSKVWEMRVSNRALKWI-----------------------------------------------

AT5G20330 ----------------------------------------------------------------

AT2G01630 CIPKYYHHPHASFGDLTLLSLLLIIALVFL----------------------------------

AT4G34480 DKVEKKNGATEPKVSSSLSFLLIFLSLIFHVYM-------------------------------

AT1G66250 TSSGIRSDLYYSRGIWSILTVMILNVANIL----------------------------------

AT5G20390 ----------------------------------------------------------------

AT3G61810 ----------------------------------------------------------------

AT1G32860 ----------------------------------------------------------------

AT2G27500 ----------------------------------------------------------------

AT1G33220 ----------------------------------------------------------------

AT3G15800 ----------------------------------------------------------------

AT3G23770 ----------------------------------------------------------------

AT5G42720 LAHICRSLSISASMFFVSVLYALIILL-------------------------------------

AT2G26600 ----------------------------------------------------------------

AT4G14080.1 ----------------------------------------------------------------

AT3G46570 ----------------------------------------------------------------

AT4G18340 ----------------------------------------------------------------

AT1G30080 ----------------------------------------------------------------

AT5G42100 ----------------------------------------------------------------

AT5G58090 -EPYYGGAAREHGFFFPLLMVAAIAVSIF-----------------------------------

AT1G11820 AAGEATSRSLSRGFCVTIMILVTFSIL-------------------------------------

AT3G24330 PGSSRVLTRVAAAVLVMLVLPIL-----------------------------------------

AT4G31140.1 SEASAMMPITRSTAVLLLLSICLYIVL-------------------------------------

AT4G17180 ----------------------------------------------------------------

AT3G13560 LGGNANARIIFSYHLPILAPLALTLLQLLLQHDRLL----------------------------

AT5G24318 ----------------------------------------------------------------

AT2G39640 CKPISPGGICFDNNNMKTRSTFIMNAYYESKGYSKDACDFRGSGIVTTTNPSTSTCVVPASVTL

AT2G19440 VASTASSFSSSLVLLIAGVWFLLSGVMFEV----------------------------------

AT3G04010 PSSGHYDYSYGSMVRLCLVMSGLVFLLI------------------------------------

AT5G18220 -GDPTSGHSDHNHRFISFSLVSMLLLFTAL----------------------------------

AT1G64760 VASSASSFSCSSYSLVVLIVWFLLSGMMF-----------------------------------

AT5G58480 SSSQTPNFFQSWPLLLLFLLSGLF----------------------------------------

AT3G55430 ----------------------------------------------------------------

AT5G20870 NPPPRNSGASEAKYRLGAAVVMVICLVFSN----------------------------------

AT3G55780 ----------------------------------------------------------------

AT5G64790 SDDFRINFVFGRFVVFGLVLLGLLTVI-------------------------------------

AT5G20560 ----------------------------------------------------------------

OsGHL17_1 ----------------------------------------------------------------

OsGHL17_2 ----------------------------------------------------------------

OsGHL17_3 ----------------------------------------------------------------

OsGHL17_4 ----------------------------------------------------------------

OsGHL17_5 ----------------------------------------------------------------

OsGHL17_6 VQSGFSPSRFDKNFYLLFSMLPIMIAACIV----------------------------------

OsGHL17_7 ----------------------------------------------------------------

OsGHL17_8 DSGAHRAIGDVSSFVRAVVAALLLSVVLLL----------------------------------

OsGHL17_9 GPVSPDNFATQIHSCWLTHLITLLSIMVFM----------------------------------

OsGHL17_10 RTGRETGFLFILRWLLSLSVVLITTNSNFWT---------------------------------

OsGHL17_11 AAAALVGAAVAAAAAVACAAAVVAALLALV----------------------------------

OsGHL17_12 AAPAVAGVGLSAAALLALLMVLV-----------------------------------------

OsGHL17_13 --KSSAAAAAAAPVAAGVVVAALAQLLLLW----------------------------------

OsGHL17_14 VASSASAAATSVAAVLLLALLGLVI---------------------------------------

OsGHL17_15 AAAGRRPAAVAVTVATAMLISVLAAMVTTP----------------------------------

OsGHL17_16 --LSASERVVPLRFLPTSFLILLMVVSILT----------------------------------

OsGHL17_17 SSCGCGCGVCCGVWVLFFWVFMYLRMMGSV----------------------------------

OsGHL17_18 YSSGDCSRRRFGLWILAFLVIGFLQIGVYL----------------------------------

OsGHL17_19 NGVAMAATVRVAGVMAAILAVFIHLVVPVF----------------------------------

OsGHL17_20 SAFSWKLQHVRSSNLLMLLLVLLQLCLSFS----------------------------------

OsGHL17_21 --AATSAGHRRRAGAAVLALLALFRLFLLH----------------------------------

OsGHL17_22 ----------------------------------------------------------------

OsGHL17_23 -----------RRLVWLGVLLCLVTLVRK*----------------------------------

OsGHL17_24 --DTSTAASFHPILAMLQILVLFFCTYNLL*---------------------------------
