# Supplementary material for: A phylogenetic approach to study the origin and evolution of plasmodesmata-localized glycosyl hydrolases family 17
Source: Front Plant Sci. 2014 May 23;5:212. doi: 10.3389/fpls.2014.00212 (PMC4033164; doi:10.3389/fpls.2014.00212)
Supplement: Supplementary file 2 [file DataSheet2.DOCX]

Alignment of GHL17 sequences from Arabidopsis thaliana (At) and *Populus trichocarpa* (Pt).

PtGHL17_1 --------------------MVLGNLLLFIVGI---FSYA-------------------------

PtGHL17_2 --------------------MVPGRLLLLIVTT---LHYA-------------------------

PtGHL17_3 -------------------MALLLLLFLLMVPV---VSAD-------------------------

PtGHL17_4 ---------------------MDAYYNICIIFL--------------------------------

PtGHL17_5 ------------MALSKLPLSTIFFLLYTSLFTISQALSSKIAVAQIKDQQD-------------

PtGHL17_6 ---------------MARGVEQLIFLISLLLTL---LVLC-------------------------

PtGHL17_7 ------------MTSLSTFTFSFLLLSFSPSFS---FFYAF------------------------

PtGHL17_8 --------------MDPNFLPCFLLLLCVVAFA--------------------------------

PtGHL17_9 --------------MEPYFLSSFLVLVCIFTSA--------------------------------

PtGHL17_10 ----------MGKPLASFSSSIVSTILLLASAF---NILG-------------------------

PtGHL17_11 ------------------MAFSILIFLYLLQSF---NLAS-------------------------

PtGHL17_12 ------------------MAFSILILLYLLQSF---NFAS-------------------------

PtGHL17_13 -------------MATITTTSITTALLIISTLL---HFST-------------------------

PtGHL17_14 ------------MVAKTNLSHHRRLLLLVSTLL---HLFT-------------------------

PtGHL17_15 ---------------MGPVAAIHVFIFALLISI---SGAV-------------------------

PtGHL17_16 ---------------MAKPRTLFLLLLTFYYHF---SSSS-------------------------

PtGHL17_17 -----------MALLLSRIAMIHIFPILFLTFS---DNYG----------FLR------------

PtGHL17_18 --------------MEYCRFYYCASLILSISGL---VFPV-------------------------

PtGHL17_19 -----------MALFCIRIAIIHIFLLLFLTSS---DNYG----------FLR------------

PtGHL17_20 ------------------------------MHS--------------------------------

PtGHL17_21 --------------MEHSRFYYHVSLILSISGL---VFPV-------------------------

PtGHL17_22 ------------------MAPASLLVLLFLATL---SPFP-------------------------

PtGHL17_23 -------------MLAALFSLECEWLHFFTVYA--------------------------------

PtGHL17_24 ---------------MATYSVHLRLLFLLLALS---GVHV-------------------------

PtGHL17_25 -------------MKSHHHQLLFMFLVFFAQKIELLTAET-------------------------

PtGHL17_26 -------------MATCFVLSRLLFLLLTLSDS---AVRV-------------------------

PtGHL17_27 --------------MVVLPYTVAFLLLSSLQTV---KIAN-------------------------

PtGHL17_28 -------------MAVLPSAVAFLLLVSFSLTV---NIAN-------------------------

PtGHL17_29 --------------MVVFPYTVAFLLLSSLQTV---KIAN-------------------------

PtGHL17_30 --------------MVVLPYTVAFLLLSCLQTV---KIAN-------------------------

PtGHL17_31 --------------MVVLPYTVAFLLLSFLQTV---KIAN-------------------------

PtGHL17_32 -------------MTATTTTSITTALLHLSTLL---HLST-------------------------

PtGHL17_33 --------------MVVLPYTVAFLLLSFLQTV---KIAN-------------------------

PtGHL17_34 --------------MVVLPYTVAFLLLSFLQTV---KIAN-------------------------

PtGHL17_35 ------MGLVKGVALSTRLRQQPLGLALCCLYI---LQLL--------LLLLS------------

PtGHL17_36 -------------MSRLSGTLLCLVLAIILLSG---SFPV-------------------------

PtGHL17_37 --------------MVVLPYTVAFLLLSCLQTV---KIAN-------------------------

PtGHL17_38 -------------------------------MI--------------------------------

PtGHL17_39 --------------------MLLNLSLFLLFLT---ASNI-------------------------

PtGHL17_40 -------------------MGTCWFLRLVLILG---LLGS-------------------------

PtGHL17_41 ---------------MGLYHLAIGLVSLLSMVS--------------------------------

PtGHL17_42 MGGLIKGVVSTRQRQQPLVVTLRCLSILQLLVD--------------------------------

PtGHL17_43 --------MAKSSASHKTSPMISVVLLAGLLMS---CLRI-------------------------

PtGHL17_44 -----------------------------------------------------------------

PtGHL17_45 --------------MNTFMATTILLLFGLIISR---LTLS-------------------------

PtGHL17_46 ---------------MGRRQYLLCFFFLCLISS---DQGL-------------------------

PtGHL17_47 ----------MARTGLLVWLYCLILASVNVVHV---LSST-------------------------

PtGHL17_48 --------MARSNIAGKSPSMISIMLLFGLLLA---SLDT-------------------------

PtGHL17_49 ------MASFSSRSRTSSLTAAMLLLLGVLFMA---NLDM-------------------------

PtGHL17_50 -----------------------------------------------------------------

PtGHL17_51 --------MAVSHLAGKSPAMLSMMLLFGILLA---SLDT-------------------------

PtGHL17_52 -----------------MAAISLLSVGFLMSGL---EAVT-------------------------

PtGHL17_53 -------------MALAGVFTTYVLVPFVIFLT---MIPD-------------------------

PtGHL17_54 -------------MATCLVLLRLLFSLLVIWGS---AVQI-------------------------

AT3G57270.1 ---------------MDLRFLASLTLLLGLFFV---NTNP-------------------------

AT3G57260.1 ----------MSESRSLASPPMLMILLSLVIAS---FFNH-------------------------

AT3G57240.1 -------MKMCNGSSFLASLPLLLLLLSFILAS---FFDT-------------------------

AT4G16260.1 ------------------------MTTLFLLIA---LFIT-------TILNPT------------

AT5G56590.1 ----------------MARDFKLIFSISILLLL---LDCC-------------------------

AT1G77790.1 ------------MDSKLIRFAVVIMLLSIQIFC---TAGV-------------------------

AT1G77780.1 ------------------MFLIATLLFLSARLT---TAGN-------------------------

AT4G26830.1 -------------MAVSFLPYFLILSFLSAIDA--------------------------------

AT4G29360 ---------------MGQRLNLVFWIFVSILAF---LNFG-------------------------

AT3G07320 -----------------MSLLLHLFALSLLISV---SGAK-------------------------

AT2G16230 -----------------MALSILFLLLFILFSI---SPSN-------------------------

AT5G20340 ------------MLYLPKKLFLFFFSCIVVIVN---YNNSDFVNAANSIGFVN------------

AT2G05790 -------------MDLLTPLIFLLLLLSPFSLS--------------------------------

AT5G55180.1 ---------------MAVFVLSLLILSSFSAIP---FTYA-------------------------

AT5G20330 ------------MLYSPKKLFLFFLSCIVLYVN---SNNS---------GFVT------------

AT2G01630 -------------------MAALLLLFLFLFAS---SALS-------------------------

AT4G34480 ----------------MALSISIYFLLIFLSHF---PSSH-------------------------

AT1G66250 --------------MASLLHLLLLSLSLLVLAS---ASPS---------PPAD------------

AT5G20390 -------------MDCHRKTFLLKFLCVAFLLN---YSNV---------GFVD------------

AT3G61810 -----------MKMILMTTMQNAFWILLVFSIS----MAS-------CSTIPSLVGPQIGINYGE

AT1G32860 -------------MELTSFHRSSLLFLISLTLI---ILPT-------------------------

AT2G27500 -------------MATHSLSFFFRVLLLLFLTL---SERI-------------------------

AT1G33220 ------------MVYSSKKLFLFFLSCIMLTFN---YNTS---------GFVA------------

AT3G15800 ----MGYCVPLRKSPTSHMVFSSFFLSFLLVFS---ILSS-------QTAVA-------------

AT3G23770 ------------MTPFALFL------FTLLALS---SSCCSAIGPQNNR-----TVLA-------

AT5G42720 ----------------MRASVYSLILLFFSCLL---HLSK-------------------------

AT2G26600 ------------MRSSKLLLLKFFFFFFFNLTS---LEYQVDGA---------------------

AT4G14080.1 ------------------MSLLAFFLFTILVFS---SSCCSATRFQGHRYMQRKTMLD-------

AT3G46570 ---------------MLGSRVVHQLYFLILTLI---LPFS-------------------------

AT4G18340 ---------MTSRTFTRHSSLIHVFLLLSLVFS--GNILQ-------------------------

AT1G30080 -----------MSNMFSRIAMTNSIVLLLFSLT---FLEH-------GLLFQ-------------

AT5G42100 ---------------MASSSLQSLFSLFCLALF---SLPL-------------------------

AT5G58090 --------------------MGWGSVLLLLAVA---LLCQ-------------------------

AT1G11820 ---------MAFTSMVSTVPVLFFFFTLLLISA---NSSS--LSHNIKVQEQD------------

AT3G24330 ------MAGERSKLTTNHFYNHQIILCYFLIIS---QVSI----------ASS------------

AT4G31140.1 ---------------MLFKGVFAVFFVITLLYA---SLLI-------------------------

AT4G17180 -------------MGSGVGVALFALSLLLVSHE--------------------------------

AT3G13560 ----------------MLLPRWFAEALLLLLSI---LACS-------------------------

AT5G24318 ------------MNYVLPFFSLSMFTIVGVLLI---LSTG-------------------------

AT2G39640 ------------MAKTIRSFILPFLLIVAGVIF---QLSA-------------------------

AT2G19440 -----------------MNLLAFVVGFGIMGIV--------------------------------

AT3G04010 ---------MNYRRKQSAITNSAVVFIIISAVC---FLSG-------------------------

AT5G18220 ------------MSNRRKQSTTAGIFLCITIVS---LLSG-------------------------

AT1G64760 -----------------MSNLLALVVGFVIVIG---HLGI-------------------------

AT5G58480 ----------------MARRLFLLLLAVTAGLS---LTGT-------------------------

AT3G55430 -------------MAKAPPSISLLLLLCAAVFL---TIPA-------------------------

AT5G20870 --------------MSSHDTYQKLILFCLSIFF---LQNI-------------------------

AT3G55780 -----------MKKMHSLSSYLLLLISLTAIAT---PTTT-------------------------

AT5G64790 --------------MAGRAAMLVNVGTVIMTVL---TLAS-------------------------

AT5G20560 ------------MEDYSKNLFLLLFSCTALIIS---YYNV---------DSLS------------

PtGHL17_1 -------SGAFVGI--NLGT----DVSNMPSAPDVVSILKANQ-ITHLRLYDADAHMLKALADS-

PtGHL17_2 -------SGAFVGI--NIGT----DVSNMPSAPDVVAILKTNQ-ITHVRLYDADAHMLKALADS-

PtGHL17_3 -------EDAFIGV--NIGT----ALSDMPSPTQVVALLKAQN-IRHVRLYDADRAMLHALANT-

PtGHL17_4 -------AEAFIGV--NIGT----DLSDMPHPTQVVALLKAQQ-IRHVRLYDADRGMLVALANT-

PtGHL17_5 -------NEPFVGI--NIGV----DVSNLMSATELVSLLQFQK-VTHIRLFDADPDMLKALAKT-

PtGHL17_6 -------RGSTIGV--CYGR----NADDLPTPDKVAQLVQQHK-IKYLRIYDSSIQVLKAFANT-

PtGHL17_7 -------AAGTIGV--NYGR----VANNLPAPAEVVSLLKSHG-INRIKLYDTDSDVLTALAGS-

PtGHL17_8 -------DAGSVGV--NYGR----IANNLPSAVKVVNLVKSQG-LERVKVYDTDPAVLKALSGS-

PtGHL17_9 -------DAGSIGV--NYGR----IANNLPAAAKVVQLVKSQG-LERIKVYDTDPIVLKALSGC-

PtGHL17_10 -------AEGSIGV--NYGT----VADNLPPPAQVAHFLLESTIINRVRLFDTNTEIIQAFAHT-

PtGHL17_11 -------SESFIGV--NYGQ----VADNLPSPSATAKLLQSTA-VQKVRLYGADPAIIRALANT-

PtGHL17_12 -------SQSFIGI--NYGQ----VADNLPPPSATAKLLQSTG-VQKVRLYGADPAIIKALADT-

PtGHL17_13 -------TAFAIGV--NYGT----LANNLPSPSQVASFLKTQTTIDSIKIFDTNPDILRAFANS-

PtGHL17_14 -------TTFAIGV--NYGT----LGNNLPPPSQVANFIKTQTIIDSIKIFDTNHDILNAFANT-

PtGHL17_15 -------ISSEVGI--NYGQ----LGNNLPSPSRSVELIKSLK-AKRVKIYDANPDILKSLKNT-

PtGHL17_16 -------AAYTVGV--NYGT----VANNLPSPSQVASFLKTQTTIDRVKIFDTNPDILRAFANT-

PtGHL17_17 -------GINSLGI--NYGQ----VGNNLPQPENVLDLLISLK-LTKARIYDTNPQILTAFSNS-

PtGHL17_18 -------MVGSIGI--NYGQ----IANNLPAPDNVVPLVKSIG-ATKVKLYDADPRVLKAFANT-

PtGHL17_19 -------GVTSLGI--NYGQ----VGNNLPQPEKVLDLLSSLK-LTKARIYDTNPQVLTAFANS-

PtGHL17_20 -------TPGTYGV--NYGK----IADNLPSPSSVVTLLKAAK-IKNTRIYDADHEVLKAFKGS-

PtGHL17_21 -------MVGSIGI--NYGQ----IANNLPTPDNVIPLVKSIG-ATKVKLYDADPRVLKAFANT-

PtGHL17_22 -------NVSAVGV--NYGT----LGNNLPSPKKVAQLLQSTL-IDKVKIYDTNPEILGAFSNT-

PtGHL17_23 -------FKGTYGV--NYGR----IADNLPSPRSVVTLLKAAK-IKNTRIYDADHEVLKAFKGS-

PtGHL17_24 -------LGAGFGI--NYGQ----IADNLPSPSRVSVMLQSLD-VSRLKLYDTDPKVLQAFSNS-

PtGHL17_25 -------FTGTYGI--NYGR----IADNIPSPDEVATLLRAAK-IKNVRIYDADHSVLKAFSGT-

PtGHL17_26 -------LGAGLGI--NYGQ----IANNLPSPSRVAVMLQSLN-VSRLKLYDADPNVLLAFSNS-

PtGHL17_27 -------SQSFIGI--NYGQ----VADNLPPPPSTAKLLQSTS-IQKVRLYGSDPTIIKALANT-

PtGHL17_28 -------SQSFIGV--NYGQ----VADNLPPPSATAKLLQSTS-IQKVRLYGSDPAIIKALANT-

PtGHL17_29 -------SQSFIGI--NYGQ----VADNLPPPPSTAKLLQSTS-IQKVRLYGSDPAIIKALANT-

PtGHL17_30 -------SQSFLGI--NYGQ----VADNLPPPSSNAKLLQSTS-IQKVRLYGSDPAIIKALANT-

PtGHL17_31 -------SQSFIGI--NYGQ----VADNLPPPPSTAKLLQSTS-IQKVRLNGSDPAIIKALANT-

PtGHL17_32 -------TAFSIGV--NYGT----LANDLPSPSQVANFLKTQTIIDSIKIFDTNPDILRGFANT-

PtGHL17_33 -------SQSFIGI--NYGQ----VADNLPPPSSTAKLLQSTS-IQMVRLYGSDPAIIKALANT-

PtGHL17_34 -------SQSFIGI--NYGQ----VADNLPSPSSTAKLLQSTS-IQKVRLYGSDTAIIKALANT-

PtGHL17_35 -------SVSGTGV--NWGT----QATHPLPPSTVVNMLRDNG-IQKVKLFDADSATLNAFANS-

PtGHL17_36 -------GESAIGV--NWGT----ISFHRLKPSTVVDLLKDNK-IKRVKLFDADPGSLTALMGS-

PtGHL17_37 -------SQSFISI--NYGQ----VADNLPPPPSTAKLLQSTS-IQKVRLYGSDPAIIKALANT-

PtGHL17_38 -------IAGAIGI--NYGL----NGDNLPAPPAVVGLYERCH-IPSVRLFEPRPEVLQALRGK-

PtGHL17_39 -------TVQAIGV--NWGT----TSSHPLPPDKVVELLKSNK-ITKVKLFDADPLVLQALSGS-

PtGHL17_40 -------CVHGLGV--NWGT----MAIRKLSPETVVQMLKDNG-ILKVKLFDADQTTMTALAGS-

PtGHL17_41 -------GAMTIGA--NWGT----QASHPLPPETVVRLLRENG-IQKVKLFDADYDTLKVLGKT-

PtGHL17_42 -------SVSGIGV--NWGT----QATHPLPPSTVVKMLRDNG-FQKVKLFDADSATLNALANS-

PtGHL17_43 -------TGAQMGV--CYGT----LGDNLPSEQEVVDLFNQYN-IRRMRIYDPNPRTLQALGGS-

PtGHL17_44 ------------------------MSD--------------------------------------

PtGHL17_45 -------DAQSIGV--CYGK----NGNNLPSDQEVVSLFQTNV-IGRMRIYDPNRDTLEALRGS-

PtGHL17_46 -------VKGVVGLACNWGT----QSTHPLQANIAVKLLKENG-FKKVKLFEADPAALKALGKS-

PtGHL17_47 -------VLPGIGV--NWGT----MASNPLPRNIVVNMLKDNG-ITKVKLFDSDSPTLKALAGT-

PtGHL17_48 -------TGAQIGV--CYG-----MNGNLPPAQEVIDLYNQRG-IRRMRLYDPNQDALRALGGT-

PtGHL17_49 -------TGAQVGI--CYGM----MGNNLPPATEVIALYKQHN-IKRMRLYDPNQAALNALRDS-

PtGHL17_50 -------MNHAISL---------------------------------VRVYDANTEVLEALSGT-

PtGHL17_51 -------AGAQIGV--CYG-----MLGNLPPRPEVIALYNERG-IQRMRLYDPDQDALRALGGT-

PtGHL17_52 -------GAQSLGV--CNGR----LGNDLPSEQEVVDFYKSNG-IGRMRIYDPNQETLQAIRET-

PtGHL17_53 -------VTSRVGV--NWGT----MATHQLAPDNVVQMLKENG-FDKLKLFDADEKIMAALIGT-

PtGHL17_54 -------HGVELGI--NYGQ----IANDLPSPTLAAVLLQSLN-VHRVKLFDADLNVLIAFSNS-

AT3G57270.1 -------TGGQVGV--CYGR----NGNNLPSPAETIALFKQKN-IQRVRLYSPDHDVLAALRGS-

AT3G57260.1 -------TAGQIGV--CYGM----LGDTLPSPSDVVALYKQQN-IQRMRLYGPDPGALAALRGS-

AT3G57240.1 -------AVGQIGV--CYGR----NGNNLRPASEVVALYQQRN-IRRMRLYDPNQETLNALRGS-

AT4G16260.1 -------SGESVGV--CYGM----MGNNLPSQSDTIALFRQNN-IRRVRLYDPNQAALNALRNT-

AT5G56590.1 -------YGGKVGV--CYGR----SADDLPTPSKVVQLIQQHN-IKYVRIYDYNSQVLKAFGNT-

AT1G77790.1 -------AGDITGD--CYGR----NGNNLPTPADTVALYKSNN-IDAIRMYEPFADMLEALRGS-

AT1G77780.1 -------MNSFAGV--CYGR----NGDNLPSPAKTVSLYKKIN-VGGIRLYEPFPDLIVSLQGT-

AT4G26830.1 -------HSGMVGV--NYGR----IANNLPSPEKVVNLLKSQG-INRIKIFDTDKNVLTALANS-

AT4G29360 -------MASKIGI--CYGR----NADNLPSPNRVSELIQHLN-IKFVRIYDANIDVLKAFANT-

AT3G07320 -------FSGRPGI--NYGQ----LGNNLPSPSDSVNLIKSLN-AKRVKLYDANPKILAALNGT-

AT2G16230 -------AQSFIGV--NYGL----LSDNLPPPSQTAKLLQSTS-IQKVRLYNADSSIITSLVGT-

AT5G20340 -------AANSIGL--NYGL----LGDNLPSPSKVITLYKSID-ITKIRIFDPNTEVLNALRGHR

AT2G05790 -------DAGSIGV--NYGR----ISDELPSAFKVVQLLKSQG-ITRVKIFDADPSVLKALSGS-

AT5G55180.1 -------DSGMIGV--NYGR----IADNLPAPEKVVELLKTQG-INRIKLYDTETTVLTALANS-

AT5G20330 -------AANSIGL--NYGL----LGDNLPSPSNVINLYKSIG-ISRIRIFDPNTEVLNALRGHR

AT2G01630 -------QDSLIGV--NIGT----EVTNMPSPTQVVALLKSQN-INRVRLYDADRSMLLAFAHT-

AT4G34480 -------AEPFIGV--NYGQ----VADNLPPPSETVKLLQSTS-IQKVRLYGADPAIIKALAGT-

AT1G66250 -------EGSYIGV--NIGT----DLSDMPHPTQVVALLKAQE-IRHIRLYNADPGLLIALANT-

AT5G20390 -------AATNIGL--NYGL----LGDNLPPPSEVINLYKSLS-VTNIRIFDTTTDVLNAFRGNR

AT3G61810 YSSNLEDSSPVIGI--NYGR----YGSNLPPPEAIPSLVNSLS-IKHVKTFDLDPRITKSFANT-

AT1G32860 -------TTTSIGV--NYGQ----IGDNLPSPTDVIPLIKSIG-ATKVKLYDANPQILKAFSNT-

AT2G27500 -------KGQGVGI--NYGQ----IANNLPSPARVAVLLRSLN-ITRVKLYDADPNVLFSFSNS-

AT1G33220 -------AANSIGL--NYGL----LGDNFPTPSNVLNLYKSIG-ITKIRIFDPKTEVLNALRGHR

AT3G15800 -------FIGTYGV--NYGR----IADNLPSPDAVATLLKSAK-IRNTRIYDADHSVLTAFRGT-

AT3G23770 -------LASRIGI--NYGK----LGNNLPFPYQSINLIKTIK-AGHVKLYDADPETLKLLSTT-

AT5G42720 -------SQPFLGV--NYGL----TADNLPPPSASAKLLQSTT-FQKVRLYGSDPAVIKALANT-

AT2G26600 -------FVGTYGI--NYGR----IADNIPSPEKVVLLLKQAK-IRNVRIYDVDHTVLEAFSGT-

AT4G14080.1 -------LASKIGI--NYGR----RGNNLPSPYQSINFIKSIK-AGHVKLYDADPESLTLLSQT-

AT3G46570 -------AAYQVGV--NYGT----VANNLPPPHQVVNFIKTKTIINHVKIFDTNHDILAAFSGIT

AT4G18340 -------TVTSLGI--NYGQ----VGNNLPSPDKVINLLRSLR-ITKTRIYDTNPQILSAFANS-

AT1G30080 -------RVSSLGI--NYGQ----VGDNLPPPDKVLQLLSSLH-INKTRIYDTNPRVLTSFANS-

AT5G42100 -------IVSSIGI--NYGQ----VANNLPPPKNVIPLLKSVG-ATKVKLYDADPQALRAFAGS-

AT5G58090 -------RASSIGA--NWGT----QASHPLPPDIVVRMLRENG-IQKVKLFDAEYDTLRALGKS-

AT1G11820 -------KDPFVGF--NIGT----DVSNLLSPTELVKFLQAQK-VNHVRLYDADPELLKALAKT-

AT3G24330 -------NTSNVGV--NWGI----MASHQLPPEKVVKMLMDNS-FTKLKLFEADQNILDALIGS-

AT4G31140.1 -------EVEGIGV--NWGS----QARHPLPPATVVRLLRENG-IQKVKLFEADSAILKALSRT-

AT4G17180 -------VESAIGV--NWGT----LSFHKMRPSTVVDLLKANK-ITKVKLFDANPDALRALMGT-

AT3G13560 -------NAAFIGV--NIGT----DLTNMPPPSDIVTLLKSQQ-ITHVRLYDANSHMLKAFANT-

AT5G24318 -------SEASIGV--NYGT----LANNLPPPQQVAEFLLHSTVINRIRLFDTDPQILQAFANT-

AT2G39640 -------VTSAIGI--NYGT----LG-NLQPPQQVVDFIKTKTTFDSVKIYDANPDILRAFAGS-

AT2G19440 -------MVDGLGV--NWGT----MATHKLPPKKVVQMLKDNN-INKVKLFDADETTMSALSGS-

AT3G04010 -------GVSGLGV--NWGT----MASHQLPPKTVVEMLKDNN-IQKVKLFDADTNTMGALAGS-

AT5G18220 -------DVSALGV--NWGT----MSTHQLPPKTVVQMLKDNN-VKKVKLFDADTNTMVALAGS-

AT1G64760 -------LVNGLGV--NWGT----MATHKLPPKTVVQMLKDNN-INKVKLFDADETTMGALAGS-

AT5G58480 -------TVRAVGI--NWGT----EASHPLPPSKVVELLKSNG-IVKVKLFDADPKVLRALSGS-

AT3G55430 -------VISAIGV--NYGT----LG-NLPPPTQVANFIKTQTSIDSVKIFDVNPDILRAFAGT-

AT5G20870 -------LKNVEGLACNWGT----QASHPLPPNIVVKLLRDNG-FNKVKLFEADPGALRALGKS-

AT3G55780 -------SATTIGV--TYSTPASISGTVQLSPDRIAEKVVSMN-IPAVRLLDSNPAMIRAFAYT-

AT5G64790 -------LVGGFGV--NWGN----IASHPLNPNIVVQMLKDNK-INKVKLFDADSWTMNALAGT-

AT5G20560 -------TASVVGL--NYGL----LGDNLPSPSNVIKFYKSQN-VAKIRIFEPNKDVLNALRGNR

PtGHL17_1 GIEVMVGVTNEEVLGIGESPSKAAAWINQNVAAY-LP-S--TSITAIAVGSEVLTSIP-----NL

PtGHL17_2 GIEVMVGVTNEEILGIGESASKAAAWINQNVAAY-LP-S--TNITAIAVGSEVLTTIP-----NV

PtGHL17_3 GIRVTVSVPNEQLLGIGQSNATAANWVARNVIAH-VP-A--TNITAIAVGSEILTTLP-----NA

PtGHL17_4 GIQVMVSVPNEQLLGIGQSNSTAANWVSHNVVAH-YP-A--TNITAICVGSEVFTAVP-----NA

PtGHL17_5 KIRVIISVPNNQLLAIGSSNATAASWIGKNVVAY-YP-Q--TVISAIAVGDEVLTTVP-----SS

PtGHL17_6 GVELMIGITNSDLLPFSQFQSNVDTWLKNSVLPY-YP-A--ARITCITVGAEVTESPG-----NA

PtGHL17_7 SINVVVALPNELLSSVAADQSFADSWVKGNISQH-FP-Q--TKIEAIAVGNEVFVDPK-----NT

PtGHL17_8 GIKVTVDLPNQLLYSAAKYPNFARSWVQKNIVAY-HP-S--TQIESIAVGNEVFVDPH-----NT

PtGHL17_9 GIKVTVDLPNELLYSAAKNPYFARTWVQKNVVAY-HP-S--TQIEAIAVGNEVFVDPH-----NT

PtGHL17_10 GIAITITVPNDQIPHL-TNLGFAQEWVKSNVQPY-VP-A--TNIVRILVGNEVISIENK----LS

PtGHL17_11 GIEIVIGAANGEIPALASDPNSATQWINSNVLPY-YP-A--SKIILITVGNEVLLSNDQ----NL

PtGHL17_12 GIGIVIGAANGDIPALASDPNFATQWVNSNVLAY--P-S--SKIILITVGNEVLLSNDQ----NL

PtGHL17_13 NITVTVTVGNGDIPAL-VDVNAASQWVANNIKPY-YP-Q--TRIKLIAVGNEILFTGNK----EW

PtGHL17_14 GITVTVTVGNGDIPSL-ANLNSARGWVAANIAPF-HP-Q--TRINRIVVGNEIMATANK----PW

PtGHL17_15 DIQVSIMVPNALIPNISKSQYFSDQWVKTNVVPY-YS-D--VKIRYLLVGNEILTNPDT---GTW

PtGHL17_16 GISVTVTVGNGDIPSL-AKLPAAQSWIAANILPF-HP-Q--TSIKYIAVGNEVLATSDK----VL

PtGHL17_17 NVELIVTIENQMLAVL-MDPQQALQWVSTHIKPY-FP-A--TRITGIAVGNEVFTDDDT----TL

PtGHL17_18 GVEFIVGLGNEYLSKM-RDPEKAQAWVKTNVQAY-LP-A--TKITCITIGNEVLTFNDT----GL

PtGHL17_19 NVELIVTVENQMLAVL-MDPQQALQWVSNHIKPY-FP-A--TRITGIAVGNEVFTDSDT----TL

PtGHL17_20 GIEIVIGLGNEFLKEMSVGEDRAMDWIKENVQPF-LP-G--TKIVGIAVGNEILGGGDH----EL

PtGHL17_21 GVEFIVGLGNEYLSKM-RDPDKAQAWVKANVQAY-LP-A--TKITCITIGNEILTLNDT----SL

PtGHL17_22 GIDLIVAVENYHVANISTDTSAADEWLTNRVVPF-IP-A--TSIVAIAVGNEYLTTDPD----HL

PtGHL17_23 GIGIIVGLGNGYLKEIAVGEDRAMNWIKENVQPF-LP-G--TNIAGIAVGNEILGGDDH----EL

PtGHL17_24 SVEFIVGIGNEYLQDM-ADPVKAQNWVQQHLQPYLAQ----TKITCISVGNEVFMSNDT----QI

PtGHL17_25 GLQLVVGLPNGFVKEMSANASHAMAWVKENVQAF-LP-K--TSVCGIAVGNEILGGGDY----EL

PtGHL17_26 NVEFIIGLGNEYLQDM-TDPIKAQNWVQQHLQPHITQ----TKITCITVGNEVFMSNDT----RL

PtGHL17_27 GIGIVVGTANGDIPGLASGPNFAESWINTNVLPF-YP-A--SNIILITVGNEVMTSNDQ----NL

PtGHL17_28 GIGIVIGTANGDIPALASDPSFAKNWINTNVLPF-YP-A--SKIILINVGNEVMTSGDQ----NL

PtGHL17_29 GIGIVIGTANGDIPGLASDPNFAKSWINTNVLPF-YP-A--SNIILITVGNEVMTSNDQ----NL

PtGHL17_30 GIGIVIGTANGDIPGLASDSNFTKSWINKNVLPF-YP-A--SNIILITVGNEVMTSNDQ----NL

PtGHL17_31 GIGIVIGTANGDIPGLASDPNFAKSWINTNVLPF-YP-A--SNIILITVGNEVMTSNDQ----NL

PtGHL17_32 NITVTVTVGNGDIPAL-VDANAASRWVANNIEQF-YP-Q--TRIKLIAVGNEILFTGNK----EW

PtGHL17_33 GIGIVIGTANGDIPGLASDPNFAKSWINTNVLPF-YP-A--SNIILITVGNEVMTSNDQ----NL

PtGHL17_34 GIGIVLGTANGDIPGLASDPNFAKSWINTNVLPF-YP-A--SNIILITVGNGVMTSNDQ----NL

PtGHL17_35 GIQIMVGIPNDMLYTLANSEQAAENWVAKNVSSHISSGG--VDIRYVAVGNEPFLSTYN---GSF

PtGHL17_36 GIEVMVGIPNEMLAALSSSTDASDLWVRQNVSRYVVKGG--ADIRYVAVGNEPFLTSYS---GQF

PtGHL17_37 RIGIVIGTANGDIPGLASDPNFAKSWINTNVLPF-YP-A--SNIILITVGNEVMTSNDQ----NL

PtGHL17_38 PLQVILGTRNEDIQSLATTLDAANSWVAANIVPYRSD----VNFTYITVGNEAIPGAMS----QY

PtGHL17_39 NIGVTVGIPNSMLKSLNSSKKVAESWVHDNVTRYVSSGNSGVRIEYVAVGDEPFQQSYG---EQY

PtGHL17_40 GIEVMVAIPNDQLAVM-GDYNRAKDWVKRNVTRYNFNGG--ITIKYVAVGNEPFLTSYN---GSF

PtGHL17_41 GIEVMVGIPNDLLASLAGSMKAAEKWVSRNVSAHVTNNN--VNIRYVAVGNEPFLQTYN---GSF

PtGHL17_42 GIQIMVGIPNDMLYNLANSEQAADTWVAKNVSSHISSGG--ADIRYVAVGNEPFLSSYN---GSF

PtGHL17_43 SIELMLGVPNSDLPSISSSQANADAWVQNNVLKY-SN----VRFRYIAVGNEVKPGD------DF

PtGHL17_44 -----------------------------------------VLFRWIILGNEVIPGPLA----NY

PtGHL17_45 NIEVVLGVPNDKLQSL-TDASAATTWVQDNVVAYSSN----VKFRYIAVGNEVHPGDA-----NA

PtGHL17_46 GIQVMVGIPNEFLAPLASSVQVAINWVQQNVSSYISRYG--TDIRYVAVGNEPFLQTFK---DTF

PtGHL17_47 GIEVMVGIPNNQMSIVAGDIEDAEDWVKENITAYLHNGG--VDIKYVAVGNEPFLSSYN----NT

PtGHL17_48 NIELMLGLPNSDLERIASSQTNANAWVQRNVRSF-GN----VRFRYIAVGNEVRPFD------SY

PtGHL17_49 GIEVMLGVPNSDLQRL-SNPSDANSWVKNNVLNF-WP-S--VKFRYIAVGNEISPVNGG---TSW

PtGHL17_50 NLVVTIGVPDEAIAHVASSQEAADKWFRDHVLTYVHK-G--VRFRYICVGNEAIPGVVQ----SL

PtGHL17_51 NIELILGILNPDLQGIASSQDNANAWVQNNVRNF-GN----VRFRYIAVGNEVKPSD------SS

PtGHL17_52 NIELTLG----------------------------------ALIQ------------------TF

PtGHL17_53 DIEVMLAIPNYMLHEISQDPAAATAWVDENVTSWSYTGG--VKIRYVAVGNEPFLQTYN---GTY

PtGHL17_54 NIELTIGLGNEDIQKM-TVPTEAENWIQQNVQPH-IP-Q--TKITCIAVGNEVFSSNDA----QL

AT3G57270.1 NIEVTLGLPNSYLQSVASSQSQANAWVQTYVMNY-AN-G--VRFRYISVGNEVKISD------SY

AT3G57260.1 DIELILDVPSSDLERLASSQTEADKWVQENVQSY-RD-G--VRFRYINVGNEVKP--------SV

AT3G57240.1 NIELVLDVPNPDLQRLASSQAEADTWVRNNVRNY-AN----VTFRYISVGNEVQPSD------QA

AT4G16260.1 GIEVIIGVPNTDLRSL-TNPSSARSWLQNNVLNY-YP-A--VSFKYIAVGNEVSPSNGG------

AT5G56590.1 SIELMIGVPNSDLNAFSQSQSNVDTWLKNSVLPY-YP-T--TKITYITVGAESTDDPHI----NA

AT1G77790.1 GLLVAFGPRNEDIQSLAHDPAAATNFVSTWITPYQND----VAIKWITIGNEVFPGEIA----QF

AT1G77780.1 GLLVAIGPRNEAIKTLAEEYQFALNWDKTFIAPY-KN----VAFNWITVGNEVIEGEIG----RY

AT4G26830.1 KIKVIVALPNELLSSAASHQSFADNWIKTHIMPY-FP-A--TEIEAIAVGNEVFVDP------TI

AT4G29360 GIELMIGVPNADLLAFAQFQSNVDTWLSNNILPY-YP-S--TKITSISVGLEVTEAPD-----NA

AT3G07320 DITVSVMVPNELLVNISKSASLSDDWIRSNILPF-YP-T--TKIRYLLVGNEILSLPDS----EL

AT2G16230 GIGIVIGVANGDLPSIASDLNIASQWINSNVLPF-YP-A--SNIILINVGNEVLLSNDL----NL

AT5G20340 DIAVTVGVRDQDLAALSASEEAVKGWFATNIEPYLSD----INIAFITVGNEVIPGP-------I

AT2G05790 GIKVTVDLPNELLFSAAKRTSFAVSWVKRNVAAY-HP-S--TQIESIAVGNEVFVDTH-----NT

AT5G55180.1 GIKVVVSLPNENLASAAADQSYTDTWVQDNIKKY-IP-A--TDIEAIAVGNEVFVDPR-----NT

AT5G20330 DIEVTVGVKDQDLAALAASEEAVKGWFAANIESYLAD----VNITFITVGNEVIPG---------

AT2G01630 GVQVIISVPNDQLLGISQSNATAANWVTRNVAAY-YP-A--TNITTIAVGSEVLTSLT-----NA

AT4G34480 GVGIVIGAANGDVPSLASDPNAATQWINSNVLPF-YP-A--SKIMLITVGNEILMSNDP----NL

AT1G66250 GIKVIISIPNDQLLGIGQSNSTAANWVKRNVIAH-YP-A--TMITAVSVGSEVLTSLS-----NA

AT5G20390 NIGVMVDVKNQDLEALSVSEEAVNTWFVTNIEPYLAD----VNITFIAVGNEVIPGE-------I

AT3G61810 GITLSLCIPNDKIPSLATNLSEAESIIRNFILPY-HK-N--TIITAISVGNEVSLLP------QF

AT1G32860 GIEFIIGLGNEYLSKM-KDPSKALTWIKQNVTPF-LP-A--TNITCITIGNEILALNDS----SL

AT2G27500 QVDFMIGLGNEYLQNMSTDPTKAQDWLQQRLEPHISK----TRITSIVVGNEIFKTNDH----VL

AT1G33220 NIEVTVGVRDQDLGALSANEEAVKGWFATNIEPYLAD----VNIAFITVGNEVIPGP-------I

AT3G15800 GIEIIVGLGNEFLKDISVGEDRAMNWIKENVEPF-IRGG--TKISGIAVGNEILGGTDI----GL

AT3G23770 NLYVTIMVPNNQIISIGADQAAADNWVATNVLPF-HP-Q--TRIRFVLVGNEVLSYSSDQDKQIW

AT5G42720 GIEIVIGASNGDVPGLASDPSFARSWVETNVVPY-YP-A--SKIVLIAVGNEITSFGDN----SL

AT2G26600 GLDLVVGLPNGFLKEMSSNADHAFSWVKENIQSF-LP-K--TRIRGIAIGNEVLGGGDS----EL

AT4G14080.1 NLYVTITVPNHQITALSSNQTIADEWVRTNILPY-YP-Q--TQIRFVLVGNEILSYNSG----NV

AT3G46570 GISLTVTVPNSDIISL-SKLSNARSWLSDNLLPFLLT----TSIRYIAVGNEVVATSDK----TL

AT4G18340 NIEIIVTIENQVLPLL-QDPQQATQWVDSHIKPY-VP-A--TRITGIMVGNELFTDDDS----SL

AT1G30080 NIELFVTVENEMLPSL-VDPQQALQWVTTRIKPY-FP-A--TKIGGIAVGNELYTDDDS----SL

AT5G42100 GFELTVALGNEYLAQM-SDPIKAQGWVKENVQAY-LP-N--TKIVAIVVGNEVLTSNQS----AL

AT5G58090 GIEVMVGIPNEMLATLASSLKAAEKWVAKNVSTHISTDN--VNIRYVAVGNEPFLSTYN---GSY

AT1G11820 KVRVIISVPNNQLLAIGSSNSTAASWIGRNVVAY-YP-E--TLITAISVGDEVLTTVP-----SS

AT3G24330 DIEVMIGIPNRFLKEMAQDTSVAASWVEENVTAYSYNGG--VNIKYIAVGNEPFLQTYN---GTY

AT4G31140.1 GIQVMVGIPNDLLAPLAGSVAAAERWVSQNVSAHVSSNG--VDIRYVAVGNEPFLKAFN---GTF

AT4G17180 GIQVMIGIPNEMLSTFNSDL-----FVQQNLSRFIGKNG--ADIRYVAVGNEPFLTGYG---GQF

AT3G13560 SIEVMVGVTNEEILKIGRFPSAAAAWVNKNVAAY-IP-S--TNITAIAVGSEVLTTIP-----HV

AT5G24318 GIAVTVTVPNDQIPHL-TNLSSAKQWISDHIQPH-FP-S--TNIIRILVGNEVISTADH----LL

AT2G39640 EINITIMVPNGNIPAM-VNVANARQWVAANVLPFQQQ----IKFKYVCVGNEILASNDN----NL

AT2G19440 GLEVMVAIPNDQLKVM-GSYDRAKDWVHKNVTRYNFNGG--VNITFVAVGNEPFLKSYN---GSF

AT3G04010 GVEVMVAIPNDLLLAM-GNYQRAKDWVQRNVSRFNFNNG--VKIKYVAVGNEPFLTAYN---GSF

AT5G18220 GIEVMVAIPNDQLKAM-GSYNRAKDWVRRNITRFNDD----VKIKYVAVGNEPFLTAYN---GSF

AT1G64760 GLEVMVAIPNDQLKVM-TSYDRAKDWVRKNVTRYNFDGG--VNITFVAVGNEPFLKSYN---GSF

AT5G58480 NIGVTIGIQNSMLKSLNASVKVAESWVHDNVTRY-FNGGNRVRIEYVAVGEEPFLQSYG---NQY

AT3G55430 GISVVVTVPNGDIPAL-ANGRQARRWVSVNILPF-HP-Q--TKIKYISVGNEILLTGDN----NM

AT5G20870 GIQVMVGIPNDLLATMASTVTNAELWVQQNVSQYISRYG--TDIRYVAVGNEPFLKTYN---NRF

AT3G55780 NVSLFLSVPNPLVPLLASNRSLAMRWVYRHVLPF-YP-R--TKISIISVGNDVISYSP-----DV

AT5G64790 GMEVMVGIPNNLLESLADDYDNAKDWVKENVTQYIRKGG--VDIKYVAVGNEPFLSAYN---GSF

AT5G20560 DIGVTVGIKNEDLEALAANKDAVKSWFSTNIDPYIAD----VNITFITVGNQAIPGD------KH

PtGHL17_1 VT----V-LVPAMNYLHKALVASNL-NFQVKISTPQAMDIIP-R-PFP-PSTATFNSSWSA-TVY

PtGHL17_2 VP----V-LVPAMNYLHKALVASNL-NFQVKVSTPQAMDVIP-R-PFP-PSTATFNSSWDS-TIY

PtGHL17_3 AP----V-LVSALKFIHSALVASNL-DGQIKVSTPQSSSIIL-D-SFP-PSQAFFNRSWDP-VMV

PtGHL17_4 AS----V-LVNAMKFIQSALVASNL-DRQIKVSTPLSSSIIL-D-SFP-PSQAFFNKTWNP-VLI

PtGHL17_5 AP----L-LVPAIESLYSALVAANL-HNLIKISTPHSASIIL-D-PFP-PSQAFFNQSWSS-VMQ

PtGHL17_6 SA----L-VVPAMHNVLMALKKVGL-HKKIKVSSTHSLGVLS-R-SFP-PSAGAFNSSHAF-FLK

PtGHL17_7 TP----F-LVPAMKNVHNSLVKFNL--SSIKISSPIALSALQ-S-SYP-SSAGSFKTELIGPVIK

PtGHL17_8 TK----F-LIPAMKNIHQALVKFNL-HSSIKVSSPIALSALQ-S-SYP-SSAGSFRPELIEPVFK

PtGHL17_9 TK----F-LIPAMRNIHQALVKFNL-HSSIKISSPVALSALQ-S-SYP-SSAGSFRPELIEPVFK

PtGHL17_10 VA----S-LVPAMKALHTALVRASL-DHRIKISTPHSLGILS-S-SSP-PSTGKFRQGYATRILK

PtGHL17_11 IS----Q-LLPAMQNMQKALSSASL-GGKVKVSTVHSMAILS-R-SDP-PSSGLFNPAYQD-TMR

PtGHL17_12 IS----Q-LLPAMQNMQKALSSASL-GGKVKVSTVHSMAILS-Q-SDP-PSSGLFSPAYQD-TMK

PtGHL17_13 IS----R-LVPCMKSLHQALVHAGI--KDVQVSTPHTLGILH-N-SVQ-PSAARIRPGYDRVIFA

PtGHL17_14 IS----N-LVPAMRTIHKALLLAGI--RNVQVTTPHSLGILS-I-SEP-PSAGQFRRGFDRAIFA

PtGHL17_15 FN------LVPAMRRIKISLTRHNI--RKIKVGTPSAINVLE-S-SFP-PSNGTFRPDISGTVIK

PtGHL17_16 IA----H-TLPAMKALTSALELANI--TTIHVATPHSLGILS-S-SEP-PSTGRFRKGYDQRIFA

PtGHL17_17 LA----Y-LVPAVVNIHSALAQLGL-DRYIQVSTPNSLAVLA-E-SFP-PSAGTFKTEVSG-VMS

PtGHL17_18 TD----N-LIPAMQNIHTALVNLGL-DKQVSVTTAHSLAILE-V-SYP-PSAGSFRKDLVG-CIT

PtGHL17_19 LA----S-VVPAIVSIHGALAQLGL-DTYIQVSTPNSLAVLA-E-SYP-PSAGSFKTEVSG-IMS

PtGHL17_20 WE----V-LLPSVKNVYGALGRLGL-TKVVEVSSPHSEAVFT-N-SFP-PSACVFKDDVLV-YMK

PtGHL17_21 TD----N-LLPAMQGIQTALVNLGL-DKQVSVTTAHSLAVLD-V-SFP-PSAGSFRKDLVG-SIT

PtGHL17_22 KP----NALIQAMQNLHAVLVQRGL-DRKIKVTTPHSMAVLA-S-SFP-PSASTFATTLMP-VMT

PtGHL17_23 WE----V-LLPAVKNVYDALRRLDL-TKVVEVSSPHSEAVFT-N-SFP-PSSCVFREDVSI-YMK

PtGHL17_24 WS----N-LLPAMKMVHNTLVNLGL-DKQVIVTTAHPFTIIG-N-SYP-PSSGTFRQDIIG-YMH

PtGHL17_25 WE----A-LLGAVKNIYNAVDKLGL-ADVVQISTAHSQAVFD-N-SYP-PSSCIFRENVAQ-FMK

PtGHL17_26 WS----N-LLPAMKMVYSTLVNLGL-DKQVIVTSAHSFNIIG-N-SYP-PSSGTFRQDLAE-YIQ

PtGHL17_27 VN----K-LLPAMQNVQNALNDASL-GGKIKVSTVHSMGVLK-Q-SEP-PSSGSFDPSYGD-LMK

PtGHL17_28 MN----K-LLPAMQNVQNALNDVSL-GGEIKVSTVHSMGVLK-Q-SEP-PSSGSFDPSYED-LMK

PtGHL17_29 VN----R-LLPAMQNVQNALNDASL-GGKIKVSTVHSMGVLK-Q-SEP-PSSGSFDPSYGD-LMK

PtGHL17_30 MN----K-LLPAMQNVQNALNDASL-GGKIKVSTVHSMGVLK-Q-SEP-PSSGSFDPSYGD-LMK

PtGHL17_31 MN----K-LLPAMQNVQNALNDASL-GGKIKVSTVHSMGVLK-Q-SEP-PSSGSFDPSYGD-LMK

PtGHL17_32 IS----H-LVPCIKSLHQALVRAGI--NDVKVSTPYTLGILQ-N-SVQ-PSAARIKPAYGKVVFA

PtGHL17_33 MN----K-LLPAMQNVQNALNDASL-GGKIKVSTVHSMGVLK-Q-SEP-PSSGSFDPSYGD-LMK

PtGHL17_34 MN----R-LLPAMQNVQNALNDASL-GGKIKVSTVHSMGVLK-Q-SEP-PSSGSFDPSYGD-LMK

PtGHL17_35 LG----T-TLPALQNIHSALTKAGL-DTRVKVTVPLNADVYE-SPTNI-PSDGNFRKDIQD-LML

PtGHL17_36 QS----F-VVPALLNLQQSLVKANL-AGYVKLVVPCNADAYE---SSL-PSQGAFRPELTQ-IMT

PtGHL17_37 MN----K-LLPAMQNVQNALDDASL-GGKIKVSTVHSMGVLK-Q-SEP-PSSGSFDPSYGD-LMK

PtGHL17_38 IA--------QAIANMYTALADAAI--TYIKVSTVVPGSSLS-I-SYP-PSAGAFTHEAAA-VIS

PtGHL17_39 HP----F-VIGAAMNIQTALARVSL-ANQVKVVVPCSYDTFQ-SESSL-PSKGHFRPDLNK-TMT

PtGHL17_40 LN----T-TFPALRNIQNALNDAGV-GDSIKATVPLNADVYGSPDDQAYPSSGRFRSDIND-IMT

PtGHL17_41 LR----T-TFPALQNVQSALIKAGL-GNLIKVTVPLNADVYE-SSSGL-PSGGDFRADIHD-LML

PtGHL17_42 LG----T-TLPALRNIQSALTKAGL-STRVKVTVPLNADVYE-SPTNL-PSDGDFRSDIHD-LML

PtGHL17_43 AS----S-LFPAMQNIQNSISAAGL-GNQIKVSTVTFAAALG-E-SYP-PSRGVFNAEYHS-LLA

PtGHL17_44 VP--------AAIANTRNSLAAIGL--ANVTVTTAIPGNALE-A-SYP-PSAGAFSSDVTD-VMI

PtGHL17_45 QS------VLPAMQNIHNAIASANL-QDQIKVSTAIDTTLLG-S-SYP-PSDGSFSDSASS-YIN

PtGHL17_46 LN----T-TFPALQNIQAALIKAGL-GRQVKVTVPLNADVYQ-TDSGL-PSGGNFRSDIHG-LMI

PtGHL17_47 YD----NITFPALQNVQKALDKAGV-GDKIKATVALNADVYE-SLSDK-PSGGDFRKDIKD-IMI

PtGHL17_48 AQ----F-LVPAMKNIRNALYSAGL--GNIKVSTAIDNGVIE-DDSSP-PSKGSFRGDHRP-FLD

PtGHL17_49 MA----PFVLPALVNVFNAVRAAGL-QDQIKVSIAVDMTLIG-T-SYP-PSAGAFRGDVIS-YLA

PtGHL17_50 VP--------QAIINLYNSVRKASV--DYIYVTTAVGGKVLE-S-SYP-PSAGRFANGVDK-IMN

PtGHL17_51 AQ----F-LVPAMQNIRNALDSAGL--GSIKVSTAIDPEVLT-DDSFP-PSKGSFRAEYRP-LLD

PtGHL17_52 KR----S-LMPQLQTIGLVMMQLNL------FYLQCETSTVN-L-SYP-PSAGAFSTSAGP-YIN

PtGHL17_53 LH----V-TLPALKNIQHALNRAGL-GSHVKATVPFNADIYNSPGSNPVPSAGDFRPDVRD-LTI

PtGHL17_54 MF----N-LLPAMKMIHKTLVNLGL-DKQVMITTPHSFNILE-N-SYP-PSCGTFREDLAE-YIK

AT3G57270.1 AQ----F-LVPAMENIDRAVLAAGL-GGRIKVSTSVDMGVLR-E-SYP-PSKGSFRGDVMV-VME

AT3G57260.1 GG----F-LLQAMQNIENAVSGAGL---EVKVSTAIATDTTT-D-TSP-PSQGRFRDEYKS-FLE

AT3G57240.1 AS----F-VLPAMQNIERAVSSLGI-----KVSTAIDTRGIS---GFP-PSSGTFTPEFRS-FIA

AT4G16260.1 -D----V-VLPAMRNVYDALRGANL-QDRIKVSTAIDMTLIG-N-SFP-PSSGEFRGDVRW-YID

AT5G56590.1 SS----F-VVPAMQNVLTALRKVGL-SRRIKVSTTLSLGILS-R-SFP-PSAGAFNSSYAY-FLR

AT1G77790.1 VA--------AAIKNVNVALTNSGV--TGISVTTVLAMTALT-N-TYP-PSAATFLPDLTE-IMT

AT1G77780.1 VP--------QAMKNIKAALTEIGN--SKIHVTTVISTAALA-N-SYP-PSAGVFKPAITE-LIT

AT4G26830.1 TP----Y-LVNAMKNIHTSLVKYKL-DKAIKISSPIALSALA-N-SYP-PSSGSFKPELIEPVVK

AT4G29360 TG----L-VLPAMRNIHTALKKSGL-DKKIKISSSHSLAILS-R-SFP-PSSASFSKKHSA-FLK

AT3G07320 KS----S-LVPAMRKIQRSLKSLGV--KKVKVGTTLATDVLQ-S-SFP-PSSGEFREDISGLIMK

AT2G16230 VN----Q-LLPAMQNVQKALEAVSL-GGKIKVSTVHAMTVLG-N-SEP-PSAGSFAPSYQA-GLK

AT5G20340 GP----Q-VLPVMQSLTNLVKSRNL---PISISTVVAMWNLE-Q-SYP-PSAGMFTSQARE-QLV

AT2G05790 TS----F-LIPAMRNIHKALMSFNL-HSDIKISSPLALSALQ-N-SYP-SSSGSFRPELIDSVIK

AT5G55180.1 TT----Y-LVPAMKNVQSSLVKFNL-DKSIKISSPIALSALA-S-SYP-PSAGSFKPELIEPVIK

AT5G20330 --PIGPQ-VLPVMQSLTNLVKSRNL---PISISTVVAMSNLE-Q-SYP-PSAGMFTSQARE-QLV

AT2G01630 AS----V-LVSALKYIQAALVTANL-DRQIKVSTPHSSTIIL-D-SFP-PSQAFFNKTWDP-VIV

AT4G34480 VN----Q-LLPAMQNVQKALEAVSL-GGKIKVSTVNSMTVLG-S-SDP-PSSGSFAAGYQT-GLK

AT1G66250 AP----V-LVSAIKNVHAALLSANL-DKLIKVSTPLSTSLIL-D-PFP-PSQAFFNRSLNA-VIV

AT5G20390 GS----Y-VLPVMKSLTNIVKSRSL---PILISTTVAMTNLG-Q-SYP-PSAGDFMPQARE-QLT

AT3G61810 SN----H-LVSAMVNVHKAIKRYRL-HKKIKVSTTHSLAILS-R-RFP-PSTAVFHQSIGDSVLE

AT1G32860 TT----N-LLPAMQGVHSALITAGL-SDQISVTTAHSLSILK-S-SFP-PSAGEFQPDLLD-SLT

AT2G27500 IQ----S-LLPAMKSVYAALTNLGL-EKQVTVTSAHSLDILS-T-SYP-PSSGSFKEEFIQ-YLQ

AT1G33220 GP----Q-VLPVMQSLTILVKSMNL---PISISTVVAMSNLE-Q-SYP-PSAGEFTSQARE-QLV

AT3G15800 WE----A-LLPAAKNVYSALRRLGL-HNVVEVSSPHSEAVFA-N-SYP-PSSCTFRDDVAP-FMK

AT3G23770 AN------LVPAMRKVVNSLRARGI--HNIKVGTPLAMDALR-S-SFP-PSSGTFREDIAVPVML

AT5G42720 MS----Q-LLPAMKNVQTALEAASLGGGKIKVSTVHIMSVLA-G-SDP-PSTAVFKPEHAD-ILK

AT2G26600 AG----A-LLGAAKNVYNALKKMNL-EDTVQITTAHSQAVFS-D-SYP-PSSCVFKENVVQ-FMK

AT4G14080.1 SV----N-LVPAMRKIVNSLRLHGI--HNIKVGTPLAMDSLR-S-SFP-PSNGTFREEITGPVML

AT3G46570 IT----H-LLPAMETLTLALHLANV--SRILVSTPHSLGILS-GSSEP-PSSGKFRKGYDKAIFS

AT4G18340 IG----Y-MMPAIINIHKALVQLGL-DRYIQVSSPSSLAVLG-E-SYP-PSAGSFKPEVSS-VMQ

AT1G30080 IG----Y-LMPAMMSIHGALVQTGL-DKYIQVSTPNSLSVLQ-E-SYP-PSAGCFRPEVAG-VMT

AT5G42100 TA----A-LFPAMQSIHGALVDCGL-NKQIFVTTAHSLAILD-V-SYP-PSATSFRRDLLG-SLT

AT5G58090 LS----T-TFPALRNIQIAIIKAGL-QNQVKVTCPLNADVYD-SSTTF-PSGGDFRANIRD-LMI

AT1G11820 AP----L-LLPAIESLYNALVASNL-HTQIKVSTPHAASIML-D-TFP-PSQAYFNQTWHS-IMV

AT3G24330 VE----F-TLPALINIQRALEEADL--KNVKVTVPFNADIYFSPEANPVPSAGDFRPELRD-ATI

AT4G31140.1 EG----I-TLPALQNIQSAIIKAGL-ATQVKVTVPLNADVYQ-SASNL-PSDGDFRPEIRD-LML

AT4G17180 QN----Y-VVPTMVNLQQSLVRANL-ASYVKLVVPCNADAYQ---SNV-PSQGMFRPELTQ-IMT

AT3G13560 AP----I-LASALNNIHKALVASNL-NFKVKVSSPMSMDIMP-K-PFP-PSTSTFSPSWNT-TVY

AT5G24318 IR----T-LIPAMQSLHTALVSASL-HRRIQISTPHSLGTLT-N-STP-PSSAKFRRGYDAQVLK

AT2G39640 IS----N-LVPAMQSLNEALKASNL--TYIKVTTPHAFTISY-N-RNT-PSESRFTNDQKD-IFT

AT2G19440 IN----L-TFPALQNIQNALNEAGL-GSSVKATVPLNADVYDSPSSNPVPSAGRFRPDIIG-QMT

AT3G04010 IN----L-TYPALFNIQTALNEAGV-GDFTKATVPLNADVYNSPPDNQVPSAGRFRSDIIQ-EMT

AT5G18220 IN----L-TYPALFNIQKALNEAGV-GDFIKATVPLNADVYNSPLENPVPSAGSFRQDIFE-EMK

AT1G64760 IN----L-TFPALANIQNALNEAGL-GNSVKATVPLNADVYDSPASNPVPSAGRFRPDIIG-QMT

AT5G58480 KP----F-VIGAAMNIQNALVKANL-ANEVKVVVPSSFDSFL-SESGR-PSSGHFRADLNK-TMI

AT3G55430 IN----N-LLPAMRNLNNALVRAGV--RDVKVTTAHSLNIIAYDLTGA-PSSGRFRPGWDKGILA

AT5G20870 VR----S-TYPALQNVQAALVKAGL-GRQVKVTVPLNADVYE-SSDGL-PSSGDFRSDIKT-LMI

AT3G55780 SP----F-LLRAMQNVHLSLVDLRI--YKISVSTTFSFFNIV-PTAFP-PSSAQFQQPNGEVIIR

AT5G64790 LK----T-TFPALKNIHKALKEAGH-TNIMKATIPQNAEVYQ-SANDK-PSEGDFRKDVKQ-TML

AT5G20560 GP----H-VLPVIQSLTDLVKSRNL---QISISTTVTTTSLA-R-LKP-PSAGVLTPQARQ-QLV

PtGHL17_1 QILQFLKNTDSFYMLNAYPYFGYTSGN-GIFPLDYALFR--SLPSVKQIV-DPNTLSHYDSMFDA

PtGHL17_2 QILQFLKNTNSYYMLNAYPYFGYTSGN-GIFPLDYALFR--SLPPVKQIV-DPNTLSHYDSMFDA

PtGHL17_3 PLLKFLQSTGSYFMLNVYPYYDYMQSN-GVIPLDYALFR--PLPPNKEAV-DANTLLHYTNVFDA

PtGHL17_4 PMLNFLQSTGSHLMLNIYPYYDYMQSN-GVIPLDYALLK--PLAPNKEAV-DANTLVHYSNVFDA

PtGHL17_5 PLLQFLSKTGSPLMMNLYPYYVFMQNK-GVVPLENSLFK--PLTPSKEMV-DPNTLLHYTNVLDA

PtGHL17_6 PMLEFLAENQSPFMIDIYPYYAYRDSP-NNVSLDYALFE----T-SSEVI-DPNTGLLYTNMFDA

PtGHL17_7 PMLDFLRQTGSYLMINAYPFFAYAANA-DVISLDYALLK----E-NQGVV-DSGNGLKYNSLLEA

PtGHL17_8 PMLDFLRQTGSYLMVNAYPFFAYESNS-DVISLDYALFR----E-NPGVV-DSGNGLKYFNLFDA

PtGHL17_9 PMLDFLRQTGSYLMVNAYPFFAYESNS-DVISLDYALLR----E-NPGVV-DSGNGLRYFSLFDA

PtGHL17_10 PLLRFLRATNSPFMINPYPFFGFSADT-----LDYALFR----P-NSGVF-DENTKLSYTNMLDG

PtGHL17_11 RLLQFQKDNGSPLAVNPYPFFAYQSDP-RPETLAFCLFQ----P-NSGRV-DSGNGIKYMNMFDA

PtGHL17_12 GLLQFQKDNGSPIAVNPYPFFAYQSDP-RPETLAFCLFQ----P-NSGRV-DSGNGMKYMNMFDA

PtGHL17_13 PMLQFLRQTKSPLMVNPYPYFSYSPSM-----ENYILFK----P-NRGVH-DTNTNITYTNMFVA

PtGHL17_14 PMLQFLRETKSPFMVNPYPYFGYSPKM-----ANYALFK----R-NRGVH-DRYTGITYTNMYDA

PtGHL17_15 PMLQFLNRTKSFFFIDFYPFFAWSENA-HNISLDYALFN----AQNVTYT-DPGTNLTYTNLFDQ

PtGHL17_16 PFLDYHRKTKSPFMVNPYPYFGFKAET-----LNYALFK----P-NSGVF-DAATGNNYTNMFDA

PtGHL17_17 QFLHFLSNTKSPFWINAYPYFAYKDKP-DDIPLDYVLFK----P-NSGMV-DPYTKLHYDNMLYA

PtGHL17_18 PILNFHAKTNSPFLINAYPFFAYKSNP-KQISLDFVLFQ----P-NQGIV-DSKSNFHYDNMLFA

PtGHL17_19 QYLQFLSSTKAPFWINAYPYFAYKDKP-DEVPLDYVLFN----P-NAGMV-DPYTKLHYDNMLYA

PtGHL17_20 PLLQFFSKIGSPFYINAYPFLAYKSDP-EHIDINYALFK----S-NQGIL-DSKTNLHYDNMFEA

PtGHL17_21 PILNFHAKTNSPFLINAYPFFAFKSNP-KQVSLDFVLFQ----P-NQGVV-DPKSNFHYDNMLFA

PtGHL17_22 SIVGFLADTGAPFMVNAYPYFAYRDNP-GMVDLEYALLG----N-ASGVR-DPK-GYVYSNMLDA

PtGHL17_23 PLLQFFSQIGSPFYINAYPFLAYKSDP-EHIDINYALFK----S-NKGIL-DAKTNLHYDNMFEA

PtGHL17_24 AILDFHSQTKSPFLINAYPFFAYKDSP-GQISLDYVLFQ----P-NEGMT-DPNTNLHYDNMLYA

PtGHL17_25 PLLEFFSQIGSPFCLNAYPFLAYMSDP-ENIDINYALFQ----K-TKGIY-DMKTDLHYDNMLDA

PtGHL17_26 AILNFHSQIKSPFLINAYPFFAYKDNP-NQISLEYVLFQ----P-NPGMT-DPNTNLHYDNMLYA

PtGHL17_27 GLLEFNSANGSPFAINPYPYYAYRSDT-RPETLAFCLFQ----P-NAGRM-DGNIKIKYMNMFDA

PtGHL17_28 GLLGFNNATASPFAINPYPYFAYRSDT-RPETLAFCLFQ----Q-NAGRV-DGNTKIKYMNMFDA

PtGHL17_29 GLLEFNSANGSPFAINPYPYYAYRSDT-RPETLAFCLFQ----P-NAGRT-DGNTKIKYMNMFDA

PtGHL17_30 GLLEFNSANGSPFAINTYPYFAYRSDT-RPEILAFCLFQ----P-NAGRM-DGNTKIKYMNMFDA

PtGHL17_31 GLLEFNSANGSPFAINPYPYFAYRSDT-RPETLAFCLFQ----P-NAGRM-DGNTKIKYMNMFDA

PtGHL17_32 PMLEFLRQTKSPLLVNPYPYFSYSPSM-----EDYILFK----P-NPGIH-DDNTNITYTNMFVA

PtGHL17_33 GLLEFNSANGSPFAINPYPYFAYRSDT-RPETLAFCLFQ----P-NAGRM-DGNTKIKYMNMFDA

PtGHL17_34 ALLEFSRANGSPFAINPYPYFAYRSDT-RPETLAFCLFQ----P-NAGRM-DGNTKIKYMNMFDA

PtGHL17_35 SIVKFLSDNGAPFTVNIYPFISLYTTP--NFPLGFAFFN------NTSSSSLTDGGKIYDNVFDA

PtGHL17_36 QLVSFLNSNGSPFVVNIYPFLSLYGNS--DFPQDYAFFE----G-STHPV--TDGSNVYYNAFDG

PtGHL17_37 GLLEFNSANGSPFAINTYPYFAYRSDT-RPETLAFCLFQ----P-NAGRM-DGNTKIKYMNMFDA

PtGHL17_38 SIAPILLNHGASLMLNVYPYFAYASDT-NSMSLDYALLR----P--GAPL-VGDQNLVYDNIFDA

PtGHL17_39 ELLTFLTKHHSPFFATISPFIISHRNK--NISLDFSLFK------ETKHS-RNDGHRTYKNSFDL

PtGHL17_40 QIVQFLSQNGAPFTVNIYPFLSLYGND--DFPFDYAFFD----G-APQPVVDKGTGIQYTNVFDA

PtGHL17_41 TIVKFLNDAGAPFTVNIYPFISLYIDS--NFPVEYAFFD----G-NANPV--NDGGTSYYNMFDA

PtGHL17_42 SIVKFLSDNGAPFTVNIYPFISLYSDP--NFPLGFAFFG------NKSFP-LNDGGTIYDNVFDA

PtGHL17_43 PIISFLVSNQSPFLVNLYPYFSRAENN--DIPLNYALLV----P-DPSAT-VSDPPFEYNNLFAA

PtGHL17_44 AVAGILASSDAPLMINVYPYFAYASNP-SQVPVDYALFA----A--TTPV-VTDGSFLYYDLFDA

PtGHL17_45 PIINFLRTNGSPLLANVYPYFSYTGNP-QSIDLSYALFT------SPGVV-VQDGQYGYQNLFDA

PtGHL17_46 SIIKFLSDNNAPLTINIYPFLSLYADP--HFPVDYAFFN----G-TSAPV--VDGSISYTNVFEA

PtGHL17_47 QIIKFLHQNKAPFVVNIYPFLSLYQNA--GFPFDYAFFD-------GGKT-ISDKNVSYSNVFDA

PtGHL17_48 PIIRFLLNNQAPLLVNLYPYLSYTGNS-EDIRLDYALFT------APSSL-VSDPPLNYQNLFDA

PtGHL17_49 PIVGHLSYAKTPLFANIYTYFSYSGNP-RDISLPYSLFT------SPSVL-VWDSGRGYQNLFDA

PtGHL17_50 NLTNYLYNIGSPLLINLYPYHALVSEP-QHISLDYALFQ------SQKPV-FTDGDLEYYNLFDA

PtGHL17_51 PIIRFLVDKQSPLLVNLYPYFTYSGDTAGNIPLDYALFT------APSSP-VSDPPLNYQNLFDA

PtGHL17_52 PIVQFLATTGAPLLVNVYTCFSYIDNP-QHIDLGYALLN----P--KGPA-VQDGDLNYHNLFDV

PtGHL17_53 EVVQFLNENDAPFTVNIYPFLSLYGDP--NFPLEFAFFD----G-PKKPI--TDGDSVYTNAFDA

PtGHL17_54 PLLSFLSQIKSPFFINAYPFFAYKADP-TQISLDYVLFQ----P-NKGMK-DPTTNLLYDNMLYA

AT3G57270.1 PIIRFLVSKNSPLLLNLYTYFSYAGNV-GQIRLDYALFT----A-PSGIV--SDPPRSYQNLFDA

AT3G57260.1 PVIGFLASKQSPLLVNLYPYFSYMGDT-ANIHLDYALFT------AQSTV-DNDPGYSYQNLFDA

AT3G57240.1 PVISFLSSKQSPLLVNNYPYFSYTGNM-RDIRLDYTLFT------APSTV-VNDGQNQYRNLFHA

AT4G16260.1 PVIGFLTSTNSALLANIYPYFSYVDNP-RDISLSYALFT----S--PSVV-VWDGSRGYQNLFDA

AT5G56590.1 PMLEFLAENKSPFMIDLYPYYAYRDSP-NNVSLDYVLFE----S-SSEVI-DPNTGLLYKNMFDA

AT1G77790.1 EITSILSETNSPLMTNIYPYFAYASDP-YHISLDYASFK------SNTPV-VIDGDLYYNNMFEA

AT1G77780.1 EIVSILSSTDSPLMVNVYPYFAYASDP-SHVSLEYATFR----S--TSPV-VTDGKYQYTNIFDA

AT4G26830.1 PMLALLQQTSSYLMVNAYPFFAYAANA-DKISLDYALFK----E-NAGNI-DSGTGLKYNSLFDA

AT4G29360 PMLEFLVENESPFMIDLYPYYAYRDST-EKVPLEYALFE----S-SSQVV-DPATGLLYSNMFDA

AT3G07320 PMLQFLNRTKSFLFVDVYPYFAWAQDP-THVDLDYAIFE----STNVTVT-DPVSNLTYHNLFDQ

AT2G16230 GILQFLSDTGSPFAINPYPFFAYQSDP-RPETLAFCLFQ----P-NPGRV-DSNTGIKYMNMFDA

AT5G20340 PVLKLLSQTNSPILVKIYPYFSYASDP-SSIRLDYATFN------TEAIV-VQDGSLGYSNMFDA

AT2G05790 PMLDFLRETGSRLMINVYPFFAYEGNS-DVIPLDYALLR----E-NPGMV-DSGNGLRYFNLFDA

AT5G55180.1 PMLDLLRKTSSHLMVNAYPFFAYAANA-DKISLDYALFK----E-NAGNV-DSGNGLKYNSLLDA

AT5G20330 PVLKLLSQTSTPILVNIYPYFAYASDP-ANIRLDYASFN----T--KSIV-VQDGSLGYSNMFDA

AT2G01630 PLLKFLQSTGSPLLLNVYPYFDYVQSN-GVIPLDYALFQ--PLQANKEAV-DANTLLHYTNVFDA

AT4G34480 GILQFLSDTGSPFAINPYPFFAYQSDP-RPETLAFCLFE----P-NAGRV-DSKTGIKYTNMFDA

AT1G66250 PLLSFLQSTNSYLMVNVYPYIDYMQSN-GVIPLDYALFK--PIPPNKEAV-DANTLVRYSNAFDA

AT5G20390 PVLKFLSQTNTPILVNIYPYFAYAADP-INIQLDYAIFN------TNKVV-VQDGPLGYTNMFDV

AT3G61810 PLIRFLQRTNSPLMVNVYPYLAYKQSF-PSIPLDFALFQPMNSPRRRRYI-DPYTGVAYTNLFDI

AT1G32860 PILEFHRKTDSPFLINAYPFFAYKGNP-KEVPLDFVLFQ----P-NQGIV-DPATGFHYDNMLFA

AT2G27500 PLLDFHSQIESPFLINAYPFFAYKDSP-KEVPLEYVLFQ----P-NQGMV-DPNTNLHYDNMLFA

AT1G33220 PVLKLLSQTSTPILVNIYPYFPYASDP-TNIPLDYATFN------TKATV-VQDGPLGYSNMFDA

AT3G15800 PLLAFFWQIQSPFYINAYPFLAYKSDP-ITIDINYALFE----H-NKGIL-DPKTKLHYDNMFDA

AT3G23770 PLLKFLNGTNSFFFLDVYPYFPWSTDP-VNNHLDFALFE----S-NSTYT-DPQTGLVYTNLLDQ

AT5G42720 GLLEFNSETGSPFAVNPYPFFAYQDDR-RPETLAYCLFQ----A-NPGRV-DPNSNLKYMNMFDA

AT2G26600 PLLEFFQQIGSPFCLNAYPFLAYTYNP-KEIDINYALFK----P-TEGIY-DPKTDLHYDNMLDA

AT4G14080.1 PLLKFLNGTNSYFFLNVHPYFRWSRNP-MNTSLDFALFQ----G-HSTYT-DPQTGLVYRNLLDQ

AT3G46570 PILDFHNRTKSPFMVNPYPYFGFGPET-----LNYALFN----T-NDVVYVDPVTKLNYTNMFDA

AT4G18340 QLLDFLEATKSPFWINAYPYFAYKDNP-QEIPVDYVLFN----R-NIGMT-DPNTRLHYDNMMYA

AT1G30080 QLLGFLRNTNSPFWINAYPYFAYKDSP-TKIPLDYVLFN----P-NPGMV-DPYTKYRYDNMLYA

AT5G42100 PILDFHVKTGSPILINAYPFFAYEENP-KHVSLDFVLFQ----P-NQGFT-DPGSNFHYDNMLFA

AT5G58090 TIVKFLSENGGPFTVNIYPYISLYTNP--DFPVDYAFFD----G-NAQPL--NDGGTFYYNMFDA

AT1G11820 PLLQFLSKTGSPLMMNLYPYYVYMQNK-GVVPLDNCLFE--PLTPSKEMV-DPNTLLHYTNVLDA

AT3G24330 EIINFLYSHDSPFTVNIYPFLSLYGNA--YFPLDFAFFD----GTNKSLR---DGNLVYTNVFDA

AT4G31140.1 NIVKFLSDNGAPFTINIYPFISLYNDP--NFPVEFAFFD------GTGTP-INDNGRIYDNVLDA

AT4G17180 QLVSFLNSNGSPFVVNIYPFLSLYGNS--DFPQDYAFFE----G-SSHPV--PDGPNTYYNAFDG

AT3G13560 QLLQFLKNTGSFFMLNAYPYYGYTTAN-GIFPLDYALFK--QLSPVKQIV-DPNTLLHYNSMFDA

AT5G24318 PLLSFLRSTSSPFVVNPYPFFGYSIET-----LDFALFR----P-NPGLF-DQHTKLLYTNMLDA

AT2G39640 KILEFHRQAKSPFMINAYTFFTMDTNN-----VNYAIFG----P-SNAIT-DTNTQQTYTNMFDA

AT2G19440 QIVDFLGNNSAPITINIYPFLSLYGND--DFPLNYAFFD------GAKPV--DDNGIAYTNVFDA

AT3G04010 QIVNFLAQNKAPFTVNIYPFLSLYLSS--DFPFEYAFFD------GQNTV--NDNGVIYTNVFDA

AT5G18220 LIVNFLAHNKAPFTVNIYPFLSLYLSS--DFPFDYAFFN------GQNTV--SDNGVIYTNVFDA

AT1G64760 QIVDFLGKNNAPITINIYPFLSLYGND--DFPLNYAFFD------GAEPI--NDNGIDYTNVFDA

AT5G58480 ELLSFLTKHHSPFFVTISPFLSFHQNK--NISLDFSLFK------ETAKA-HKDGRKTYRNSFDL

AT3G55430 PILAYHRRTKSPFMVNPYPYFGFDPKN-----VNFAIFR----TPYKAVR-DPFTRHVYTNMFDA

AT5G20870 SIVRFLADSVSPITFNIYPFLSLNADP--NFPREYAFFPNGGGGGGAKPV--VDGSISYTNVFDA

AT3G55780 PILQFLERTNSSFLINLYPYNMYRSSF--SIPIGFALFEEF--P-FNFRD-DLTTGVRYRNLFDM

AT5G64790 DIVNFFHENDLPFTVNIYPFLSLYLNE--HFPVEFAFLD------GDGQT-MTDKGKNYDNVFDA

AT5G20560 PVLRLLSQTSTPIFVNIYPYYFHASDP-KNVPLEYANFN------NDQIV-VKDGALKYSNLFDA

PtGHL17_1 LVDATYYSIEALN--MSG--ISIVVTETGWPWLG-GANEPDATAENAETFNSNLIRRVL--NDSG

PtGHL17_2 MVDATYYSIDALN--MSG--IPIVVTETGWPWLG-GANEPDATADNAETFNNNMIRRVQ--NDSG

PtGHL17_3 IVDAAYFSMSYLN--FTK--IPIFVTESGWPSKG-DSSEPDATLDNANTYNSNLIRHVL--NNTG

PtGHL17_4 MIDATYFAMDFLN--FTN--VPVMVTETGWPSKG-DSNEPDATLDNANTYNSNLIRHVL--NKTG

PtGHL17_5 MVDAAYYSMKNFN--FTD--VAVLVTESGWPSKG-DSKEPYATIDNADTYNSNMIKHVL--DRSG

PtGHL17_6 QIDAIYFALMALN--FRT--IKVMVTETGWPSKG-SSKEKAATPDNAQIYNTNLIRHVI--NNSG

PtGHL17_7 QLDAVHAAMSAIQ--YND--VKMVVTETGWPSLG-DEDEIGAGEANAASYNGNLVKRVL--TGNG

PtGHL17_8 QIDAVFAALSALK--YDD--VKMVVTETGWPSKG-DENEVGASVENAAAYNGNLVRRIL--TGGG

PtGHL17_9 QIDAVFAALSALK--YDD--IKIVVTETGWPSKG-DENEIGSGVENAAAYNGNLVRRIL--TGGG

PtGHL17_10 QLDAVFSAMKLLG--FSD--IEIVIAETGWPSQG-ESSQLGVDAESAAQYNRNLMQHVT--SGAG

PtGHL17_11 QVDAVRSALNAMG--FID--VEIVVAETGWPYKG-DSNEVGPGIENARAYNGNLVAHLR--SMVG

PtGHL17_12 QVDAVRSALNAMG--FND--IEILVAETGWPYKG-DSNEVGPSVENARAYNGNLISRLR--SMVG

PtGHL17_13 MMDAVYSAIKAMG--YGD--LDIVVAESGWPSLG-DPNQPMCTVENAVSYNKNMIKVVT--SGNG

PtGHL17_14 MLDATYSAMRKLG--YGD--VGIVVGETGWPSVC-DPGQPACSMENAAWFNGNLVRRAR--QGKG

PtGHL17_15 MFDAVVFAMKRLG--YPG--IRVFIAETGWPNGG-DFEQLGANIYNSATYNRNVVKKLTTIPAIG

PtGHL17_16 QLDAVYSAMKRLG--YGD--VDIVVAETGWPSVG-DPNQPGVSMENAISYNKNLVKHVN--SGKG

PtGHL17_17 QVDAVIFAIARMG--FNG--IEVRVSETGWPSKG-DSDEVGATIENAAAYNKNILRRQL--NSEG

PtGHL17_18 QIDAVHSALASLG--YSK--LPVHISETGWPSKG-DADEVGATLENAKKYNGNLLKIIC--QRKG

PtGHL17_19 QVDAVLFAIARMG--FGG--IEVGVSETGWPSKG-DADEVGAIVDNAAAYSKNILRRQL--KNEG

PtGHL17_20 QVDAAYAALEKAG--FPK--MEVIVSETGWASRG-DADEAGASLENARTYNRNLRKRLM--KKKG

PtGHL17_21 QIDAVYSALASLG--YSK--LPVHISETGWPSKG-DEDEVGATLENAKKYNGNLFKTIC--QRKG

PtGHL17_22 QVDAVRSAIIALG--FGNRTVEMTISESGWPSKG-ESGDDAATPENAKTYNTRLIERAQ--SNKG

PtGHL17_23 QVDAAYAALDKAG--FPK--MEVIVSETGWASRG-DDNEAGASLENARTYNRNLRKRLA--KKKG

PtGHL17_24 QVDAVYSAIKAIG--HTD--VEVKISETGWPSKG-DPTEVGSTLQNAELYHSNLLKRIQ--QKQG

PtGHL17_25 QIDATYAALEDAG--FKK--MEVIVTETGWASLG-DTNEAAATVNNARTFNYNLRKRLA--KKKG

PtGHL17_26 QVDAVYSAIKAMG--HTD--IEVMISETGWPSKG-DPDEVGSTPENAALYHSNLLNRIQ--ARQG

PtGHL17_27 QVDAVYSALNSMG--FKN--VEIVVAETGWPFKG-DDNDVGPSIENAKAYNGNLIAHLR--SMVG

PtGHL17_28 QVDAVFSALNSIG--FKN--VEIVVAETGWPYKG-DDNEIGPSIENAKAYNGNLIAHLR--SMVG

PtGHL17_29 QVDAVFSALNSMG--FKN--VEIVVAETGWPFKG-DDNDVGPSIENAKAYNGNLIAHLR--SMVG

PtGHL17_30 QVDAVYSALNSMG--FKN--VEIVVAETGWPFKG-DDNDVGPSIENAKAYNGNLIAHLR--SMVG

PtGHL17_31 QVDAVYSALNSMG--FKN--VEIVVAETGWPFKG-DDNDVGPSIENAKAYNGNLIAHLR--SMVG

PtGHL17_32 MMDAVYSAIKAMG--YDD--LDIVVAESGWPSLG-DPNQPMCTVENAVLYNKNMIKVVT--SGEG

PtGHL17_33 QVDAVYSALNSMG--FKN--VEIVVAETGWPYKG-DDNEVGPSIENAKAYNGNLIAHLR--SLVG

PtGHL17_34 QVDAVYSALNSMG--FKN--VEIVVAETGWPYKG-DDNEVGPSIENAKAYNGNLIAHLR--SLVG

PtGHL17_35 NHDTLVWALQKNG--YGN--LSIVIGEIGWPTDG----DKNANLNYAQQFNQGFMNNVI--AGKG

PtGHL17_36 NFDTLVAALNKLG--YGQ--MPIVIGEVGWPTDG----AIGANLTAARVFNQGLIKHVL--SNKG

PtGHL17_37 QVDAVYSALSSMG--FKN--VEIVVAETGWPFKG-DDNDVGPSIENAKAYNGNLIAHLR--SMVG

PtGHL17_38 MVDAFYAALEKIS--EPG--LTVVISESGWPTAG---NEPITSPENARTYNRNLLNHVQ--EGRG

PtGHL17_39 GYDTLVSALSTAG--FPE--MDVVVAKIGWPTDG----AANATPSAAETFMKGLMDHLH--SKSG

PtGHL17_40 NFDTLVSALKAAG--HGD--MPIVVGEVGWPTDG----DKNANIGYATRFYNGLIPRLV--GNRG

PtGHL17_41 NYDTLVNALQKNG--FGN--LPIIVGEIGWPTDG----DRNANVEYARRFNQGFMSHIA--SGKG

PtGHL17_42 NHDTLIWALQKNG--YGS--LPVVIGEIGWPTDG----DKNANLNYAQQFNQGFMDSVI--SNKG

PtGHL17_43 MVDAVYSALEKAG--GGS--LEIVVSESGWPSAG---GGPETNIDNARTYNTNLVQQVK----NG

PtGHL17_44 MVDAFHAALEKIG--YPG--LRVAIGESGWPSAG---NDPYTSIDNAMIYNRNLVNHVL---TNG

PtGHL17_45 LLDSLYAALEKAG--APD--LNIVVSESGWPSEG----GTAATADNAGTFYRNLINHVK----QG

PtGHL17_46 NFDTLISALEKNG--FSS--MPVIVGEVGWPTDG----DRNANMDYARRFNQALVDRIN--QGQG

PtGHL17_47 NYDTLVWTLKKNG--VGD--LKIIIGEVGWPTDG----NFNANNKLAKKFYDGLLKKLV--AEKG

PtGHL17_48 ILDTVYAALEKSG--GGS--LDIVVSESGWPTAG----GTGTSVDNARIYNNNLVQHVK----RG

PtGHL17_49 MLDSLYSALERLG--GGN-TLDVVVSESGWPSAG----GFGTTSDNAGTYLSNLIRHVK----GG

PtGHL17_50 MVDAFVAAMVRVV-QQED--VKLVVAETGWPTAG---VGSYACTENARIYNLNLRKHAI--EKGC

PtGHL17_51 ILDTIYAALEKSG--GGS--LDIVVSESGWPTAG----GKGTSVDNARTYNNNLVQHVK----TG

PtGHL17_52 SLDALYSALERAG--GLN--VEIVVSETGWLSMG----NDAATFSHAEDYYQNVINHIA----NG

PtGHL17_53 NLDTLIWSLDKAG--YPG--MQIMIGEVGWPTDG----DKNANIQNAKRFNQGLIRHAL--SGNG

PtGHL17_54 QVDAVYSAM---G--HTD--IEVKISETGWPSKG-DPDEVGSTPENARLYHSNLIKRIQ--EKQG

AT3G57270.1 MLDAMYSALEKSG--GAS--LEIVVAETGWPTGG----GTDTNIENARIYNNNLIKHVK----NG

AT3G57260.1 NLDSVYAALEKSG--GGS--LEIVVSETGWPTEG----AVGTSVENAKTYVNNLIQHVK----NG

AT3G57240.1 ILDTVYASLEKAG--GGS--LEIVVSESGWPTAG----GAATGVDNARTYVNNLIQTVK----NG

AT4G16260.1 LLDVVYSAVERSG--GGS--LPVVVSESGWPSNG----GNAASFDNARAFYTNLASRVR--ENRG

AT5G56590.1 QVDALYYALTALN--FRT--IKIMVTETGWPTKGSPKEKAAASSDNAETYNSNIIRHVV--TNQG

AT1G77790.1 MVDGFNAALEKIN--AAN--VVVMVAETGWPTEG---NPPHTSVDNAKAYNMGIRTCGRSAERKR

AT1G77780.1 TLDAFNVALEKIN--HGS--VKVYVAETGWPTRG---NDPYTSVENARAYNQGLLKKLT--TGKG

AT4G26830.1 QIDAVYAALSAVG--FKG--VKVMVTETGWPSVG-DENEIGASESNAAAYNAGLVKRVL--TGKG

AT4G29360 QLDAIYFALTAMS--FKT--VKVMVTESGWPSKG-SPKETAATPENALAYNTNLIRHVI--GDPG

AT3G07320 MIDAFVFAMKRVG--YPD--IRIWVAETGWPNNG-DYDQIGANIYNAATYNRNVVKKLAADPPVG

AT2G16230 QVDAVHSALKSIG--FEK--VEVLVAETGWPSTG-DSNEVGPSVENAKAYNGNLIAHLR--SMVG

AT5G20340 IFDAFVWAMEKEG--VKD--LPMVVSETGWPSAG---NGNITTPDIAGTYNRNFVKHIA--SGKG

AT2G05790 QIDAVFAAMSALK--YDD--IEIIVTETGWPSKG-DENEVGATLANAASYNGNLIRRIL--TRGG

AT5G55180.1 QIDAVFAAMSAVG--FND--VKLVVTETGWPSAG-DENEIGAGSANAAAYNGGLVKRVL--TGNG

AT5G20330 IFDAFVWAMEKEG--VKN--LPMVVSETGWPSAG---NGNFTTPAIASTYNRNFVKHIA--SGKG

AT2G01630 IVDAAYFAMSYLN--FTN--IPIVVTESGWPSKG-GPSEHDATVENANTYNSNLIQHVI--NKTG

AT4G34480 QVDAVHSALKSMG--FEK--VEIVVAETGWASRG-DANEVGASVDNAKAYNGNLIAHLR--SMVG

AT1G66250 MVDATYFAMAFLN--FTN--IPVLVTESGWPSKG-ETNEPDATLDNANTYNSNLIRHVL--NKTG

AT5G20390 IFDAFVWAMEKEG--VKD--LPMVVTETGWPSAG---NGNLTTPDIASIYNTNFVKHVE--SGKG

AT3G61810 MLDSVDSAVKSLG--LPE--IPVVVSEIGWPTRG-DPGETAANLENARVFNQRLVEHLR------

AT1G32860 QIDAVYSALAAAG--FKS--LRVEISETGWPSKG-DDDEVGATPENAKRYNGNLIKMMMSGKKTK

AT2G27500 QVDALYSAIKTLG--HTD--IEVRISETGWPSKG-DENEIGASPENAALYNGNLLKLIQ--QRKG

AT1G33220 IFDAFVWAMEKEG--VKD--LPMVVSETGWPSAG---NGNLTTPDIAGTYNRNFVKHIV--SGKG

AT3G15800 MVDASYAALEKAG--YTK--VPVIVSETGWASKG-DADEPGASVKNARTYNRNLRKRLQ--KRKG

AT3G23770 MLDSVIFAMTKLG--YPN--ISLAISETGWPNDG-DIHETGANIVNAATYNRNLIKKMTANPPLG

AT5G42720 QVDAVYSALNSMG--FKD--VEIMVAETGWPYKG-DPEEAGATVENARAYNKNLIAHLK--SGSG

AT2G26600 QIDAAYMALQDAG--FKK--MEVMITETGWASKG-DSDEPAATPENARTYNYNLRKRLA--KKKG

AT4G14080.1 MLDSVLFAMTKLG--YPH--MRLAISETGWPNFG-DIDETGANILNAATYNRNLIKKMSASPPIG

AT3G46570 QLDAVYSAMKRFG--YGD--VDIVVAETGWPSAG-EPNQTGVGLDYAAAYNGNLIKHVN--SGKG

AT4G18340 QVDAVAFAAAKLG--YRN--IEVRVAETGWPSKG-DVGEIGASPVNAATYNRNLMMRQF--AGEG

AT1G30080 QVDAVIFAMARLG--FKD--IEVGVSETGWPSKG-DGDEVGATVANAAVYNKNILRRQL--QNEG

AT5G42100 QVDAVYHALDAVGISYKK--VPIVVSETGWPSNG-DPQEVGATCDNARKYNGNLIKMMM-SKKMR

AT5G58090 NYDTLVHALEKNG--FGN--MPIIIGEIGWPTDG----DSNANLDYAKKFNQGFMAHIS--GGKG

AT1G11820 MVDAAYVSMKNLN--VSD--VAVLVTESGWPSKG-DSKEPYATIDNADTYNSNLIKHVF--DRTG

AT3G24330 NLDTLICAMERYS--FLG--MKIIVGEVGWPTDG----DKNANVKSAKRFNQGMVKHAM--SGNG

AT4G31140.1 NYDTLVWSLQKNG--FGN--LTIIVGEVGWPTDG----DKNANLMYARRYNQGFMNRQK--ANKG

AT4G17180 NFDTLVAALTKLG--YGQ--MPIVIGEIGWPTDG----AVGANLTAARVFNQGLISHVL--SNKG

AT3G13560 MVDAAYYSMEALN--FSK--IPVVVTETGWPSSG-GSDEAAATVANAETFNTNLIKRVL--NNSG

AT5G24318 QLDSVYSAMDKLG--FSD--VEIVIGEIGWPSEG-DIDQIGVDVDTAAEFNKNLIARVD--SGTG

AT2G39640 VMDATYSAMKALG--YGD--VDIAVGETGWPTAC---DASWCSPQNAENYNLNIIKRAQ---VIG

AT2G19440 NFDTLVSALKAVG--HGD--MPIIVGEVGWPTEG----DKHANSGSAYRFYNGLLPRLG--ENRG

AT3G04010 SFDTLLASLNALN--HGN--MEVIVGEVGWPTDG----DKNANVPNAERFYSGLLPRLA--NNVG

AT5G18220 NFDTLLASLKALG--HGD--MTVIVGEVGWPTDG----DKNANIPNAERFYTGLLPKLA--ANRG

AT1G64760 NFDTLVSSLKAVG--HGD--MPIIVGEVGWPTEG----DKHANAGSAYRFYNGLLPRLG--TNKG

AT5G58480 SYDTLVSALFTIG--FSE--VDIVVSKIGWPTDG----AENATSLTAEAFFKGLIVHLE----KK

AT3G55430 LMDSTYSAMKALG--YGD--VNIVVGETGWPSAC---DAPWCSPANAAWFNLNIIKRAQ---GQG

AT5G20870 NFDTLVSALEKNG--FDANKIEIIVGEVGWPTDG----DQNANPAMAQRFNQGLLNRIL--QGQG

AT3G55780 MVDAVISSMAVMG--HEN--LPVIVAETGWPSSGIDASEVDATLLYSEMFLKALLTHLR--SGCG

AT5G64790 NYDTLVYALKKAG--IHD--MKIIVGEVGWPTDG----HKYASPKLAEKFYAGLMKRLA--KDGG

AT5G20560 IFDAFLWAMEKEG--VKG--LPLVVSETGWPSAG---NGGMTTPALQYTYIGNFVKHVA--SGKG

PtGHL17_1 PPSQPK-VPINTYIYELFNEDK-R--PGPVSEKNWGLFFT-NGSAVYTFSL--------------

PtGHL17_2 PPSQPK-FPINTYIYEMFNEDK-R--PGPVSEKNWGLFFT-NGSSVYTFSL--------------

PtGHL17_3 TPKHPG-IVVSTYIYELYNEDS-R--PGPVSEKNWGLFDA-NGMPVYTLHL--------------

PtGHL17_4 TPKHPG-IAVSTYIYELYNEDL-K--PGPVSEKNWGLFNA-NGEPVYILHL--------------

PtGHL17_5 TPLHPE-ITSSVYLYELFNEDL-R--SPPVSEANWGLFYA-NATPVYLLHV--------------

PtGHL17_6 TPAKLG-EELDVYIFSLFNENR-K--PGMESERNWGLFYP-DQTSVYSLDF--------------

PtGHL17_7 TPLRPQ-EPLNVYLFALFNENE-K--PGPTSERNYGLFYP-NEKRVYDVPF--------------

PtGHL17_8 TPLKPQ-ADLTVYLFALFNENE-K--DGPTSERNYGLFYP-DQQKVYDIPF--------------

PtGHL17_9 TPLRPQ-ADLTVYLFALFNENE-K--DGPTSERNYGFFYP-DEQKVYDIPF--------------

PtGHL17_10 TPLMPN-RTFETYIFALFNEDL-K--PGPPSERNFGLFQP-DMTPVYNIGI--------------

PtGHL17_11 TPLMPG-KSVDTYIFALYDEDL-K--SGPASERSFGLFKP-DLSMTYDIGL--------------

PtGHL17_12 TPLMPG-KSVDTYIFALYDEDL-K--PGPASERSFGLFKP-DLSMTYDIGL--------------

PtGHL17_13 TPLMPK-RRFQTYVFSLFNENL-K--PGSTAERNWGLFRP-EFTPVYDVGI--------------

PtGHL17_14 TPLMPN-RRFETYLFSLFNENL-K--PGPTAERNWGLFRP-DFSPIYDAGI--------------

PtGHL17_15 TPAQPG-VVIPAFIFSLYNENQ-K--PGPGTERQFGLYYP-NGTEVFEIDL--------------

PtGHL17_16 TPLMPN-RTFETYVFSLFNENL----KPSVSERNFGLFKP-DLTPVYDVGI--------------

PtGHL17_17 TPLRPN-MRLEVYLFALFNEDL-K--PGPTSERNYGLFQP-DCSMAYNVGL--------------

PtGHL17_18 TPMRPN-TDFNIYVFALFNENM-K--PGPASERNYGLFKP-DGTPAYSLGI--------------

PtGHL17_19 TPLRPN-MKLEVYLFALFNEDM-K--PGPTSERNYGLFQP-DCTMVYNVGI--------------

PtGHL17_20 TPYRPK-FVARAYIFALFNENL-K--PGPTSERNFGLFKP-DGSIAYDIGF--------------

PtGHL17_21 TPMRPN-TDLNIYVFALFNENM-K--PGPTSERNYGLFKP-DGSPAYLLGI--------------

PtGHL17_22 TPMSPK-KNIEIFVFALFNENK-K--EGGVSERNFGMFNG-DGSKVYEVDL--------------

PtGHL17_23 TPYRPK-FVAKAYIFALFNENL-K--PGPTSERNFGLFKP-DGSISYDIGF--------------

PtGHL17_24 TPAKPS-VPIDVYVFALFNENL-K--PGPTSERNYGLFYP-DGTPVFNIGL--------------

PtGHL17_25 TPLRPK-MVVKAYIFAIFNENL-K--SGPTSERNFGLFKP-DGSISYDIGF--------------

PtGHL17_26 TPAKPS-VPIDIYVFALFNENL-K--PGPTSEKNYGLFYP-DGTPVYNSGL--------------

PtGHL17_27 TPLMPG-KSVDTYLFALYDEDL-K--PGRGSERSFGLFKT-DLTMVYDVGL--------------

PtGHL17_28 TPLMPG-KSVDTYLFALYDEDL-K--PGPGSERSFGLFKP-DLTMAYNVGL--------------

PtGHL17_29 TPLMPG-KSVDTYLFALYDEDL-K--PGPGSERSFGLFKT-DLTMVYDVGL--------------

PtGHL17_30 TPLMPG-KSVDTYLFALYDEDL-K--PGPGSERSPGIFKT-DLTMVYDVGL--------------

PtGHL17_31 TPLMPG-KSVDTYLFALYDEDL-K--PGPGSERSFGLFKT-DLTMVYDIGL--------------

PtGHL17_32 TPLMPK-RRFETYVFALFNENL-K--PGTAAERNWGLFRP-DFSPVYDVGILSNIGKSTGSSPSP

PtGHL17_33 TPLMPG-ESVDTYLFAFYDEDLIK--PGPGSERSSGLFKT-GVTMVYDVGL--------------

PtGHL17_34 TPLMPG-ESVDTYLFAFYDEDLIK--PGPGSERSSGLFKT-GVTMVYDVGL--------------

PtGHL17_35 TPMRPA--PVDAYLFSLFDEDA-KSIQPGNFERHWGLFYL-DGQPKYALSL--------------

PtGHL17_36 TPLRPDAPPMDIYLFALLDEGA-KSVLPGNFERHWGIFSF-DGQAKYALNL--------------

PtGHL17_37 TPLMPG-KSVDTF--------------GPGSERSSGSQTQ-SLAATPQLPP--------------

PtGHL17_38 TPRRPG-QPLDVYFFAMFNEDL----KQAGIEQHWGFFYP-NMQPVYPF----------------

PtGHL17_39 TPLRPRNPPIETYIFSLLDEDQ-RSIVNGNFERHWGVFTF-DGQAKYNVDL--------------

PtGHL17_40 TPLRPG--YIEVYLFGLLDEDA-KSIAPGNFERHWGIFRY-DGQPKFPLDL--------------

PtGHL17_41 TPMRPN-AGINAYLFSLIDEDA-KSIDPGNFERHWGIFTF-DGIPKYSLNL--------------

PtGHL17_42 TPLRPG--YVDAYLFSLIDEDA-KSIQPGNFERHWGLFYL-DGQPKYAVSL--------------

PtGHL17_43 TPKRPG-RPIETYIFATFDENQ----KQPEYEKFWGLFLP-SKQPKYQIQL--------------

PtGHL17_44 TPRRPG-EIMETFLFAMFNENL----KQGAVEQNFGFFYP-NMNPVYPF----------------

PtGHL17_45 TPRRSG-QAIETYLFAMFDENL----KAAGIEQHFGLFLP-NKQPKYQLTF--------------

PtGHL17_46 TPKRKT--PPDIYLFALTDEDA-KSVRPGNFERHWGIFYY-DGAIKYQMDM--------------

PtGHL17_47 TPLRPG--QLDLYLFGLIDENQ-KSIAPGHFERHWGLFYY-DGKPKFPIDL--------------

PtGHL17_48 SPKKPG-KPIETYIFSMFDENY----KNPELEKHWGLFLP-NKQPKYNINL--------------

PtGHL17_49 TPKRPG-KAIETYIFAMFDENQ----KQPELEKHFGAFSP-NKQPKYNLNF--------------

PtGHL17_50 TPRKAD-INLEVYISEMFNENL-Q--P-DEFERNFGTFYP-NLTEVYQL----------------

PtGHL17_51 SPKRPG-KPIETYIFAMFDEVN----KSPELEKNWGLFFP-NKQPKYQIDL--------------

PtGHL17_52 TPKRPG-RPIETYLFAMFDENQ-K--SGAETERHFGLFFP-NKQPKYQLQF--------------

PtGHL17_53 TPKRKG-KNIDVYLFSLIDENA-KAIAPGSFERHWGIFEF-DGKPKYGLDL--------------

PtGHL17_54 TPAKPS-VPIEVYVSALFNEDL-K--TGPTSERNYGLFYP-DCSPVYNIGL--------------

AT3G57270.1 TPKRPG-KEIETYLFAIYDENQ-KPTP-PYVEKFWGLFYP-NKQPKYDINF--------------

AT3G57260.1 SPRRPG-KAIETYIFAMFDENK----KEPTYEKFWGLFHP-DRQSKYEVNF--------------

AT3G57240.1 SPRRPG-RATETYIFAMFDENS-K--QGPETEKFWGLFLP-NLQPKYVVNF--------------

AT4G16260.1 TPKRPG-RGVETYLFAMFDENQ----KSPEIEKNFGLFFP-NKQPKFPITF--------------

AT5G56590.1 TPAKPG-EAMNVYIFSLFNENR-K--AGLDSERNWGLFYP-DQTSVYQLDF--------------

AT1G77790.1 TPRRQN-TPVDVFLFAMFKENQ----KDGPVEQSFGIFAP-DMTPVYDLFC--------------

AT1G77780.1 TPRRPN-VPVITFFFEMFNEDL----KQGAVEQSFGFFDP-NMAPVYDM----------------

AT4G26830.1 TPLRPT-EPLNVYLFALFNENQ-K--PGPTSERNYGLFYP-NEGKVYNVPF--------------

AT4G29360 TPAKPG-EEIDVYLFSLFNENR-K--PGIESERNWGMFYA-NGTNVYALDF--------------

AT3G07320 TPARPG-KVLPAFVFALYNENQ-K--TGPGTERHFGLLHP-NGTQVYGIDL--------------

AT2G16230 TPLMPG-KSIDTYIFALFDENL-K--PGPSFEQSFGLFKP-DLSMAYDIGL--------------

AT5G20340 TPKRPN-KGIDGFLFATFNENQ----KPVGTEQNFGLYNPNDMKPIYNL-F--------------

AT2G05790 TPLRPK-ADLTVYLFALFNENK-K--LGPTSERNYGLFFP-DEKKVYDIPF--------------

AT5G55180.1 TPLKPK-EPLNVYLFALFNENQ-K--TGPTSERNYGLFYP-NENKVYDVSL--------------

AT5G20330 TPKRPN-KSMNGFLFATFNENQ----KPAGTEQNFGLYNPSDMKPIYKL-F--------------

AT2G01630 TPKHPG-TAVTTYIYELYNEDT-R--PGPVSEKNWGLFYT-NGTPVYTLRL--------------

AT4G34480 TPLMPG-KPVDTYIFALYDENL-K--PGPSSERAFGLFKT-DLSMVYDVGL--------------

AT1G66250 TPKRPG-IAVSTYIYELYNEDT----KAGLSEKNWGLFNA-NGEPVYVLRL--------------

AT5G20390 TPKRPK-SGISGFLFATFNENQ----KPAGTEQNFGLYNPTDMKPIYKM-F--------------

AT3G61810 ---RRW-NKVPVYIFALFDEDQ-K--TGNAVEKHWGLLYG-NGSRKYDLNF--------------

AT1G32860 TPLKPN-NDLSIYVFALFNENL-K--PGPTSERNYGLFKP-DGTQAYSLGF--------------

AT2G27500 TPAKQS-VPIDVYVFALFNENL-K--PGPVSERNYGLFYP-DGKPVYNVGM--------------

AT1G33220 TPKRPN-NGMDGFLFATFNENQ-K--PAGT-----------DMKPIYKL-F--------------

AT3G15800 TPYRPD-MVVRAYVFALFNENS-K--PGPTSERNFGLFKP-DGTIAYDIGL--------------

AT3G23770 TPARRG-APIPTFLFSLFNENQ-K--PGSGTERHWGILNP-DGTPIYDIDF--------------

AT5G42720 TPLMPG-RVIDTYLFALYDENL-K--PGKGSERAFGLFRP-DLTMTYDIGL--------------

AT2G26600 TPLRPK-TVLKAYIFALFNENS-K--PGKSSETHFGLFKP-DGTISYDIGF--------------

AT4G14080.1 TPSRPG-LPIPTFVFSLFNENQ-K--SGSGTQRHWGILHP-DGSPIYDVDF--------------

AT3G46570 TPLMPN-RVFETYVFSLFNENL----KSSVSEQNFGLFKP-DFTPVYDVGI--------------

AT4G18340 TPARRN-ARLDVYIFALFNEDM-K--PGPTSEKNYGIFQP-DGSLAYNLGF--------------

AT1G30080 TPLRPN-LSFDVYLFALFNEDL-K--PGPTSERNYGLYQP-DETMTYNVGL--------------

AT5G42100 TPIRPE-CDLTIFVFALFNENM-K--PGPTSERNYGLFNP-DGTPVYSLGI--------------

AT5G58090 TPRRPG--PIDAYLFSLIDEDA-KSVQPGYFERHWGIFTF-DGLPKYALNL--------------

AT1G11820 TPLHPE-MTSSVYIYELFNEDL-R--APPVSEASWGLFYG-NSTPVYLLHV--------------

AT3G24330 TPARKG-VIMDVYLFSLVDEDA-KSIAPGTFERHWGIFEF-DGRPKYELDL--------------

AT4G31140.1 TPMRPG--AMDAYLFGLIDEDA-KSIQPGNFERHWGIFYI-DGQPKYQLSL--------------

AT4G17180 TPLRPGSPPADVYLFGLLDEGA-KSTLPGNFERHWGIFSF-DGQAKYRLNL--------------

AT3G13560 PPSQPD-IPINTYIYELYNEDK-R--SGPVSERNWGILFP-NGTSVYPLSL--------------

AT5G24318 TPLMPN-RTFETYIFALFNENL-K--SGPTSERNFGIFRS-DLTPIYDIGI--------------

AT2G39640 TPLMPN-RHIDIFIFALFNEDG-K--PGPTRERNWGIFKP-DFSPMYDVGV--------------

AT2G19440 TPLRPT--YIEVYLFGLLDEDA-KSIAPGEFERHWGIFKF-DGQPKFPIDL--------------

AT3G04010 TPMRKG--YIEVYLFGFIDEDA-KSVAPGNFERHWGIFKF-DGQPKFPVDF--------------

AT5G18220 TPMRPG--YIEVYLFGFIDEDA-KSIAPGNFERHWGIFKY-DGQPKFPADL--------------

AT1G64760 TPLRPT--YIEVYLFGLLDEDA-KSIAPGPFERHWGIFKF-DGQPKFPIDL--------------

AT5G58480 TASLPR-PPVETYIESLLDEDQ-RNLSAGNFERHWGVFTF-DGQAKYNFSF--------------

AT3G55430 TPLMPN-RRFETYIFGLFNEEG-K--PGPTAERNWGLFRA-DFSPVYDVGL--------------

AT5G20870 TPRRR--MAPEVYIFSLVDEDA-KSIDPGKFERHWGIFSY-DGAVKYPLSL--------------

AT3G55780 TPLRKE-GVSEVYIFELVEKDA-K--QGI---RNWGLLHH-NMTSKYSFDF--------------

AT5G64790 TPTRPE--RLEVYLFGFLDEDM-KSILPGPFERHWGIFRY-DGTPKFMLDF--------------

AT5G20560 TPKRPN-SRIDAYIFETYNENQ----KPVGIYQHFGLYDP------Y------------------

PtGHL17_1 -----------------------------------------------STSNQITGNNSDFCVAKP

PtGHL17_2 -----------------------------------------------STSNRITGNNSDFCVAKP

PtGHL17_3 --------------------------------------------TGAGTLLANDTTNQTFCVAKE

PtGHL17_4 --------------------------------------------TGSGLVLANDTTNQTYCTAKQ

PtGHL17_5 --------------------------------------------SGSGTFLANDTTNQTYCIVMD

PtGHL17_6 --------------------------------------SGKGVLDVPANKSLTSFNGTTWCIASN

PtGHL17_7 ---------------------TLEQLGNGQSMPVNKSNSPAPSVQSGGDVSTTSSVGQTWCVANG

PtGHL17_8 ---------------------TVEGLKNYKAPSRSPVSGGQQVSAPVRGGVSKSTTGNTWCVANP

PtGHL17_9 ---------------------TVEGLKSYKDSNRSSDTGSHQVAAPVNGGVSKSTTGKTWCVANP

PtGHL17_10 --------------------------------LRPKAKSSIPTSPAPVSPGPGGPKEKIWCLPKP

PtGHL17_11 -----------------------------------SKSSLPPSTPKTPVTPSPKPTKADWCVPKA

PtGHL17_12 --------------------------------------SKSSLTPSTPVTSSPKPATVGWCVPKT

PtGHL17_13 ----------------------------------------MRNGQSSRPTPPSPTKSKKWCVPKA

PtGHL17_14 -----------------------------------LRNGQRGSGRGGGRQRPRPTPGKQWCVPKP

PtGHL17_15 ---------------------------------SGKTPLSGYKKPLPLPTNNEPYKGKLWCIVAK

PtGHL17_16 -------------------------------LRDDKALAPTPGTAADAPSSSSPGRQRQWCVPKS

PtGHL17_17 -----------------------------------------------------------------

PtGHL17_18 -----------------------------------------------------------------

PtGHL17_19 -----------------------------------------------------------------

PtGHL17_20 -----------------------------------------------------------------

PtGHL17_21 -----------------------------------------NGTDAISTNSTPTTAATTTTSPAP

PtGHL17_22 --------------------------------SCQFCSSNGGTLGFGEKMSSGVRGPSVWCVAKP

PtGHL17_23 -----------------------------------------------------------------

PtGHL17_24 -----------------------------------------------------------------

PtGHL17_25 -----------------------------------------------------------------

PtGHL17_26 -----------------------------------------------------------------

PtGHL17_27 --------------------------------STSSQVRTKFTCISLQHHLQSRTNKATWCVPKS

PtGHL17_28 -----------------------------------SKSSQTPATPKTPTNPSPTSKKATWCVPKS

PtGHL17_29 ---------------------------------------------------STSSQIKSLAAASQ

PtGHL17_30 -----------------------------------------------------------------

PtGHL17_31 -------------------------------------------------STSSQVRTKFTCISLI

PtGHL17_32 TKSPFTGPSTSPTKRPPTGSSSSPTKSPSTGSSPSTDSSPSPTTSPSTGSSPAPTTSKTWSEPKA

PtGHL17_33 -----------------------------------------------------------------

PtGHL17_34 -----------------------------------------------------------------

PtGHL17_35 ---------------------------------------GTTNSNGLVPARGVSYLAKKWCIMSP

PtGHL17_36 ----------------------------------------GLGNKLLKNAKNVEYLPSRWCVADP

PtGHL17_37 -----------------------------------------------------------------

PtGHL17_38 -----------------------------------------------------------------

PtGHL17_39 ----------------------------------------GQGSKNLVNAQYVEYLSSKWCVVNN

PtGHL17_40 --------------------------------------SGQNQNKFLAGARNVQYLPAKWCMFNP

PtGHL17_41 ---------------------------------------GTTNTGALIPARSVHYLERKWCVMKP

PtGHL17_42 ---------------------------------------VTANSKGLVPARDVHYLARQWCIMSP

PtGHL17_43 -----------------------------------------------------------------

PtGHL17_44 -----------------------------------------------------------------

PtGHL17_45 -----------------------------------------------------------------

PtGHL17_46 -----------------------------------------GNGKPLVPAKGVRYLARQWCVMSP

PtGHL17_47 --------------------------------------SGKGNDKMLIAAKGVQYMSPRWCVLNE

PtGHL17_48 -------------R---------------------------------------------------

PtGHL17_49 -----------------------------------------------------------------

PtGHL17_50 -----------------------------------------------------------------

PtGHL17_51 -----------------------------------------------------------------

PtGHL17_52 -----------------------------------------------------------------

PtGHL17_53 --------------------------------------MGLEEDKGLAPVEGVRYQLRRWCVLDP

PtGHL17_54 -----------------------------------------------------------------

AT3G57270.1 -----------------------------------------------------------------

AT3G57260.1 -----------------------------------------------------------------

AT3G57240.1 -----------------------------------------------------------------

AT4G16260.1 -----------------------------------------------------------------

AT5G56590.1 -------------------------------------TGKSNGFHSNSSGTNSSGSSNSWCIASS

AT1G77790.1 ----------------------------------------------------------KWR----

AT1G77780.1 -----------------------------------------------------------WNIART

AT4G26830.1 ------------------------------------TKKSTTPVNGNRGKVPVTHEGHTWCVSNG

AT4G29360 --------------TGENTTPVSPTNSTTGTSPSPSSSPIINGNSTVTIGGGGGGGTKKWCIASS

AT3G07320 ------------------------------------SGKTEYKESLPAPENNDLYKGKIWCVVAK

AT2G16230 --------------------------------------TKTTSSQTSQSPQLGKVTSMGWCVPKE

AT5G20340 -----------------------------------------------------------------

AT2G05790 --------------------TTEGLKHYRDGGHTPVTGGDQVTKPPMSGGVSKSLNGYTWCVANG

AT5G55180.1 ----------------------------------NGKSTPVNDNKEKVVPVKPSLVGQTWCVANG

AT5G20330 -----------------------------------------------------------------

AT2G01630 --------------------------------------------AGAGAILANDTTNQTFCIAKE

AT4G34480 -----------------------------------------AKSSSSSQTPSGKVTSSGWCVPKK

AT1G66250 --------------------------------------------TNSGSVLANDTTNQTYCTARE

AT5G20390 -----------------------------------------------------------------

AT3G61810 -----------------------------------------------------------------

AT1G32860 -----------------------------------------------------------------

AT2G27500 -----------------------------------------------------------------

AT1G33220 -----------------------------------------------------------------

AT3G15800 -----------------T------------------------------------------GLKSS

AT3G23770 ----------------------------------SGRRSFSGFDSLPKPSNNVPFKGNVWCVAVD

AT5G42720 --------------------------------------TKTTNYNQTSMAPLSPTRPRLPPAAAP

AT2G26600 -----------------------------------------------------------------

AT4G14080.1 ----------------------------------TGQTPLTGFNPLPKPTNNVPYKGQVWCVPVE

AT3G46570 -----------------------------------------------------------------

AT4G18340 -----------------------------------------------------------------

AT1G30080 -----------------------------------------------------------------

AT5G42100 -----------------------------------------------------------------

AT5G58090 ---------------------------------------GTTNTGALIQAKGVRYLERKWCVMKP

AT1G11820 --------------------------------------------SGSGTFLANDTTNQTYCIAMD

AT3G24330 --------------------------------------SGKGNDKPLVPVEDVKYLPKTWCILDP

AT4G31140.1 -----------------------------------------GSGNGLIPAKDVHYLAKKWCILAP

AT4G17180 ----------------------------------------GLGNRGLKNAKNVQYLPSRWCVAHP

AT3G13560 ---------------------------------------------SGGSSSAALNGSSMFCVAKA

AT5G24318 ----------------------------------LRPTFRSSDPVYNPRSPVRGSSSKRWCVTKA

AT2G39640 -----------------------------------------LKGGGSPLPFPPINNNGKWCVGKP

AT2G19440 --------------------------------------SGQGQNKLLIGAENVTYQPKKWCMFNT

AT3G04010 --------------------------------------RGQGQKKFLTGAQNVQYFLNQWCMFNP

AT5G18220 --------------------------------------SGAGQKKILTGAQNVQYLRNQWCMFNP

AT1G64760 --------------------------------------SGQGQSKFLIGAQNVPYLPNKWCTFNP

AT5G58480 ------------------------------------------NHKNQVNAQNVQYLPPKWCVVNN

AT3G55430 -------------------------------------LRNGQGGGGRPALPAPSTAGGKWCVARS

AT5G20870 -----------------------------------------GNGRPLVPTKGVRYQAREWCVLST

AT3G55780 -----------------------------------------------------------------

AT5G64790 --------------------------------------TGQGRQMVPVAAKGVQYLEKQWCVVNK

AT5G20560 -----------------------------------------------------------------

PtGHL17_1 N-----ADPGKLQAGLDWACGQGGANCDAIQ-EGKPCYLPNTYQNHASYAYNDYYKKKRSVGATC

PtGHL17_2 N-----ADPGKLQV------------MDPVNLLGVQLPLRTQLEDSHHLWHLDLCLLKEGAG---

PtGHL17_3 G-----ADPKMLQAALDWACGPGKVDCSFLL-QGQPCYEPDNVVAHSTYAFNAYFQKMAKSPGTC

PtGHL17_4 G-----ADPKMLQAALDWACGPGKVDCSAML-QGEPCYQPDNVIAHATYAFNSYYNQMGKAPGTC

PtGHL17_5 G-----VDSKTLQAALDWACGPGQANCSEIQ-PGENCYQPNNVKNHASYAFDSYYQKEGRAAGSC

PtGHL17_6 N-----ASQLDLQNALDWACGSGDVDCSAIQ-PSQPCFEPDTLVSHASYAFNSYYQQNGASDVAC

PtGHL17_7 N-----AGAEKLQAGLDYACGEGGADCRPIQ-TGSTCYNPNTVEAHASYAFNSYYQKKARGAGTC

PtGHL17_8 D-----AGKEKLQAALDFACGEGGADCRPIQ-PDATCYSPNTLVAHSSFAFNSYYQKKGRGMGDC

PtGHL17_9 D-----AGKQKLQAGLDFACGEGGADCRPIQ-PGATCYDPNTLVAHSSFAFNSYYQKQGRGMGDC

PtGHL17_10 G-----ADVEALQRNIDYVCGLEAEYCKPIQ-EGGECFMPNTVKAHAAFAMNAYYQGTEKNGYDC

PtGHL17_11 G-----VSDAQLQASLDYACGQG-IDCGPIQ-PGGACFEPNTVASHASYAMNLYYQKSAKNPWNC

PtGHL17_12 G-----ASEAQLQASLDYACGQG-IDCGPIQ-PGGACFIPDTLASHAAYAINLYFQASAKSPWNC

PtGHL17_13 D-----ATDKALQANIDYVCSQG-MDCKPIQ-AGGACFSPNNIRSHASYIMNSYYQSHGSNDFNC

PtGHL17_14 G-----VSDQALQANIDYACSQG-VDCKPIQ-PGGACFDPNNVRSHASYVMNFFYQTHGRQAFNC

PtGHL17_15 E-----ANRSAVKDALAWACSQGNKTCDEIQ-PGKGCYKPVSLFWHASYAFSSYWAEFKKIGGVC

PtGHL17_16 D-----ASDDALQKNIDYVCSNG-VDCKPIQ-QGGPCFVPDTVKSHASYAMNAFYQASGRHDYDC

PtGHL17_17 ---------------------------SALSSPSTPSASISLTSSATKVKT--------------

PtGHL17_18 ----------------------SGTDAQAIQ---SNCFQPGHV----------------------

PtGHL17_19 ----------------SALSSPSSTSSASISDLASSATKVKT-----------------------

PtGHL17_20 -------------TGLKDSSASSLIPFKGIG--GSSCTLLLTTCITLLLLISAS-----------

PtGHL17_21 R-----SPESSSTGYLSISAAVKGRWISCIG------QLLFPLLLLSCLAFRLAF----------

PtGHL17_22 H-----ADEKVLQAVLDFCCGPGGVDCREIY-ESGDCFAPDKLHAHASYAMNVYYQIHGRNYWNC

PtGHL17_23 ---------TGLKESSGVSSFIHFKVIGAYGWLGSSCALLLTTCITLVLLISAS-----------

PtGHL17_24 -----------------------QGNLPGIMYSSASNINVTHILSHHHHVLV-------------

PtGHL17_25 ------HGLSSAESSL--------LSLKAVQSQGLTKSYILILTISAAALILFLKQ---------

PtGHL17_26 -----------------------------QGYLPGIVYYSSASTINVSHILSYHY----------

PtGHL17_27 G-----VSDAQLQDNLDFAYGRG-IDHDPIQ-PGGACFEPNTIASLT------------------

PtGHL17_28 G-----VSDAQLQDNLDYACGRG-IDCSPIE-PGGACFEPNTLASHAAYAMNLFYQASDKNPLNC

PtGHL17_29 PLIAATTNTINNNNSMSMNTSTCNCMCNCTS-TINISSGSNKVYLIIIFNLDFLYGFMGLSLICL

PtGHL17_30 ------STSNQVRT---------KFMCISLV--------------FRVFNSQVFYS---------

PtGHL17_31 WHLNFYVF--------GWEHGFG------------------------------------------

PtGHL17_32 D-----ASDEALQANKDYVCSQG-AHCKPIQ-TGGACFNPNNIRSHAALLIPLIMMWFVYI----

PtGHL17_33 ----------------------------------STSSQAYSVRKLTSF----------------

PtGHL17_34 ----------------------------------STSSQVRTKFMCISLVFRVFNSQVFYS----

PtGHL17_35 SAS---LDDPQVAPSVSYACASA--DCTSLG-YGTSCGDLSA-QGNISYAFNSYYQQNNQLESAC

PtGHL17_36 S-----KDLTSVANHLRIACSAA--DCTTLN-YGGSCNEIGA-KGNISYAFNSYYQLQMQNAQSC

PtGHL17_37 ------TAATNTSTSTTTSNNNSTTSTSTSTGTGPSHTAGQP-----------------------

PtGHL17_38 -----------------WQCS--------------------------------------------

PtGHL17_39 N-----KDLSNATASALDACSTA--DCSALS-PGGSCFNI-SWPANISYAFNNYYQVHDQRADSC

PtGHL17_40 NA----KDLSKLAENIDYACSRS--DCTALG-YGSSCNSLDS-NGNASYAFNMYYQVQNQDEFAC

PtGHL17_41 SAK---LDDPQVAPSVSYACGLA--DCTSLG-YGTSCGNLDP-RENISYAFNSYFQIQNQLGDAC

PtGHL17_42 SAS---LDDPQVGPSVSYACANA--DCTSLG-YGTSCENLDA-RGNISYAFNSYYQQNNQLESAC

PtGHL17_43 -----------------------------------------------------------------

PtGHL17_44 -----------------W-----------------------------------------------

PtGHL17_45 ------------------------------G----------------------------------

PtGHL17_46 EAS---ISDPNLPNSIAYACNYA--DCTSLG-YGSSCGTLDA-KSNASYAFNMYYQTVDQRNGAC

PtGHL17_47 EN----KNLSMIADEISYACSSA--DCTSLG-YGSSCSKMDI-DGNVSYAFNMYFQMQDQGDYAC

PtGHL17_48 -----------------------------------------------------------------

PtGHL17_49 ------------GGHRNWDISTE---------QNATVSLKSDM----------------------

PtGHL17_50 -----------------WR----------------------------------------------

PtGHL17_51 ------------------------------N----------------------------------

PtGHL17_52 --------------S--------------------------------------------------

PtGHL17_53 EA----TELAELPDSIDYACSQS--DCTALG-YGSSCNHLSA-EGNASYAFNMYYQLNNQGYWDC

PtGHL17_54 ---------------------QDHFPTNGVV-YSSAVSNINALSVFSLLIFVMAYVILV------

AT3G57270.1 --------------Y--------------------------------------------------

AT3G57260.1 ------------------------------N----------------------------------

AT3G57240.1 ------------------------------N----------------------------------

AT4G16260.1 ---------------------SAARDGTAVE----------------------------------

AT5G56590.1 K-----ASERDLKGALDWACGPGNVDCTAIQ-PSQPCFQPDTLVSHASFVFNSYFQQNRATDVAC

AT1G77790.1 -----------------------------------------------------------------

AT1G77780.1 S-----------NASPSWVIWTN-------------------WVIWTMLITRLFY----------

AT4G26830.1 E-----VAKEKLQEALDYACGEGGADCRPIQ-PGATCYHPESLEAHASYAFNSYYQKNSRRVGTC

AT4G29360 Q-----ASVTELQTALDWACGPGNVDCSAVQ-PDQPCFEPDTVLSHASYAFNTYYQQSGASSIDC

AT3G07320 G-----ANWTQLGDALSYACSQGNNTCDPIQ-RGGPCQKPDLTVLHASYAFSSYWAQFRKIGGTC

AT2G16230 D-----ATQEQLQDSLDWVCGQG-IDCGPIM-PGGVCFEPNNVASHTAYAMNLYFQKSPENPTDC

AT5G20340 -----------------------------------------------------------------

AT2G05790 D-----AGEERLQGGLDYACGEGGADCRPIQ-PGANCYSPDTLEAHASFAFNSYYQKKGRAGGSC

AT5G55180.1 K-----TTKEKLQEGLDYACGEGGADCRPIQ-PGATCYNPESLEAHASYAFNSYYQKNARGVGTC

AT5G20330 -----------------------------------------------------------------

AT2G01630 K-----VDRKMLQAALDWACGPGKVDCSALM-QGESCYEPDDVVAHSTYAFNAYYQKMGKASGSC

AT4G34480 G-----ATNEELQASLDWACGHG-IDCGAIQ-PGGACFEPNNVVSHAAYAMNMYFQKSPKQPTDC

AT1G66250 G-----ADTKMLQAALDWACGPGKIDCSPIK-QGETCYEPDNVVAHANYAFDTYYHQTGNNPDAC

AT5G20390 -----------------------------------------------------------------

AT3G61810 --------------------------SPPI-----------------------------------

AT1G32860 ----------ALNDVVRGASGGGTGGGNSSSGGGRDKSPVFPVSPVAPDSASTGYLAISASPVTG

AT2G27500 ---------QGYLPDIIYTS------------RATTIKILNLWRVVMGLAVAWFILDMGDKMRMR

AT1G33220 -----------------------------------------------------------------

AT3G15800 S-----ATRYRFKSSLVSAS---AFTCLLLL-FHRLFHL--------------------------

AT3G23770 G-----ADEAELGQALNFACGRSNATCAALA-PGGECYAPVTVTWHASYAFSSYWAQFRNQSSQC

AT5G42720 TRQTLPSPPQMILPSPVTPSDKNSGQTDVHN-STPRSASLAHICRSLSISASMFFVSVLYALIIL

AT2G26600 ------NNLKSDSPKSLISSSKSARYYVALV-ISVSAFLLMI-----------------------

AT4G14080.1 G-----ANETELEETLRMACAQSNTTCAALA-PGRECYEPVSIYWHASYALNSYWAQFRNQSIQC

AT3G46570 ------------------------------------------MKTNKVYTLQLL-----------

AT4G18340 -----------------STMSTTTANSESVTYSSSATKAKRSLEYWTILILAMVQVVMLRLF---

AT1G30080 ------------LSSSSLTSTSTTSSTSIISLTSSASTALKKGKQRLMYWTCVYLLAIHMLIRRS

AT5G42100 ------KTSSTHSSGSGSSNSTGGSSSGGGGNTGGSSSGGGIYQPVTGNPSPDYMSISSAGGKGR

AT5G58090 NVR---LDDPQVAPAVSYACSLG--DCTSLG-VGTSCANLDG-KQNISYAFNSYYQIQDQLDTAC

AT1G11820 G-----VDAKTLQAALDWACGPGRSNCSEIQ-PGESCYQPNNVKGHASFAFNSYYQKEGRASGSC

AT3G24330 NA----YNLDDLPDNIDYACSLS--DCTALG-YGSSCNHLTA-TGNVSYAFNMYYQMHDQKTWDC

AT4G31140.1 NAN---LQDPQLGPSVSYACDHA--DCTSLG-YGSSCGNLNL-AQNVSYAFNSYYQVSNQLDSAC

AT4G17180 S-----RDMTQVGDHLRLACSEA--DCTTLN-DGGSCSQLGE-KDNISYAFNSYYQMQMQHEKSC

AT3G13560 D-----ADDDKLVDGLNWACGQGRANCAAIQ-PGQPCYLPNDVKSHASFAFNDYYQKMKSAGGTC

AT5G24318 G-----AETVALQRNIDYVCGLG-LDCRPIN-EGGLCYLPNTVKAHSKYAMNLYYQTMEKHEFDC

AT2G39640 E-----ATLMQLQANIDWVCSHG-IDCTPIS-PGGICFDNNNMTTRSSFIMNAYYQSKGCVDVVC

AT2G19440 EA----KDLTKLAANIDYACTFS--DCTALG-YGSSCNTLDA-NGNASYAFNMYFQVKNQDEDAC

AT3G04010 NGR---GNMSRLGDNINYACSHS--DCTALG-YGSSCGNLDA-NGNASYAFNMYFQVQNQEAQAC

AT5G18220 NALTFSNNTNQLGDNVNYACTFS--DCTALG-YGSSCGNLDE-VGNASYAFNMYFQVQNQKAEAC

AT1G64760 EA----KDLTKLAANIDYACTFS--DCTALG-YGSSCNTLDA-NGNASYAFNMFFQVKNQDESAC

AT5G58480 N-----KDLSNASARALEACAVA--DCTSIL-PGGSCSGI-RWPGNVSYAFNSLYQQNDHSAESC

AT3G55430 G-----ATNTQLQDSINWVCGQG-VDCKPIQ-AGGSCFNPSSLRTHASFVMNAYFQSHGRTDGAC

AT5G20870 QAAG--NGAATWQASATYACQNA--DCTSLG-PGSSCAALDP-TANASYAFNMYFQKMDHRRGSC

AT3G55780 ------SDGGKVRRFKEILVGFF-VQVVMIG-YGYVCRYFETIIWYVVNFMNNYECLDTRKTLIC

AT5G64790 DT----VNLDEVGPDLDYACYHG--DCTAME-AGSTCSKLTK-VQNISYAFNMYFQIQDQDVRAC

AT5G20560 -----------------------------------------------------------------

PtGHL17_1 D-FDGTAATTTVDPSYGSCKFTGSSTITPNSNGGFTTTVAPGPVTPQGGSATVNLPVSKIQFLIS

PtGHL17_2 ---------------LRTCKF--------------------------------------------

PtGHL17_3 D-FKGVATITTSDPSHGSCIFPGSAGRNGTFPNTTSLAPSSNSSTSGCHSVYFYGAGSFTTSVII

PtGHL17_4 D-FNGVAAITTTNPSHGTCVFPGSTGRMNGTMVNITAPSMNSTSAAPSARDLYNLGSTNFLVLLR

PtGHL17_5 D-FKGVAMITTTDPSHGSCIFPGSKKITNKTRTVVNTTTPSNAAGGSRLISFRSSRISAMDKALQ

PtGHL17_6 S-FGGTGVKVNKDPSYDNCMYMITGTNKTAASNTTAIASTSSSAQNEACAWISSFLLMTCVVYFL

PtGHL17_7 D-FGGAAYVVTQQPRFGNCKFPTGY----------------------------------------

PtGHL17_8 Y-FGGAAFVVTQEPKFGVCEFPTGY----------------------------------------

PtGHL17_9 Y-FGGAAYVVTQEPKFGQCEFPTGY----------------------------------------

PtGHL17_10 D-FEQTAAISNVNPSYGNCKY--------------------------------------------

PtGHL17_11 D-FSETATLTFKNPSYNGCTYPGGSA---------------------------------------

PtGHL17_12 D-FSETATLTSKNPSYNGCTYPGGST---------------------------------------

PtGHL17_13 D-FSQTAVLTTSDPSHGTCKYN-------------------------------------------

PtGHL17_14 D-FSNTGVLTAVNPGHGTCRFV-------------------------------------------

PtGHL17_15 S-FNGLATTTVKDPSFGQCKFPSVTL---------------------------------------

PtGHL17_16 D-FSHTGVLTSIDPSKLSWPSSSPR----------------------------------------

PtGHL17_17 -----------------------------------------------------------------

PtGHL17_18 -----------------------------------------------------------------

PtGHL17_19 -----------------------------------------------------------------

PtGHL17_20 -----------------------------------------------------------------

PtGHL17_21 -----------------------------------------------------------------

PtGHL17_22 D-FKGTGLVTFSDPSYGTCKYSQQ-----------------------------------------

PtGHL17_23 -----------------------------------------------------------------

PtGHL17_24 -----------------------------------------------------------------

PtGHL17_25 -----------------------------------------------------------------

PtGHL17_26 -----------------------------------------------------------------

PtGHL17_27 --LPEIHLIPTELPTE-------------------------------------------------

PtGHL17_28 D-FSQSATLSSNNPSYNACTYPSGSGSN-------------------------------------

PtGHL17_29 F-FYDLQT---------------------------------------------------------

PtGHL17_30 -----------------------------------------------------------------

PtGHL17_31 -----------------------------------------------------------------

PtGHL17_32 -----------------------------------------------------------------

PtGHL17_33 -----------------------------------------------------------------

PtGHL17_34 -----------------------------------------------------------------

PtGHL17_35 RFPNNLSVVTSNDPSTGTCKFMIMIQSQAVTSGVEGGRGFSMSSFVTFAVAFLSAFL--------

PtGHL17_36 D-FDGLGMVTFLDPSVGDCRFPVGVNDHKSSLAFRSCHRWTTIWTSILGGVWFFLML--------

PtGHL17_37 -----------------------------------------------------------------

PtGHL17_38 -----------------------------------------------------------------

PtGHL17_39 D-FGGLGLITTVDPSVGNCRFPVELRTSHSESLYGTCLLQWMILLTINTILHDFL----------

PtGHL17_40 N-FEGLATLTNQNISQGNCNFIIQIVASSSSSLTLSLMAFVTVLLTFLFL---------------

PtGHL17_41 K-FPNLSTITRTDPSTSTCRFAIMIEPYYGGAGQTFRYGKKVALGGLIALFFLTIV---------

PtGHL17_42 KFPNNLSVVINNDPSTGTCKFRIMIQGQAVISGAGGGKGFSRSSLVVFILVFFPLYFCDFLGLKL

PtGHL17_43 D----------------------------------------------------------------

PtGHL17_44 -----------------------------------------------------------------

PtGHL17_45 -----------------------------------------------------------------

PtGHL17_46 S-FSNLSTLTKVDPSQNPCRFEIMMDLGKHETPPRRSFAGGKENPAAMMAFISALILIICGAY--

PtGHL17_47 N-FNGLAMIVKTNASRGNCLFPLQLVGAGERLELAYGVRIIAGLMLAFFSLM-------------

PtGHL17_48 -----------------------------------------------------------------

PtGHL17_49 -----------------------------------------------------------------

PtGHL17_50 -----------------------------------------------------------------

PtGHL17_51 -----------------------------------------------------------------

PtGHL17_52 -----------------------------------------------------------------

PtGHL17_53 D-FSGLALVTDKDPSEEDCQFPVMIAYGHSLVLNRNGLSDVLLGIVVGNLLFLLLLS--------

PtGHL17_54 -----------------------------------------------------------------

AT3G57270.1 -----------------------------------------------------------------

AT3G57260.1 -----------------------------------------------------------------

AT3G57240.1 -----------------------------------------------------------------

AT4G16260.1 -----------------------------------------------------------------

AT5G56590.1 S-FGGAGVKVNKDPSYDKCIYITAGGNKTKATNATALTSSASTPRGNELLQWILKLCLMISLFFS

AT1G77790.1 -----------------------------------------------------------------

AT1G77780.1 -----------------------------------------------------------------

AT4G26830.1 F-FGGAAHVVTQPPRYGKCEFPTGH----------------------------------------

AT4G29360 S-FNGASVEVDKDPSYGNCLYMIAPATDGFNRTMAGNITGNITAIDSPLASPSSTNEAFRQMVVA

AT3G07320 S-FNGLATQTIKDPSYGRCEFPSVTL---------------------------------------

AT2G16230 D-FSKTARITSENPSYSSCVYPRAGDGSITGEVTKYVTSDKATEKNGSECFSSLYLARFIISIYF

AT5G20340 -----------------------------------------------------------------

AT2G05790 Y-FGGAAYVVSQPPKYGRCEFPTGY----------------------------------------

AT5G55180.1 N-FGGAAYVVSQPPSKVWEMRVSNRALKWI-----------------------------------

AT5G20330 -----------------------------------------------------------------

AT2G01630 D-FKGVATVTTTDPSRGTCVFPGSAKSNQTLGNNTSALAPSANSTTSGCIPKYYHHPHASFGDLT

AT4G34480 D-FSKTATVTSQNPSYNNCVYPGGGGGGGGGGGGSKAVMNKYVSSDKVEKKNGATEPKVSSSLSF

AT1G66250 N-FNGVASITTTDPSHGTCVFAGSRGNGRNGTSVNITAPSANSTTSSGIRSDLYYSRGIWSILTV

AT5G20390 -----------------------------------------------------------------

AT3G61810 -----------------------------------------------------------------

AT1G32860 K-RKGKGAILSLVVSMLLARHLL------------------------------------------

AT2G27500 -----------------------------------------------------------------

AT1G33220 -----------------------------------------------------------------

AT3G15800 -----------------------------------------------------------------

AT3G23770 Y-FNGLARETTTNPGNERCKFPSVTL---------------------------------------

AT5G42720 L----------------------------------------------------------------

AT2G26600 -----------------------------------------------------------------

AT4G14080.1 F-FNGLAHETTTNPGNDRCKFPSVTL---------------------------------------

AT3G46570 -----------------------------------------------------------------

AT4G18340 -----------------------------------------------------------------

AT1G30080 Y----------------------------------------------------------------

AT5G42100 F-------VECVLFFFLLCIIKLRL----------------------------------------

AT5G58090 K-FPNISEVTKTDPSTGTCRFPIMIEPYYGGAAREHGFFFPLLMVAAIAVSIF------------

AT1G11820 D-FKGVAMITTTDPSHGSCIFPGSKKVGNRTQTVVNSTEVAAGEATSRSLSRGFCVTIMILVTFS

AT3G24330 D-FLGLGLITDEDPSDELCEFPVMIDTGDSTRLQPGSSRVLTRVAAAVLVMLVLPIL--------

AT4G31140.1 K-FPGLSIVSTRDPSVGSCKFKIMIKSEDASEASAMMPITRSTAVLLLLSICLYIVL--------

AT4G17180 -----------------------------------------------------------------

AT3G13560 D-FDGTAITTTRDPSYRTCAYTGSLNANATNGNFPPDALGPASPLGGNANARIIFSYHLPILAPL

AT5G24318 D-FDNTGEITTIDPSYGNCEYQAN-----------------------------------------

AT2G39640 D-FSGTGIVTSTNPSTSTCPIPIGEGGGGNGAKSKSANWCMAKQEATETQLQANIDWVCSQGIDC

AT2G19440 I-FQGLATITTKNISQGQCNFPIQIVASTASSFSSSLVLLIAGVWFLLSGVMFEV----------

AT3G04010 D-FEGLATITTQNISQGQCNFPIQIGEPSSGHYDYSYGSMVRLCLVMSGLVFLLI----------

AT5G18220 D-FEGLAIITTRNISREQCNFPIQIGDPTSGHSDHNHRFISFSLVSMLLLFTAL-----------

AT1G64760 Y-FQGLATITTQNISQGQCNFPIQIVASSASSFSCSSYSLVVLIVWFLLSGMMF-----------

AT5G58480 N-FGGLGLITTVDPSEDNCRFSIQLDTSHSSSQTPNFFQSWPLLLLFLLSGLF------------

AT3G55430 N-FSGTGMIVGNNPSNGACKY--------------------------------------------

AT5G20870 D-FNNLGVVTKIDPSSGSCRFPIEIDTSRQQMSNPPPRNSGASEAKYRLGAAVVMVICLVFSN--

AT3G55780 DDYSSGNGILGLTHRNYRS----------------------------------------------

AT5G64790 D-FKGAAMITKVNASVGSCLFPVQIVSGSDDFRINFVFGRFVVFGLVLLGLLTVI----------

AT5G20560 -----------------------------------------------------------------

PtGHL17_1 AVFLALVLL------------------------------------------------------

PtGHL17_2 ---------------------------------------------------------------

PtGHL17_3 GVLLMSMVCL-----------------------------------------------------

PtGHL17_4 VLICSIVFL------------------------------------------------------

PtGHL17_5 AFLTIIFSILLHIPFVVS---------------------------------------------

PtGHL17_6 SFEH-----------------------------------------------------------

PtGHL17_7 ---------------------------------------------------------------

PtGHL17_8 ---------------------------------------------------------------

PtGHL17_9 ---------------------------------------------------------------

PtGHL17_10 ---------------------------------------------------------------

PtGHL17_11 ---------------------------------------------------------------

PtGHL17_12 ---------------------------------------------------------------

PtGHL17_13 ---------------------------------------------------------------

PtGHL17_14 ---------------------------------------------------------------

PtGHL17_15 ---------------------------------------------------------------

PtGHL17_16 ---------------------------------------------------------------

PtGHL17_17 ---------------------------------------------------------------

PtGHL17_18 ---------------------------------------------------------------

PtGHL17_19 ---------------------------------------------------------------

PtGHL17_20 ---------------------------------------------------------------

PtGHL17_21 ---------------------------------------------------------------

PtGHL17_22 ---------------------------------------------------------------

PtGHL17_23 ---------------------------------------------------------------

PtGHL17_24 ---------------------------------------------------------------

PtGHL17_25 ---------------------------------------------------------------

PtGHL17_26 ---------------------------------------------------------------

PtGHL17_27 ---------------------------------------------------------------

PtGHL17_28 ---------------------------------------------------------------

PtGHL17_29 ---------------------------------------------------------------

PtGHL17_30 ---------------------------------------------------------------

PtGHL17_31 ---------------------------------------------------------------

PtGHL17_32 ---------------------------------------------------------------

PtGHL17_33 ---------------------------------------------------------------

PtGHL17_34 ---------------------------------------------------------------

PtGHL17_35 ---------------------------------------------------------------

PtGHL17_36 ---------------------------------------------------------------

PtGHL17_37 ---------------------------------------------------------------

PtGHL17_38 ---------------------------------------------------------------

PtGHL17_39 ---------------------------------------------------------------

PtGHL17_40 ---------------------------------------------------------------

PtGHL17_41 ---------------------------------------------------------------

PtGHL17_42 KVYGLGFCDF-----------------------------------------------------

PtGHL17_43 ---------------------------------------------------------------

PtGHL17_44 ---------------------------------------------------------------

PtGHL17_45 ---------------------------------------------------------------

PtGHL17_46 ---------------------------------------------------------------

PtGHL17_47 ---------------------------------------------------------------

PtGHL17_48 ---------------------------------------------------------------

PtGHL17_49 ---------------------------------------------------------------

PtGHL17_50 ---------------------------------------------------------------

PtGHL17_51 ---------------------------------------------------------------

PtGHL17_52 ---------------------------------------------------------------

PtGHL17_53 ---------------------------------------------------------------

PtGHL17_54 ---------------------------------------------------------------

AT3G57270.1 ---------------------------------------------------------------

AT3G57260.1 ---------------------------------------------------------------

AT3G57240.1 ---------------------------------------------------------------

AT4G16260.1 ---------------------------------------------------------------

AT5G56590.1 LQTMNSQAL------------------------------------------------------

AT1G77790.1 ---------------------------------------------------------------

AT1G77780.1 ---------------------------------------------------------------

AT4G26830.1 ---------------------------------------------------------------

AT4G29360 VSVLLPCFVVCSSIW------------------------------------------------

AT3G07320 ---------------------------------------------------------------

AT2G16230 FCLFPSLRIM-----------------------------------------------------

AT5G20340 ---------------------------------------------------------------

AT2G05790 ---------------------------------------------------------------

AT5G55180.1 ---------------------------------------------------------------

AT5G20330 ---------------------------------------------------------------

AT2G01630 LLSLLLIIALVFL--------------------------------------------------

AT4G34480 LLIFLSLIFHVYM--------------------------------------------------

AT1G66250 MILNVANIL------------------------------------------------------

AT5G20390 ---------------------------------------------------------------

AT3G61810 ---------------------------------------------------------------

AT1G32860 ---------------------------------------------------------------

AT2G27500 ---------------------------------------------------------------

AT1G33220 ---------------------------------------------------------------

AT3G15800 ---------------------------------------------------------------

AT3G23770 ---------------------------------------------------------------

AT5G42720 ---------------------------------------------------------------

AT2G26600 ---------------------------------------------------------------

AT4G14080.1 ---------------------------------------------------------------

AT3G46570 ---------------------------------------------------------------

AT4G18340 ---------------------------------------------------------------

AT1G30080 ---------------------------------------------------------------

AT5G42100 ---------------------------------------------------------------

AT5G58090 ---------------------------------------------------------------

AT1G11820 IL-------------------------------------------------------------

AT3G24330 ---------------------------------------------------------------

AT4G31140.1 ---------------------------------------------------------------

AT4G17180 ---------------------------------------------------------------

AT3G13560 ALTLLQLLLQHDRLL------------------------------------------------

AT5G24318 ---------------------------------------------------------------

AT2G39640 KPISPGGICFDNNNMKTRSTFIMNAYYESKGYSKDACDFRGSGIVTTTNPSTSTCVVPASVTL

AT2G19440 ---------------------------------------------------------------

AT3G04010 ---------------------------------------------------------------

AT5G18220 ---------------------------------------------------------------

AT1G64760 ---------------------------------------------------------------

AT5G58480 ---------------------------------------------------------------

AT3G55430 ---------------------------------------------------------------

AT5G20870 ---------------------------------------------------------------

AT3G55780 ---------------------------------------------------------------

AT5G64790 ---------------------------------------------------------------

AT5G20560 ---------------------------------------------------------------

;

end;
