# Supplementary material for: A phylogenetic approach to study the origin and evolution of plasmodesmata-localized glycosyl hydrolases family 17
Source: Front Plant Sci. 2014 May 23;5:212. doi: 10.3389/fpls.2014.00212 (PMC4033164; doi:10.3389/fpls.2014.00212)
Supplement: Supplementary file 3 [file DataSheet3.DOCX]

GREEN ALGAE, FUNGI, MOSS, ARABIDOPSIS AND RICE TREES (NEWICK FORMAT)

NJ TREE

(((((((ScGHL17_1:100.0,(KlGHL17_1:100.0,CglGHL17_1:100.0):52.0):38.0,AgGHL17_1:100.0):48.0,((DhGHL17_1:100.0,SsGHL17_1:100.0):60.0,CaGHL17_1:100.0):37.0):73.0,YlGHL17_1:100.0):92.0,((SpoGHL17_1:100.0,FgGHL17_1:100.0):67.0,((AfgHL17_1:100.0,AcGHL17_1:100.0):99.0,AnGHL17_1:100.0):100.0):50.0):100.0,(((((((((PpGHL17_16:100.0,PpGHL17_15:100.0):100.0,PpGHL17_14:100.0):100.0,((PpGHL17_10:100.0,PpGHL17_13:100.0):100.0,PpGHL17_12:100.0):100.0):91.0,((((((AT3G04010:100.0,AT5G18220:100.0):100.0,(AT2G19440:100.0,AT1G64760:100.0):100.0):100.0,(((OsGHL17_21:100.0,OsGHL17_13:100.0):97.0,OsGHL17_15:100.0):95.0,OsGHL17_11:100.0):77.0):86.0,((OsGHL17_12:100.0,OsGHL17_16:100.0):52.0,AT5G64790:100.0):59.0):90.0,AT3G24330:100.0):87.0,((AT5G20870:100.0,OsGHL17_14:100.0):100.0,(((OsGHL17_20:100.0,AT5G58090:100.0):51.0,AT4G31140.:100.0):36.0,OsGHL17_19:100.0):99.0):96.0):88.0):38.0,((OsGHL17_24:100.0,AT5G58480:100.0):100.0,((OsGHL17_17:100.0,OsGHL17_18:100.0):100.0,AT4G17180:100.0):100.0):75.0):100.0,NtGHL17_2:100.0):62.0,((PmGHL17_1:100.0,NtGHL17_3:100.0):56.0,KfGHL17_1:100.0):17.0):9.00,(KfGHL17_2:100.0,CgGHL17_1:100.0):38.0):10.0,(((((((((((OsGHL17_7:100.0,OsGHL17_23:100.0):100.0,OsGHL17_22:100.0):66.0,((AT2G16230:100.0,AT4G34480:100.0):100.0,AT5G42720:100.0):58.0):100.0,(((AT3G55430:100.0,AT2G39640:100.0):100.0,AT3G46570:100.0):81.0,AT5G24318:100.0):100.0):81.0,((((AT5G42100:100.0,AT1G32860:100.0):100.0,(((OsGHL17_1:100.0,OsGHL17_3:100.0):100.0,AT2G27500:100.0):52.0,OsGHL17_2:100.0):92.0):93.0,(AT1G30080:100.0,AT4G18340:100.0):100.0):79.0,((OsGHL17_4:100.0,OsGHL17_5:100.0):78.0,(AT3G15800:100.0,AT2G26600:100.0):52.0):100.0):99.0):76.0,(PpGHL17_2:100.0,PpGHL17_1:100.0):100.0):37.0,((AT5G55180.:100.0,AT4G26830.:100.0):100.0,AT2G05790:100.0):100.0):14.0,(((PpGHL17_11:100.0,PpGHL17_9:100.0):100.0,PpGHL17_17:100.0):85.0,PpGHL17_3:100.0):60.0):9.00,(((((AT5G56590.:100.0,OsGHL17_6:100.0):94.0,AT4G29360:100.0):100.0,PpGHL17_8:100.0):42.0,(((((AT1G66250:100.0,OsGHL17_8:100.0):53.0,AT2G01630:100.0):99.0,(OsGHL17_10:100.0,AT1G11820:100.0):99.0):100.0,(AT3G13560:100.0,OsGHL17_9:100.0):99.0):100.0,PpGHL17_7:100.0):55.0):29.0,(PpGHL17_5:100.0,(AT3G55780:100.0,AT3G61810:100.0):97.0):23.0):6.00):21.0,((PpGHL17_4:100.0,(PpGHL17_6:100.0,PpGHL17_18:100.0):84.0):99.0,((AT3G23770:100.0,AT4G14080.:100.0):100.0,AT3G07320:100.0):100.0):89.0):37.0,(((AT5G20390:100.0,(((AT1G33220:100.0,AT5G20340:100.0):78.0,AT5G20330:100.0):100.0,AT5G20560:100.0):66.0):100.0,(AT1G77790.:100.0,AT1G77780.:100.0):100.0):80.0,(((AT3G57240.:100.0,AT3G57260.:100.0):100.0,AT3G57270.:100.0):100.0,AT4G16260.:100.0):99.0):79.0):37.0):30.0):100.0,NtGHL17_1:100.0);

ML TREE

((((((((((((((((OsGHL17_1,OsGHL17_3)1.0000,OsGHL17_2)0.5000,AT2G27500)0.9400,(AT1G32860,AT5G42100)1.0000)0.9900,(AT4G18340,AT1G30080)1.0000)0.7500,((AT3G15800,AT2G26600)0.5500,(OsGHL17_4,OsGHL17_5)0.9700)1.0000)0.9400,((((AT2G39640,AT3G55430)1.0000,AT3G46570)0.7400,AT5G24318)0.9900,(((AT2G16230,AT4G34480)1.0000,AT5G42720)0.8800,(OsGHL17_22,(OsGHL17_7,OsGHL17_23)0.9400)0.8800)1.0000)0.8700)0.4100,(PpGHL17_1,PpGHL17_2)1.0000)0.2400,(PpGHL17_3,(PpGHL17_17,(PpGHL17_9,PpGHL17_11)1.0000)0.7700)0.7200)0.0500,(((AT3G61810,AT3G55780)0.9600,PpGHL17_5)0.1600,((((AT5G56590.1,OsGHL17_6)0.9300,AT4G29360)1.0000,PpGHL17_8)0.4700,(PpGHL17_7,((AT3G13560,OsGHL17_9)1.0000,((AT1G11820,OsGHL17_10)0.9900,(OsGHL17_8,(AT2G01630,AT1G66250)0.4900)1.0000)1.0000)1.0000)0.5800)0.6000)0.0500)0.0700,(((AT4G26830.1,AT5G55180.1)1.0000,AT2G05790)1.0000,(((PpGHL17_6,PpGHL17_18)0.7500,PpGHL17_4)0.9600,(AT3G07320,(AT3G23770,AT4G14080.1)1.0000)1.0000)0.9800)0.1500)0.2800,((((AT3G57260.1,AT3G57240.1)0.9800,AT3G57270.1)0.9800,AT4G16260.1)1.0000,((AT1G77790.1,AT1G77780.1)1.0000,(AT5G20560,(AT5G20390,(AT5G20330,(AT5G20340,AT1G33220)0.4800)0.9600)0.3900)1.0000)0.7900)0.9400)0.5800,((KfGHL17_1,NtGHL17_1)0.3700,(CgGHL17_1,(PmGHL17_1,(NtGHL17_2,((((OsGHL17_17,OsGHL17_18)1.0000,AT4G17180)1.0000,(AT5G58480,OsGHL17_24)1.0000)0.6600,((((PpGHL17_10,PpGHL17_13)0.9800,PpGHL17_12)1.0000,(PpGHL17_14,(PpGHL17_15,PpGHL17_16)0.9800)1.0000)0.9200,(AT3G24330,(((((AT5G58090,OsGHL17_20)0.6800,OsGHL17_19)0.3800,AT4G31140.1)0.8500,(AT5G20870,OsGHL17_14)1.0000)0.9800,(((OsGHL17_12,OsGHL17_16)0.5100,AT5G64790)0.9000,(OsGHL17_11,(((OsGHL17_13,OsGHL17_21)0.8200,OsGHL17_15)0.9700,((AT2G19440,AT1G64760)1.0000,(AT3G04010,AT5G18220)1.0000)1.0000)0.3700)0.9700)0.9800)0.4800)0.8900)0.4600)0.9900)0.7600)0.2800)0.2200)0.0700)0.1000,KfGHL17_2)0.5400,NtGHL17_3)1.0000,((((AcGHL17_1,AfgHL17_1)0.8300,AnGHL17_1)0.9900,(FgGHL17_1,SpoGHL17_1)0.6200)0.5800,(YlGHL17_1,(((DhGHL17_1,SsGHL17_1)0.9900,CaGHL17_1)0.7000,(AgGHL17_1,(KlGHL17_1,(CglGHL17_1,ScGHL17_1)0.7200)0.5500)0.9200)0.8900)1.0000)1.0000);

BAYESIAN TREE

(KfGHL17_1:1.031488,NtGHL17_1:0.6478878,(KfGHL17_2:1.284067,((PmGHL17_1:0.8973442,CgGHL17_1:1.374014):0.2464038,(NtGHL17_2:0.9866501,((((PpGHL17_10:0.221581,PpGHL17_13:0.1868388):0.1245157,PpGHL17_12:0.3857224):0.2587651,(PpGHL17_14:0.1364139,(PpGHL17_15:0.111675,PpGHL17_16:0.08119378):0.1087115):0.3141253):0.115806,((((((AT5G58090:0.3104455,'AT4G31140.1':0.3096722):0.08886421,(OsGHL17_19:0.3354698,OsGHL17_20:0.257651):0.08644777):0.08457154,(AT5G20870:0.342458,OsGHL17_14:0.3479997):0.1976215):0.1376526,((((AT2G19440:0.101169,AT1G64760:0.06308204):0.1473963,(AT3G04010:0.1342862,AT5G18220:0.1593813):0.1861395):0.1194884,(OsGHL17_11:0.3359076,((OsGHL17_13:0.1730749,OsGHL17_21:0.2183986):0.07071446,OsGHL17_15:0.2441407):0.08798612):0.05200727):0.09505165,((AT5G64790:0.4955978,OsGHL17_12:0.3229228):0.04719033,OsGHL17_16:0.451583):0.09545136):0.1186628):0.05429891,AT3G24330:0.5143957):0.0878675,((AT4G17180:0.2011661,(OsGHL17_17:0.1243003,OsGHL17_18:0.1487486):0.1263252):0.2237758,(AT5G58480:0.4448695,OsGHL17_24:0.4092978):0.2953447):0.195037):0.08475673):0.229083):0.1018101):0.1557867,(NtGHL17_3:1.451931,((((CaGHL17_1:0.2792461,(DhGHL17_1:0.2349224,SsGHL17_1:0.2433345):0.1264618):0.07101665,((CglGHL17_1:0.1991189,ScGHL17_1:0.1848496):0.05807429,(AgGHL17_1:0.3214291,KlGHL17_1:0.2058725):0.07271186):0.1326265):0.1132425,YlGHL17_1:0.4153185):0.3419017,(((AcGHL17_1:0.1751123,AfgHL17_1:0.1987274):0.1305707,AnGHL17_1:0.2707218):0.5358066,(FgGHL17_1:1.064288,SpoGHL17_1:0.4226008):0.2262929):0.1032092):1.544895):0.3335915,((((((PpGHL17_1:0.109643,PpGHL17_2:0.1355925):0.5670178,(((('AT4G26830.1':0.1859132,'AT5G55180.1':0.1903609):0.1784638,AT2G05790:0.2722895):0.3821292,((((AT1G32860:0.3599643,AT5G42100:0.4110037):0.272035,(AT2G27500:0.4262786,((OsGHL17_1:0.1670458,OsGHL17_3:0.3021467):0.216281,OsGHL17_2:0.3391436):0.08247595):0.1580908):0.1585223,(AT4G18340:0.3421923,AT1G30080:0.2940851):0.3402503):0.1090464,((AT3G15800:0.3180699,AT2G26600:0.3526178):0.09155361,(OsGHL17_4:0.4279166,OsGHL17_5:0.2257808):0.2032136):0.4838219):0.1321713):0.09711569,((((AT2G16230:0.2034993,AT4G34480:0.1671506):0.1826771,AT5G42720:0.4386958):0.09038645,((OsGHL17_7:0.4827346,OsGHL17_23:0.3450507):0.2269201,OsGHL17_22:0.3240542):0.1214734):0.2564338,((AT3G46570:0.3990325,(AT2G39640:0.5136429,AT3G55430:0.2133267):0.3146755):0.1348286,AT5G24318:0.5075208):0.2199191):0.1872811):0.05199555):0.04823414,(PpGHL17_3:0.5275576,((PpGHL17_9:0.2590513,PpGHL17_11:0.2929861):0.4384024,PpGHL17_17:0.6501676):0.1757492):0.2308739,((PpGHL17_7:0.7325663,((((AT2G01630:0.2254093,AT1G66250:0.2808531):0.0577531,OsGHL17_8:0.2507284):0.09738209,(AT1G11820:0.257071,OsGHL17_10:0.4360389):0.1578334):0.1325845,(AT3G13560:0.3752797,OsGHL17_9:0.4443871):0.1779039):0.1996501):0.07203192,(PpGHL17_8:0.7816582,(('AT5G56590.1':0.2969655,OsGHL17_6:0.2335467):0.08238239,AT4G29360:0.3255049):0.3405335):0.1030652):0.1306085):0.0386467,(PpGHL17_5:0.6278635,(AT3G61810:0.617445,AT3G55780:1.144437):0.2859033):0.1752845):0.07089904,((PpGHL17_4:0.5915651,(PpGHL17_6:0.4680573,PpGHL17_18:0.4507252):0.09219458):0.1565042,(AT3G07320:0.4481729,(AT3G23770:0.1066634,'AT4G14080.1':0.2108284):0.2656685):0.305928):0.1852722):0.04838255,((('AT3G57270.1':0.3613252,('AT3G57260.1':0.2845147,'AT3G57240.1':0.2630875):0.1815072):0.1617902,'AT4G16260.1':0.4664056):0.2402923,(('AT1G77790.1':0.3831816,'AT1G77780.1':0.4653805):0.461838,((((AT5G20340:0.07471666,AT5G20330:0.05666373):0.02661867,AT1G33220:0.07318805):0.06838869,AT5G20560:0.3771053):0.04341315,AT5G20390:0.2255816):0.6265517):0.2069014):0.2246535):0.1988561):0.2011334);

DOMAIN ONLY TREES

NJ TREE

((((((((((((AgGHL17_1:100.0,((CglGHL17_1:100.0,ScGHL17_1:100.0):60.0,KlGHL17_1:100.0):69.0):54.0,(DhGHL17_1:100.0,(SsGHL17_1:100.0,CaGHL17_1:100.0):30.0):34.0):28.0,YlGHL17_1:100.0):97.0,(FgGHL17_1:100.0,((AfgHL17_1:100.0,AcGHL17_1:100.0):100.0,AnGHL17_1:100.0):100.0):38.0):64.0,SpoGHL17_1:100.0):100.0,NtGHL17_3:100.0):46.0,CgGHL17_1:100.0):14.0,((((((PpGHL17_12:100.0,(PpGHL17_10:100.0,PpGHL17_13:100.0):100.0):100.0,((PpGHL17_16:100.0,PpGHL17_15:100.0):100.0,PpGHL17_14:100.0):100.0):88.0,((((((((AT2G19440:100.0,AT1G64760:100.0):100.0,(AT5G18220:100.0,AT3G04010:100.0):100.0):98.0,((OsGHL17_21:100.0,OsGHL17_13:100.0):71.0,OsGHL17_15:100.0):48.0):44.0,OsGHL17_11:100.0):89.0,((AT5G64790:100.0,OsGHL17_12:100.0):95.0,OsGHL17_16:100.0):51.0):99.0,AT3G24330:100.0):53.0,((AT5G20870:100.0,OsGHL17_14:100.0):98.0,((OsGHL17_19:100.0,(OsGHL17_20:100.0,AT5G58090:100.0):64.0):45.0,AT4G31140.:100.0):90.0):95.0):77.0,((OsGHL17_17:100.0,OsGHL17_18:100.0):98.0,AT4G17180:100.0):100.0):38.0):65.0,(AT5G58480:100.0,OsGHL17_24:100.0):100.0):95.0,NtGHL17_2:100.0):58.0,PmGHL17_1:100.0):17.0):3.00,((((((((OsGHL17_7:100.0,OsGHL17_23:100.0):89.0,OsGHL17_22:100.0):99.0,((AT2G16230:100.0,AT4G34480:100.0):100.0,AT5G42720:100.0):100.0):100.0,(((AT3G55430:100.0,AT2G39640:100.0):98.0,AT3G46570:100.0):75.0,AT5G24318:100.0):100.0):77.0,((((((AT2G27500:100.0,OsGHL17_2:100.0):60.0,(OsGHL17_1:100.0,OsGHL17_3:100.0):100.0):100.0,(AT1G32860:100.0,AT5G42100:100.0):100.0):96.0,(AT4G18340:100.0,AT1G30080:100.0):100.0):65.0,((OsGHL17_4:100.0,OsGHL17_5:100.0):91.0,(AT2G26600:100.0,AT3G15800:100.0):53.0):100.0):88.0,((AT5G55180.:100.0,AT4G26830.:100.0):100.0,AT2G05790:100.0):100.0):61.0):33.0,(((((PpGHL17_11:100.0,PpGHL17_9:100.0):100.0,PpGHL17_17:100.0):92.0,PpGHL17_3:100.0):58.0,((PpGHL17_2:100.0,PpGHL17_1:100.0):100.0,PpGHL17_5:100.0):32.0):10.0,((((AT5G56590.:100.0,OsGHL17_6:100.0):61.0,AT4G29360:100.0):100.0,PpGHL17_8:100.0):41.0,((((AT2G01630:100.0,(OsGHL17_8:100.0,AT1G66250:100.0):47.0):100.0,(OsGHL17_10:100.0,AT1G11820:100.0):100.0):100.0,(OsGHL17_9:100.0,AT3G13560:100.0):99.0):100.0,PpGHL17_7:100.0):62.0):35.0):3.00):13.0,((((AT1G77780.:100.0,AT1G77790.:100.0):100.0,((((AT5G20330:100.0,AT1G33220:100.0):56.0,AT5G20340:100.0):96.0,AT5G20390:100.0):69.0,AT5G20560:100.0):100.0):84.0,(AT4G16260.:100.0,(AT3G57270.:100.0,(AT3G57260.:100.0,AT3G57240.:100.0):98.0):96.0):100.0):95.0,(((AT4G14080.:100.0,AT3G23770:100.0):100.0,AT3G07320:100.0):100.0,((PpGHL17_6:100.0,PpGHL17_18:100.0):78.0,PpGHL17_4:100.0):98.0):85.0):28.0):29.0,(AT3G61810:100.0,AT3G55780:100.0):83.0):56.0):7.00,NtGHL17_1:100.0):35.0,KfGHL17_1:100.0):100.0,

KfGHL17_2:100.0);

ML TREE

((((((((((((((((OsGHL17_1,OsGHL17_3)1.0000,OsGHL17_2)0.5900,AT2G27500)0.9700,(AT1G32860,AT5G42100)1.0000)0.9900,(AT4G18340,AT1G30080)1.0000)0.7700,(AT3G15800,(AT2G26600,(OsGHL17_4,OsGHL17_5)0.9700)0.6000)1.0000)0.6600,(AT2G05790,(AT4G26830.1,AT5G55180.1)1.0000)1.0000)0.4900,((((AT2G39640,AT3G55430)1.0000,AT3G46570)0.9800,AT5G24318)0.9900,(((AT2G16230,AT4G34480)1.0000,AT5G42720)0.8200,(OsGHL17_22,(OsGHL17_7,OsGHL17_23)0.8800)1.0000)1.0000)0.8300)0.2500,(PpGHL17_1,PpGHL17_2)1.0000)0.0700,((((AT5G56590.1,OsGHL17_6)0.6700,AT4G29360)1.0000,PpGHL17_8)0.1800,(PpGHL17_7,((AT3G13560,OsGHL17_9)0.9700,((AT1G11820,OsGHL17_10)0.9900,(AT1G66250,(AT2G01630,OsGHL17_8)0.4700)0.9200)0.9700)1.0000)0.5100)0.2600)0.0500,(PpGHL17_5,(AT3G61810,AT3G55780)0.9500)0.2400)0.0400,(PpGHL17_3,(PpGHL17_17,(PpGHL17_9,PpGHL17_11)1.0000)0.7000)0.5600)0.2200,((((PpGHL17_6,PpGHL17_18)0.8000,PpGHL17_4)0.9000,(AT3G07320,(AT3G23770,AT4G14080.1)1.0000)1.0000)0.9700,((((AT3G57260.1,AT3G57240.1)1.0000,AT3G57270.1)0.9400,AT4G16260.1)1.0000,((AT1G77790.1,AT1G77780.1)1.0000,((AT5G20390,AT5G20560)0.5200,(AT5G20330,(AT5G20340,AT1G33220)0.7800)0.9700)1.0000)0.9400)1.0000)0.1600)0.7700,((KfGHL17_2,CgGHL17_1)0.2500,((KfGHL17_1,NtGHL17_1)0.4400,(PmGHL17_1,(NtGHL17_2,((((OsGHL17_17,OsGHL17_18)1.0000,AT4G17180)1.0000,(AT5G58480,OsGHL17_24)1.0000)0.4300,((((PpGHL17_10,PpGHL17_13)0.9400,PpGHL17_12)1.0000,(PpGHL17_14,(PpGHL17_15,PpGHL17_16)0.9900)1.0000)0.8600,(((((AT5G58090,OsGHL17_20)0.6000,OsGHL17_19)0.5200,AT4G31140.1)0.7700,(AT5G20870,OsGHL17_14)1.0000)0.9900,(AT3G24330,(((AT5G64790,OsGHL17_12)0.8800,OsGHL17_16)0.6900,(OsGHL17_11,(((OsGHL17_13,OsGHL17_21)0.5600,OsGHL17_15)0.7100,((AT2G19440,AT1G64760)1.0000,(AT3G04010,AT5G18220)1.0000)0.9800)0.6400)0.9200)1.0000)0.3900)0.6200)0.4100)0.9700)0.6000)0.2400)0.0900)0.0800)0.3100,NtGHL17_3)1.0000,((FgGHL17_1,SpoGHL17_1)0.6200,(((AcGHL17_1,AfgHL17_1)0.9700,AnGHL17_1)0.9900,(YlGHL17_1,(((CaGHL17_1,DhGHL17_1)0.3600,SsGHL17_1)0.8900,(AgGHL17_1,(KlGHL17_1,(CglGHL17_1,ScGHL17_1)0.9800)0.4000)0.8900)0.9500)1.0000)0.4600)1.0000);

BAYESIAN TREE

(KfGHL17_1:0.6759972,KfGHL17_2:0.9821537,NtGHL17_3:0.90014,((PmGHL17_1:0.6184508,CgGHL17_1:1.123791,NtGHL17_2:0.5483034,((((PpGHL17_10:0.1270162,PpGHL17_13:0.0883473):0.08103918,PpGHL17_12:0.2405879):0.2003704,(PpGHL17_14:0.08620577,(PpGHL17_15:0.04957237,PpGHL17_16:0.06027912):0.05992874):0.2138416):0.1047436,((((((AT5G58090:0.1680072,OsGHL17_20:0.1295174):0.05509003,OsGHL17_19:0.189248):0.04815275,'AT4G31140.1':0.1920563):0.0659118,(AT5G20870:0.1461991,OsGHL17_14:0.2269478):0.1238884):0.05837225,((AT4G17180:0.1199674,(OsGHL17_17:0.04837335,OsGHL17_18:0.07192791):0.07998719):0.1518275,(AT5G58480:0.2828222,OsGHL17_24:0.3342873):0.2283809):0.1291336):0.06395308,AT3G24330:0.3473027,(((((AT2G19440:0.03058169,AT1G64760:0.02710663):0.07635127,(AT3G04010:0.07472696,AT5G18220:0.06440295):0.1258144):0.06210208,((OsGHL17_13:0.1355529,OsGHL17_21:0.1217111):0.04204751,OsGHL17_15:0.1738679):0.05504582):0.03060751,OsGHL17_11:0.2232119):0.0585768,((AT5G64790:0.2527345,OsGHL17_12:0.2159103):0.06447427,OsGHL17_16:0.2792724):0.0522127):0.1247552):0.05478908):0.1628832):0.131451,(NtGHL17_1:0.2281719,((((CaGHL17_1:0.1754302,DhGHL17_1:0.1493396,SsGHL17_1:0.1288016):0.07322181,((CglGHL17_1:0.06491683,ScGHL17_1:0.07571782):0.06872949,(AgGHL17_1:0.1954605,KlGHL17_1:0.1188077):0.03325476):0.07664648):0.0947962,YlGHL17_1:0.1976318):0.2408899,((AcGHL17_1:0.07935216,AfgHL17_1:0.1100282):0.07286084,AnGHL17_1:0.1283034):0.3014277,(FgGHL17_1:0.7555211,SpoGHL17_1:0.2831224):0.1635616):1.328792):0.1882343,(((((((PpGHL17_1:0.04326708,PpGHL17_2:0.04364905):0.3373376,(((('AT4G26830.1':0.09367252,'AT5G55180.1':0.09210694):0.1259157,AT2G05790:0.1834106):0.2281058,((((AT1G32860:0.1597793,AT5G42100:0.2235818):0.1469386,(AT2G27500:0.1891062,((OsGHL17_1:0.06407116,OsGHL17_3:0.1404472):0.1280771,OsGHL17_2:0.1626771):0.06102572):0.08787204):0.09613128,(AT4G18340:0.1782456,AT1G30080:0.1442584):0.212571):0.05916175,(AT3G15800:0.1848138,(AT2G26600:0.1935841,(OsGHL17_4:0.2018819,OsGHL17_5:0.09360695):0.1219473):0.05523233):0.2955388):0.03245476):0.07908062,((((AT2G16230:0.09836321,AT4G34480:0.04926358):0.1124191,AT5G42720:0.1701517):0.06335478,((OsGHL17_7:0.190501,OsGHL17_23:0.1686546):0.08548841,OsGHL17_22:0.1576043):0.09424199):0.1908991,((AT3G46570:0.2047675,(AT2G39640:0.3303237,AT3G55430:0.1483443):0.1979492):0.1105942,AT5G24318:0.2672545):0.139118):0.1199812):0.05632443):0.02553606,((PpGHL17_7:0.4943998,((((AT2G01630:0.110953,OsGHL17_8:0.1377076):0.02696754,AT1G66250:0.1535054):0.05990128,(AT1G11820:0.1581071,OsGHL17_10:0.2533634):0.1214514):0.08799741,(AT3G13560:0.2074581,OsGHL17_9:0.2295921):0.1002311):0.1684965):0.09125936,(PpGHL17_8:0.4693795,(('AT5G56590.1':0.1698941,OsGHL17_6:0.102688):0.04222032,AT4G29360:0.146572):0.2504744):0.05355799):0.1000944):0.03228137,(PpGHL17_3:0.3548342,((PpGHL17_9:0.1407967,PpGHL17_11:0.1345552):0.2761628,PpGHL17_17:0.3474025):0.1335187):0.1241456):0.03977359,(PpGHL17_5:0.402355,(AT3G61810:0.3608744,AT3G55780:0.6314111):0.1691243):0.08497957):0.05873139,((PpGHL17_4:0.3331534,(PpGHL17_6:0.2661545,PpGHL17_18:0.3077108):0.07091219):0.1060654,(AT3G07320:0.3056665,(AT3G23770:0.08176392,'AT4G14080.1':0.1328733):0.1576989):0.1739659):0.1260776):0.0570553,((('AT3G57270.1':0.2026473,('AT3G57260.1':0.2006313,'AT3G57240.1':0.1862936):0.0985048):0.1002596,'AT4G16260.1':0.2803323):0.1696136,(('AT1G77790.1':0.2362281,'AT1G77780.1':0.2625758):0.2621971,(((AT5G20340:0.05291306,AT1G33220:0.04963356):0.01861828,AT5G20330:0.03182954):0.05161788,AT5G20390:0.1224817,AT5G20560:0.223312):0.3678078):0.1566303):0.1604308):0.2050121):0.07434213);
