# Supplementary material for: A phylogenetic approach to study the origin and evolution of plasmodesmata-localized glycosyl hydrolases family 17
Source: Front Plant Sci. 2014 May 23;5:212. doi: 10.3389/fpls.2014.00212 (PMC4033164; doi:10.3389/fpls.2014.00212)
Supplement: Supplementary file 4 [file DataSheet4.DOCX]

ARABIDOPSIS AND POPULUS TREE_NEWICK FORMAT

BAYESIAN TREE

(PtGHL17_1:0.07819036,PtGHL17_2:0.1684682,(((((PtGHL17_3:0.1560598,(PtGHL17_4:0.2106077,AT1G66250:0.264096):0.06903101):0.04204635,AT2G01630:0.2346508):0.1139069,(PtGHL17_5:0.1795894,AT1G11820:0.1339627):0.322708):0.1772904,(((PtGHL17_6:0.1579811,'AT5G56590.1':0.2876251):0.1407557,AT4G29360:0.3446818):0.4506757,((((PtGHL17_7:0.1561203,('AT4G26830.1':0.2351374,'AT5G55180.1':0.2226109):0.1028117):0.09998991,((PtGHL17_8:0.06928931,PtGHL17_9:0.07283315):0.1065473,AT2G05790:0.2726965):0.2115124):0.4714504,(((((PtGHL17_10:0.3275507,AT5G24318:0.3812945):0.2164207,(((PtGHL17_13:0.04582558,PtGHL17_32:0.2080954):0.2106389,(PtGHL17_14:0.2553094,(AT2G39640:0.5080885,AT3G55430:0.2429647):0.2823005):0.07826214):0.1715154,(PtGHL17_16:0.1514264,AT3G46570:0.4096796):0.184477):0.1771587):0.2298576,(((PtGHL17_11:0.06023381,PtGHL17_12:0.07299603):0.09856211,((PtGHL17_27:0.07991828,((((PtGHL17_29:0.1200644,PtGHL17_37:0.1278382):0.03700789,PtGHL17_30:0.06915865):0.03395814,((PtGHL17_33:0.0130959,PtGHL17_34:0.05840152):0.03799595,AT5G42720:0.7393964):0.01976546):0.08629835,PtGHL17_31:0.0443394):0.06756233):0.1397048,PtGHL17_28:0.08851517):0.1553023):0.05922116,(AT2G16230:0.2391805,AT4G34480:0.1720745):0.1035198):0.4227015):0.1394991,PtGHL17_22:0.7920059):0.0974185,(((((PtGHL17_17:0.1141068,PtGHL17_19:0.1178584):0.1454705,AT1G30080:0.3119219):0.07652949,AT4G18340:0.4060854):0.4336877,(((PtGHL17_18:0.08516501,PtGHL17_21:0.09455182):0.1855136,(AT1G32860:0.4882891,AT5G42100:0.4970571):0.1121896):0.2998299,(((PtGHL17_24:0.2609476,PtGHL17_26:0.1273969):0.09094975,PtGHL17_54:0.320806):0.1158291,AT2G27500:0.4101899):0.2993021):0.1720305):0.1337649,(((PtGHL17_20:0.1032552,PtGHL17_23:0.1465578):0.1415981,AT3G15800:0.3200294):0.09882207,(PtGHL17_25:0.2080344,AT2G26600:0.318312):0.2227017):0.577329):0.2917004):0.07733526):0.08366623,((((PtGHL17_15:0.2957856,AT3G07320:0.2626368):0.1862942,(AT3G23770:0.1538856,'AT4G14080.1':0.2046567):0.3370516):0.3973092,(AT3G61810:0.6044366,AT3G55780:1.318057):0.3987552):0.1551208,(((((((PtGHL17_35:0.1210003,PtGHL17_42:0.1243051):0.1735138,'AT4G31140.1':0.3094225):0.1175773,(PtGHL17_41:0.1703335,AT5G58090:0.1973827):0.1422353):0.1119018,(PtGHL17_46:0.2437473,AT5G20870:0.3925466):0.244259):0.1689137,(((PtGHL17_40:0.1663935,((AT2G19440:0.1117682,AT1G64760:0.06573314):0.1512912,(AT3G04010:0.1209855,AT5G18220:0.1549025):0.21136):0.05754206):0.1954937,(PtGHL17_47:0.311109,AT5G64790:0.5065061):0.1812659):0.1568811,(PtGHL17_53:0.3267589,AT3G24330:0.335315):0.2797958):0.05204034):0.1090069,((PtGHL17_36:0.1327149,AT4G17180:0.1989439):0.295232,(PtGHL17_39:0.1910441,AT5G58480:0.2712827):0.4686356):0.1985001):0.6926951,(((((PtGHL17_38:0.5768118,PtGHL17_44:0.5315699):0.1354244,('AT1G77790.1':0.5206543,'AT1G77780.1':0.4434403):0.4065598):0.1728703,PtGHL17_50:0.9483687):0.2331876,((((PtGHL17_43:0.4150309,((PtGHL17_48:0.1403734,PtGHL17_51:0.1535622):0.1546075,('AT3G57260.1':0.324217,'AT3G57240.1':0.3213955):0.1779671):0.07950645):0.07270514,'AT3G57270.1':0.436504):0.1986965,(PtGHL17_49:0.3442073,'AT4G16260.1':0.4121975):0.1640205):0.1644095,(PtGHL17_45:0.2825742,PtGHL17_52:0.7240536):0.2205093):0.2724757):0.07146501,((((AT5G20340:0.0748627,AT1G33220:0.1120123):0.01667544,AT5G20330:0.05768726):0.1109068,AT5G20560:0.4199855):0.0559262,AT5G20390:0.2191734):0.715515):0.2213312):0.2677686):0.08088733):0.1294386):0.3320105):0.3209133,AT3G13560:0.2612677):0.2846265);

ML TREE

(((((((((((((((PtGHL17_30,PtGHL17_37)0.4000,PtGHL17_29)0.3100,(PtGHL17_33,PtGHL17_34)0.8900)0.3400,PtGHL17_31)0.9400,PtGHL17_27)1.0000,PtGHL17_28)0.9700,((PtGHL17_11,PtGHL17_12)1.0000,(AT2G16230,AT4G34480)0.9900)0.5100)0.6700,AT5G42720)1.0000,((PtGHL17_10,AT5G24318)1.0000,((PtGHL17_16,AT3G46570)0.9900,((AT2G39640,AT3G55430)0.9000,(PtGHL17_14,(PtGHL17_13,PtGHL17_32)1.0000)0.5300)0.9000)0.9100)1.0000)0.9500,PtGHL17_22)0.3500,((((PtGHL17_20,PtGHL17_23)0.9900,AT3G15800)0.9700,(PtGHL17_25,AT2G26600)1.0000)1.0000,((((PtGHL17_17,PtGHL17_19)1.0000,AT1G30080)0.8100,AT4G18340)1.0000,(((PtGHL17_18,PtGHL17_21)0.9900,(AT1G32860,AT5G42100)0.5000)1.0000,(AT2G27500,(PtGHL17_54,(PtGHL17_24,PtGHL17_26)0.9700)0.7900)1.0000)0.8900)0.7200)0.9800)0.3800,(((AT4G26830.1,AT5G55180.1)0.7300,PtGHL17_7)0.9700,(AT2G05790,(PtGHL17_8,PtGHL17_9)0.9800)1.0000)1.0000)0.5100,(((PtGHL17_6,AT5G56590.1)0.9900,AT4G29360)1.0000,(((PtGHL17_1,PtGHL17_2)1.0000,AT3G13560)1.0000,((PtGHL17_5,AT1G11820)1.0000,(AT2G01630,(PtGHL17_3,(PtGHL17_4,AT1G66250)0.8800)0.5100)1.0000)1.0000)1.0000)0.7200)0.4600,((AT3G61810,AT3G55780)0.9900,((PtGHL17_15,AT3G07320)1.0000,(AT3G23770,AT4G14080.1)1.0000)1.0000)0.4500)0.9400,(((((((((AT2G19440,AT1G64760)1.0000,(AT3G04010,AT5G18220)1.0000)0.7700,PtGHL17_40)1.0000,(PtGHL17_47,AT5G64790)0.8300)0.8600,(PtGHL17_53,AT3G24330)1.0000)0.6600,((PtGHL17_46,AT5G20870)1.0000,((PtGHL17_41,AT5G58090)1.0000,(AT4G31140.1,(PtGHL17_35,PtGHL17_42)1.0000)0.9600)0.9700)1.0000)0.9700,(PtGHL17_36,AT4G17180)1.0000)0.7200,(PtGHL17_39,AT5G58480)1.0000)1.0000,(((((AT5G20340,AT1G33220)0.5500,AT5G20330)0.9200,AT5G20390)0.5600,AT5G20560)1.0000,((((PtGHL17_38,PtGHL17_44)0.5500,(AT1G77790.1,AT1G77780.1)1.0000)0.8500,PtGHL17_50)0.7800,((PtGHL17_45,PtGHL17_52)0.7300,((PtGHL17_49,AT4G16260.1)0.8700,(AT3G57270.1,(PtGHL17_43,((PtGHL17_48,PtGHL17_51)1.0000,(AT3G57260.1,AT3G57240.1)0.9600)0.6500)0.6200)0.9800)0.7300)0.9800)0.4400)0.9900)0.9400);

NJ TREE

((((((((((((((((PtGHL17_40:100.0,(AT1G64760:100.0,AT2G19440:100.0):100.0):65.0,(AT3G04010:100.0,AT5G18220:100.0):100.0):100.0,(PtGHL17_47:100.0,AT5G64790:100.0):100.0):100.0,(AT3G24330:100.0,PtGHL17_53:100.0):100.0):75.0,((((PtGHL17_35:100.0,PtGHL17_42:100.0):100.0,AT4G31140.:100.0):81.0,(PtGHL17_41:100.0,AT5G58090:100.0):100.0):100.0,(PtGHL17_46:100.0,AT5G20870:100.0):100.0):98.0):93.0,((AT4G17180:100.0,PtGHL17_36:100.0):100.0,(AT5G58480:100.0,PtGHL17_39:100.0):100.0):78.0):100.0,(((((PtGHL17_44:100.0,(AT1G77780.:100.0,AT1G77790.:100.0):98.0):76.0,PtGHL17_38:100.0):98.0,PtGHL17_50:100.0):72.0,(AT5G20390:100.0,(((AT5G20340:100.0,AT1G33220:100.0):78.0,AT5G20330:100.0):99.0,AT5G20560:100.0):58.0):100.0):74.0,((PtGHL17_45:100.0,PtGHL17_52:100.0):73.0,((((PtGHL17_43:100.0,(PtGHL17_48:100.0,PtGHL17_51:100.0):100.0):61.0,(AT3G57260.:100.0,AT3G57240.:100.0):95.0):84.0,AT3G57270.:100.0):86.0,(AT4G16260.:100.0,PtGHL17_49:100.0):99.0):57.0):99.0):53.0):94.0,((AT4G14080.:100.0,AT3G23770:100.0):100.0,(AT3G07320:100.0,PtGHL17_15:100.0):100.0):100.0):49.0,(AT3G55780:100.0,AT3G61810:100.0):100.0):57.0,((((((((((PtGHL17_31:100.0,(PtGHL17_30:100.0,(PtGHL17_37:100.0,PtGHL17_29:100.0):68.0):56.0):57.0,PtGHL17_34:100.0):43.0,PtGHL17_33:100.0):56.0,PtGHL17_27:100.0):100.0,PtGHL17_28:100.0):100.0,(PtGHL17_12:100.0,PtGHL17_11:100.0):100.0):79.0,(AT4G34480:100.0,AT2G16230:100.0):67.0):94.0,AT5G42720:100.0):100.0,((((((PtGHL17_18:100.0,PtGHL17_21:100.0):100.0,AT5G42100:100.0):81.0,AT1G32860:100.0):100.0,((PtGHL17_54:100.0,(PtGHL17_26:100.0,PtGHL17_24:100.0):86.0):52.0,AT2G27500:100.0):100.0):90.0,(((PtGHL17_19:100.0,PtGHL17_17:100.0):100.0,AT1G30080:100.0):94.0,AT4G18340:100.0):100.0):53.0,((AT3G15800:100.0,(PtGHL17_20:100.0,PtGHL17_23:100.0):100.0):94.0,(AT2G26600:100.0,PtGHL17_25:100.0):100.0):100.0):91.0):50.0,(((((PtGHL17_32:100.0,PtGHL17_13:100.0):100.0,PtGHL17_14:100.0):98.0,(AT2G39640:100.0,AT3G55430:100.0):98.0):88.0,(PtGHL17_16:100.0,AT3G46570:100.0):100.0):100.0,(AT5G24318:100.0,PtGHL17_10:100.0):100.0):100.0):55.0):32.0,(((AT5G56590.:100.0,PtGHL17_6:100.0):100.0,AT4G29360:100.0):100.0,PtGHL17_22:100.0):42.0):70.0,((AT2G05790:100.0,(PtGHL17_9:100.0,PtGHL17_8:100.0):100.0):100.0,(PtGHL17_7:100.0,(AT4G26830.:100.0,AT5G55180.:100.0):67.0):99.0):100.0):100.0,((((PtGHL17_4:100.0,AT1G66250:100.0):42.0,PtGHL17_3:100.0):72.0,AT2G01630:100.0):100.0,(AT1G11820:100.0,PtGHL17_5:100.0):100.0):100.0):100.0,AT3G13560:100.0):100.0,PtGHL17_1:100.0):100.0,PtGHL17_2:100.0);
